# Emulation of the structure of the Saposin protein fold by a lung surfactant peptide construct of surfactant Protein B

**DOI:** 10.1371/journal.pone.0276787

**Published:** 2022-11-03

**Authors:** Alan J. Waring, Julian P. Whitelegge, Shantanu K. Sharma, Larry M. Gordon, Frans J. Walther

**Affiliations:** 1 Lundquist Institute for Biomedical Innovation at Harbor-UCLA Medical Center, Torrance, California, United States of America; 2 Department of Medicine, David Geffen School of Medicine, University of California Los Angeles, Los Angeles, California, United States of America; 3 Jane & Terry Semel Institute for Neuroscience and Human Behavior, Department of Psychiatry and Biobehavioral Sciences, David Geffen School of Medicine, University of California Los Angeles, Los Angeles, California, United States of America; 4 Materials and Process Simulation Center, California Institute of Technology, Pasadena, California, United States of America; 5 Department of Pediatrics, David Geffen School of Medicine, University of California Los Angeles, Los Angeles, California, United States of America; Rijksuniversiteit Groningen, NETHERLANDS

## Abstract

The three-dimensional structure of the synthetic lung Surfactant Protein B Peptide Super Mini-B was determined using an integrative experimental approach, including mass spectrometry and isotope enhanced Fourier-transform infrared (FTIR) spectroscopy. Mass spectral analysis of the peptide, oxidized by solvent assisted region-specific disulfide formation, confirmed that the correct folding and disulfide pairing could be facilitated using two different oxidative structure-promoting solvent systems. Residue specific analysis by isotope enhanced FTIR indicated that the N-terminal and C-terminal domains have well defined α-helical amino acid sequences. Using these experimentally derived measures of distance constraints and disulfide connectivity, the ensemble was further refined with molecular dynamics to provide a medium resolution, residue-specific structure for the peptide construct in a simulated synthetic lung surfactant lipid multilayer environment. The disulfide connectivity combined with the α-helical elements stabilize the peptide conformationally to form a helical hairpin structure that resembles critical elements of the Saposin protein fold of the predicted full-length Surfactant Protein B structure.

## Introduction

Surfactant Protein B (SP-B) [1] is an essential component for lung surfactant function since mutations can result in fatal lung disease [2, 3]. SP-B is a small 79-amino acid residue, lipid interactive protein that has a conserved antigenic structure for approximately 300 million years [4–6]. SP-B belongs to the Saposin family of proteins (SAPLIP) that carry out diverse functions on a common backbone structure [7, 8]. The high-resolution structures for the Saposin proteins have been determined and their atomistic coordinates have been deposited in the Protein Data Bank (PDB: www.rcsb.org). Some examples of these proteins as autonomous domains include Saposin A, Saposin B, Saposin C, and Saposin D that are involved in lipid catabolism-transfer [9–12] and the defense-oriented proteins Ganulysin and NK-lysin [13, 14].

Lung surfactant contains four surfactant proteins: SP-A, SP-B, SP-C, and SP-D. The hydrophobic surfactant proteins SP-B and SP-C associate with lung surfactant lipids to reduce surface tension and prevent atelectasis of the lower airways, thus guaranteeing continuous exchange of oxygen and carbon dioxide between the alveoli and the capillary network in the lung. Whereas absence and mutations of SP-B are lethal at birth, mutations of SP-C are linked to progressive respiratory failure secondary to familial lung fibrosis [15]. The hydrophilic surfactant proteins SP-A and SP-D provide innate immunity in the lung. Respiratory distress syndrome (RDS) in premature infants is caused by lung immaturity and surfactant deficiency and exogenous surfactant therapy has greatly improved their outcome and survival [16]. Surfactant therapy may also benefit children and adults with acute respiratory distress syndrome (ARDS) secondary to pneumonia [17]. The high costs and limited supply of animal-derived lung surfactant have led to the search for synthetic lung surfactant preparations with SP-B (and SP-C) peptide mimics that can emulate the therapeutic function of native surfactant containing full length SP-B [18]. A recent study in premature infants by Ramanathan et al. has demonstrated the feasibility of this approach [19]. Furthermore, synthetic lung surfactant can also function as a vehicle for optimization of the delivery of pulmonary drugs [20].

**Fig 1:**
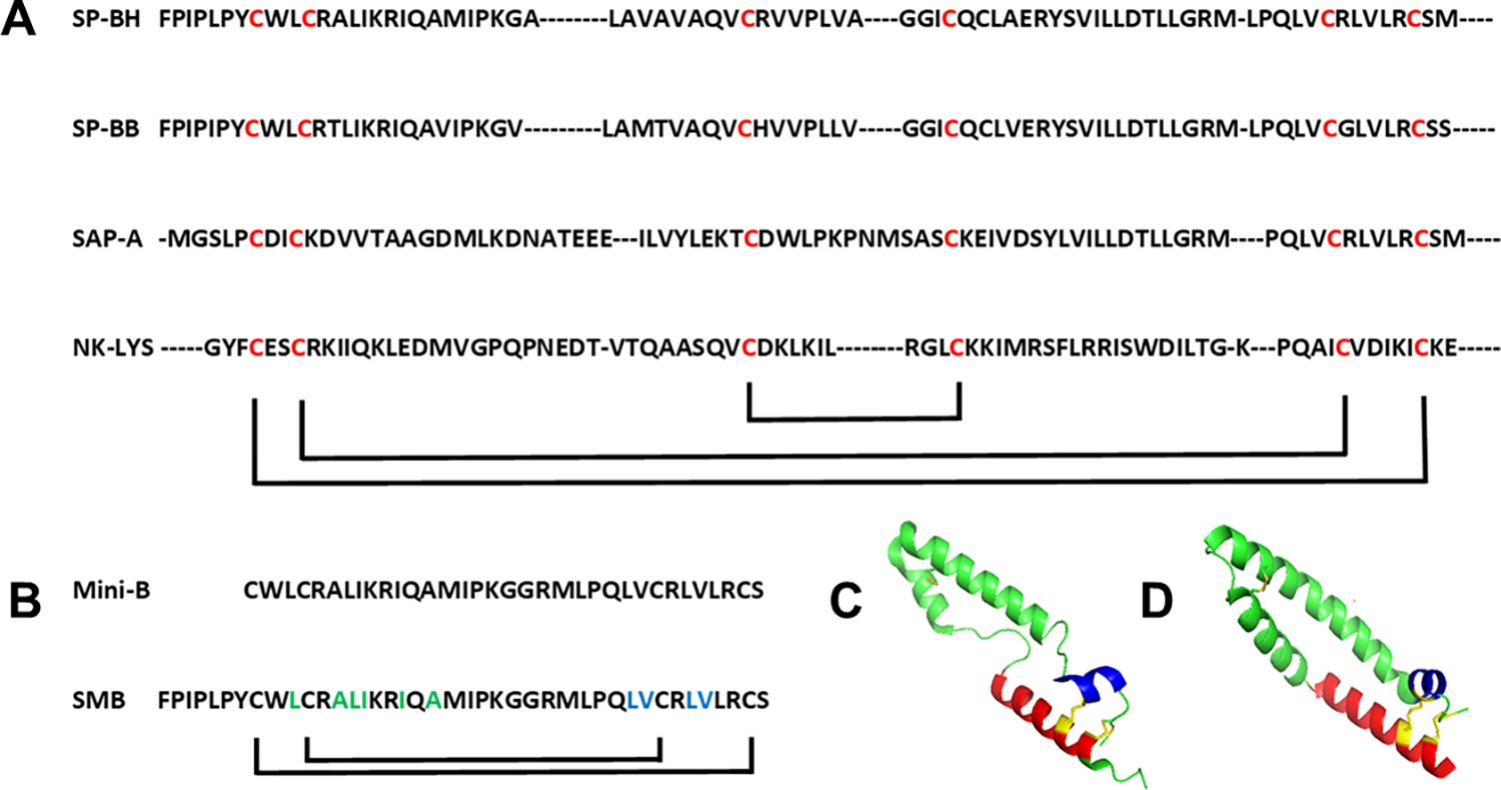
**(A)** Alignment of the partial primary amino acid sequences of surfactant protein B (SP-B human: SP-BH; SP-B Bovine–SP-BB) and two other members of the Saposin family of proteins using Clustal Omega Multiple Sequence Alignment. Small dashed lines in sequence represent non-matching gap sequences in the primary amino acid sequence alignment. (https://www.ebi.ac.uk/seqdb/confluence/display/JDSAT/EMBL-EBI+Web+Services+APIs+-+Data+Retrieval). Conserved cysteine residue alignment is shown in red highlight, while disulfide connectivity is indicated by black lines at the bottom of the sequence alignment. **(B)** Mini-B and Super Mini-B (SMB) peptides amino acid sequence and disulfide connectivity. Mini-B has a disulfide linkage between the N-terminal Cys-1 and Cys-33 as well as Cys-4 and Cys-27, whereas SMB has a similar disulfide pairing with a disulfide linkage between Cys-8 and Cys-40 and between Cys-11 and Cys-34. Amino acid residue carbonyls of SMB peptides enhanced with ^13^C to permit residue specific secondary structure assignments using FTIR are shown in green highlight for the N-terminal domain and in blue highlight for the C-terminal domain. **(C, D)** Ribbon secondary structural illustrations of SP-B bovine monomer theoretical model (PDB: 2IP3; ModelArchive: maca33c) and Saposin A (SAP-A) (PDB: 4DDJ), showing disulfide connectivity highlighted in yellow with the N-terminal helixes in red and C-terminal helixes in blue with the remaining helixes, bends, and disordered domains in green.

Disulfide connectivity of native SP-B and other Saposin proteins is similar and stabilizes the helix-hairpin fold of this family of proteins. At least 10 high resolution residue specific structures in the PDB represent the Saposin protein family. These proteins are a family of lipid interactive proteins that have a similar secondary structure and tertiary folding and carry out diverse functions [7, 8]. Their overall secondary structure is that of a helix hairpin stabilized by specific disulfide linked cysteine pairs. This highly conserved structural format is illustrated in Fig 1A by comparing the primary amino acid sequence of several Saposins. Comparison of the above SAPLIP primary amino acid sequences by CLUSTAL-W reveals that the distribution of cysteine residues and their intramolecular disulfide linkages is highly conserved [21].

Although an experimentally determined high-resolution structure for the SP-B protein is not yet available, the primary amino acid sequence and disulfide connectivity have been characterized by mass spectrometry [22, 23] and the overall secondary structure with circular dichroism (CD) [24–26] and Fourier-transform infrared (FTIR) methodologies [27–29]. Amino acid sequences from SP-B proteins from all species in which amino acid sequences were determined thus far, have the same disulfide linkage pattern between the N-terminal and C-terminal domains. Based on these experimental findings, theoretical models for the protein have been determined by homology templating and refined by molecular dynamics [30, 31]. These models suggest that the protein is a typical member of the Saposin protein family that has well defined amphipathic helical domains that form a helix-hairpin structure stabilized by disulfide connectivity between the N-terminal and C-terminal helical domains and the disulfide linked hydrophobic mid-sequence bend. Similar to all of the Saposin protein family structures analyzed to date, the N-terminal and C-terminal helixes have a disulfide linkage pattern that is the same as illustrated in the three-dimensional (3D) structures for the disulfide connectivity in Fig 1C and 1D, which compare the atomic coordinates of the hypothetical model of monomeric bovine SP-B (PDB:2IL7; Modeling Archive: doi: 10.2210/pdb2IL7/pdb) with that of Saposin A (PDB: 4DDJ). The N-terminal amino acid sequence has 2 cysteine residues that are separated by 2 amino acids in the sequence, whereas the C-terminal sequence has 2 cysteines separated by 5 amino acids. The N-terminal and C-terminal disulfide linkage between the cysteines of these domains also has specific pairing. The cysteines closest to the termini form one disulfide pair, whereas the more distal cysteines constitute the second disulfide pair.

Recent all-atom simulations of covalently linked, full length homo-dimeric SP-B in surfactant lipid bilayers help validate the presence of stable, helical domains, disulfide linkages, and flexible bends that optimize the protein’s functional interaction with surfactant lipids [32]. This distinct Saposin disulfide structure may also, at least in part, contribute to the notable structure-function stability of the Super Mini B (SMB) peptide (Fig 1B) in surfactant lipid multilayers that lasts for over five years without detectable chemical degradation and peptide aggregation [33]. However, SMB peptide does have some concentration dependent ß-sheet propensity in synthetic surfactant lipid multilayers. This ß-sheet formation is most likely associated with the so-called insertion sequence that spans the N-terminal seven residues consisting of multiple proline residues interspersed between hydrophobic amino acids, as shown by isotope edited FTIR of this sequence [34]. Surface plasmon resonance measurements that compare the binding of SMB with that of Mini-B that lacks the N-terminal seven residue insertion sequence (Fig 1B), indicate self-association based binding of SMB to this domain [35]. Since ß-sheet segment hydrogen bonds pair to optimize structure, there is a strong possibility that SMB may form non-covalent dimers in surfactant films since dimer formation is observed in SDS gels [35].

The structural determination strategy for the present study has been to identify a few specific key residue elements of the peptide of interest and then using this information to construct a model of the peptide that closely resembles the predicted monomeric structure in a synthetic lipid bilayer environment. We describe in detail the secondary structure of the two α-helical domains that encompass the disulfide stabilized surfactant peptide construct Super Mini-B (SMB) and resemble the Saposin fold of the full-length human, monomeric SP-B protein. The two domains are represented by the SMB peptide, a disulfide stabilized helix-hairpin construct of the N-C terminal helical domains linked by a bend sequence. These two helical domains are stabilized by intra-molecular disulfide linkages to form a peptide construct that emulates the Saposin fold by conservation of the disulfide connectivity between the N-terminal domain and the C-terminal domain of the full-length protein (Fig 1B). This Saposin like construct has been folded into a stable structure by disulfide formation with region-specific structure promoting solvents [36]. The secondary structure of the disulfide stabilized peptide was then determined by mapping the residue level structure using isotope edited FTIR spectroscopy [37] and the directed disulfide connectivity was detailed with high resolution mass spectrometry [38].

## Materials and methods

### Rationale for the peptide structure study

A minimal experimental structural determination strategy for the present study was to identify several specific key structural elements of the peptide of interest and then using this information to construct a model of the peptide that closely resembles the monomeric structure of the peptide in a surfactant multilayer environment. This was accomplished by first using predictive modeling based on homology modeling and artificial intelligence approaches. Then using these predictive models as a guide to experimental measures, the disulfide connectivity of the peptide was confirmed by high resolution mass spectrometry [39–42]. The predicted helical segments of the sequence were then confirmed through specific residue labeling by preparing “cassettes” of multiple ^13^C carbonyl labeled amino acids corresponding to the predicted helical domains [32, 34, 43]. Since the stretching frequency of the labeled carbonyl oscillators is shifted in the infrared spectrum of the amide I band by approximately 35 to 40 wavenumbers, one can identify the amino acid residues participating in a given conformation. Finally, using the experimentally determined conformational information as connectivity-distance constraints, the initial modeled structure was then refined with molecular dynamics in a relevant simulated molecular environment to determine a relatively accurate “medium resolution” model of the peptide [37].

### Materials

All solvents used in the synthesis, purification, and folding of the SMB peptide were HPLC grade or better and were purchased from Fisher Scientific (Waltham, MA 02451). Trifluoroethanol (TFE) was obtained from Millipore-Sigma (Saint Louis, MO 63103), synthetic phospholipids were supplied by Avanti Polar Lipids (Alabaster, AL 35007), and carbonyl ^13^C isotope enhanced Fmoc amino acids were obtained from AnaSpec (Fremont, CA 94555).

### Synthesis and folding of Super Mini-B peptides

SMB peptides were synthesized as detailed previously [35]. The suite of isotopically enhanced SMB peptides, shown in Fig 1, were synthesized using standard Fmoc procedures with a H-Ser (OtBu)-HMPB NovaPEG resin (Millipore-Sigma, St. Louis, MO 63103) using a Liberty Microwave Peptide (CEM Corp, Matthews, NC 28104) synthesizer. The SMB surfactant peptides were double coupled for all residues to insure optimal yield. Peptides were cleaved from the resin using the standard phenol:thioanisole:ethanedithiol:water:trifluoroacetic acid (0.75:0.25:0.5:0.5: 10, v:v) cleavage-deprotection mixture [35]. Crude peptides were then purified to better than 95% with a Jasco preparative HPLC (Easton, MD 216010), using a VYDAC diphenyl or C8 (1” by 12” width by length) column at 20 mL/min. The SMB peptide was eluted from the column using 0 to 100% ACN (water to acetonitrile) with 0.1% trifluoroacetic acid as an ion pairing agent added to both aqueous and organic phases with a linear gradient in 1 h. The purified product was freeze-dried and the mass confirmed by Maldi TOF mass spectrometry. The region-specific disulfide formation of the SMB peptide to form disulfide bonds between Cys-8 and Cys-40 and between Cys-11 and Cys-34 by air oxidation was then facilitated by dissolving peptide at a concentration of 0.4 mg/mL in an aqueous-organic structure promoting solvent system of 40% TFE by volume and 60% by volume with 50 mM phosphate-buffered saline (PBS) adjusted to pH 7.5. Alternatively, the peptide was dissolved in TFE with the oxidant dimethylsulfoxide (DMSO) and 50 mM PBS pH 7.5 (TFE:DMSO:PBS, 3:1.5:5.5, v:v) to mediate oxidation of the thiols to disulfides [36]. The peptide-aqueous-organic solvent solutions were stirred for 48 h at 25°C to ensure complete disulfide formation before concentration of the peptide by Speed-Vac^**®**^. Purification of the oxidized SMB by HPLC was performed as described above for the crude product and the oxidized peptide disulfide linked molecular mass was confirmed by Maldi-TOF mass spectrometry. Trifluoroacetate counter ions were removed from all peptide samples prior to the FTIR measurements by repeated suspension in HCl, followed by freeze drying to prevent residual acetate counter ion interference with infrared spectroscopy as detailed previously [28, 44]. Peptide concentration was determined by the UV spectrometry method of Anthis and Clore [45].

### Circular dichroic characterization of SMB with a similar disulfide linked peptide

To gain insights into the unique secondary structure of the disulfide stabilized SMB peptide, the overall secondary structure of oxidized SMB was compared to the closely related high-resolution solution nuclear magnetic resonance (NMR) structure of Mini-B [46] (PDB accession code: 2DWF) in sodium dodecyl sulfate (SDS) anionic detergent micelles. CD spectra of the oxidized SMB and Mini-B in the SDS-buffer structure-promoting environment were measured with a Chirascan V100 spectropolarimeter (Applied Photophysics, Surrey KT22 7BA, UK). This instrument was routinely calibrated for wavelength and optical rotation using 10-camphorsulphonic acid [47]. The sample solutions of the anionic detergent SDS were scanned using 0.01 cm pathlength cells at a rate of 50 nm per minute, a sample interval of 1 nm, and a temperature of 37°C. Peptide concentration was 100 μM in a sample solution of SDS:PBS buffer (20 mM detergent:10 mM buffer, pH 7.5) scanning at a wavelength range of 185 to 350 nm. Sample spectra were baseline corrected by subtracting spectra of peptide-free solution from that of the peptide containing solution and expressed as the Mean Residue Ellipticity [θ]_MRE_ as shown in Eq (1):

$${\left[\theta \right]}_{MRE}=([\theta ]\;x\;100)/(l\;x\;C\;x\;N)$$ (1)

The symbol θ is the measured ellipticity in millidegrees, l is the pathlength in cm, N is the number of residues in the peptide, and C is the concentration of the peptide in mM.

CD spectra were scanned in the wavelengths spanning 250 to 350 nm that are sensitive to disulfide bond formation and chirality [48, 49]. This chiral determination compared the known disulfide connectivity of the Mini-B peptide, determined by high-resolution solution NMR [46], with the SMB peptide structure to confirm both the formation and chiral connectivity was similar for both peptides.

### Mass spectrometry of intact SMB and peptides following pepsin digest for disulfide connectivity

Intact protein mass spectrometry was performed using liquid chromatography with positive-ion electrospray ionization mass spectrometry (LC-MS), as described previously [48–51]. In this case, SMB was dissolved in formic acid (100 μL; 90% Fisher) for immediate injection to size-exclusion chromatography in ‘441’ (chloroform/methanol/ 1% aqueous formic acid; 4/4/1, v/v) at 40˚C using a silica-based matrix (4 mm x 15 cm; SW2000; Tosoh Bioscience Inc, South San Francisco, CA 94080), as described previously [42]. Mass spectrometry was performed using a Finnigan LTQ ion-trap MS (Thermo Fisher Scientific, Waltham, MA 02451) and MagTran 1.1 freeware was used to deconvolute spectra. Average mass was used for the intact protein data. Pepsin treatment was performed in aqueous HCl at 37°C. SMB peptide in water (100 μg, 10 μL) was acidified with HCl (100 μL, 0.04 N) and pepsin added (10 μL, 1 mg/mL). After 4 h, the mixture was analyzed by LC-MS. Peptide MS was performed using reverse-phase chromatography on a polymeric support at 40°C (PLRP/S, 5 μm x 300 Å; 2 x 150 mm; Agilent, Santa Clara, CA 95051) and an orbitrap high-resolution mass spectrometer (Orbitrap XL; Thermo Scientific, San Jose, CA 95134) operated at 100,000 resolution at m/z 400. The column was equilibrated at 95% A, 5% B (A: 0.1% formic acid in water; B: 0.1% formic acid in acetonitrile/isopropanol, 1:1) for 20 min prior to loading the sample and initiating a gradient from 95% A at 5 minutes to 98% B at 55 minutes) at a flow rate of 120 μL/min [41]. Detailed mass spectra data have been deposited in the ModelArchive database (https://modelarchive.org/doi/10.5452/ma-axqi2) (S1 File).

### Attenuated Total Reflection-Fourier Transform Infrared (ATR-FTIR) spectroscopy

Infrared spectra were recorded at 37°C using a Bruker Vector 22 FTIR spectrometer (Bruker Optics, Billerica, MA 01821) with a deuterium triglyceride sulfate (DTGS) detector, signal averaged over 256 scans per spectra at a gain of 4 and a resolution of 2 cm^-1^. For FTIR spectra of the SMB peptide in synthetic surfactant lipid multilayers, the peptide in TFE was co-solvated with lipid systems dissolved in chloroform (lipid:peptide mole ratio of 10:1) and transferred onto an ATR crystal (Pike Technologies, Madison, WI 53719). The organic solvent was then removed by flowing nitrogen gas over the sample to produce a lipid-peptide film. The lipid-peptide films were next hydrated (≥ 35%) with deuterium vapor in nitrogen for 1 h prior to acquiring spectra [52] with PBS pH 7.5. The pH for the deuterium buffer solutions was determined using the correction for pD as detailed by Glasoe [51]. The spectra for peptide in lipid mixtures were obtained by subtracting the lipid spectrum with D_2_O buffer from that of peptide in lipid with D_2_O hydration. Infrared spectra were subsequently recorded for the lipid-peptide films at 37°C.

### Prediction of SMB preliminary structure using homology modeling and neural network structure approximation followed by molecular refinement based on mass spectral and isotope enhanced FTIR experimental data using constrained molecular dynamics

An initial structure for SMB used for mapping of the peptide conformational domains was approximated by template homology modeling of the peptide [53] as well as by neural network-based protein structure prediction that uses machine learning by incorporating multi-amino acid sequence alignments into the design of the deep learning prediction algorithm [54].

A preliminary 3D structural model for SMB was determined by analysis of the peptide amino acid sequence with version 5.1 of the I-TASSER program [52, 55], using the automated I-TASSER web servicer (http://zhanglab.ccmb.med.umich.edu/I-TASSER) [56]. I-TASSER is a homology algorithm that models specific regions of the protein using the molecular coordinates of multiple PDB depositions. In addition to the amino acid sequence of SMB, disulfide distance constraints were imposed on the sequence to link Cys-8 with Cys-40 and Cys-11 to Cys-34. The output for a predicted 3D protein structure was a PDB file, and the accuracy of these models was estimated using such parameters as C-score that is a confidence score for estimating model quality, while TM-score and RMSD are measures of the accuracy of the predicted models compared with native structure (Fig 4A). This homology templated model of the peptide had the N-terminal helical axis oriented at approximately 15° to that of the C-terminal axis.

An alternative predictive protein structure approach using AlphaFold artificial intelligence (AI) software [54] without cysteine disulfide constraints was also utilized to estimate the initial secondary structure of the SMB peptide. The AlphaFold program was run through the Chimera X (version 1.3) molecular modeling system environment [57], available at https://www.cgl.ucsf.edu/chimera/docs/relnotes.html. In this AI based predictive molecular mode of the SMB peptide, the α-helical axis of the N-terminal secondary structural domain is close and parallel to that of the C-terminal helix (S2 File).

The above predicted structure for SMB was modeled as a monomer to serve as a template for subsequent ^13^C amino acid residue isotope edited versions of the peptide to map the helical domains. The initial peptide model was oriented in a synthetic surfactant lipid bilayer with the OPM database and PPM web server [58]. The resulting oriented peptide was then uploaded to the Charmm Membrane Builder (http://www.charmm-gui.org/?doc=input/m embrane.bilayer) [59, 60] and disulfide bonds were added between Cys-8 and Cys-40 and Cys-11 and Cys-34 to emulate the oxidized version of the SMB peptide. The peptide was inserted into a lipid bilayer having the same proportions of lipid molecular species (64 lipids per monolayer leaflet) as the experimental formulation using the lipid replacement method. The lipid-peptide ensemble was then placed in a 69.19 x 69.12 x 87.00 Å simulation box and hydrated with 2160 TIP3 waters and potassium ions were added to render the solution electrically neutral. The simulation box was then downloaded from the Charmm GUI website server using the Gromacs simulation option to set up the system for equilibration and production runs employing molecular dynamics (MD) simulation structural refinement.

MD simulations were carried out using the Charmm 36m all atom force field implementation for lipids and proteins in the Gromacs (Version 2021.5) environment (http://www.gromacs.org). The system was first minimized using a steepest descent strategy followed by a six-step equilibration process at 311 K for a total of 100 or 500 ns. This included both NVT (constant number, volume, temperature) and NPT (constant number, pressure, temperature) equilibration phases to allow water molecules to reorient around the lipid headgroups and any exposed parts of the peptide, as well as permitting lipids to optimize their orientation around the peptide. Equilibration protocols employed a PME (Particle Mesh Ewald) strategy for Coulombic long-range interactions and Berendsen temperature coupling. A Berendsen strategy was also used for pressure coupling in a semi-isotropic mode to emulate bilayer motion. After equilibration, the system was subjected to a dynamics production run at the same temperature using the Nose-Hoover protocol and pressure (Parrinello-Rahman) values used in the pre-run steps. The Verlet cut-off scheme was employed for all minimization, equilibration, and production steps. Detailed protocols and parameter files for this type of membrane simulation are available from the Charmm-GUI website (http://www.charmm-gui.org). The output of the production run simulations was analyzed with the Gromacs suite of analysis tools.

The structural quality of the molecular dynamics refined structures was analyzed by Procheck [57, 61] generated with PDBsum (https://ebi.ac.uk). Molecular graphics were rendered using Pymol Version 2.2.3. SMB disulfide linkages were analyzed using the disulfide bond dihedral angle energy server (https://services.mbi.ucla.edu) that is based on estimates derived from formulas detailed in Katz et al. [62] for comparison with disulfide bonds of Saposins available in the PDB [63]. Analysis of helical geometry and orientation was determined using HELANAL [64]. The coordinates for the lowest energy conformer of oxidized SMB have been deposited in the ModelArchive (S3 File).

## Results

### Region specific folding and disulfide formation

Peptide folding and time course of disulfide formation were compared using two different structure-promoting, disulfide forming solvent systems [36]. TFE has been used extensively to facilitate α-helical and turn secondary structure in peptides [65, 66]. The peptide was dissolved in a solvent system of TFE and phosphate buffer that was stirred in the presence of oxygen-room air at neutral pH and monitored as a function of time for a total period of 48 h. TFE-buffer air oxidation of the peptide to better than 95% completion of oxidation of the sample was observed to require at least 24 h.

DMSO has also been shown to enhance the rapid formation of intra and inter disulfide bonds between cysteine residues in proteins and peptides [36]. To promote helix formation and enhance disulfide formation, a mixture of TFE and DMSO was used to compare the rate of formation of a disulfide stabilized helical hairpin peptide with that of the TFE air-oxygen solvent system. The addition of 15% DMSO to the structure promoting TFE-neutral pH buffer system enhanced the rate of oxidation of the thiols to disulfides by reducing the time to complete the oxidation to 95% in 12 h.

**Fig 2:**
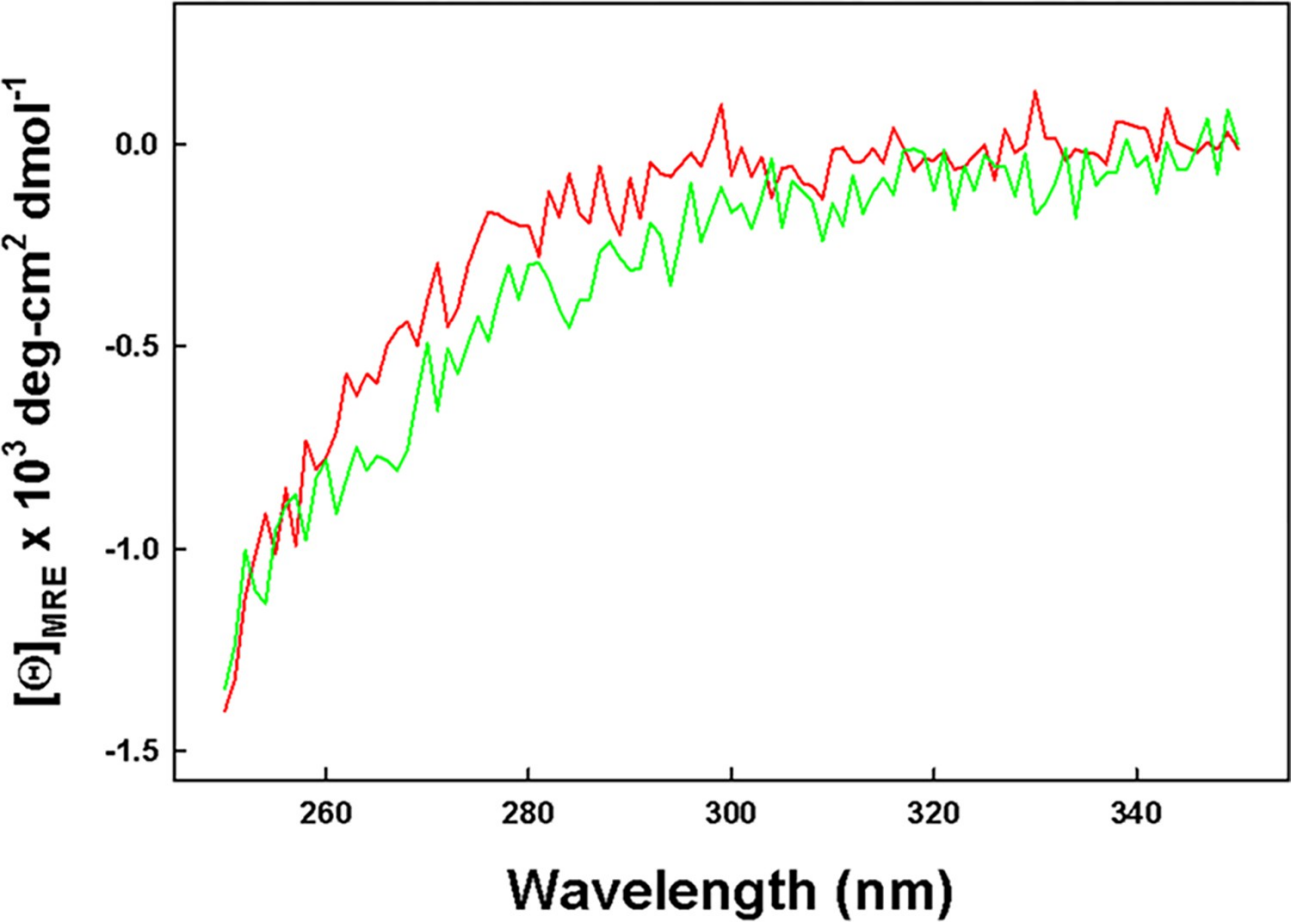
Comparison of the circular dichroism spectra of Mini-B (red line) and SMB (green line) in SDS micelles for the disulfide spectral region (260nm to 350 nm). Raw data are available in S1 File.

In addition to the initial mass spectral observation that there is a definitive mass shift consistent with the disulfide formation of both cysteine pairs, circular dichroism measurements of the oxidized peptides were made to identify the formation of disulfide linkages. Disulfide bond formation in proteins and peptides has been shown to be associated with the circular dichroic spectral region from 260 to 350 nm [48, 49]. The CD spectra for disulfide linked Mini-B and SMB show a similar absorbance in the 260 nm to 350 nm range for oxidized peptide samples (Fig 2) suggesting that the peptides with a similar amino acid sequence have a similar disulfide conformational connectivity.

**Fig 3:**
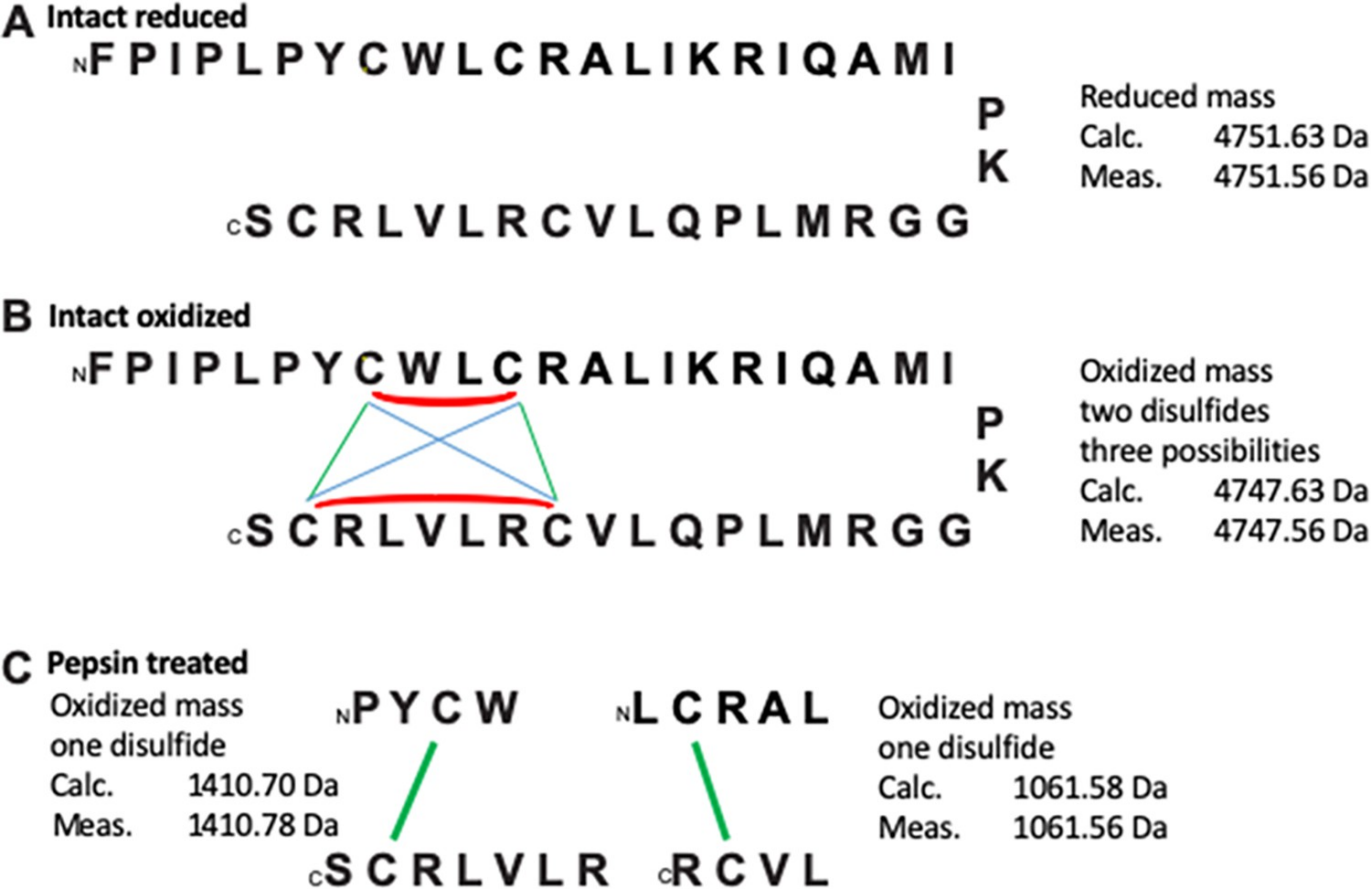
Illustration of mass spectral data of SMB peptide prepared by oxidation of reduced SMB peptide by solvent mediated region-specific disulfide formation. **(A)** reduced SMB peptide, **(B)** oxidized SMB in 40% TFE, 60% PBS or 30% TFE, 15% DMSO, 55% PBS buffer for 48 h, **(C)** pepsin treated oxidized SMB. Monoisotopic masses are given. Detailed mass spectral data, including experimental data collection parameters, and spectral data are found in S1 File.

Although it is highly unlikely that the disulfide bridges formed by solvent mediated folding would be mis-paired because of potential steric hinderance, we further examined the samples by selective protease digestion of the oxidized peptide samples to confirm there was no disulfide mismatch. Mass spectrometry was used to confirm the presence of two disulfides and the crosslinking locations. The monoisotopic mass of reduced SMB agrees with its primary sequence (Fig 3). Oxidation of SMB in air is spontaneous (either using a TFE-air oxygen protocol or a TFE-DMSO protocol) linking 4 cysteine residues in a pair of disulfides, though the intact SMB mass does not define which of three disulfide crosslinking possibilities occurs (Fig 3). Treatment with the proteolytic enzyme pepsin at low pH is favored because disulfides are stable under these conditions, and the cleavage specificity allows discrimination of specific crosslinks in the SMB structure. At a pH of 1.4, pepsin cleavage is nearly exclusively restricted to the C-terminal side of Phe and Leu residues, and we used these conditions for cleavage of oxidized SMB. Two crosslinked peptide pairs were recovered, one with the Cys-8 to Cys-40 crosslink and the other with the Cys-11 to Cys-34 crosslink (Fig 3). These crosslinks are homologous to those observed for the full-length native SP-B protein [22, 23].

### Preliminary secondary structure prediction using homology and AI based methodology

**Fig 4:**
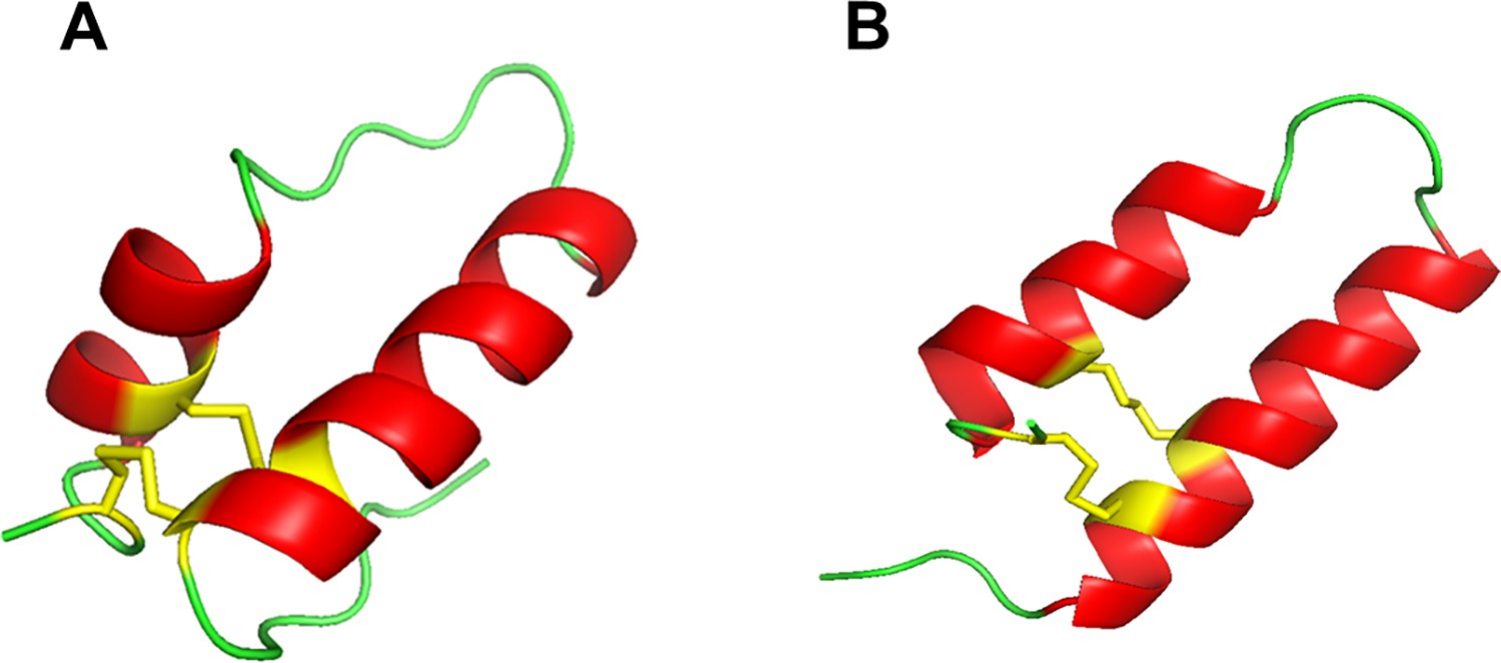
**(A)** Predicted structure of SMB using I-Tasser [52, 56]. The α-helical domains are highlighted in red ribbon, while bend and disordered segments are in green tube illustrations. Disulfide connectivities are colored in yellow. The N-terminal helix spans from Cys-8 to Met-21, while the C-terminal helix is from Pro-30 to Val-37. The C-score for the model is -0.63 falling in the -5 to 2 range of a good model of high confidence and the TM-Score of 0.63 +/- 0.13 and RMSD score of 3.4 +/- 2.4 Å indicate a good quality protein structure (data in S1 File). **(B)** SMB structural model predicted with the AlphaFold [54]. N-terminal helical domain spans residues Leu-5 to Met-21 and the C-terminal helical sequence that includes Arg-27 to Cys-38 is highlighted in red ribbon with the disulfide linkages in yellow. Bend and disordered domains are represented as green tubes with the coordinates deposited in S2 File. The full-length human SP-B AlphaFold predicted structure (S5 File) is highlighted in green and is overlayed on the predicted SMB structure. The global RMSD value for the backbone atoms of SMB residues 1–25 and 26–41 compared with SP-B residues 1–25 and 63–78 is 4.36 Å (Superpose–webserver: http://superpose.wishartlab.com).

Initial estimates of the secondary structure of the SMB peptide with homology modeling using the I-Tasser suite of programs characterized the peptide as a well-defined helix hairpin joined by a bend domain. There were two α-helical segments, one in the N-terminal domain from Tyr-7 to Met-21 and the other in the C-terminal peptide segment from Pro-30 to Leu-37 that were stabilized by a single disulfide bond between Cys-11 and Cys-34. However, the experimental analysis using high resolution two-dimensional solution NMR spectroscopy, described in the previous section, indicated that there was one additional intra-molecular disulfide linkage between Cys-8 and Cys-40. Based on this experimental observation, the I-Tasser model prediction was refined using the programs distance constraint option by imposing a 2.05 Å distance between the sulfur atoms of Cys-8 and Cys-40. The resulting model is shown in Fig 4A is consistent with that of a hairpin like structure with N-terminal and C-terminal α-helical segments stabilized by two disulfide linkages. The N-terminal helix spans from Tyr-7 to Met-21 and the C-terminal domain has a continuous helix from Pro-30 to Leu-37 that indicates about 55% total α-helix, like the percent determined experimentally for the peptide in SDS micelles. It is of interest that the disulfide stabilized helical axis orientation of the N-terminal helix to that of the C-terminal helix of approximately 15 degrees is typical of that observed for many Saposin structures determined by X-ray crystallography [10, 12, 13].

An alternative approach to obtaining a preliminary secondary structure for the SMB peptide was also considered. The AlphaFold AI suite of programs [54] for secondary protein structure prediction provided additional insights into the helix-hairpin conformation of the SMB peptide. Although the predicted overall structure was like the homology templated structure, the α-helical segments were longer with the N-terminal helix spanning from Leu-5 to Met-21 and the C-terminal helix segment from Met-28 to Leu-36. The AI based methodology predicted the correct pairing of both disulfide bonds connectivity without having to impose distance constraints required with the homology templating program. However, there was a second noticeable structural difference observed using the AI predictive approach. The two α-helical domains were almost parallel to one another (Fig 4B) with a mean helical axial angle of approximately 5 degrees. This α-helical axis orientation of the disulfide linked N-terminal–C-terminal helical domains is more typical of Saposin protein structures determined in anionic micelles by high-resolution solution NMR [9, 11, 14, 46].

### Conformational mapping of the SMB peptide α helical domains

**Fig 5:**
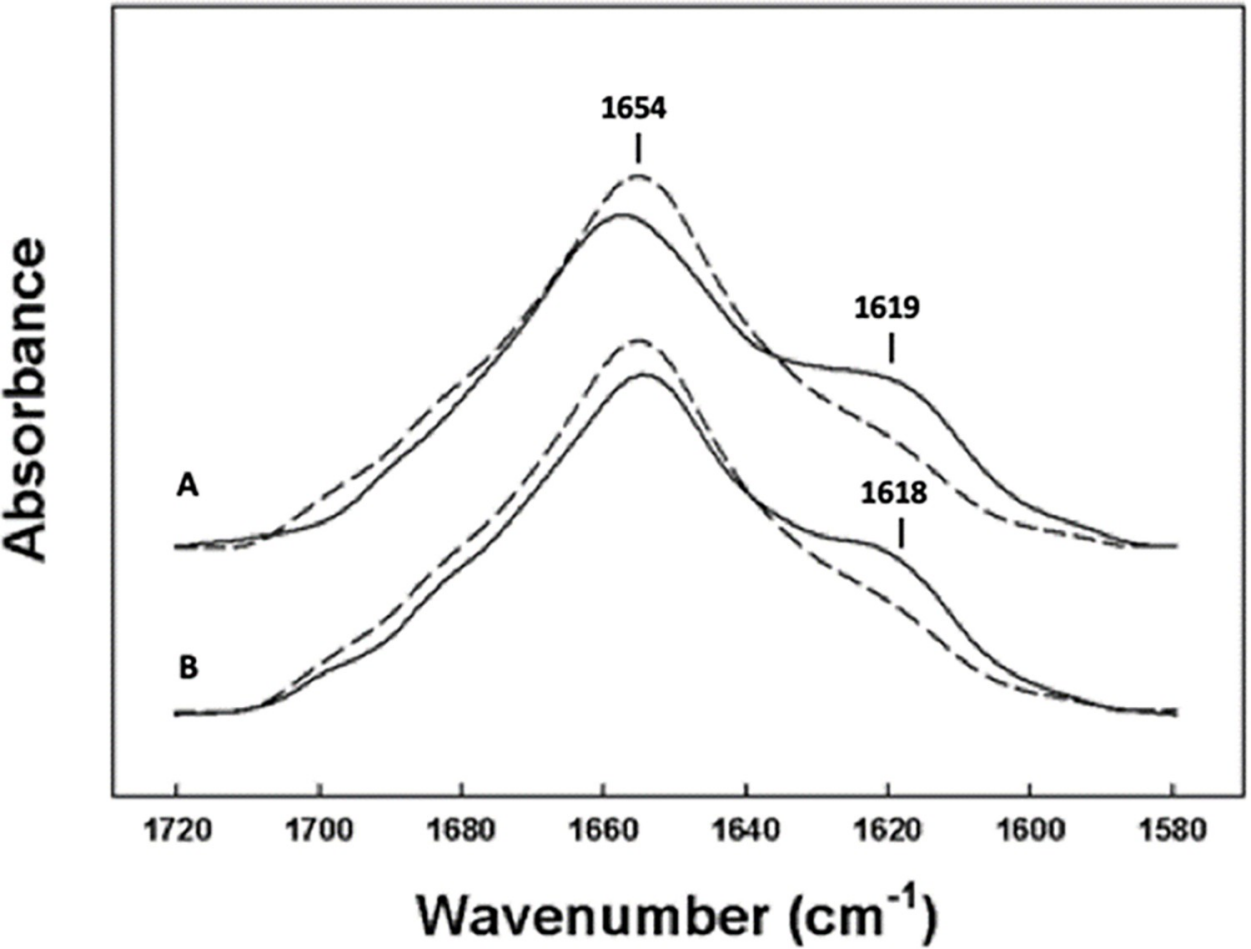
FTIR spectra of the amide I band for **(A)** ^12^C = O (non-isotopically enhanced amino acids) SMB peptide with dashed line compared with ^13^C = O N-terminal helical domain of labeled SMB (^13^C amino acid residues: L_10_/A_13_/L_14_/I_15_/I_18_/A_20_) solid line. **(B)** ^12^C = O (non-isotopically enhanced amino acids) SMB peptide with dashed line compared with ^13^C = O C-terminal helical domain labeled SMB (^13^C amino acid residues: L_32_/V_33_/L_36_/V_37_) solid line. Peptides were co-solvated with surfactant lipids (DPPC:POPC:POPG, 5:3:2 mole:mole) at a ratio of peptide to lipid of 1:10 mole:mole and dried onto the germanium ATR sample plate with a stream of dry nitrogen gas. The dry peptide-lipid film was then hydrated by passing nitrogen gas saturated with D_2_O for one hour before spectral measurements. FTIR spectral data are deposited in S3 File.

Isotope edited versions of SMB were used to make residue specific assignments for structural refinement of the SMB peptide in synthetic surfactant multilayer lipid films. The N-terminal domain of SMB was labeled with ^13^C carbonyl amino acid residues from Leu-10, Ala-13, Leu-14, Ile-15, Ile-18, and Ala-20 (Fig 1B). Measurements of this isotope enhanced peptide and comparison with unlabeled SMB allowed assignment of a well-defined secondary structure to this domain. The amino acid residues spanning position Leu-10 to Ala-20 were identified as α-helix as indicated by the spectral shift of ~35 wavenumbers from 1654 cm^-1^ of the unlabeled peptide spectra (Fig 5A) to 1619 cm^-1^.

The local α helical conformation of the C-terminal segment was also confirmed using the isotope enhanced version of the peptide and included amino acid residues from Leu-32 to Val-37 (Fig 1B). There was a decrease in the α helical peak around 1654 cm^-1^ with a concurrent increase in absorption near 1618 cm^-1^, indicating a spectral shift of 36 wavenumbers typical of residues participating in α helical conformations (Fig 5B).

In general, the rather broad absorbance for the isotope-enhanced helical amino acid segments observed in these spectra indicates that these sequences may include some disordered helix residues that are associated with residues undergoing disordered helical conformations that are typical of amphipathic helix fraying near the termini of this type of secondary structure. The FTIR spectra also indicate there is a small proportion of ß-sheet formation in the samples and may indicate that the sample has some self-association of the peptide to form non-covalent dimeric ensembles in the lipid films at the concentration used for these determinations [35].

### Molecular dynamics refinement of SMB peptide structure based on MS and FTIR residue specific experimental data

**Fig 6:**
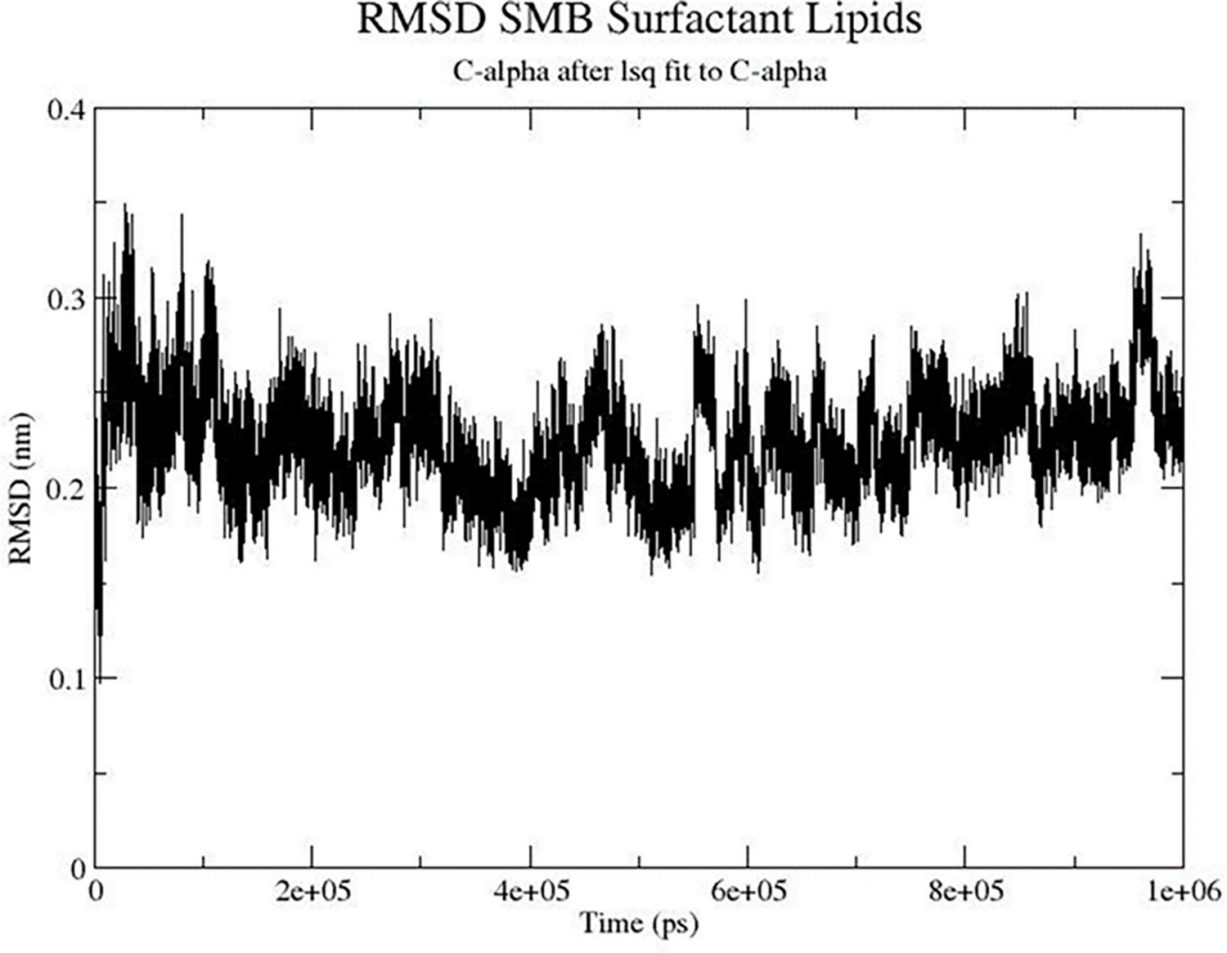
RMSD plot of the SMB peptide backbone as a function of time in a bilayer of synthetic surfactant lipids.

Combining the experimental data on disulfide pairing and ^13^C residue specific conformational mapping of N-terminal and C-terminal α-helical domains permitted a detailed, refined model of the SMB monomeric peptide in a synthetic surfactant lipid film. Using the above experimental determinations as distance constraints, the homology modeled preliminary SMB structure was inserted into a bilayer of DPPC: POPC: POPG (5:3:2 mole: mole), followed by solvation with water and subjecting the ensemble to a full microsecond of molecular dynamics to insure equilibration of the peptide in the lipid. Examination of the peptide backbone as a function of time is shown in Fig 6. There is a rapid change in the C-α carbons in the first few nanoseconds, followed by a plateau in the RMSD values for the remainder of the simulation suggesting an equilibrium state for the peptide structure in this environment.

**Fig 7:**
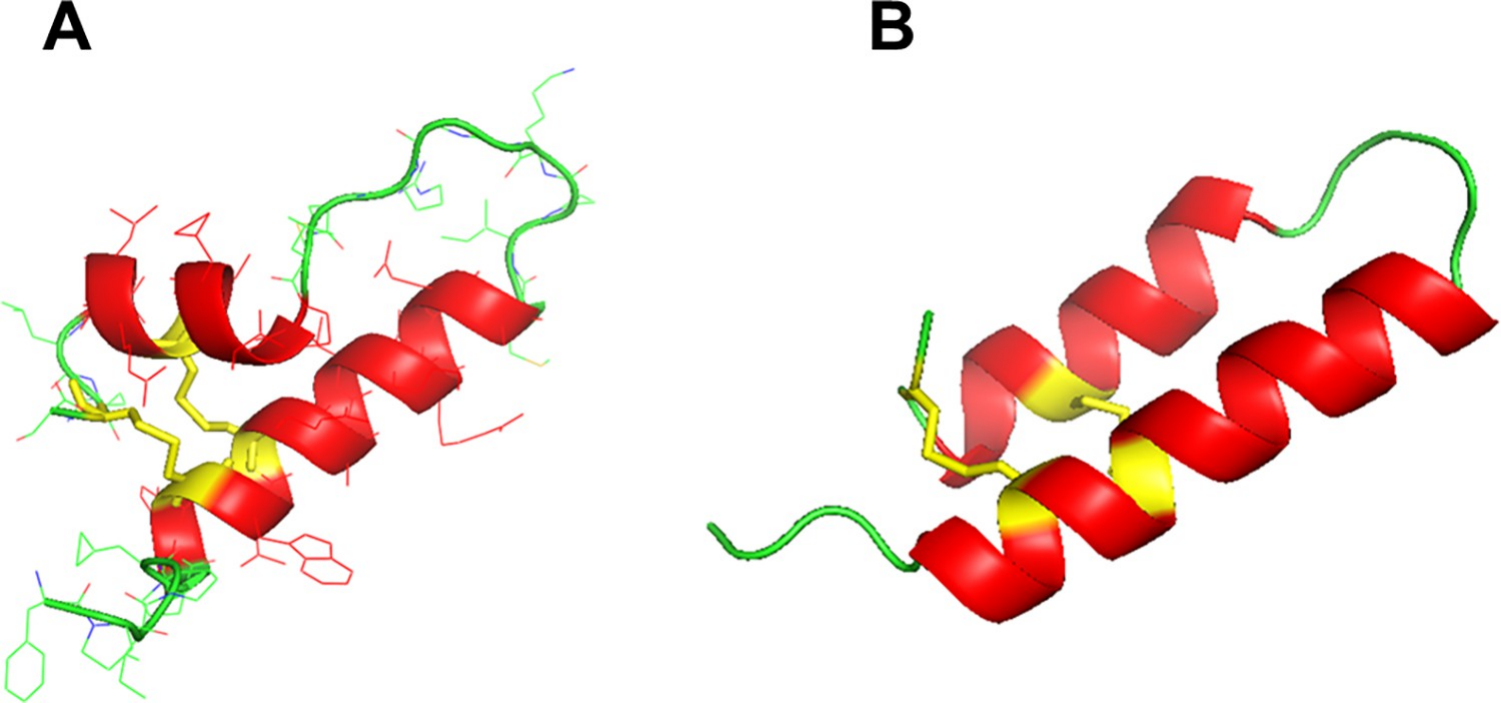
**(A)** Molecular illustration of 3D SMB based on initial homology templating predictions inserted into a bilayer of synthetic surfactant lipids and refined with molecular dynamics (S3 File). Disulfide connectivities were assigned with mass spectral analysis and helical domains determined with^13^C isotope enhanced backbone mapping. α-helical domains are highlighted in red ribbons, disulfide linkages and cysteines are shown in yellow, and bend and disordered backbone structures are shown as green tubes. **(B)** Molecular illustration of SMB 3D molecular dynamics refined structure in synthetic surfactant lipids (S4 File), based on initial AlphaFold predictions on the mass spectral determined disulfide connectivities and ^13^C isotope enhanced backbone mapping of helical domains. α-helical domains are highlighted in red ribbons, disulfide linkages and cysteines are shown in yellow, and bend and disordered backbone structures are shown as green tubes.

**Fig 8:**
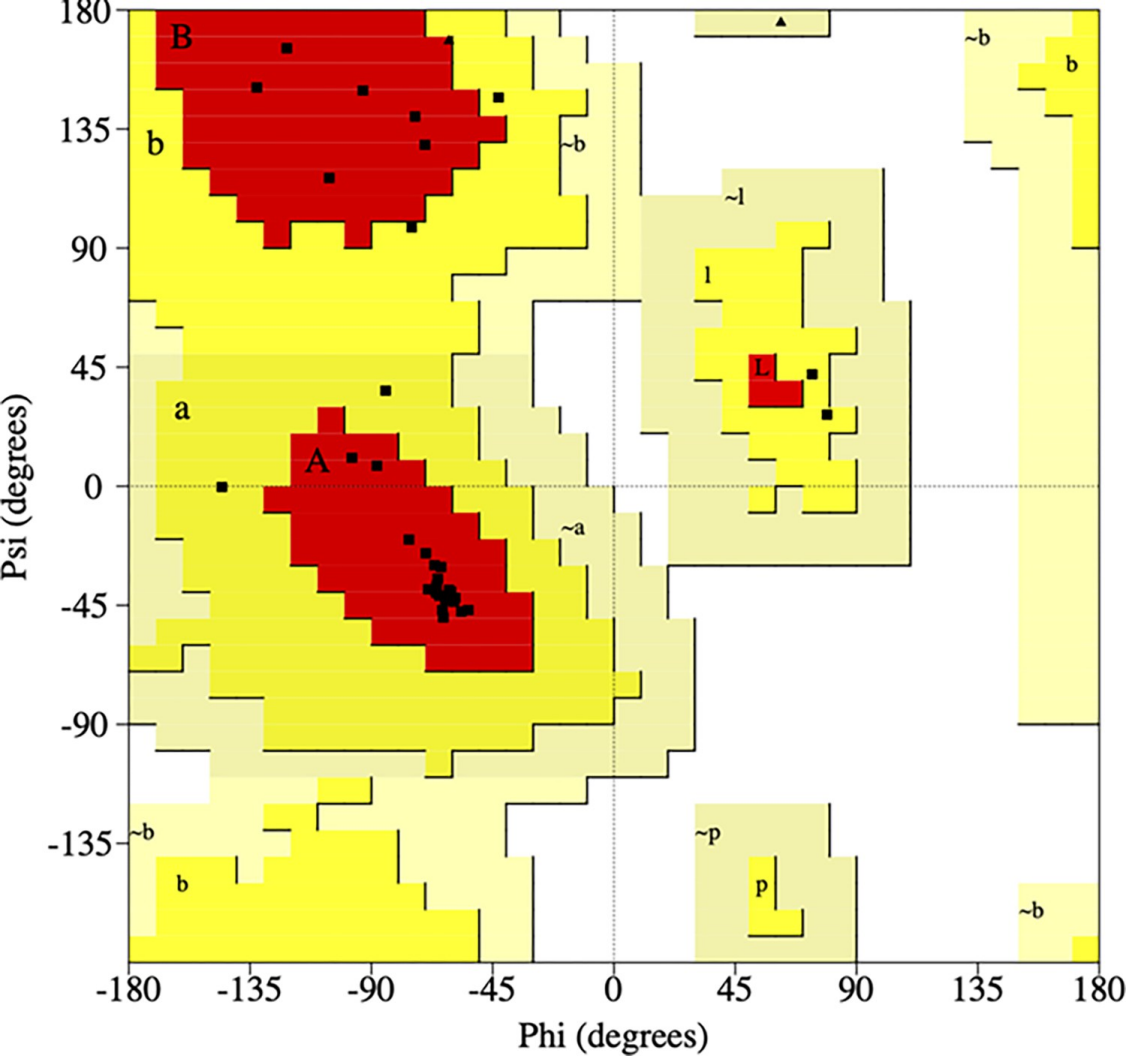
Ramachandran plot (http://www.ebi.ac.uk) PROsum server for the lowest energy structure of SMB derived from molecular dynamics refined peptide molecular coordinates of simulations in synthetic surfactant lipid bilayers. Red areas are those of most favored conformations (A, B, L, P, core conformations) while regions in brown (a, b, l, p) and dark yellow (a, b, l, p) represent additional allowed conformations. Black squares denote individual amino acid residues. Procheck metrics indicated that 90.6% of the amino acids are associated with Ramachandran plot core domains, while the remaining 9.4% fall in the yellow additional allowable plot areas.

Using the combination of the disulfide linkages and ^13^C measurement constraints followed by molecular dynamics refinement, a molecular model representing possible conformations of SMB in simulated surfactant lipid bilayers was generated. Fig 7A shows a ribbon-cartoon representation of the lowest energy conformer of the peptide in the surfactant multilayer environment. Geometry parameters of the final models were evaluated using Procheck [57, 61] to generate a Ramachandran plot (Fig 8) that confirms that the backbone torsion angles for the well-defined domains fall within the most favored regions of α-helix (core “A” labeled highlighted in red in Fig 8). High levels of backbone conformations associated with α-helical sequences include residues 6 to 21 in the N-terminal domain while amino acid residues 30 to 37 define the C-terminal domain (Fig 7A). Other sequences in the refined molecular model include the more disordered N-terminal insertion sequence (residues Phe-1 to Leu-5) and the bend region including residues 22 to 29 bridging the N-terminal–C-terminal helical domains to form the helix-hairpin structure.

Molecular dynamics refinement of the initial structure of SMB peptide predicted by AI in a simulated synthetic surfactant lipid bilayer is shown in Fig 7B. After 100 nano seconds of molecular dynamics there were several notable changes in the peptide structure. These included the orientation of the C-terminal helix from an almost parallel axial alinement with the N-terminal helix to a mean axis angle of about nine degrees. The C-terminal helix also had a bend in the axis rather than a straight axis that remained for the N-terminal helix during the time course of the simulation. However, the high amount of helix in both the N and C-terminal helix was unaltered from the initial AI predicted secondary structure that exceeds the total per cent helix observed experimentally in this membrane-like lipid bilayer system [33] suggesting that it is a less accurate predictor of the extent of the peptide helical domains.

**Fig 9:**
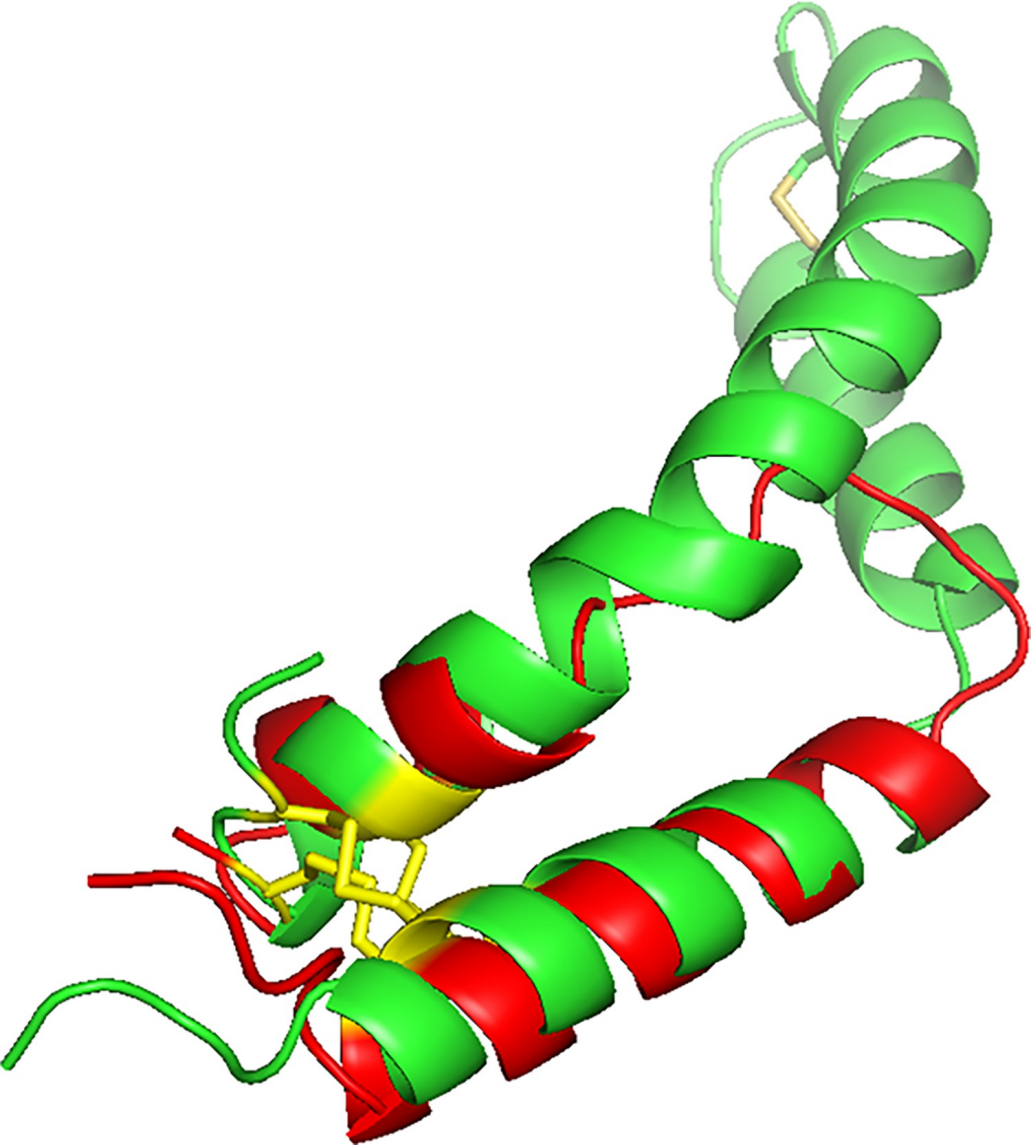
Comparison of predicted full length SP-B protein monomer with that of SMB peptide. The full-length human SP-B AlphaFold predicted structure (S5 File) is highlighted in green and is overlayed on the predicted SMB structure. The global RMSD value for the backbone atoms of SMB residues 1–25 and 26–41 compared with SP-B residues 1–25 and 63–78 is 4.36 Å (Superpose–webserver: http://superpose.wishartlab.com).

The optimal folding of SMB into a helix hairpin stabilized by disulfide pairing of the N-terminal and C-terminal domains is further supported by comparing the 3D structures by superimposing the SMB model on the homology templated model of human SP-B (Fig 4B). Both the N-terminal and C-terminal helical domains as well as the disulfide connectivity clearly overlap for the functional SMB fragment and the predicted monomeric SP-B full length protein indicating that the peptide construct closely emulates the 3D Saposin fold of the parent protein (Fig 9).

## Discussion

Although the Saposin family of proteins shares common disulfide connectivities as well as a conserved four-helical fold and performs functional roles by interacting with membrane lipids, the individual Saposins show a well-defined specificity for the class of lipids they interact with, resulting in unique lipid related structure-functions. Some of these functions include the formation of antimicrobial membrane pores in the case of NK-lysin and Granulysin, the extraction of lipids and transport of lipids for hydrolysis as seen with CS Activator (Saposin B), and with SP-B the stabilization of lipid films in the lung as a function of lateral compression and expansion of the lipid monolayer [8]. This well-defined specificity for target lipids appears in large part due to their unique amino acid sequences. This is emphasized by the observation that the structural alignments of Saposin B (PDB accession code: 4V2O) and SP-B (PDB accession code: 2IP3) have only a 21% identity (DALI–Protein Structure Comparison Server; http://ekhdua2.biocenter.helsinki.fi) and only 40% sequence similarity for all human Saposins [67]. Since all Saposins have amphipathic α-helices, the disposition of polar and non-polar amino acid residues in each helical segment impairs a protein polar footprint for interaction with a given target lipid class.

Another important function of the Saposin protein disulfide connectivity may be related to the lifetime of the protein in the biological milieu. Disulfide connectivity in Cerebroside Sulfate Activator (CSAct) is not necessary for biological activity or α-helical content but is required for trypsin resistance and strong ligand binding [64].

Krimm and Bandekar [68] were the first to suggest that ^13^C carbonyl labeling of select amino acid residues of the infrared Amide I C = O stretching conformational bands could be used to provide residue-specific information on the secondary structure of peptides and proteins. There are many examples of this approach to delineate well defined domains, so the entire backbone of local conformations for proteins can be mapped by selective isotope labeling of a peptide sequence. For example, the location of ß-sheet forming sequences in amyloid proteins has been determined by isotope enhanced FTIR [68, 69]. There are also many detailed reports on the identification of α-helical sequences in peptides [34, 70–72]. The medium level resolution of the isotope-enhanced FTIR structure for the Mini-B peptide [73] (PDB accession code: 1SSZ) compares well with the high-resolution solution NMR structure [46] (PDB accession code: 2DWF) suggesting that the vibrational spectroscopic approach can provide a reasonably accurate residue-specific approximation (S4 File) of the secondary structure of a peptide in similar membrane-like environments.

Additional information on the conformation of α-helical peptide domains can be directly obtained by isotope enhanced FTIR. The fraying of the ends of α-helical segments termini can be determined by isotope edited FTIR [43, 74, 75]. Isotope edited FTIR can also be employed to detect peptide backbone-solvent hydrogen bonding in helices by spectral shifts induced by water-backbone peptide carbonyls. This spectral shift can then be used to distinguish between helical segments that are bulk aqueous solvent exposed and those that are buried in the hydrophobic protein interior or in trans membrane environments [76]. In the present study using a ^13^C residue carbonyl cassette sequence scan of approximately six residues for the entire peptide may have also enhanced the resolution of the helical segments and permitted the characterization of helix transitional sequences that clearly identify helical residues that are undergoing disordered helix conformations, commonly referred to as helix fraying at the N and C termini of the helix domain.

One limitation of the isotope enhanced FTIR experimental approach is that it does not provide direct experimental distance determination of intra and inter molecular connectivity on a residue specific basis for peptides that have multiple disulfide linkages within and between multimeric protein ensembles. This limitation is emphasized by the differences in the N-terminal–C-terminal α-helical axis orientation predictions using the homology templating approach compared with the AI based methodology. The apparent predicted differences in helix orientation may simply represent the different structural databases used by the two predictive approaches for coordinate sampling, resulting in different disulfide distances and associated disulfide strain energies [63]. This can be seen in various structures among members of the Saposin family of proteins in the Protein Data Bank. Protein structures determined by X-ray crystallography typically have N-terminal–C-terminal helical axis orientations with a definitive angle and at least one of the two disulfide pairs having lower disulfide strain energies (PDB: 4DDJ, 1N69, 2GTG, 5U85). However, Saposin protein structures determined in solution or incorporated into detergent micelles experimentally by high-resolution NMR have N-terminal–C-terminal helical axis that have a more parallel orientation and high disulfide strain energies (PDB: 3NKL, 2DWF, 1SN6, 1OF9) (Table 1). These observations suggest that although the N-terminal–C-terminal disulfide connectivity of the Saposin proteins does constrain the helical pair, there is some flexibility in the structure that is dependent on the protein’s environment.

**Table 1:** Saposin protein N-terminal–C-terminal helical disulfide strain of members of the Saposin family deposited in the Protein Data Bank determined with X-ray crystallography and high-resolution NMR spectroscopy. Distance and disulfide strain energy were calculated using the disulfide bond dihedral angle energy server available through the IGP Institute for Genomics and Proteomics US Department of Energy Office of Science at UCLA (https://services.mbi.ucla.edu), based on the formalism of Katz et al. [62]. This web based utility uses the following equation: E(kJ/mol = 8.37(1+cos(3x1)) + 8.37(1+cos(3x1’)) = 4.18(1 + cos(3x2)) + 4.18(1+cos(3x2’)) + 14.64(1 +cos(2x3)) + 2.51(1+cos(3x3)) described in the original paper to provide the above energetic estimates. The server measures the dihedral angles and calculates a dihedral energy for all disulfide bonds found in the uploaded PDB coordinate file. Values for disulfide distance and strain energy are the mean and standard deviation (±) for the ten lowest energy conformers of the PDB coordinate set. No statistical estimates are available for PDB accession codes 4DDJ.PDB, 2GTG.PDB, and 5U85.PDB since the deposition has only one coordinate set for the protein.

Protein Name	PDB ID	Experimental Method	Residue Pair	S–S distance (Å)	Disulfide Strain Energy (kJ/mole)
NK-Lysin.	1NKL	NMR	Cys-4 Cys-76	2.034 ± 0.0055	18.006853 ± 2.964952
NK-Lysin.	1NKL	NMR	Cys-7 Cys-70	2.033 ± 0.0048	11.376348 ± 1.758260
Mini-B	2DWF	NMR	Cys-1 Cys-33	2.032 ± 0.0042	40.690171 ± 7.342447
Mini-B	2DWF	NMR	Cys-4 Cys-27	2.028 ± 0.0042	63.449626 ± 4.687185
Saposin C	1SN6	NMR	Cys-5 Cys-78	2.020 ± 0.0000	32.135855 ± 6.685295
Saposin C	1SN6	NMR	Cys-8 Cys-72	2.020 ± 0.0000	21.806422 ± 15.413616
Amoeba Pore	1OF9	NMR	Cys-5 Cys-77	2.057 ± 0.0581	29.956059 ± 9.231149
Amoeba Pore	1OF9	NMR	Cys-8 Cys-71	2.065 ± 0.7967	44.520839 ± 17.204087
Saposin A	4DDJ	X-ray	Cys-4 Cys-79	2.04	6.981088
Saposin A	4DDJ	X-ray	Cys-7 Cys-73	2.01	8.739460
Saposin B	1N69	X-ray	Cys-4 Cys-77	2.043 ± 0.0057	8.605333 ± 0.613185
Saposin B	1N69	X-ray	Cys-7 Cys-71	2.047 ± 0.0058	9.778188 ± 1.023867
Saposin C	2GTG	X-ray	Cys-5 Cys-78	2.04	6.383609
Saposin C	2GTG	X-ray	Cys-8 Cys-72	2.08	11.350614
Saposin D	5U85	X-ray	Cys-5 Cys-78	2.04	10.840251
Saposin D	5U85	X-ray	Cys-8 Cys-72	2.03	7.521138

Polarized FTIR measurements of the isotope enhanced helical domains of the SMB peptide in synthetic surfactant lipid multilayer films might have contributed to information on the helical axis orientation. However, this was not an option due to the lack of sensitivity of the polarization measurements to small differences in the helical orientation in the lipid film. Clearly high-resolution solid-state NMR studies of the peptide in surfactant films will be needed to better define the conformation of the peptide that would include detailed interaction of the N-terminal insertion sequence to form a homo-dimeric structure. Other important NMR measurements with regard to the molecular topography of the peptide in surfactant lipids would help reveal the depth of insertion of the peptide in the lipid and the influence of the peptide on the lipid environment as described by a recent detailed study of the insertion sequence (residues 1–7) per se [77]. These detailed studies should help provide better domain specific structure-activity correlations for the peptide in surfactant lipids.

In the present study of the amphipathic helix-hairpin peptide SMB, we boost the residue-specific isotope edited FTIR measurements with high resolution mass spectroscopy to determine residue specific intra molecular backbone connectivity. Finally, we refine the intermediate level structure using molecular dynamics in simulated lipid bilayers with constraints determined by the above experimental measurements to permit a reasonable approximation of the detailed structure of the peptide Saposin fold.

## Supporting information

S1 File (DOCX)

S2 File (DOCX)

S3 File (DOCX)

S4 File (DOCX)

S5 File (DOCX)
