# Supplementary material for: Emulation of the structure of the Saposin protein fold by a lung surfactant peptide construct of surfactant Protein B
Source: PLoS One. 2022 Nov 3;17(11):e0276787. doi: 10.1371/journal.pone.0276787 (PMC9632872; doi:10.1371/journal.pone.0276787)

**S1** **Supplement file** **- deposited in the ModelArchive** (<https://modelarchive.org/doi/10.5452/ma-axqi2>)

**Emulation of the Structure of the Saposin Protein Fold by a Lung Surfactant Peptide Construct of Surfactant Protein B**

Alan J. Waring^1,2^, Julian P. Whitelegge^3^, Shantanu K. Sharma^4^, Larry M. Gordon^1^,

Frans J. Walther^1,5,*^

^1^ Lundquist Institute for Biomedical Innovation at Harbor-UCLA Medical Center

1124 West Carson Street

Torrance, CA, USA

^2^ Department of Medicine

David Geffen School of Medicine

University of California Los Angeles

405 Hilgard Avenue

Los Angeles, CA, USA

^3^ NPI-Semel Institute for Neuroscience & Human Behavior Department of Psychiatry

& Biobehavioral Sciences, David Geffen School of Medicine at UCLA,

760 Westwood Plaza, Los Angeles, CA, USA

^4^ Materials and Process Simulation Center

California Institute of Technology

1200 East California Boulevard

Pasadena, CA, USA

^5^ Department of Pediatrics

David Geffen School of Medicine

University of California Los Angeles

405 Hilgard Avenue

Los Angeles, CA, USA

# ModelArchive (<https://modelarchive.org/doi/10.5452/ma-axqi2>) pdb

**Molecular coordinate set for for I-Tasser homology model with Cys disulfide atoms highlighted in yellow:**

ATOM 1 N PHE A 1 38.099 42.145 53.516 1.00 7.08

ATOM 2 CA PHE A 1 39.286 42.683 52.871 1.00 7.08

ATOM 3 HA PHE A 1 39.058 43.710 52.587 1.00 7.08

ATOM 4 CB PHE A 1 40.449 42.712 53.876 1.00 7.08

ATOM 5 HB1 PHE A 1 41.322 43.147 53.389 1.00 7.08

ATOM 6 HB2 PHE A 1 40.707 41.684 54.139 1.00 7.08

ATOM 7 CG PHE A 1 40.200 43.499 55.153 1.00 7.08

ATOM 8 CD1 PHE A 1 40.060 42.826 56.383 1.00 7.08

ATOM 9 HD1 PHE A 1 40.100 41.745 56.417 1.00 7.08

ATOM 10 CE1 PHE A 1 39.801 43.551 57.560 1.00 7.08

ATOM 11 HE1 PHE A 1 39.644 43.022 58.491 1.00 7.08

ATOM 12 CZ PHE A 1 39.679 44.951 57.513 1.00 7.08

ATOM 13 HZ PHE A 1 39.429 45.500 58.412 1.00 7.08

ATOM 14 CE2 PHE A 1 39.838 45.626 56.289 1.00 7.08

ATOM 15 HE2 PHE A 1 39.710 46.702 56.248 1.00 7.08

ATOM 16 CD2 PHE A 1 40.096 44.901 55.113 1.00 7.08

ATOM 17 HD2 PHE A 1 40.170 45.422 54.164 1.00 7.08

ATOM 18 C PHE A 1 39.773 41.980 51.583 1.00 7.08

ATOM 19 O PHE A 1 40.426 42.669 50.798 1.00 7.08

ATOM 20 N PRO A 2 39.515 40.680 51.298 1.00 5.89

ATOM 21 CD PRO A 2 38.772 39.690 52.074 1.00 5.89

ATOM 22 HD1 PRO A 2 37.768 40.034 52.317 1.00 5.89

ATOM 23 HD2 PRO A 2 39.321 39.450 52.985 1.00 5.89

ATOM 24 CG PRO A 2 38.650 38.447 51.194 1.00 5.89

ATOM 25 HG1 PRO A 2 37.731 38.506 50.608 1.00 5.89

ATOM 26 HG2 PRO A 2 38.667 37.532 51.787 1.00 5.89

ATOM 27 CB PRO A 2 39.855 38.547 50.266 1.00 5.89

ATOM 28 HB1 PRO A 2 39.688 38.024 49.324 1.00 5.89

ATOM 29 HB2 PRO A 2 40.734 38.144 50.770 1.00 5.89

ATOM 30 CA PRO A 2 40.030 40.056 50.073 1.00 5.89

ATOM 31 HA PRO A 2 41.095 40.273 49.969 1.00 5.89

ATOM 32 C PRO A 2 39.324 40.570 48.797 1.00 5.89

ATOM 33 O PRO A 2 39.761 40.290 47.684 1.00 5.89

ATOM 34 N ILE A 3 38.229 41.327 48.945 1.00 4.62

ATOM 35 H ILE A 3 37.957 41.551 49.889 1.00 4.62

ATOM 36 CA ILE A 3 37.402 41.867 47.858 1.00 4.62

ATOM 37 HA ILE A 3 37.081 41.022 47.252 1.00 4.62

ATOM 38 CB ILE A 3 36.134 42.539 48.446 1.00 4.62

ATOM 39 HB ILE A 3 36.443 43.410 49.027 1.00 4.62

ATOM 40 CG2 ILE A 3 35.205 43.024 47.314 1.00 4.62

ATOM 41 1HG2 ILE A 3 34.376 43.597 47.728 1.00 4.62

ATOM 42 2HG2 ILE A 3 35.732 43.678 46.623 1.00 4.62

ATOM 43 3HG2 ILE A 3 34.804 42.170 46.767 1.00 4.62

ATOM 44 CG1 ILE A 3 35.364 41.576 49.388 1.00 4.62

ATOM 45 1HG1 ILE A 3 36.023 41.250 50.190 1.00 4.62

ATOM 46 2HG1 ILE A 3 35.057 40.693 48.828 1.00 4.62

ATOM 47 CD1 ILE A 3 34.130 42.185 50.064 1.00 4.62

ATOM 48 HD1 ILE A 3 33.742 41.482 50.798 1.00 4.62

ATOM 49 HD2 ILE A 3 34.400 43.111 50.570 1.00 4.62

ATOM 50 HD3 ILE A 3 33.349 42.384 49.333 1.00 4.62

ATOM 51 C ILE A 3 38.213 42.871 46.990 1.00 4.62

ATOM 52 O ILE A 3 38.806 43.817 47.521 1.00 4.62

ATOM 53 N PRO A 4 38.237 42.733 45.648 1.00 3.89

ATOM 54 CD PRO A 4 37.531 41.699 44.896 1.00 3.89

ATOM 55 HD1 PRO A 4 36.451 41.797 44.995 1.00 3.89

ATOM 56 HD2 PRO A 4 37.856 40.715 45.238 1.00 3.89

ATOM 57 CG PRO A 4 37.943 41.875 43.437 1.00 3.89

ATOM 58 HG1 PRO A 4 37.312 42.624 42.956 1.00 3.89

ATOM 59 HG2 PRO A 4 37.917 40.931 42.892 1.00 3.89

ATOM 60 CB PRO A 4 39.363 42.407 43.587 1.00 3.89

ATOM 61 HB1 PRO A 4 39.716 42.880 42.670 1.00 3.89

ATOM 62 HB2 PRO A 4 40.029 41.585 43.855 1.00 3.89

ATOM 63 CA PRO A 4 39.240 43.371 44.780 1.00 3.89

ATOM 64 HA PRO A 4 40.196 43.371 45.302 1.00 3.89

ATOM 65 C PRO A 4 38.974 44.831 44.327 1.00 3.89

ATOM 66 O PRO A 4 39.485 45.245 43.294 1.00 3.89

ATOM 67 N LEU A 5 38.207 45.635 45.077 1.00 3.09

ATOM 68 H LEU A 5 37.852 45.240 45.937 1.00 3.09

ATOM 69 CA LEU A 5 37.731 46.985 44.688 1.00 3.09

ATOM 70 HA LEU A 5 36.891 46.803 44.025 1.00 3.09

ATOM 71 CB LEU A 5 37.183 47.710 45.933 1.00 3.09

ATOM 72 HB1 LEU A 5 36.846 48.698 45.621 1.00 3.09

ATOM 73 HB2 LEU A 5 38.000 47.846 46.643 1.00 3.09

ATOM 74 CG LEU A 5 36.010 47.021 46.660 1.00 3.09

ATOM 75 HG LEU A 5 36.336 46.044 47.016 1.00 3.09

ATOM 76 CD1 LEU A 5 35.605 47.861 47.868 1.00 3.09

ATOM 77 1HD1 LEU A 5 34.781 47.380 48.395 1.00 3.09

ATOM 78 2HD1 LEU A 5 36.444 47.947 48.556 1.00 3.09

ATOM 79 3HD1 LEU A 5 35.289 48.854 47.550 1.00 3.09

ATOM 80 CD2 LEU A 5 34.779 46.838 45.773 1.00 3.09

ATOM 81 1HD2 LEU A 5 33.959 46.423 46.359 1.00 3.09

ATOM 82 2HD2 LEU A 5 34.465 47.794 45.358 1.00 3.09

ATOM 83 3HD2 LEU A 5 34.990 46.146 44.961 1.00 3.09

ATOM 84 C LEU A 5 38.737 47.903 43.936 1.00 3.09

ATOM 85 O LEU A 5 39.864 48.071 44.416 1.00 3.09

ATOM 86 N PRO A 6 38.323 48.545 42.819 1.00 3.02

ATOM 87 CD PRO A 6 36.968 48.508 42.267 1.00 3.02

ATOM 88 HD1 PRO A 6 36.244 48.921 42.969 1.00 3.02

ATOM 89 HD2 PRO A 6 36.707 47.481 42.013 1.00 3.02

ATOM 90 CG PRO A 6 36.989 49.336 40.984 1.00 3.02

ATOM 91 HG1 PRO A 6 36.782 50.383 41.208 1.00 3.02

ATOM 92 HG2 PRO A 6 36.288 48.954 40.242 1.00 3.02

ATOM 93 CB PRO A 6 38.437 49.176 40.535 1.00 3.02

ATOM 94 HB1 PRO A 6 38.735 49.940 39.817 1.00 3.02

ATOM 95 HB2 PRO A 6 38.574 48.188 40.092 1.00 3.02

ATOM 96 CA PRO A 6 39.195 49.250 41.868 1.00 3.02

ATOM 97 HA PRO A 6 40.133 48.707 41.767 1.00 3.02

ATOM 98 C PRO A 6 39.542 50.697 42.276 1.00 3.02

ATOM 99 O PRO A 6 39.494 51.623 41.469 1.00 3.02

ATOM 100 N TYR A 7 39.841 50.923 43.556 1.00 2.45

ATOM 101 H TYR A 7 39.924 50.121 44.167 1.00 2.45

ATOM 102 CA TYR A 7 39.933 52.265 44.134 1.00 2.45

ATOM 103 HA TYR A 7 39.821 53.010 43.347 1.00 2.45

ATOM 104 CB TYR A 7 38.764 52.469 45.115 1.00 2.45

ATOM 105 HB1 TYR A 7 38.815 53.482 45.511 1.00 2.45

ATOM 106 HB2 TYR A 7 38.901 51.781 45.947 1.00 2.45

ATOM 107 CG TYR A 7 37.382 52.257 44.514 1.00 2.45

ATOM 108 CD1 TYR A 7 36.432 51.461 45.188 1.00 2.45

ATOM 109 HD1 TYR A 7 36.661 51.030 46.151 1.00 2.45

ATOM 110 CE1 TYR A 7 35.162 51.236 44.622 1.00 2.45

ATOM 111 HE1 TYR A 7 34.424 50.637 45.132 1.00 2.45

ATOM 112 CZ TYR A 7 34.827 51.822 43.384 1.00 2.45

ATOM 113 OH TYR A 7 33.598 51.613 42.847 1.00 2.45

ATOM 114 HH TYR A 7 33.479 52.085 42.021 1.00 2.45

ATOM 115 CE2 TYR A 7 35.767 52.631 42.715 1.00 2.45

ATOM 116 HE2 TYR A 7 35.517 53.082 41.767 1.00 2.45

ATOM 117 CD2 TYR A 7 37.038 52.845 43.280 1.00 2.45

ATOM 118 HD2 TYR A 7 37.752 53.456 42.747 1.00 2.45

ATOM 119 C TYR A 7 41.305 52.485 44.785 1.00 2.45

ATOM 120 O TYR A 7 41.662 51.891 45.808 1.00 2.45

ATOM 121 N CYS A 8 42.080 53.383 44.176 1.00 1.55

ATOM 122 H CYS A 8 41.740 53.792 43.318 1.00 1.55

ATOM 123 CA CYS A 8 43.473 53.648 44.532 1.00 1.55

ATOM 124 HA CYS A 8 44.057 52.755 44.304 1.00 1.55

ATOM 125 CB CYS A 8 43.974 54.789 43.635 1.00 1.55

ATOM 126 HB1 CYS A 8 43.540 55.730 43.973 1.00 1.55

ATOM 127 HB2 CYS A 8 43.636 54.616 42.611 1.00 1.55

ATOM 128 SG CYS A 8 45.771 54.961 43.603 1.00 1.55

ATOM 129 C CYS A 8 43.708 53.989 46.013 1.00 1.55

ATOM 130 O CYS A 8 44.715 53.583 46.591 1.00 1.55

ATOM 131 N TRP A 9 42.785 54.713 46.656 1.00 1.80

ATOM 132 H TRP A 9 41.990 55.057 46.141 1.00 1.80

ATOM 133 CA TRP A 9 42.913 55.056 48.079 1.00 1.80

ATOM 134 HA TRP A 9 43.930 55.392 48.283 1.00 1.80

ATOM 135 CB TRP A 9 41.975 56.212 48.427 1.00 1.80

ATOM 136 HB1 TRP A 9 41.866 56.234 49.513 1.00 1.80

ATOM 137 HB2 TRP A 9 40.985 56.006 48.016 1.00 1.80

ATOM 138 CG TRP A 9 42.401 57.590 48.006 1.00 1.80

ATOM 139 CD1 TRP A 9 41.726 58.704 48.360 1.00 1.80

ATOM 140 HD1 TRP A 9 40.813 58.716 48.946 1.00 1.80

ATOM 141 NE1 TRP A 9 42.370 59.822 47.880 1.00 1.80

ATOM 142 HE1 TRP A 9 42.018 60.764 48.001 1.00 1.80

ATOM 143 CE2 TRP A 9 43.516 59.491 47.197 1.00 1.80

ATOM 144 CZ2 TRP A 9 44.509 60.274 46.588 1.00 1.80

ATOM 145 HZ2 TRP A 9 44.425 61.348 46.584 1.00 1.80

ATOM 146 CH2 TRP A 9 45.579 59.647 45.936 1.00 1.80

ATOM 147 HH2 TRP A 9 46.320 60.240 45.415 1.00 1.80

ATOM 148 CZ3 TRP A 9 45.652 58.246 45.923 1.00 1.80

ATOM 149 HZ3 TRP A 9 46.455 57.754 45.386 1.00 1.80

ATOM 150 CE3 TRP A 9 44.672 57.471 46.572 1.00 1.80

ATOM 151 HE3 TRP A 9 44.777 56.404 46.544 1.00 1.80

ATOM 152 CD2 TRP A 9 43.562 58.064 47.227 1.00 1.80

ATOM 153 C TRP A 9 42.697 53.853 49.012 1.00 1.80

ATOM 154 O TRP A 9 43.404 53.717 50.013 1.00 1.80

ATOM 155 N LEU A 10 41.802 52.928 48.647 1.00 2.02

ATOM 156 H LEU A 10 41.315 53.037 47.771 1.00 2.02

ATOM 157 CA LEU A 10 41.678 51.648 49.351 1.00 2.02

ATOM 158 HA LEU A 10 41.580 51.841 50.421 1.00 2.02

ATOM 159 CB LEU A 10 40.440 50.863 48.884 1.00 2.02

ATOM 160 HB1 LEU A 10 40.381 49.957 49.488 1.00 2.02

ATOM 161 HB2 LEU A 10 40.584 50.561 47.846 1.00 2.02

ATOM 162 CG LEU A 10 39.098 51.609 48.993 1.00 2.02

ATOM 163 HG LEU A 10 39.074 52.410 48.254 1.00 2.02

ATOM 164 CD1 LEU A 10 37.972 50.625 48.682 1.00 2.02

ATOM 165 1HD1 LEU A 10 37.018 51.152 48.667 1.00 2.02

ATOM 166 2HD1 LEU A 10 38.142 50.163 47.712 1.00 2.02

ATOM 167 3HD1 LEU A 10 37.935 49.849 49.447 1.00 2.02

ATOM 168 CD2 LEU A 10 38.843 52.216 50.372 1.00 2.02

ATOM 169 1HD2 LEU A 10 37.845 52.652 50.409 1.00 2.02

ATOM 170 2HD2 LEU A 10 38.919 51.450 51.142 1.00 2.02

ATOM 171 3HD2 LEU A 10 39.562 53.006 50.575 1.00 2.02

ATOM 172 C LEU A 10 42.954 50.794 49.182 1.00 2.02

ATOM 173 O LEU A 10 43.415 50.115 50.112 1.00 2.02

ATOM 174 N CYS A 11 43.562 50.889 47.991 1.00 1.58

ATOM 175 H CYS A 11 43.110 51.398 47.242 1.00 1.58

ATOM 176 CA CYS A 11 44.886 50.316 47.785 1.00 1.58

ATOM 177 HA CYS A 11 44.812 49.264 48.066 1.00 1.58

ATOM 178 CB CYS A 11 45.249 50.319 46.301 1.00 1.58

ATOM 179 HB1 CYS A 11 45.413 51.337 45.955 1.00 1.58

ATOM 180 HB2 CYS A 11 44.419 49.889 45.740 1.00 1.58

ATOM 181 SG CYS A 11 46.725 49.337 45.957 1.00 1.58

ATOM 182 C CYS A 11 45.951 50.913 48.725 1.00 1.58

ATOM 183 O CYS A 11 46.649 50.144 49.387 1.00 1.58

ATOM 184 N ARG A 12 46.005 52.243 48.917 1.00 1.31

ATOM 185 H ARG A 12 45.446 52.843 48.319 1.00 1.31

ATOM 186 CA ARG A 12 46.832 52.837 49.988 1.00 1.31

ATOM 187 HA ARG A 12 47.857 52.523 49.786 1.00 1.31

ATOM 188 CB ARG A 12 46.835 54.376 49.959 1.00 1.31

ATOM 189 HB1 ARG A 12 45.824 54.756 50.111 1.00 1.31

ATOM 190 HB2 ARG A 12 47.190 54.702 48.979 1.00 1.31

ATOM 191 CG ARG A 12 47.769 54.936 51.054 1.00 1.31

ATOM 192 HG1 ARG A 12 48.683 54.342 51.087 1.00 1.31

ATOM 193 HG2 ARG A 12 47.275 54.852 52.023 1.00 1.31

ATOM 194 CD ARG A 12 48.186 56.398 50.846 1.00 1.31

ATOM 195 HD1 ARG A 12 48.830 56.447 49.968 1.00 1.31

ATOM 196 HD2 ARG A 12 48.783 56.712 51.703 1.00 1.31

ATOM 197 NE ARG A 12 47.024 57.297 50.708 1.00 1.31

ATOM 198 HE ARG A 12 46.423 57.394 51.511 1.00 1.31

ATOM 199 CZ ARG A 12 46.661 57.939 49.614 1.00 1.31

ATOM 200 NH1 ARG A 12 45.533 58.584 49.588 1.00 1.31

ATOM 201 1HH1 ARG A 12 44.934 58.616 50.392 1.00 1.31

ATOM 202 2HH1 ARG A 12 45.245 59.025 48.731 1.00 1.31

ATOM 203 NH2 ARG A 12 47.378 57.961 48.527 1.00 1.31

ATOM 204 1HH2 ARG A 12 48.307 57.547 48.519 1.00 1.31

ATOM 205 2HH2 ARG A 12 47.012 58.397 47.697 1.00 1.31

ATOM 206 C ARG A 12 46.527 52.247 51.363 1.00 1.31

ATOM 207 O ARG A 12 47.472 52.014 52.120 1.00 1.31

ATOM 208 N ALA A 13 45.262 51.941 51.664 1.00 1.77

ATOM 209 H ALA A 13 44.527 52.171 51.007 1.00 1.77

ATOM 210 CA ALA A 13 44.911 51.324 52.939 1.00 1.77

ATOM 211 HA ALA A 13 45.295 51.991 53.713 1.00 1.77

ATOM 212 CB ALA A 13 43.389 51.293 53.122 1.00 1.77

ATOM 213 HB1 ALA A 13 43.154 51.054 54.160 1.00 1.77

ATOM 214 HB2 ALA A 13 42.964 52.270 52.887 1.00 1.77

ATOM 215 HB3 ALA A 13 42.931 50.534 52.492 1.00 1.77

ATOM 216 C ALA A 13 45.586 49.966 53.217 1.00 1.77

ATOM 217 O ALA A 13 46.365 49.816 54.175 1.00 1.77

ATOM 218 N LEU A 14 45.313 48.972 52.362 1.00 1.75

ATOM 219 H LEU A 14 44.711 49.149 51.563 1.00 1.75

ATOM 220 CA LEU A 14 45.889 47.630 52.598 1.00 1.75

ATOM 221 HA LEU A 14 45.723 47.348 53.636 1.00 1.75

ATOM 222 CB LEU A 14 45.196 46.611 51.677 1.00 1.75

ATOM 223 HB1 LEU A 14 45.736 45.664 51.728 1.00 1.75

ATOM 224 HB2 LEU A 14 45.255 46.973 50.649 1.00 1.75

ATOM 225 CG LEU A 14 43.719 46.353 52.033 1.00 1.75

ATOM 226 HG LEU A 14 43.171 47.295 52.049 1.00 1.75

ATOM 227 CD1 LEU A 14 43.081 45.448 50.979 1.00 1.75

ATOM 228 1HD1 LEU A 14 42.029 45.287 51.214 1.00 1.75

ATOM 229 2HD1 LEU A 14 43.150 45.923 50.002 1.00 1.75

ATOM 230 3HD1 LEU A 14 43.586 44.483 50.949 1.00 1.75

ATOM 231 CD2 LEU A 14 43.571 45.671 53.399 1.00 1.75

ATOM 232 1HD2 LEU A 14 44.175 44.767 53.419 1.00 1.75

ATOM 233 2HD2 LEU A 14 43.882 46.348 54.191 1.00 1.75

ATOM 234 3HD2 LEU A 14 42.526 45.415 53.559 1.00 1.75

ATOM 235 C LEU A 14 47.400 47.621 52.387 1.00 1.75

ATOM 236 O LEU A 14 48.077 46.913 53.125 1.00 1.75

ATOM 237 N ILE A 15 47.946 48.420 51.463 1.00 1.33

ATOM 238 H ILE A 15 47.350 48.980 50.861 1.00 1.33

ATOM 239 CA ILE A 15 49.405 48.507 51.318 1.00 1.33

ATOM 240 HA ILE A 15 49.776 47.486 51.239 1.00 1.33

ATOM 241 CB ILE A 15 49.836 49.233 50.020 1.00 1.33

ATOM 242 HB ILE A 15 49.374 50.223 50.009 1.00 1.33

ATOM 243 CG2 ILE A 15 51.366 49.429 49.977 1.00 1.33

ATOM 244 1HG2 ILE A 15 51.668 49.816 49.005 1.00 1.33

ATOM 245 2HG2 ILE A 15 51.678 50.139 50.743 1.00 1.33

ATOM 246 3HG2 ILE A 15 51.875 48.482 50.152 1.00 1.33

ATOM 247 CG1 ILE A 15 49.392 48.476 48.743 1.00 1.33

ATOM 248 1HG1 ILE A 15 49.746 49.030 47.872 1.00 1.33

ATOM 249 2HG1 ILE A 15 48.306 48.458 48.690 1.00 1.33

ATOM 250 CD1 ILE A 15 49.866 47.021 48.605 1.00 1.33

ATOM 251 HD1 ILE A 15 49.585 46.647 47.621 1.00 1.33

ATOM 252 HD2 ILE A 15 50.947 46.952 48.710 1.00 1.33

ATOM 253 HD3 ILE A 15 49.391 46.395 49.358 1.00 1.33

ATOM 254 C ILE A 15 50.076 49.059 52.584 1.00 1.33

ATOM 255 O ILE A 15 51.061 48.472 53.049 1.00 1.33

ATOM 256 N LYS A 16 49.532 50.111 53.222 1.00 1.35

ATOM 257 H LYS A 16 48.715 50.564 52.820 1.00 1.35

ATOM 258 CA LYS A 16 50.066 50.609 54.505 1.00 1.35

ATOM 259 HA LYS A 16 51.117 50.842 54.341 1.00 1.35

ATOM 260 CB LYS A 16 49.325 51.893 54.919 1.00 1.35

ATOM 261 HB1 LYS A 16 48.280 51.653 55.118 1.00 1.35

ATOM 262 HB2 LYS A 16 49.345 52.604 54.092 1.00 1.35

ATOM 263 CG LYS A 16 49.920 52.568 56.168 1.00 1.35

ATOM 264 HG1 LYS A 16 49.974 51.854 56.991 1.00 1.35

ATOM 265 HG2 LYS A 16 49.236 53.360 56.479 1.00 1.35

ATOM 266 CD LYS A 16 51.305 53.210 55.930 1.00 1.35

ATOM 267 HD1 LYS A 16 51.188 54.288 56.055 1.00 1.35

ATOM 268 HD2 LYS A 16 51.634 53.045 54.902 1.00 1.35

ATOM 269 CE LYS A 16 52.388 52.734 56.910 1.00 1.35

ATOM 270 HE1 LYS A 16 51.981 52.745 57.925 1.00 1.35

ATOM 271 HE2 LYS A 16 53.211 53.453 56.875 1.00 1.35

ATOM 272 NZ LYS A 16 52.906 51.389 56.566 1.00 1.35

ATOM 273 HZ1 LYS A 16 52.192 50.669 56.669 1.00 1.35

ATOM 274 HZ2 LYS A 16 53.214 51.366 55.593 1.00 1.35

ATOM 275 HZ3 LYS A 16 53.691 51.130 57.149 1.00 1.35

ATOM 276 C LYS A 16 50.004 49.536 55.611 1.00 1.35

ATOM 277 O LYS A 16 50.989 49.331 56.349 1.00 1.35

ATOM 278 N ARG A 17 48.865 48.833 55.716 1.00 1.76

ATOM 279 H ARG A 17 48.097 49.065 55.090 1.00 1.76

ATOM 280 CA ARG A 17 48.708 47.760 56.718 1.00 1.76

ATOM 281 HA ARG A 17 48.918 48.187 57.702 1.00 1.76

ATOM 282 CB ARG A 17 47.253 47.252 56.718 1.00 1.76

ATOM 283 HB1 ARG A 17 47.064 46.665 55.818 1.00 1.76

ATOM 284 HB2 ARG A 17 46.582 48.112 56.721 1.00 1.76

ATOM 285 CG ARG A 17 46.973 46.406 57.972 1.00 1.76

ATOM 286 HG1 ARG A 17 47.249 46.989 58.852 1.00 1.76

ATOM 287 HG2 ARG A 17 47.588 45.507 57.963 1.00 1.76

ATOM 288 CD ARG A 17 45.498 46.015 58.114 1.00 1.76

ATOM 289 HD1 ARG A 17 44.884 46.912 58.006 1.00 1.76

ATOM 290 HD2 ARG A 17 45.348 45.613 59.118 1.00 1.76

ATOM 291 NE ARG A 17 45.078 44.992 57.135 1.00 1.76

ATOM 292 HE ARG A 17 45.810 44.535 56.615 1.00 1.76

ATOM 293 CZ ARG A 17 43.835 44.580 56.930 1.00 1.76

ATOM 294 NH1 ARG A 17 43.563 43.643 56.070 1.00 1.76

ATOM 295 1HH1 ARG A 17 44.293 43.262 55.495 1.00 1.76

ATOM 296 2HH1 ARG A 17 42.603 43.377 55.907 1.00 1.76

ATOM 297 NH2 ARG A 17 42.819 45.091 57.564 1.00 1.76

ATOM 298 1HH2 ARG A 17 42.969 45.833 58.220 1.00 1.76

ATOM 299 2HH2 ARG A 17 41.878 44.787 57.352 1.00 1.76

ATOM 300 C ARG A 17 49.719 46.619 56.526 1.00 1.76

ATOM 301 O ARG A 17 50.383 46.219 57.481 1.00 1.76

ATOM 302 N ILE A 18 49.896 46.148 55.298 1.00 1.63

ATOM 303 H ILE A 18 49.324 46.539 54.557 1.00 1.63

ATOM 304 CA ILE A 18 50.821 45.065 54.936 1.00 1.63

ATOM 305 HA ILE A 18 50.625 44.221 55.592 1.00 1.63

ATOM 306 CB ILE A 18 50.530 44.593 53.487 1.00 1.63

ATOM 307 HB ILE A 18 50.532 45.470 52.836 1.00 1.63

ATOM 308 CG2 ILE A 18 51.596 43.607 52.976 1.00 1.63

ATOM 309 1HG2 ILE A 18 51.365 43.286 51.962 1.00 1.63

ATOM 310 2HG2 ILE A 18 52.577 44.081 52.950 1.00 1.63

ATOM 311 3HG2 ILE A 18 51.638 42.735 53.629 1.00 1.63

ATOM 312 CG1 ILE A 18 49.143 43.907 53.428 1.00 1.63

ATOM 313 1HG1 ILE A 18 48.407 44.491 53.978 1.00 1.63

ATOM 314 2HG1 ILE A 18 49.195 42.932 53.913 1.00 1.63

ATOM 315 CD1 ILE A 18 48.592 43.723 52.005 1.00 1.63

ATOM 316 HD1 ILE A 18 47.576 43.333 52.061 1.00 1.63

ATOM 317 HD2 ILE A 18 48.573 44.682 51.487 1.00 1.63

ATOM 318 HD3 ILE A 18 49.205 43.020 51.445 1.00 1.63

ATOM 319 C ILE A 18 52.295 45.460 55.166 1.00 1.63

ATOM 320 O ILE A 18 53.095 44.654 55.640 1.00 1.63

ATOM 321 N GLN A 19 52.642 46.733 54.936 1.00 1.17

ATOM 322 H GLN A 19 51.954 47.345 54.511 1.00 1.17

ATOM 323 CA GLN A 19 53.939 47.297 55.336 1.00 1.17

ATOM 324 HA GLN A 19 54.744 46.709 54.891 1.00 1.17

ATOM 325 CB GLN A 19 54.038 48.748 54.843 1.00 1.17

ATOM 326 HB1 GLN A 19 54.854 49.249 55.366 1.00 1.17

ATOM 327 HB2 GLN A 19 53.112 49.237 55.121 1.00 1.17

ATOM 328 CG GLN A 19 54.256 48.995 53.340 1.00 1.17

ATOM 329 HG1 GLN A 19 53.564 48.392 52.755 1.00 1.17

ATOM 330 HG2 GLN A 19 55.269 48.690 53.077 1.00 1.17

ATOM 331 CD GLN A 19 54.062 50.476 52.983 1.00 1.17

ATOM 332 OE1 GLN A 19 53.548 51.278 53.760 1.00 1.17

ATOM 333 NE2 GLN A 19 54.485 50.900 51.811 1.00 1.17

ATOM 334 1HE2 GLN A 19 54.941 50.288 51.151 1.00 1.17

ATOM 335 2HE2 GLN A 19 54.377 51.874 51.603 1.00 1.17

ATOM 336 C GLN A 19 54.160 47.305 56.854 1.00 1.17

ATOM 337 O GLN A 19 55.317 47.260 57.265 1.00 1.17

ATOM 338 N ALA A 20 53.116 47.378 57.696 1.00 1.53

ATOM 339 H ALA A 20 52.172 47.415 57.334 1.00 1.53

ATOM 340 CA ALA A 20 53.329 47.027 59.117 1.00 1.53

ATOM 341 HA ALA A 20 54.251 47.494 59.470 1.00 1.53

ATOM 342 CB ALA A 20 52.179 47.605 59.952 1.00 1.53

ATOM 343 HB1 ALA A 20 52.378 47.427 61.010 1.00 1.53

ATOM 344 HB2 ALA A 20 52.104 48.679 59.783 1.00 1.53

ATOM 345 HB3 ALA A 20 51.236 47.127 59.691 1.00 1.53

ATOM 346 C ALA A 20 53.493 45.508 59.349 1.00 1.53

ATOM 347 O ALA A 20 54.336 45.094 60.139 1.00 1.53

ATOM 348 N MET A 21 52.705 44.681 58.651 1.00 1.84

ATOM 349 H MET A 21 52.021 45.096 58.031 1.00 1.84

ATOM 350 CA MET A 21 52.670 43.220 58.836 1.00 1.84

ATOM 351 HA MET A 21 52.491 43.017 59.893 1.00 1.84

ATOM 352 CB MET A 21 51.501 42.627 58.029 1.00 1.84

ATOM 353 HB1 MET A 21 51.551 41.538 58.085 1.00 1.84

ATOM 354 HB2 MET A 21 51.616 42.910 56.986 1.00 1.84

ATOM 355 CG MET A 21 50.116 43.067 58.531 1.00 1.84

ATOM 356 HG1 MET A 21 50.155 44.116 58.820 1.00 1.84

ATOM 357 HG2 MET A 21 49.881 42.493 59.429 1.00 1.84

ATOM 358 SD MET A 21 48.749 42.878 57.340 1.00 1.84

ATOM 359 CE MET A 21 48.887 41.110 56.958 1.00 1.84

ATOM 360 HE1 MET A 21 49.830 40.914 56.447 1.00 1.84

ATOM 361 HE2 MET A 21 48.849 40.529 57.881 1.00 1.84

ATOM 362 HE3 MET A 21 48.067 40.812 56.306 1.00 1.84

ATOM 363 C MET A 21 53.963 42.466 58.468 1.00 1.84

ATOM 364 O MET A 21 54.208 41.412 59.054 1.00 1.84

ATOM 365 N ILE A 22 54.782 42.944 57.515 1.00 1.73

ATOM 366 H ILE A 22 54.491 43.785 57.031 1.00 1.73

ATOM 367 CA ILE A 22 55.886 42.135 56.938 1.00 1.73

ATOM 368 HA ILE A 22 56.031 41.266 57.579 1.00 1.73

ATOM 369 CB ILE A 22 55.498 41.562 55.543 1.00 1.73

ATOM 370 HB ILE A 22 55.599 42.356 54.802 1.00 1.73

ATOM 371 CG2 ILE A 22 56.463 40.413 55.173 1.00 1.73

ATOM 372 1HG2 ILE A 22 56.316 40.117 54.135 1.00 1.73

ATOM 373 2HG2 ILE A 22 57.502 40.719 55.275 1.00 1.73

ATOM 374 3HG2 ILE A 22 56.283 39.551 55.817 1.00 1.73

ATOM 375 CG1 ILE A 22 54.032 41.057 55.475 1.00 1.73

ATOM 376 1HG1 ILE A 22 53.363 41.897 55.653 1.00 1.73

ATOM 377 2HG1 ILE A 22 53.867 40.316 56.259 1.00 1.73

ATOM 378 CD1 ILE A 22 53.608 40.451 54.130 1.00 1.73

ATOM 379 HD1 ILE A 22 52.534 40.266 54.146 1.00 1.73

ATOM 380 HD2 ILE A 22 53.840 41.145 53.322 1.00 1.73

ATOM 381 HD3 ILE A 22 54.117 39.503 53.960 1.00 1.73

ATOM 382 C ILE A 22 57.260 42.872 56.928 1.00 1.73

ATOM 383 O ILE A 22 57.826 43.163 55.864 1.00 1.73

ATOM 384 N PRO A 23 57.860 43.165 58.102 1.00 1.75

ATOM 385 CD PRO A 23 57.297 42.880 59.420 1.00 1.75

ATOM 386 HD1 PRO A 23 57.051 41.824 59.536 1.00 1.75

ATOM 387 HD2 PRO A 23 56.407 43.491 59.575 1.00 1.75

ATOM 388 CG PRO A 23 58.356 43.289 60.439 1.00 1.75

ATOM 389 HG1 PRO A 23 59.041 42.460 60.615 1.00 1.75

ATOM 390 HG2 PRO A 23 57.908 43.628 61.374 1.00 1.75

ATOM 391 CB PRO A 23 59.080 44.420 59.714 1.00 1.75

ATOM 392 HB1 PRO A 23 60.068 44.611 60.130 1.00 1.75

ATOM 393 HB2 PRO A 23 58.474 45.326 59.772 1.00 1.75

ATOM 394 CA PRO A 23 59.110 43.932 58.255 1.00 1.75

ATOM 395 HA PRO A 23 59.054 44.810 57.611 1.00 1.75

ATOM 396 C PRO A 23 60.408 43.177 57.876 1.00 1.75

ATOM 397 O PRO A 23 61.334 43.022 58.680 1.00 1.75

ATOM 398 N LYS A 24 60.478 42.674 56.640 1.00 2.45

ATOM 399 H LYS A 24 59.615 42.746 56.108 1.00 2.45

ATOM 400 CA LYS A 24 61.689 42.158 55.949 1.00 2.45

ATOM 401 HA LYS A 24 62.490 42.886 56.082 1.00 2.45

ATOM 402 CB LYS A 24 62.106 40.810 56.583 1.00 2.45

ATOM 403 HB1 LYS A 24 62.124 40.011 55.841 1.00 2.45

ATOM 404 HB2 LYS A 24 61.378 40.520 57.341 1.00 2.45

ATOM 405 CG LYS A 24 63.505 40.921 57.221 1.00 2.45

ATOM 406 HG1 LYS A 24 63.621 41.899 57.691 1.00 2.45

ATOM 407 HG2 LYS A 24 64.249 40.854 56.425 1.00 2.45

ATOM 408 CD LYS A 24 63.794 39.837 58.271 1.00 2.45

ATOM 409 HD1 LYS A 24 64.829 39.945 58.598 1.00 2.45

ATOM 410 HD2 LYS A 24 63.697 38.858 57.799 1.00 2.45

ATOM 411 CE LYS A 24 62.875 39.887 59.505 1.00 2.45

ATOM 412 HE1 LYS A 24 63.216 39.130 60.217 1.00 2.45

ATOM 413 HE2 LYS A 24 61.861 39.616 59.198 1.00 2.45

ATOM 414 NZ LYS A 24 62.859 41.225 60.154 1.00 2.45

ATOM 415 HZ1 LYS A 24 62.310 41.212 61.002 1.00 2.45

ATOM 416 HZ2 LYS A 24 63.793 41.537 60.376 1.00 2.45

ATOM 417 HZ3 LYS A 24 62.434 41.912 59.528 1.00 2.45

ATOM 418 C LYS A 24 61.506 42.050 54.422 1.00 2.45

ATOM 419 O LYS A 24 62.338 41.462 53.753 1.00 2.45

ATOM 420 N GLY A 25 60.421 42.619 53.872 1.00 2.99

ATOM 421 H GLY A 25 59.786 43.108 54.481 1.00 2.99

ATOM 422 CA GLY A 25 59.959 42.376 52.498 1.00 2.99

ATOM 423 HA1 GLY A 25 59.129 41.671 52.531 1.00 2.99

ATOM 424 HA2 GLY A 25 60.759 41.919 51.916 1.00 2.99

ATOM 425 C GLY A 25 59.498 43.623 51.742 1.00 2.99

ATOM 426 O GLY A 25 58.428 43.609 51.134 1.00 2.99

ATOM 427 N GLY A 26 60.281 44.705 51.813 1.00 2.34

ATOM 428 H GLY A 26 61.166 44.595 52.286 1.00 2.34

ATOM 429 CA GLY A 26 59.968 46.043 51.287 1.00 2.34

ATOM 430 HA1 GLY A 26 60.902 46.576 51.108 1.00 2.34

ATOM 431 HA2 GLY A 26 59.427 46.590 52.059 1.00 2.34

ATOM 432 C GLY A 26 59.159 46.090 49.994 1.00 2.34

ATOM 433 O GLY A 26 59.492 45.445 48.992 1.00 2.34

ATOM 434 N ARG A 27 58.104 46.899 50.014 1.00 2.24

ATOM 435 H ARG A 27 57.970 47.409 50.878 1.00 2.24

ATOM 436 CA ARG A 27 57.343 47.403 48.863 1.00 2.24

ATOM 437 HA ARG A 27 57.695 46.948 47.936 1.00 2.24

ATOM 438 CB ARG A 27 55.834 47.138 49.075 1.00 2.24

ATOM 439 HB1 ARG A 27 55.295 48.062 48.866 1.00 2.24

ATOM 440 HB2 ARG A 27 55.632 46.903 50.122 1.00 2.24

ATOM 441 CG ARG A 27 55.221 46.043 48.182 1.00 2.24

ATOM 442 HG1 ARG A 27 55.506 46.216 47.143 1.00 2.24

ATOM 443 HG2 ARG A 27 54.137 46.146 48.248 1.00 2.24

ATOM 444 CD ARG A 27 55.568 44.604 48.585 1.00 2.24

ATOM 445 HD1 ARG A 27 54.903 43.933 48.043 1.00 2.24

ATOM 446 HD2 ARG A 27 55.374 44.475 49.652 1.00 2.24

ATOM 447 NE ARG A 27 56.977 44.269 48.309 1.00 2.24

ATOM 448 HE ARG A 27 57.676 44.826 48.782 1.00 2.24

ATOM 449 CZ ARG A 27 57.479 43.250 47.644 1.00 2.24

ATOM 450 NH1 ARG A 27 58.769 43.128 47.542 1.00 2.24

ATOM 451 1HH1 ARG A 27 59.334 43.838 48.004 1.00 2.24

ATOM 452 2HH1 ARG A 27 59.198 42.332 47.114 1.00 2.24

ATOM 453 NH2 ARG A 27 56.728 42.350 47.075 1.00 2.24

ATOM 454 1HH2 ARG A 27 55.732 42.430 47.166 1.00 2.24

ATOM 455 2HH2 ARG A 27 57.138 41.602 46.554 1.00 2.24

ATOM 456 C ARG A 27 57.628 48.897 48.791 1.00 2.24

ATOM 457 O ARG A 27 57.811 49.532 49.828 1.00 2.24

ATOM 458 N MET A 28 57.669 49.422 47.572 1.00 1.95

ATOM 459 H MET A 28 57.519 48.815 46.788 1.00 1.95

ATOM 460 CA MET A 28 57.899 50.839 47.301 1.00 1.95

ATOM 461 HA MET A 28 58.826 51.142 47.791 1.00 1.95

ATOM 462 CB MET A 28 58.047 51.060 45.781 1.00 1.95

ATOM 463 HB1 MET A 28 58.399 52.075 45.604 1.00 1.95

ATOM 464 HB2 MET A 28 57.074 50.947 45.301 1.00 1.95

ATOM 465 CG MET A 28 59.031 50.095 45.099 1.00 1.95

ATOM 466 HG1 MET A 28 59.181 50.421 44.069 1.00 1.95

ATOM 467 HG2 MET A 28 58.572 49.108 45.061 1.00 1.95

ATOM 468 SD MET A 28 60.649 49.928 45.900 1.00 1.95

ATOM 469 CE MET A 28 61.410 51.516 45.470 1.00 1.95

ATOM 470 HE1 MET A 28 62.428 51.546 45.858 1.00 1.95

ATOM 471 HE2 MET A 28 61.441 51.627 44.387 1.00 1.95

ATOM 472 HE3 MET A 28 60.840 52.333 45.907 1.00 1.95

ATOM 473 C MET A 28 56.770 51.719 47.874 1.00 1.95

ATOM 474 O MET A 28 55.828 51.239 48.521 1.00 1.95

ATOM 475 N LEU A 29 56.828 53.022 47.595 1.00 2.48

ATOM 476 H LEU A 29 57.608 53.361 47.057 1.00 2.48

ATOM 477 CA LEU A 29 55.753 53.949 47.932 1.00 2.48

ATOM 478 HA LEU A 29 55.697 53.988 49.017 1.00 2.48

ATOM 479 CB LEU A 29 56.082 55.354 47.392 1.00 2.48

ATOM 480 HB1 LEU A 29 55.228 56.005 47.579 1.00 2.48

ATOM 481 HB2 LEU A 29 56.217 55.287 46.312 1.00 2.48

ATOM 482 CG LEU A 29 57.332 56.013 48.007 1.00 2.48

ATOM 483 HG LEU A 29 58.203 55.389 47.811 1.00 2.48

ATOM 484 CD1 LEU A 29 57.551 57.376 47.356 1.00 2.48

ATOM 485 1HD1 LEU A 29 58.459 57.829 47.753 1.00 2.48

ATOM 486 2HD1 LEU A 29 57.673 57.261 46.279 1.00 2.48

ATOM 487 3HD1 LEU A 29 56.709 58.037 47.556 1.00 2.48

ATOM 488 CD2 LEU A 29 57.199 56.221 49.518 1.00 2.48

ATOM 489 1HD2 LEU A 29 58.073 56.757 49.889 1.00 2.48

ATOM 490 2HD2 LEU A 29 56.308 56.805 49.746 1.00 2.48

ATOM 491 3HD2 LEU A 29 57.159 55.264 50.032 1.00 2.48

ATOM 492 C LEU A 29 54.396 53.465 47.400 1.00 2.48

ATOM 493 O LEU A 29 54.308 53.006 46.257 1.00 2.48

ATOM 494 N PRO A 30 53.317 53.594 48.192 1.00 1.84

ATOM 495 CD PRO A 30 53.218 54.298 49.461 1.00 1.84

ATOM 496 HD1 PRO A 30 53.646 55.298 49.401 1.00 1.84

ATOM 497 HD2 PRO A 30 53.718 53.718 50.238 1.00 1.84

ATOM 498 CG PRO A 30 51.724 54.381 49.764 1.00 1.84

ATOM 499 HG1 PRO A 30 51.298 55.253 49.271 1.00 1.84

ATOM 500 HG2 PRO A 30 51.529 54.409 50.836 1.00 1.84

ATOM 501 CB PRO A 30 51.175 53.110 49.119 1.00 1.84

ATOM 502 HB1 PRO A 30 50.127 53.218 48.839 1.00 1.84

ATOM 503 HB2 PRO A 30 51.289 52.285 49.819 1.00 1.84

ATOM 504 CA PRO A 30 52.085 52.883 47.906 1.00 1.84

ATOM 505 HA PRO A 30 52.298 51.817 47.824 1.00 1.84

ATOM 506 C PRO A 30 51.411 53.317 46.606 1.00 1.84

ATOM 507 O PRO A 30 50.823 52.480 45.935 1.00 1.84

ATOM 508 N GLN A 31 51.553 54.579 46.188 1.00 1.88

ATOM 509 H GLN A 31 52.035 55.235 46.783 1.00 1.88

ATOM 510 CA GLN A 31 51.088 55.006 44.864 1.00 1.88

ATOM 511 HA GLN A 31 50.006 54.864 44.813 1.00 1.88

ATOM 512 CB GLN A 31 51.386 56.497 44.619 1.00 1.88

ATOM 513 HB1 GLN A 31 51.169 56.709 43.571 1.00 1.88

ATOM 514 HB2 GLN A 31 52.448 56.688 44.779 1.00 1.88

ATOM 515 CG GLN A 31 50.541 57.465 45.465 1.00 1.88

ATOM 516 HG1 GLN A 31 49.485 57.229 45.337 1.00 1.88

ATOM 517 HG2 GLN A 31 50.706 58.476 45.093 1.00 1.88

ATOM 518 CD GLN A 31 50.884 57.451 46.952 1.00 1.88

ATOM 519 OE1 GLN A 31 50.056 57.167 47.807 1.00 1.88

ATOM 520 NE2 GLN A 31 52.114 57.743 47.321 1.00 1.88

ATOM 521 1HE2 GLN A 31 52.778 58.084 46.645 1.00 1.88

ATOM 522 2HE2 GLN A 31 52.273 57.839 48.309 1.00 1.88

ATOM 523 C GLN A 31 51.694 54.148 43.740 1.00 1.88

ATOM 524 O GLN A 31 50.987 53.629 42.872 1.00 1.88

ATOM 525 N LEU A 32 53.014 53.953 43.812 1.00 2.24

ATOM 526 H LEU A 32 53.501 54.280 44.633 1.00 2.24

ATOM 527 CA LEU A 32 53.783 53.170 42.855 1.00 2.24

ATOM 528 HA LEU A 32 53.513 53.515 41.857 1.00 2.24

ATOM 529 CB LEU A 32 55.285 53.430 43.088 1.00 2.24

ATOM 530 HB1 LEU A 32 55.575 52.984 44.039 1.00 2.24

ATOM 531 HB2 LEU A 32 55.447 54.507 43.161 1.00 2.24

ATOM 532 CG LEU A 32 56.215 52.876 41.994 1.00 2.24

ATOM 533 HG LEU A 32 56.066 51.801 41.888 1.00 2.24

ATOM 534 CD1 LEU A 32 55.986 53.552 40.640 1.00 2.24

ATOM 535 1HD1 LEU A 32 56.722 53.191 39.921 1.00 2.24

ATOM 536 2HD1 LEU A 32 54.995 53.303 40.263 1.00 2.24

ATOM 537 3HD1 LEU A 32 56.082 54.633 40.738 1.00 2.24

ATOM 538 CD2 LEU A 32 57.668 53.125 42.398 1.00 2.24

ATOM 539 1HD2 LEU A 32 58.333 52.715 41.638 1.00 2.24

ATOM 540 2HD2 LEU A 32 57.857 54.195 42.493 1.00 2.24

ATOM 541 3HD2 LEU A 32 57.877 52.630 43.343 1.00 2.24

ATOM 542 C LEU A 32 53.436 51.681 42.925 1.00 2.24

ATOM 543 O LEU A 32 53.315 51.052 41.882 1.00 2.24

ATOM 544 N VAL A 33 53.205 51.134 44.121 1.00 2.08

ATOM 545 H VAL A 33 53.329 51.710 44.947 1.00 2.08

ATOM 546 CA VAL A 33 52.763 49.733 44.268 1.00 2.08

ATOM 547 HA VAL A 33 53.473 49.093 43.741 1.00 2.08

ATOM 548 CB VAL A 33 52.746 49.299 45.749 1.00 2.08

ATOM 549 HB VAL A 33 52.052 49.932 46.302 1.00 2.08

ATOM 550 CG1 VAL A 33 52.321 47.835 45.914 1.00 2.08

ATOM 551 1HG1 VAL A 33 52.408 47.543 46.959 1.00 2.08

ATOM 552 2HG1 VAL A 33 51.280 47.707 45.617 1.00 2.08

ATOM 553 3HG1 VAL A 33 52.948 47.186 45.303 1.00 2.08

ATOM 554 CG2 VAL A 33 54.136 49.426 46.383 1.00 2.08

ATOM 555 1HG2 VAL A 33 54.058 49.225 47.449 1.00 2.08

ATOM 556 2HG2 VAL A 33 54.828 48.727 45.918 1.00 2.08

ATOM 557 3HG2 VAL A 33 54.519 50.436 46.268 1.00 2.08

ATOM 558 C VAL A 33 51.397 49.488 43.635 1.00 2.08

ATOM 559 O VAL A 33 51.207 48.508 42.921 1.00 2.08

ATOM 560 N CYS A 34 50.446 50.388 43.868 1.00 1.56

ATOM 561 H CYS A 34 50.647 51.155 44.501 1.00 1.56

ATOM 562 CA CYS A 34 49.081 50.267 43.374 1.00 1.56

ATOM 563 HA CYS A 34 48.743 49.245 43.551 1.00 1.56

ATOM 564 CB CYS A 34 48.210 51.201 44.209 1.00 1.56

ATOM 565 HB1 CYS A 34 47.191 51.119 43.853 1.00 1.56

ATOM 566 HB2 CYS A 34 48.561 52.230 44.099 1.00 1.56

ATOM 567 SG CYS A 34 48.157 50.767 45.963 1.00 1.56

ATOM 568 C CYS A 34 48.943 50.522 41.861 1.00 1.56

ATOM 569 O CYS A 34 48.010 50.005 41.230 1.00 1.56

ATOM 570 N ARG A 35 49.881 51.293 41.281 1.00 1.93

ATOM 571 H ARG A 35 50.526 51.784 41.895 1.00 1.93

ATOM 572 CA ARG A 35 50.088 51.388 39.828 1.00 1.93

ATOM 573 HA ARG A 35 49.113 51.507 39.353 1.00 1.93

ATOM 574 CB ARG A 35 50.950 52.629 39.516 1.00 1.93

ATOM 575 HB1 ARG A 35 52.010 52.372 39.577 1.00 1.93

ATOM 576 HB2 ARG A 35 50.759 53.385 40.280 1.00 1.93

ATOM 577 CG ARG A 35 50.666 53.321 38.168 1.00 1.93

ATOM 578 HG1 ARG A 35 51.312 54.199 38.120 1.00 1.93

ATOM 579 HG2 ARG A 35 49.636 53.682 38.157 1.00 1.93

ATOM 580 CD ARG A 35 50.935 52.484 36.910 1.00 1.93

ATOM 581 HD1 ARG A 35 51.737 51.773 37.120 1.00 1.93

ATOM 582 HD2 ARG A 35 51.276 53.157 36.120 1.00 1.93

ATOM 583 NE ARG A 35 49.727 51.784 36.426 1.00 1.93

ATOM 584 HE ARG A 35 48.835 52.071 36.799 1.00 1.93

ATOM 585 CZ ARG A 35 49.686 50.766 35.586 1.00 1.93

ATOM 586 NH1 ARG A 35 48.543 50.257 35.238 1.00 1.93

ATOM 587 1HH1 ARG A 35 47.701 50.609 35.680 1.00 1.93

ATOM 588 2HH1 ARG A 35 48.499 49.469 34.626 1.00 1.93

ATOM 589 NH2 ARG A 35 50.760 50.242 35.064 1.00 1.93

ATOM 590 1HH2 ARG A 35 51.653 50.589 35.355 1.00 1.93

ATOM 591 2HH2 ARG A 35 50.696 49.427 34.484 1.00 1.93

ATOM 592 C ARG A 35 50.685 50.093 39.259 1.00 1.93

ATOM 593 O ARG A 35 50.157 49.561 38.288 1.00 1.93

ATOM 594 N LEU A 36 51.750 49.573 39.873 1.00 2.74

ATOM 595 H LEU A 36 52.127 50.079 40.666 1.00 2.74

ATOM 596 CA LEU A 36 52.460 48.357 39.463 1.00 2.74

ATOM 597 HA LEU A 36 52.863 48.517 38.463 1.00 2.74

ATOM 598 CB LEU A 36 53.642 48.119 40.429 1.00 2.74

ATOM 599 HB1 LEU A 36 53.256 48.089 41.447 1.00 2.74

ATOM 600 HB2 LEU A 36 54.320 48.971 40.359 1.00 2.74

ATOM 601 CG LEU A 36 54.444 46.826 40.188 1.00 2.74

ATOM 602 HG LEU A 36 53.784 45.962 40.254 1.00 2.74

ATOM 603 CD1 LEU A 36 55.142 46.814 38.827 1.00 2.74

ATOM 604 1HD1 LEU A 36 55.748 45.913 38.737 1.00 2.74

ATOM 605 2HD1 LEU A 36 54.399 46.804 38.030 1.00 2.74

ATOM 606 3HD1 LEU A 36 55.782 47.689 38.726 1.00 2.74

ATOM 607 CD2 LEU A 36 55.509 46.689 41.279 1.00 2.74

ATOM 608 1HD2 LEU A 36 56.071 45.768 41.128 1.00 2.74

ATOM 609 2HD2 LEU A 36 56.193 47.537 41.242 1.00 2.74

ATOM 610 3HD2 LEU A 36 55.030 46.651 42.257 1.00 2.74

ATOM 611 C LEU A 36 51.526 47.145 39.384 1.00 2.74

ATOM 612 O LEU A 36 51.399 46.524 38.331 1.00 2.74

ATOM 613 N VAL A 37 50.800 46.877 40.468 1.00 3.05

ATOM 614 H VAL A 37 50.970 47.444 41.295 1.00 3.05

ATOM 615 CA VAL A 37 49.811 45.790 40.579 1.00 3.05

ATOM 616 HA VAL A 37 50.107 45.013 39.872 1.00 3.05

ATOM 617 CB VAL A 37 49.864 45.141 41.988 1.00 3.05

ATOM 618 HB VAL A 37 49.344 45.784 42.699 1.00 3.05

ATOM 619 CG1 VAL A 37 49.229 43.740 42.007 1.00 3.05

ATOM 620 1HG1 VAL A 37 49.289 43.321 43.011 1.00 3.05

ATOM 621 2HG1 VAL A 37 48.179 43.785 41.728 1.00 3.05

ATOM 622 3HG1 VAL A 37 49.750 43.084 41.310 1.00 3.05

ATOM 623 CG2 VAL A 37 51.313 44.939 42.483 1.00 3.05

ATOM 624 1HG2 VAL A 37 51.311 44.408 43.435 1.00 3.05

ATOM 625 2HG2 VAL A 37 51.880 44.361 41.754 1.00 3.05

ATOM 626 3HG2 VAL A 37 51.799 45.900 42.644 1.00 3.05

ATOM 627 C VAL A 37 48.415 46.263 40.127 1.00 3.05

ATOM 628 O VAL A 37 47.381 45.797 40.596 1.00 3.05

ATOM 629 N LEU A 38 48.404 47.217 39.188 1.00 3.31

ATOM 630 H LEU A 38 49.321 47.533 38.898 1.00 3.31

ATOM 631 CA LEU A 38 47.303 47.730 38.362 1.00 3.31

ATOM 632 HA LEU A 38 47.736 48.535 37.769 1.00 3.31

ATOM 633 CB LEU A 38 46.856 46.638 37.367 1.00 3.31

ATOM 634 HB1 LEU A 38 46.158 47.081 36.656 1.00 3.31

ATOM 635 HB2 LEU A 38 46.312 45.870 37.921 1.00 3.31

ATOM 636 CG LEU A 38 47.987 45.942 36.578 1.00 3.31

ATOM 637 HG LEU A 38 48.642 45.410 37.265 1.00 3.31

ATOM 638 CD1 LEU A 38 47.382 44.920 35.618 1.00 3.31

ATOM 639 1HD1 LEU A 38 48.177 44.404 35.082 1.00 3.31

ATOM 640 2HD1 LEU A 38 46.814 44.182 36.183 1.00 3.31

ATOM 641 3HD1 LEU A 38 46.724 45.408 34.901 1.00 3.31

ATOM 642 CD2 LEU A 38 48.828 46.924 35.756 1.00 3.31

ATOM 643 1HD2 LEU A 38 49.593 46.365 35.216 1.00 3.31

ATOM 644 2HD2 LEU A 38 48.188 47.445 35.049 1.00 3.31

ATOM 645 3HD2 LEU A 38 49.332 47.623 36.422 1.00 3.31

ATOM 646 C LEU A 38 46.099 48.378 39.061 1.00 3.31

ATOM 647 O LEU A 38 45.435 49.210 38.446 1.00 3.31

ATOM 648 N ARG A 39 45.808 48.051 40.317 1.00 2.49

ATOM 649 H ARG A 39 46.450 47.400 40.756 1.00 2.49

ATOM 650 CA ARG A 39 44.475 48.215 40.925 1.00 2.49

ATOM 651 HA ARG A 39 43.725 47.786 40.255 1.00 2.49

ATOM 652 CB ARG A 39 44.496 47.402 42.240 1.00 2.49

ATOM 653 HB1 ARG A 39 45.266 47.815 42.893 1.00 2.49

ATOM 654 HB2 ARG A 39 44.782 46.378 41.994 1.00 2.49

ATOM 655 CG ARG A 39 43.171 47.350 43.022 1.00 2.49

ATOM 656 HG1 ARG A 39 42.368 47.015 42.366 1.00 2.49

ATOM 657 HG2 ARG A 39 42.929 48.343 43.400 1.00 2.49

ATOM 658 CD ARG A 39 43.284 46.381 44.213 1.00 2.49

ATOM 659 HD1 ARG A 39 44.156 46.660 44.807 1.00 2.49

ATOM 660 HD2 ARG A 39 43.427 45.372 43.822 1.00 2.49

ATOM 661 NE ARG A 39 42.082 46.420 45.066 1.00 2.49

ATOM 662 HE ARG A 39 41.352 47.072 44.788 1.00 2.49

ATOM 663 CZ ARG A 39 41.790 45.646 46.091 1.00 2.49

ATOM 664 NH1 ARG A 39 40.699 45.846 46.766 1.00 2.49

ATOM 665 1HH1 ARG A 39 40.074 46.563 46.442 1.00 2.49

ATOM 666 2HH1 ARG A 39 40.368 45.167 47.441 1.00 2.49

ATOM 667 NH2 ARG A 39 42.546 44.653 46.471 1.00 2.49

ATOM 668 1HH2 ARG A 39 43.369 44.432 45.939 1.00 2.49

ATOM 669 2HH2 ARG A 39 42.279 44.090 47.259 1.00 2.49

ATOM 670 C ARG A 39 44.047 49.670 41.167 1.00 2.49

ATOM 671 O ARG A 39 42.861 49.950 41.277 1.00 2.49

ATOM 672 N CYS A 40 45.007 50.593 41.234 1.00 2.00

ATOM 673 H CYS A 40 45.964 50.268 41.156 1.00 2.00

ATOM 674 CA CYS A 40 44.761 52.039 41.272 1.00 2.00

ATOM 675 HA CYS A 40 44.017 52.269 42.035 1.00 2.00

ATOM 676 CB CYS A 40 46.104 52.703 41.627 1.00 2.00

ATOM 677 HB1 CYS A 40 46.852 52.365 40.908 1.00 2.00

ATOM 678 HB2 CYS A 40 46.392 52.362 42.603 1.00 2.00

ATOM 679 SG CYS A 40 46.213 54.511 41.671 1.00 2.00

ATOM 680 C CYS A 40 44.277 52.628 39.942 1.00 2.00

ATOM 681 O CYS A 40 43.674 53.697 39.924 1.00 2.00

ATOM 682 N SER A 41 44.619 51.978 38.829 1.00 2.92

ATOM 683 H SER A 41 45.000 51.048 38.917 1.00 2.92

ATOM 684 CA SER A 41 44.700 52.621 37.524 1.00 2.92

ATOM 685 HA SER A 41 45.353 53.489 37.617 1.00 2.92

ATOM 686 CB SER A 41 45.311 51.690 36.472 1.00 2.92

ATOM 687 HB1 SER A 41 45.538 52.256 35.567 1.00 2.92

ATOM 688 HB2 SER A 41 44.605 50.894 36.226 1.00 2.92

ATOM 689 OG SER A 41 46.503 51.112 36.980 1.00 2.92

ATOM 690 HG SER A 41 46.190 50.401 37.580 1.00 2.92

ATOM 691 C SER A 41 43.338 53.163 37.058 1.00 2.92

ATOM 692 O SER A 41 42.300 53.065 37.712 1.00 2.92

TER

# ModelArchive (<https://modelarchive.org>/doi/105452/ma-axqi2) > Procedures & Data

# S1 SMB: Mass Spectral Analysis, disulflide CD, Homology Templating

# Procedures & Data

**Mass spectrometry Data of SMB**

1. ***Mass spectrometry Data of intact SMB***

*Comparison of Reduced and Oxidized Intact Super Mini B (SMB) in Structure Promoting Solvent Systems*


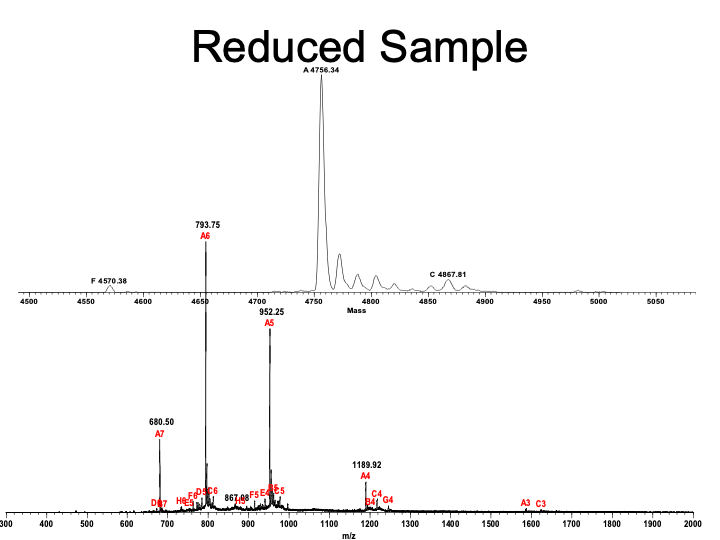


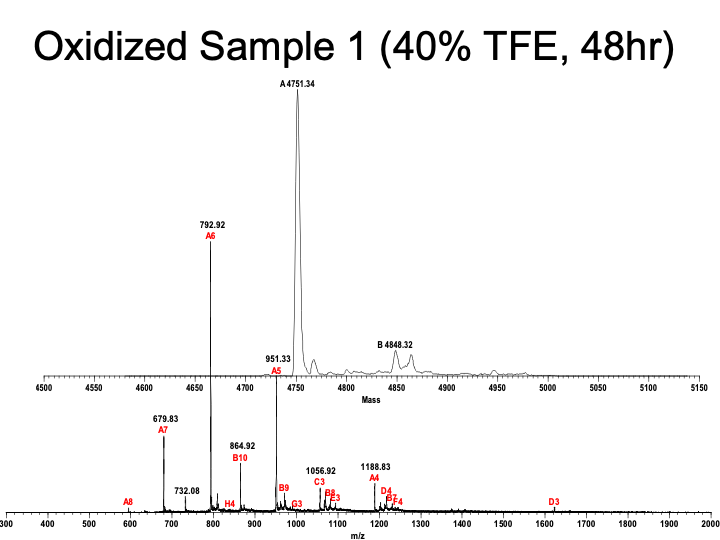


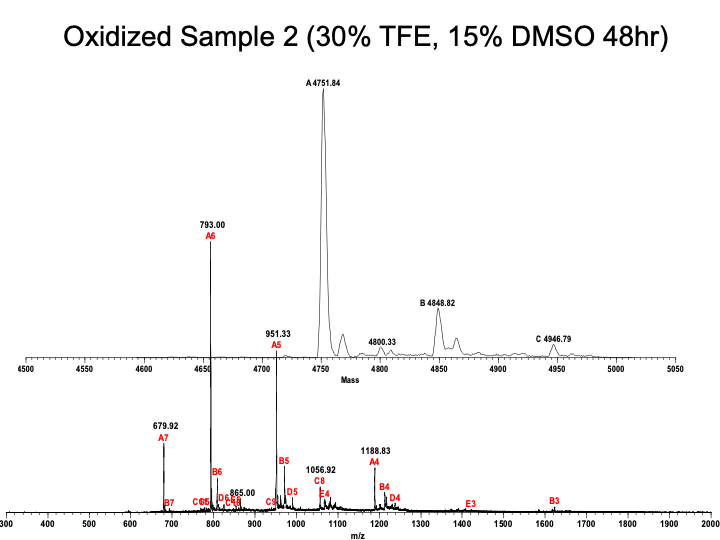


1. ***SMB peptides following pepsin digest of parent protein to determine disulfide connectivity:***

*Peptide spectra were collected at high resolution using an orbitrap mass spectrometer.*

1. Mass spectrum of crosslinked peptides from Figure 3, lefthand side.

1. Mass spectrum of crosslinked peptides from Figure 3, righthand side.

**Circular Dichroic Spectral Data for oxidized Mini-B (MB) peptide in SDS micelles 250 nm to 350 nm spanning the dichroic disulfide signature wavelength region. Spectral data is an average of 32 spectral scans with a Chiroscan CD spectrometer (Applied Photophysics**

Wavelength Absorbance

(nm) (MRE x 10E3)

350 -0.130289

349 -0.0740173

348 -0.180709

347 -0.0886155

346 -0.134787

345 -0.171822

344 -0.17172

343 -0.129463

342 -0.212495

341 -0.150682

340 -0.168245

339 -0.122692

338 -0.177589

337 -0.178234

336 -0.198268

335 -0.138241

334 -0.251598

333 -0.138521

332 -0.194156

331 -0.227941

330 -0.245936

329 -0.146683

328 -0.216051

327 -0.166308

326 -0.16691

325 -0.148133

324 -0.207625

323 -0.166313

322 -0.237329

321 -0.141749

320 -0.207165

319 -0.145035

318 -0.139241

317 -0.140659

316 -0.213578

315 -0.186493

314 -0.20914

313 -0.244478

312 -0.182016

311 -0.264492

310 -0.227984

309 -0.289889

308 -0.224509

307 -0.208736

306 -0.191351

305 -0.268343

304 -0.154375

303 -0.21807

302 -0.269976

301 -0.229441

300 -0.242983

299 -0.201692

298 -0.245391

297 -0.291427

296 -0.19536

295 -0.28223

294 -0.362753

293 -0.279235

292 -0.260254

291 -0.335214

290 -0.337156

289 -0.318398

288 -0.2904

287 -0.308874

286 -0.385589

285 -0.386562

284 -0.432541

283 -0.401221

282 -0.35538

281 -0.325047

280 -0.32902

279 -0.385686

278 -0.329615

277 -0.383926

276 -0.453924

275 -0.413741

274 -0.456846

273 -0.507723

272 -0.466228

271 -0.56793

270 -0.457196

269 -0.543096

268 -0.633879

267 -0.666644

266 -0.650069

265 -0.642513

264 -0.665834

263 -0.627354

262 -0.680199

261 -0.737269

260 -0.6489

*Circular Dichroic Spectral Data for SMB in SDS micelles 250 nm to 350 nm*

Wavelength Absorbance

(nm) (MRE x 10E3)

350 -0.135575

349 -0.101489

348 -0.137014

347 -0.12337

346 -0.144831

345 -0.133763

344 -0.122415

343 -0.0488434

342 -0.162897

341 -0.0936837

340 -0.0892443

339 -0.0807906

338 -0.0782639

337 -0.169649

336 -0.147726

335 -0.144148

334 -0.137757

333 -0.161629

332 -0.113513

331 -0.112171

330 -0.0128315

329 -0.12966

328 -0.14419

327 -0.0936998

326 -0.202438

325 -0.127498

324 -0.149606

323 -0.175067

322 -0.180165

321 -0.141027

320 -0.161627

319 -0.158366

318 -0.183974

317 -0.133949

316 -0.0906271

315 -0.166927

314 -0.135266

313 -0.162575

312 -0.165273

311 -0.133627

310 -0.138711

309 -0.245176

308 -0.215465

307 -0.210343

306 -0.173359

305 -0.17833

304 -0.241871

303 -0.154148

302 -0.197184

301 -0.133791

300 -0.191975

299 -0.0414423

298 -0.117726

297 -0.173827

296 -0.14482

295 -0.171626

294 -0.195894

293 -0.189263

292 -0.165587

291 -0.286307

290 -0.199113

289 -0.32135

288 -0.270006

287 -0.173123

286 -0.29719

285 -0.275942

284 -0.190432

283 -0.283484

282 -0.227772

281 -0.367898

280 -0.302808

279 -0.300614

278 -0.291916

277 -0.278415

276 -0.272228

275 -0.327815

274 -0.387351

273 -0.478753

272 -0.519486

271 -0.382803

270 -0.460735

269 -0.559613

268 -0.508031

267 -0.525863

266 -0.560142

265 -0.640278

264 -0.621052

263 -0.667671

262 -0.619816

261 -0.745541

260 -0.800493

**Figure 4A. Predicted SMB Secondary Structure using Homology Modeling Refined by Constrained Disulfides based on CD and Mass Spectral Analysis experimental measurements.**

*SMB preliminary secondary structure determination by homology modeling*

*Input primary amino acid sequence in FASTA format for initial peptide model*

# SMB iTasser with Distance constraint Deposition Data

*Predicted SMB Preliminary Structure Homology Modeling using iTasser with disulfide distance constraints*

**Input FASTA sequence**

>SMB_cys_distance_constraits

FPIPLPYCWLCRALIKRIQAMIPKGGRMLPQLVCRLVLRCS

REMARK : The file contains contact/distance restraints specified by the user.

REMARK : Read about assigning restraints during the structure modeling at: <http://zhanglab.ccmb.med.umich.edu/I-TASSER/restraint.html>

**Input SMB Disulfide Distance Constraints**

Residue # Atom Type Residue # Atom Type Distance (Angstroms)

DIST 8 SG 40 SG 2.05

DIST 11 SG 34 SG 2.05

**Name C-score Exp.TM-Score Exp.RMSD No.of decoys Cluster density**

Model1: -0.09 0.70+-0.12 2.4+-1.8 8843 0.4014

Model2: -1.60 1725 0.0885

Model3: -3.23 415 0.0174

Model4: -0.77 3556 0.2027

Model5: -4.29 151 0.0060

C-score is a confidence score for estimating the quality of predicted models by I-TASSER. It is calculated based on the significance of threading template alignments and the convergence parameters of the structure assembly simulations. C-score is typically in the range of [-5,2], where a C-score of higher value signifies

a model with a high confidence and vice-versa.

TM-score and RMSD are known standards for measuring structural similarity between two structures which are usually used to measure the accuracy of structure modeling when the native structure is known. In case where the native structure is not known, it becomes necessary to predict the quality of the modeling prediction, i.e. what is the distance between the predicted model and the native structures? To answer this

question, we tried predicted the TM-score and RMSD of the predicted models relative the native structures based on the C-score.

In a benchmark test set of 500 non-homologous proteins, we found that C-score is highly correlated with TM-score and RMSD. Correlation coefficient of C-score of the first model with TM-score to the native structure is 0.91, while the coefficient of C-score with RMSD to the native structure is 0.75. These data actually lay the base for the reliable prediction of the TM-score and RMSD using C-score. Values reported

in Column 3 & 4 are the estimated values of TM-score and RMSD based on their correlation with C-score. Here we only report the quality prediction (TM-score and RMSD) for the first model, because we found that the correlation between C-score and TM-score is weak for lower rank models. However, we list the C-score

of all models just for a reference.

What is TM-score?

TM-score is a recently proposed scale for measuring the structural similarity between two structures (see Zhang and Skolnick, Scoring function for automated assessment of protein structure template quality, Proteins, 2004 57: 702-710). The purpose of proposing TM-score is to solve the problem of RMSD which is sensitive to the local error. Because RMSD is an average distance of all residue pairs in two structures,

a local error (e.g. a misorientation of the tail) will araise a big RMSD value although the global topology is correct. In TM-score, however, the small distance is weighted stronger than the big distance which makes the score insensitive to the local modeling error. A TM-score >0.5 indicates a model of correct topology and a TM-score<0.17 means a random similarity. These cutoff does not depends on the protein length.

What is Cluster density?

I-TASSER generates full length model of proteins by excising continuous fragments from threading alignments and then reassembling them using replica-exchanged Monte Carlo simulations. Low temperature replicas (decoys) generated during the simulation are clustered by SPICKER and top five cluster centroids are selected for

generating full atomic models. The cluster density is defined as the number of structure decoys at an unit of space in the SPICKER cluster. A higher cluster density means the structure occurs more often in the simulation trajectory and therefore signifies a better quality model. The values in the second last columns of the above mentioned table repesents the number of structural decoys that are used in generating

each model. The last column represents the density of cluster.

Reference citations for use of the I-TASSER server:

1) Jianyi Yang, Renxiang Yan, Ambrish Roy, Dong Xu, Jonathan Poisson, Yang Zhang. The I-TASSER Suite: Protein structure and function prediction. Nature Methods, 12: 7-8 (2015).

2) Ambrish Roy, Alper Kucukural, Yang Zhang. I-TASSER: a unified platform for automated protein structure and function prediction. Nature Protocols, 5: 725-738 (2010).

3) Yang Zhang. I-TASSER server for protein 3D structure prediction. BMC Bioinformatics, 9:40 (2008).

%%%%%%%%%%%%%%%%%%%%%%%%%%%%%%%%%%%%%%%%%%%

% B-factor and local structure quality estimation %

% in I-TASSER structure modeling %%%%%%%%%%%%%%%%%%%%%%%%%%%%%%%%%%%%%%%%%%%

1. Local accuracy estimation

The local accuracy was defined as the distance deviation (in Angstrom) between residue positions in the model and the native structure. It was estimated using support vector regression that makes use of the coverage of threading alignment, divergence of I-TASSER simulation decoys, and sequence-based secondary structure and solvent accessibility predictions. Large-scale benchmark tests show that the estimated local accuracy has an average error of 2.21 Angstrom and the Pearson's correlation coefficient between estimated and actual error is 0.7.

Based on these tests, the local accuracy estimations tend to be more accurate for residues:

1) that have higher threading alignment coverage

2) that are located at alpha-helix and beta-strand regions

3) that are buried (at 25% threshold)

The estimated local accuracy for each model is available at the columns 61-66 in the model's PDB file and also at the bottom of this page (columns with label RSQ_*).

2. Normalized B-factor

Normalized B-factor for a target protein is defined as z-score-based normalization of the raw B-factor values. The normalized B-factor (called B-factor profile, BFP) is predicted using a combination of both template-based assignment and profile-based prediction. Based on the distributions and predictions of the BFP, residues with BFP values higher than 0 are less stable in experimental structures. The estimated normalized B-factor is shown at the bottom of this page.

For more information about the local accuracy and normalized B-factor predictions, please refer to the following article:

J Yang, Y Wang, Y Zhang. ResQ: An approach to unified estimation of B-factor and residue-specific error in protein structure prediction, Journal of Molecular Biology, 428: 693-701 (2016).

#**RES SS SA COV BFP RSQ_1 RSQ_2 RSQ_3 RSQ_4 RSQ_5**

1 C E 0.39 1.75 7.08 11.24 13.08 7.34 14.00

2 C E 0.40 1.24 5.89 9.35 11.45 6.15 12.08

3 C E 0.51 0.60 4.62 7.61 9.75 5.37 10.11

4 C E 0.63 0.26 3.89 6.50 8.82 4.76 8.84

5 C E 0.69 -0.16 3.09 5.83 7.78 4.34 7.10

6 C E 0.72 -0.31 3.02 6.76 8.00 4.42 7.47

7 H E 0.71 -0.46 2.45 5.74 7.06 3.64 7.74

8 H B 0.85 -0.61 1.55 5.00 6.30 3.09 6.58

9 H E 0.87 -0.63 1.80 5.50 6.86 3.03 6.37

10 H E 0.88 -0.62 2.02 5.38 6.33 3.10 6.23

11 H B 0.91 -0.73 1.58 4.61 5.77 2.87 5.91

12 H E 0.98 -0.54 1.31 4.64 5.66 2.66 5.26

13 H E 0.98 -0.51 1.77 5.09 5.96 2.93 5.31

14 H B 0.98 -0.63 1.75 4.84 5.64 2.98 5.81

15 H B 0.98 -0.60 1.33 4.10 5.10 2.75 5.18

16 H E 0.98 -0.30 1.35 4.61 5.45 2.77 4.88

17 H E 0.97 -0.40 1.76 5.11 5.75 3.14 5.47

18 H B 0.97 -0.49 1.63 4.66 5.33 3.10 5.42

19 H E 0.99 -0.14 1.17 3.99 4.96 2.75 5.00

20 H E 0.98 -0.07 1.53 4.69 5.47 3.11 5.78

21 H E 0.98 -0.02 1.84 4.96 5.68 3.40 6.01

22 C B 0.93 -0.21 1.73 4.66 5.67 3.31 5.96

23 C E 0.93 -0.05 1.75 4.66 6.21 3.27 6.18

24 C E 0.95 0.28 2.45 5.43 7.10 3.83 6.67

25 C E 0.93 0.31 2.99 6.10 7.27 3.83 7.11

26 C E 0.89 0.37 2.34 5.36 6.60 3.81 6.78

27 C E 0.86 0.20 2.24 5.29 6.36 3.77 6.43

28 C E 0.84 -0.03 1.95 4.75 6.77 3.26 6.06

29 C B 0.83 -0.31 2.48 5.15 7.14 4.06 7.09

30 H B 0.82 -0.09 1.84 4.78 6.45 3.48 5.90

31 H E 0.85 -0.01 1.88 4.90 7.14 3.41 6.11

32 H B 0.83 -0.17 2.24 4.80 6.98 3.56 5.91

33 H B 0.83 -0.16 2.08 4.36 5.77 3.54 5.66

34 H B 0.82 -0.13 1.56 4.25 5.46 3.10 5.91

35 H E 0.82 0.19 1.93 4.54 6.30 3.24 6.24

36 H B 0.81 0.40 2.74 5.11 6.74 3.67 6.43

37 H B 0.75 0.19 3.05 5.63 6.79 4.14 7.11

38 S E 0.72 0.66 3.31 5.84 6.98 4.53 7.29

39 S E 0.72 1.00 2.49 5.27 6.18 3.96 7.78

40 C B 0.72 1.41 2.00 4.68 6.18 3.60 7.37

41 C E 0.62 2.53 2.92 5.66 7.40 4.64 8.23

Model structural quality of the iTasser predicted structure with disulfide distance constraints analyzed by PROCHECK (Laskowski et al., 1993, 1996) generated with PDBsum (<https://ebi.ac.uk>).


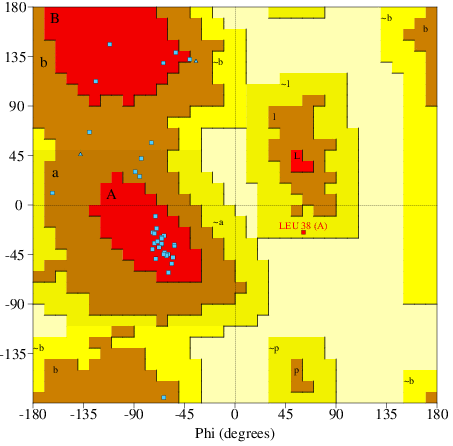


**PROCHECK statistics**

**1. Ramachandran Plot statistics**

**No. of**

**residues %-tage**

**------ ------**

Most favoured regions [A,B,L] 27 84.4%*****

Additional allowed regions [a,b,l,p] 4 12.5%

Generously allowed regions [~a,~b,~l,~p] 1 3.1%

Disallowed regions [XX] 0 0.0%

---- ------

Non-glycine and non-proline residues 32 100.0%

End-residues (excl. Gly and Pro) 2

Glycine residues 2

Proline residues 5

----

Total number of residues 41

Based on an analysis of **118** structures of resolution of at least **2.0** Angstroms and *R*-factor no greater than **20.0** a good quality model would be expected to have over **90%** in the most favoured regions [A,B,L].

**2. G-Factors**

**Average**

**Parameter Score Score**

**--------- ----- -----**

Dihedral angles:-

Phi-psi distribution -0.47

Chi1-chi2 distribution -0.10

Chi1 only 0.06

Chi3 & chi4 0.39

Omega  **-1.37****

-0.50*

=====

Main-chain covalent forces:-

Main-chain bond lengths 0.43

Main-chain bond angles -0.28

0.02

=====

OVERALL AVERAGE -0.28

=====

**G-factors** provide a measure of how **unusual**, or out-of-the-ordinary, a property is.

Values below -0.5* - unusual

Values below **-1.0**** - highly unusual

**Important note:** The main-chain bond-lengths and bond angles are compared with the Engh & Huber (1991) ideal values derived from small-molecule data. Therefore, structures refined using different restraints may show apparently large deviations from normality

| **Disulphides** |
| --- |
|  |
| \|  \| \| \| \| \| \| \| \| \| \| \| \| \| \| \| \| --- \| --- \| --- \| --- \| --- \| --- \| --- \| --- \| --- \| --- \| --- \| --- \| --- \| --- \| --- \| \| **1st cysteine** \|  \| **2nd cysteine** \|  \| **Type** \|  \| **Chi1** \|  \| **Chi2** \|  \| **Chi3** \|  \| **Chi2p** \|  \| **Chi1p** \| \|  \| \| \| \| \| \| \| \| \| \| \| \| \| \| \| \| A 8 \|  \| A 40 \|  \|  \|  \| -164.2 \|  \| 114.9 \|  \| -77.6 \|  \| 90.8 \|  \| 176.5 \| \| A 11 \|  \| A 34 \|  \| LHS \|  \| -168.9 \|  \| -95.9 \|  \| -92.5 \|  \| -83.3 \|  \| -62.7 \| \|  \| \| \| \| \| \| \| \| \| \| \| \| \| \| \| |
|  |


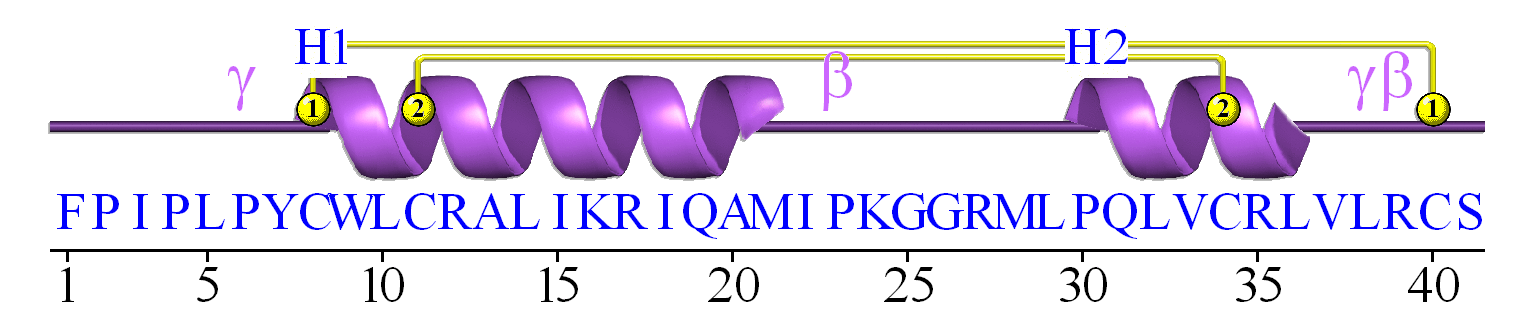


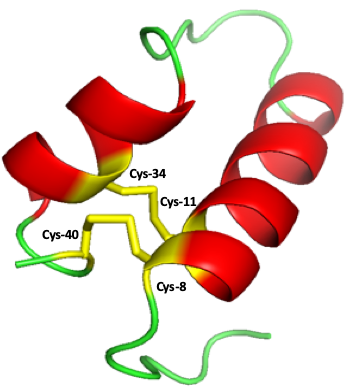

Supplement: S1 File — (DOCX) [file pone.0276787.s001.docx]
