# Supplementary material for: Emulation of the structure of the Saposin protein fold by a lung surfactant peptide construct of surfactant Protein B
Source: PLoS One. 2022 Nov 3;17(11):e0276787. doi: 10.1371/journal.pone.0276787 (PMC9632872; doi:10.1371/journal.pone.0276787)

**S2** **- deposited in the ModelArchive (**<https://modelarchive.org>/doi/10.5452/ma-scodz**)**

**Emulation of the Structure of the Saposin Protein Fold by a Lung Surfactant Peptide Construct of Surfactant Protein B**

Alan J. Waring^1,2^, Julian P. Whitelegge^3^, Shantanu K. Sharma^4^, Larry M. Gordon^1^,

Frans J. Walther^1,5,*^

^1^ Lundquist Institute for Biomedical Innovation at Harbor-UCLA Medical Center

1124 West Carson Street

Torrance, CA, USA

^2^ Department of Medicine

David Geffen School of Medicine

University of California Los Angeles

405 Hilgard Avenue

Los Angeles, CA, USA

^3^ NPI-Semel Institute for Neuroscience & Human Behavior Department of Psychiatry

& Biobehavioral Sciences, David Geffen School of Medicine at UCLA,

760 Westwood Plaza, Los Angeles, CA, USA

^4^ Materials and Process Simulation Center

California Institute of Technology

1200 East California Boulevard

Pasadena, CA, USA

^5^ Department of Pediatrics

David Geffen School of Medicine

University of California Los Angeles

405 Hilgard Avenue

Los Angeles, CA, USA

# SMB Peptide AlphaFold Protocol/Deposition Data

**Predicted Super Mini B (SMB) Structure by AI Modeling using AlphaFold**

# ModelArchive (<https://modelarchive.org>/doi/10.5452/scodz)

**AlphaFold Output PDB file**

SEQRES 1 A 41 PHE PRO ILE PRO LEU PRO TYR CYS TRP LEU CYS ARG ALA

SEQRES 2 A 41 LEU ILE LYS ARG ILE GLN ALA MET ILE PRO LYS GLY GLY

SEQRES 3 A 41 ARG MET LEU PRO GLN LEU VAL CYS ARG LEU VAL LEU ARG

SEQRES 4 A 41 CYS SER

HELIX 1 1 LEU A 5 MET A 21 1 17

HELIX 2 2 MET A 28 LEU A 36 1 9

ATOM 1 N PHE A 1 4.800 9.011 -17.896 1.00 57.18 N

ATOM 2 H PHE A 1 5.159 9.614 -18.622 1.00 57.18 H

ATOM 3 H2 PHE A 1 4.437 8.165 -18.311 1.00 57.18 H

ATOM 4 H3 PHE A 1 4.054 9.490 -17.411 1.00 57.18 H

ATOM 5 CA PHE A 1 5.869 8.694 -16.933 1.00 57.18 C

ATOM 6 HA PHE A 1 6.667 8.149 -17.438 1.00 57.18 H

ATOM 7 C PHE A 1 5.273 7.807 -15.843 1.00 57.18 C

ATOM 8 CB PHE A 1 6.456 10.004 -16.378 1.00 57.18 C

ATOM 9 HB2 PHE A 1 5.796 10.431 -15.623 1.00 57.18 H

ATOM 10 HB3 PHE A 1 6.506 10.730 -17.189 1.00 57.18 H

ATOM 11 O PHE A 1 4.527 8.329 -15.020 1.00 57.18 O

ATOM 12 CG PHE A 1 7.855 9.875 -15.816 1.00 57.18 C

ATOM 13 CD1 PHE A 1 8.064 9.678 -14.438 1.00 57.18 C

ATOM 14 HD1 PHE A 1 7.226 9.594 -13.761 1.00 57.18 H

ATOM 15 CD2 PHE A 1 8.959 9.994 -16.683 1.00 57.18 C

ATOM 16 HD2 PHE A 1 8.818 10.169 -17.740 1.00 57.18 H

ATOM 17 CE1 PHE A 1 9.374 9.606 -13.932 1.00 57.18 C

ATOM 18 HE1 PHE A 1 9.541 9.463 -12.874 1.00 57.18 H

ATOM 19 CE2 PHE A 1 10.266 9.919 -16.175 1.00 57.18 C

ATOM 20 HE2 PHE A 1 11.114 10.022 -16.836 1.00 57.18 H

ATOM 21 CZ PHE A 1 10.474 9.727 -14.800 1.00 57.18 C

ATOM 22 HZ PHE A 1 11.479 9.678 -14.409 1.00 57.18 H

ATOM 23 N PRO A 2 5.443 6.474 -15.885 1.00 63.75 N

ATOM 24 CA PRO A 2 4.992 5.626 -14.785 1.00 63.75 C

ATOM 25 HA PRO A 2 3.936 5.796 -14.574 1.00 63.75 H

ATOM 26 C PRO A 2 5.822 5.979 -13.546 1.00 63.75 C

ATOM 27 CB PRO A 2 5.185 4.185 -15.264 1.00 63.75 C

ATOM 28 HB2 PRO A 2 5.422 3.507 -14.444 1.00 63.75 H

ATOM 29 HB3 PRO A 2 4.285 3.852 -15.782 1.00 63.75 H

ATOM 30 O PRO A 2 7.046 5.887 -13.565 1.00 63.75 O

ATOM 31 CG PRO A 2 6.336 4.290 -16.266 1.00 63.75 C

ATOM 32 HG2 PRO A 2 6.275 3.518 -17.033 1.00 63.75 H

ATOM 33 HG3 PRO A 2 7.286 4.219 -15.737 1.00 63.75 H

ATOM 34 CD PRO A 2 6.189 5.694 -16.863 1.00 63.75 C

ATOM 35 HD2 PRO A 2 5.626 5.635 -17.794 1.00 63.75 H

ATOM 36 HD3 PRO A 2 7.176 6.119 -17.043 1.00 63.75 H

ATOM 37 N ILE A 3 5.160 6.464 -12.497 1.00 66.18 N

ATOM 38 H ILE A 3 4.154 6.532 -12.547 1.00 66.18 H

ATOM 39 CA ILE A 3 5.805 6.778 -11.218 1.00 66.18 C

ATOM 40 HA ILE A 3 6.615 7.481 -11.411 1.00 66.18 H

ATOM 41 C ILE A 3 6.382 5.461 -10.676 1.00 66.18 C

ATOM 42 CB ILE A 3 4.787 7.441 -10.265 1.00 66.18 C

ATOM 43 HB ILE A 3 3.936 6.766 -10.179 1.00 66.18 H

ATOM 44 O ILE A 3 5.703 4.431 -10.749 1.00 66.18 O

ATOM 45 CG1 ILE A 3 4.304 8.789 -10.854 1.00 66.18 C

ATOM 46 HG12 ILE A 3 4.995 9.586 -10.579 1.00 66.18 H

ATOM 47 HG13 ILE A 3 4.272 8.753 -11.943 1.00 66.18 H

ATOM 48 CG2 ILE A 3 5.343 7.684 -8.849 1.00 66.18 C

ATOM 49 HG21 ILE A 3 6.136 8.431 -8.867 1.00 66.18 H

ATOM 50 HG22 ILE A 3 4.550 8.051 -8.198 1.00 66.18 H

ATOM 51 HG23 ILE A 3 5.720 6.754 -8.423 1.00 66.18 H

ATOM 52 CD1 ILE A 3 2.894 9.152 -10.389 1.00 66.18 C

ATOM 53 HD11 ILE A 3 2.850 9.215 -9.302 1.00 66.18 H

ATOM 54 HD12 ILE A 3 2.189 8.399 -10.743 1.00 66.18 H

ATOM 55 HD13 ILE A 3 2.612 10.118 -10.808 1.00 66.18 H

ATOM 56 N PRO A 4 7.633 5.452 -10.190 1.00 71.40 N

ATOM 57 CA PRO A 4 8.347 4.222 -9.885 1.00 71.40 C

ATOM 58 HA PRO A 4 8.465 3.650 -10.806 1.00 71.40 H

ATOM 59 C PRO A 4 7.569 3.365 -8.876 1.00 71.40 C

ATOM 60 CB PRO A 4 9.736 4.675 -9.407 1.00 71.40 C

ATOM 61 HB2 PRO A 4 10.151 4.045 -8.620 1.00 71.40 H

ATOM 62 HB3 PRO A 4 10.417 4.688 -10.257 1.00 71.40 H

ATOM 63 O PRO A 4 7.090 3.859 -7.855 1.00 71.40 O

ATOM 64 CG PRO A 4 9.515 6.107 -8.926 1.00 71.40 C

ATOM 65 HG2 PRO A 4 9.096 6.098 -7.920 1.00 71.40 H

ATOM 66 HG3 PRO A 4 10.431 6.696 -8.964 1.00 71.40 H

ATOM 67 CD PRO A 4 8.475 6.605 -9.918 1.00 71.40 C

ATOM 68 HD2 PRO A 4 8.964 6.919 -10.841 1.00 71.40 H

ATOM 69 HD3 PRO A 4 7.924 7.439 -9.485 1.00 71.40 H

ATOM 70 N LEU A 5 7.492 2.058 -9.149 1.00 77.80 N

ATOM 71 H LEU A 5 7.883 1.758 -10.031 1.00 77.80 H

ATOM 72 CA LEU A 5 6.889 1.018 -8.304 1.00 77.80 C

ATOM 73 HA LEU A 5 5.813 1.039 -8.477 1.00 77.80 H

ATOM 74 C LEU A 5 7.088 1.178 -6.775 1.00 77.80 C

ATOM 75 CB LEU A 5 7.414 -0.367 -8.733 1.00 77.80 C

ATOM 76 HB2 LEU A 5 8.499 -0.402 -8.637 1.00 77.80 H

ATOM 77 HB3 LEU A 5 7.013 -1.085 -8.018 1.00 77.80 H

ATOM 78 O LEU A 5 6.131 0.894 -6.046 1.00 77.80 O

ATOM 79 CG LEU A 5 6.999 -0.823 -10.149 1.00 77.80 C

ATOM 80 HG LEU A 5 6.060 -0.344 -10.426 1.00 77.80 H

ATOM 81 CD1 LEU A 5 8.060 -0.499 -11.205 1.00 77.80 C

ATOM 82 HD11 LEU A 5 7.739 -0.884 -12.173 1.00 77.80 H

ATOM 83 HD12 LEU A 5 8.200 0.578 -11.304 1.00 77.80 H

ATOM 84 HD13 LEU A 5 9.009 -0.967 -10.944 1.00 77.80 H

ATOM 85 CD2 LEU A 5 6.782 -2.337 -10.164 1.00 77.80 C

ATOM 86 HD21 LEU A 5 7.702 -2.852 -9.890 1.00 77.80 H

ATOM 87 HD22 LEU A 5 6.481 -2.657 -11.162 1.00 77.80 H

ATOM 88 HD23 LEU A 5 5.992 -2.610 -9.464 1.00 77.80 H

ATOM 89 N PRO A 6 8.241 1.643 -6.237 1.00 81.87 N

ATOM 90 CA PRO A 6 8.390 1.923 -4.803 1.00 81.87 C

ATOM 91 HA PRO A 6 8.374 0.984 -4.251 1.00 81.87 H

ATOM 92 C PRO A 6 7.304 2.832 -4.220 1.00 81.87 C

ATOM 93 CB PRO A 6 9.774 2.573 -4.645 1.00 81.87 C

ATOM 94 HB2 PRO A 6 10.486 1.823 -4.300 1.00 81.87 H

ATOM 95 HB3 PRO A 6 9.765 3.417 -3.955 1.00 81.87 H

ATOM 96 O PRO A 6 6.837 2.586 -3.109 1.00 81.87 O

ATOM 97 CG PRO A 6 10.147 3.015 -6.057 1.00 81.87 C

ATOM 98 HG2 PRO A 6 11.227 3.078 -6.193 1.00 81.87 H

ATOM 99 HG3 PRO A 6 9.660 3.960 -6.296 1.00 81.87 H

ATOM 100 CD PRO A 6 9.536 1.879 -6.864 1.00 81.87 C

ATOM 101 HD2 PRO A 6 10.148 0.985 -6.740 1.00 81.87 H

ATOM 102 HD3 PRO A 6 9.519 2.123 -7.926 1.00 81.87 H

ATOM 103 N TYR A 7 6.842 3.838 -4.964 1.00 83.90 N

ATOM 104 H TYR A 7 7.195 3.954 -5.903 1.00 83.90 H

ATOM 105 CA TYR A 7 5.789 4.745 -4.502 1.00 83.90 C

ATOM 106 HA TYR A 7 6.076 5.154 -3.533 1.00 83.90 H

ATOM 107 C TYR A 7 4.460 4.011 -4.301 1.00 83.90 C

ATOM 108 CB TYR A 7 5.654 5.901 -5.501 1.00 83.90 C

ATOM 109 HB2 TYR A 7 6.126 5.640 -6.449 1.00 83.90 H

ATOM 110 HB3 TYR A 7 4.607 6.084 -5.740 1.00 83.90 H

ATOM 111 O TYR A 7 3.760 4.236 -3.314 1.00 83.90 O

ATOM 112 CG TYR A 7 6.257 7.189 -5.003 1.00 83.90 C

ATOM 113 CD1 TYR A 7 5.505 8.052 -4.181 1.00 83.90 C

ATOM 114 HD1 TYR A 7 4.492 7.797 -3.907 1.00 83.90 H

ATOM 115 CD2 TYR A 7 7.580 7.514 -5.350 1.00 83.90 C

ATOM 116 HD2 TYR A 7 8.161 6.845 -5.968 1.00 83.90 H

ATOM 117 CE1 TYR A 7 6.076 9.252 -3.716 1.00 83.90 C

ATOM 118 HE1 TYR A 7 5.503 9.922 -3.091 1.00 83.90 H

ATOM 119 CE2 TYR A 7 8.151 8.712 -4.888 1.00 83.90 C

ATOM 120 HE2 TYR A 7 9.165 8.981 -5.148 1.00 83.90 H

ATOM 121 OH TYR A 7 7.966 10.736 -3.636 1.00 83.90 O

ATOM 122 HH TYR A 7 7.372 11.268 -3.101 1.00 83.90 H

ATOM 123 CZ TYR A 7 7.401 9.583 -4.073 1.00 83.90 C

ATOM 124 N CYS A 8 4.142 3.078 -5.200 1.00 86.40 N

ATOM 125 H CYS A 8 4.796 2.918 -5.953 1.00 86.40 H

ATOM 126 CA CYS A 8 2.962 2.219 -5.120 1.00 86.40 C

ATOM 127 HA CYS A 8 2.058 2.828 -5.132 1.00 86.40 H

ATOM 128 C CYS A 8 2.954 1.384 -3.832 1.00 86.40 C

ATOM 129 CB CYS A 8 2.984 1.303 -6.352 1.00 86.40 C

ATOM 130 HB2 CYS A 8 2.875 0.264 -6.043 1.00 86.40 H

ATOM 131 HB3 CYS A 8 3.949 1.380 -6.853 1.00 86.40 H

ATOM 132 O CYS A 8 1.935 1.279 -3.143 1.00 86.40 O

ATOM 133 SG CYS A 8 1.746 1.636 -7.605 1.00 86.40 S

ATOM 134 N TRP A 9 4.103 0.803 -3.492 1.00 86.99 N

ATOM 135 H TRP A 9 4.903 0.934 -4.095 1.00 86.99 H

ATOM 136 CA TRP A 9 4.289 0.047 -2.256 1.00 86.99 C

ATOM 137 HA TRP A 9 3.507 -0.706 -2.165 1.00 86.99 H

ATOM 138 C TRP A 9 4.181 0.923 -1.016 1.00 86.99 C

ATOM 139 CB TRP A 9 5.645 -0.655 -2.301 1.00 86.99 C

ATOM 140 HB2 TRP A 9 6.335 -0.119 -2.952 1.00 86.99 H

ATOM 141 HB3 TRP A 9 6.079 -0.639 -1.301 1.00 86.99 H

ATOM 142 O TRP A 9 3.452 0.568 -0.088 1.00 86.99 O

ATOM 143 CG TRP A 9 5.595 -2.084 -2.721 1.00 86.99 C

ATOM 144 CD1 TRP A 9 4.719 -2.653 -3.582 1.00 86.99 C

ATOM 145 HD1 TRP A 9 3.940 -2.127 -4.114 1.00 86.99 H

ATOM 146 CD2 TRP A 9 6.491 -3.147 -2.293 1.00 86.99 C

ATOM 147 CE2 TRP A 9 6.077 -4.360 -2.917 1.00 86.99 C

ATOM 148 CE3 TRP A 9 7.622 -3.198 -1.452 1.00 86.99 C

ATOM 149 HE3 TRP A 9 7.976 -2.295 -0.977 1.00 86.99 H

ATOM 150 NE1 TRP A 9 4.993 -4.003 -3.689 1.00 86.99 N

ATOM 151 HE1 TRP A 9 4.516 -4.629 -4.322 1.00 86.99 H

ATOM 152 CH2 TRP A 9 7.873 -5.592 -1.869 1.00 86.99 C

ATOM 153 HH2 TRP A 9 8.415 -6.512 -1.711 1.00 86.99 H

ATOM 154 CZ2 TRP A 9 6.749 -5.572 -2.711 1.00 86.99 C

ATOM 155 HZ2 TRP A 9 6.424 -6.474 -3.208 1.00 86.99 H

ATOM 156 CZ3 TRP A 9 8.308 -4.409 -1.244 1.00 86.99 C

ATOM 157 HZ3 TRP A 9 9.182 -4.432 -0.610 1.00 86.99 H

ATOM 158 N LEU A 10 4.842 2.082 -1.023 1.00 88.20 N

ATOM 159 H LEU A 10 5.450 2.287 -1.803 1.00 88.20 H

ATOM 160 CA LEU A 10 4.778 3.046 0.072 1.00 88.20 C

ATOM 161 HA LEU A 10 5.202 2.585 0.964 1.00 88.20 H

ATOM 162 C LEU A 10 3.323 3.421 0.364 1.00 88.20 C

ATOM 163 CB LEU A 10 5.593 4.296 -0.315 1.00 88.20 C

ATOM 164 HB2 LEU A 10 6.344 4.050 -1.066 1.00 88.20 H

ATOM 165 HB3 LEU A 10 4.933 5.038 -0.764 1.00 88.20 H

ATOM 166 O LEU A 10 2.842 3.335 1.487 1.00 88.20 O

ATOM 167 CG LEU A 10 6.307 4.906 0.899 1.00 88.20 C

ATOM 168 HG LEU A 10 5.703 4.761 1.794 1.00 88.20 H

ATOM 169 CD1 LEU A 10 7.669 4.236 1.090 1.00 88.20 C

ATOM 170 HD11 LEU A 10 8.303 4.414 0.222 1.00 88.20 H

ATOM 171 HD12 LEU A 10 8.160 4.650 1.972 1.00 88.20 H

ATOM 172 HD13 LEU A 10 7.545 3.162 1.228 1.00 88.20 H

ATOM 173 CD2 LEU A 10 6.516 6.404 0.705 1.00 88.20 C

ATOM 174 HD21 LEU A 10 5.553 6.906 0.611 1.00 88.20 H

ATOM 175 HD22 LEU A 10 7.108 6.588 -0.192 1.00 88.20 H

ATOM 176 HD23 LEU A 10 7.041 6.817 1.566 1.00 88.20 H

ATOM 177 N CYS A 11 2.590 3.742 -0.691 1.00 89.44 N

ATOM 178 H CYS A 11 3.059 3.782 -1.585 1.00 89.44 H

ATOM 179 CA CYS A 11 1.213 4.178 -0.632 1.00 89.44 C

ATOM 180 HA CYS A 11 1.142 5.027 0.048 1.00 89.44 H

ATOM 181 C CYS A 11 0.279 3.067 -0.095 1.00 89.44 C

ATOM 182 CB CYS A 11 0.959 4.684 -2.048 1.00 89.44 C

ATOM 183 HB2 CYS A 11 1.064 3.858 -2.752 1.00 89.44 H

ATOM 184 HB3 CYS A 11 1.750 5.397 -2.285 1.00 89.44 H

ATOM 185 O CYS A 11 -0.517 3.321 0.812 1.00 89.44 O

ATOM 186 SG CYS A 11 -0.560 5.546 -2.385 1.00 89.44 S

ATOM 187 N ARG A 12 0.460 1.799 -0.509 1.00 85.92 N

ATOM 188 H ARG A 12 1.140 1.634 -1.237 1.00 85.92 H

ATOM 189 CA ARG A 12 -0.227 0.641 0.113 1.00 85.92 C

ATOM 190 HA ARG A 12 -1.303 0.814 0.091 1.00 85.92 H

ATOM 191 C ARG A 12 0.136 0.453 1.587 1.00 85.92 C

ATOM 192 CB ARG A 12 0.082 -0.666 -0.634 1.00 85.92 C

ATOM 193 HB2 ARG A 12 -0.261 -1.503 -0.026 1.00 85.92 H

ATOM 194 HB3 ARG A 12 1.160 -0.761 -0.762 1.00 85.92 H

ATOM 195 O ARG A 12 -0.746 0.139 2.388 1.00 85.92 O

ATOM 196 CG ARG A 12 -0.609 -0.768 -1.997 1.00 85.92 C

ATOM 197 HG2 ARG A 12 -0.262 0.051 -2.626 1.00 85.92 H

ATOM 198 HG3 ARG A 12 -1.689 -0.678 -1.880 1.00 85.92 H

ATOM 199 CD ARG A 12 -0.279 -2.115 -2.665 1.00 85.92 C

ATOM 200 HD2 ARG A 12 -1.022 -2.854 -2.368 1.00 85.92 H

ATOM 201 HD3 ARG A 12 0.700 -2.456 -2.330 1.00 85.92 H

ATOM 202 NE ARG A 12 -0.224 -1.967 -4.129 1.00 85.92 N

ATOM 203 HE ARG A 12 0.287 -1.166 -4.472 1.00 85.92 H

ATOM 204 NH1 ARG A 12 -1.541 -3.743 -4.835 1.00 85.92 N

ATOM 205 HH11 ARG A 12 -1.683 -4.091 -3.897 1.00 85.92 H

ATOM 206 HH12 ARG A 12 -2.013 -4.152 -5.629 1.00 85.92 H

ATOM 207 NH2 ARG A 12 -0.695 -2.324 -6.301 1.00 85.92 N

ATOM 208 HH21 ARG A 12 -1.409 -2.608 -6.956 1.00 85.92 H

ATOM 209 HH22 ARG A 12 -0.202 -1.483 -6.565 1.00 85.92 H

ATOM 210 CZ ARG A 12 -0.817 -2.687 -5.066 1.00 85.92 C

ATOM 211 N ALA A 13 1.405 0.628 1.953 1.00 88.78 N

ATOM 212 H ALA A 13 2.088 0.858 1.246 1.00 88.78 H

ATOM 213 CA ALA A 13 1.858 0.502 3.336 1.00 88.78 C

ATOM 214 HA ALA A 13 1.539 -0.468 3.718 1.00 88.78 H

ATOM 215 C ALA A 13 1.226 1.579 4.231 1.00 88.78 C

ATOM 216 CB ALA A 13 3.390 0.542 3.374 1.00 88.78 C

ATOM 217 HB1 ALA A 13 3.736 0.375 4.394 1.00 88.78 H

ATOM 218 HB2 ALA A 13 3.799 -0.238 2.731 1.00 88.78 H

ATOM 219 HB3 ALA A 13 3.758 1.510 3.033 1.00 88.78 H

ATOM 220 O ALA A 13 0.730 1.256 5.312 1.00 88.78 O

ATOM 221 N LEU A 14 1.151 2.827 3.755 1.00 87.88 N

ATOM 222 H LEU A 14 1.613 3.034 2.881 1.00 87.88 H

ATOM 223 CA LEU A 14 0.446 3.911 4.442 1.00 87.88 C

ATOM 224 HA LEU A 14 0.856 4.018 5.446 1.00 87.88 H

ATOM 225 C LEU A 14 -1.040 3.584 4.611 1.00 87.88 C

ATOM 226 CB LEU A 14 0.616 5.247 3.692 1.00 87.88 C

ATOM 227 HB2 LEU A 14 0.627 5.086 2.614 1.00 87.88 H

ATOM 228 HB3 LEU A 14 -0.261 5.855 3.916 1.00 87.88 H

ATOM 229 O LEU A 14 -1.560 3.678 5.719 1.00 87.88 O

ATOM 230 CG LEU A 14 1.852 6.058 4.122 1.00 87.88 C

ATOM 231 HG LEU A 14 1.978 5.977 5.201 1.00 87.88 H

ATOM 232 CD1 LEU A 14 3.140 5.608 3.439 1.00 87.88 C

ATOM 233 HD11 LEU A 14 3.347 4.566 3.684 1.00 87.88 H

ATOM 234 HD12 LEU A 14 3.053 5.730 2.359 1.00 87.88 H

ATOM 235 HD13 LEU A 14 3.974 6.214 3.793 1.00 87.88 H

ATOM 236 CD2 LEU A 14 1.643 7.533 3.785 1.00 87.88 C

ATOM 237 HD21 LEU A 14 2.513 8.111 4.097 1.00 87.88 H

ATOM 238 HD22 LEU A 14 0.772 7.918 4.315 1.00 87.88 H

ATOM 239 HD23 LEU A 14 1.497 7.659 2.713 1.00 87.88 H

ATOM 240 N ILE A 15 -1.713 3.124 3.552 1.00 86.64 N

ATOM 241 H ILE A 15 -1.242 3.111 2.659 1.00 86.64 H

ATOM 242 CA ILE A 15 -3.126 2.724 3.616 1.00 86.64 C

ATOM 243 HA ILE A 15 -3.714 3.579 3.949 1.00 86.64 H

ATOM 244 C ILE A 15 -3.334 1.605 4.648 1.00 86.64 C

ATOM 245 CB ILE A 15 -3.625 2.306 2.213 1.00 86.64 C

ATOM 246 HB ILE A 15 -2.947 1.550 1.817 1.00 86.64 H

ATOM 247 O ILE A 15 -4.309 1.635 5.395 1.00 86.64 O

ATOM 248 CG1 ILE A 15 -3.675 3.493 1.235 1.00 86.64 C

ATOM 249 HG12 ILE A 15 -4.599 4.047 1.403 1.00 86.64 H

ATOM 250 HG13 ILE A 15 -2.841 4.171 1.412 1.00 86.64 H

ATOM 251 CG2 ILE A 15 -5.037 1.705 2.274 1.00 86.64 C

ATOM 252 HG21 ILE A 15 -4.979 0.721 2.738 1.00 86.64 H

ATOM 253 HG22 ILE A 15 -5.430 1.543 1.271 1.00 86.64 H

ATOM 254 HG23 ILE A 15 -5.711 2.358 2.830 1.00 86.64 H

ATOM 255 CD1 ILE A 15 -3.636 3.067 -0.237 1.00 86.64 C

ATOM 256 HD11 ILE A 15 -3.612 3.961 -0.860 1.00 86.64 H

ATOM 257 HD12 ILE A 15 -4.513 2.478 -0.502 1.00 86.64 H

ATOM 258 HD13 ILE A 15 -2.739 2.482 -0.438 1.00 86.64 H

ATOM 259 N LYS A 16 -2.437 0.612 4.724 1.00 85.16 N

ATOM 260 H LYS A 16 -1.668 0.621 4.069 1.00 85.16 H

ATOM 261 CA LYS A 16 -2.494 -0.449 5.746 1.00 85.16 C

ATOM 262 HA LYS A 16 -3.495 -0.880 5.773 1.00 85.16 H

ATOM 263 C LYS A 16 -2.261 0.085 7.160 1.00 85.16 C

ATOM 264 CB LYS A 16 -1.496 -1.572 5.430 1.00 85.16 C

ATOM 265 HB2 LYS A 16 -0.566 -1.154 5.045 1.00 85.16 H

ATOM 266 HB3 LYS A 16 -1.268 -2.095 6.359 1.00 85.16 H

ATOM 267 O LYS A 16 -2.976 -0.321 8.070 1.00 85.16 O

ATOM 268 CG LYS A 16 -2.064 -2.599 4.439 1.00 85.16 C

ATOM 269 HG2 LYS A 16 -2.208 -2.133 3.464 1.00 85.16 H

ATOM 270 HG3 LYS A 16 -3.026 -2.954 4.808 1.00 85.16 H

ATOM 271 CD LYS A 16 -1.108 -3.795 4.310 1.00 85.16 C

ATOM 272 HD2 LYS A 16 -0.876 -4.169 5.308 1.00 85.16 H

ATOM 273 HD3 LYS A 16 -0.184 -3.465 3.836 1.00 85.16 H

ATOM 274 CE LYS A 16 -1.740 -4.931 3.494 1.00 85.16 C

ATOM 275 HE2 LYS A 16 -2.701 -5.185 3.941 1.00 85.16 H

ATOM 276 HE3 LYS A 16 -1.927 -4.574 2.481 1.00 85.16 H

ATOM 277 NZ LYS A 16 -0.870 -6.138 3.462 1.00 85.16 N

ATOM 278 HZ1 LYS A 16 0.028 -5.935 3.045 1.00 85.16 H

ATOM 279 HZ2 LYS A 16 -0.707 -6.488 4.395 1.00 85.16 H

ATOM 280 HZ3 LYS A 16 -1.301 -6.882 2.932 1.00 85.16 H

ATOM 281 N ARG A 17 -1.319 1.012 7.352 1.00 86.79 N

ATOM 282 H ARG A 17 -0.748 1.286 6.565 1.00 86.79 H

ATOM 283 CA ARG A 17 -1.093 1.650 8.659 1.00 86.79 C

ATOM 284 HA ARG A 17 -0.966 0.882 9.422 1.00 86.79 H

ATOM 285 C ARG A 17 -2.298 2.463 9.116 1.00 86.79 C

ATOM 286 CB ARG A 17 0.151 2.544 8.631 1.00 86.79 C

ATOM 287 HB2 ARG A 17 0.074 3.241 9.465 1.00 86.79 H

ATOM 288 HB3 ARG A 17 0.185 3.127 7.711 1.00 86.79 H

ATOM 289 O ARG A 17 -2.737 2.307 10.247 1.00 86.79 O

ATOM 290 CG ARG A 17 1.449 1.747 8.797 1.00 86.79 C

ATOM 291 HG2 ARG A 17 1.702 1.243 7.864 1.00 86.79 H

ATOM 292 HG3 ARG A 17 1.327 1.006 9.587 1.00 86.79 H

ATOM 293 CD ARG A 17 2.564 2.716 9.202 1.00 86.79 C

ATOM 294 HD2 ARG A 17 2.695 3.456 8.413 1.00 86.79 H

ATOM 295 HD3 ARG A 17 2.258 3.229 10.114 1.00 86.79 H

ATOM 296 NE ARG A 17 3.844 2.025 9.440 1.00 86.79 N

ATOM 297 HE ARG A 17 3.938 1.086 9.080 1.00 86.79 H

ATOM 298 NH1 ARG A 17 4.879 3.727 10.595 1.00 86.79 N

ATOM 299 HH11 ARG A 17 4.038 4.285 10.543 1.00 86.79 H

ATOM 300 HH12 ARG A 17 5.690 4.088 11.076 1.00 86.79 H

ATOM 301 NH2 ARG A 17 5.989 1.838 10.189 1.00 86.79 N

ATOM 302 HH21 ARG A 17 6.053 0.898 9.825 1.00 86.79 H

ATOM 303 HH22 ARG A 17 6.779 2.226 10.684 1.00 86.79 H

ATOM 304 CZ ARG A 17 4.892 2.532 10.071 1.00 86.79 C

ATOM 305 N ILE A 18 -2.868 3.272 8.227 1.00 84.63 N

ATOM 306 H ILE A 18 -2.427 3.379 7.324 1.00 84.63 H

ATOM 307 CA ILE A 18 -4.063 4.069 8.526 1.00 84.63 C

ATOM 308 HA ILE A 18 -3.883 4.649 9.431 1.00 84.63 H

ATOM 309 C ILE A 18 -5.248 3.147 8.813 1.00 84.63 C

ATOM 310 CB ILE A 18 -4.367 5.053 7.378 1.00 84.63 C

ATOM 311 HB ILE A 18 -4.455 4.484 6.453 1.00 84.63 H

ATOM 312 O ILE A 18 -6.002 3.410 9.735 1.00 84.63 O

ATOM 313 CG1 ILE A 18 -3.224 6.083 7.228 1.00 84.63 C

ATOM 314 HG12 ILE A 18 -2.255 5.586 7.282 1.00 84.63 H

ATOM 315 HG13 ILE A 18 -3.265 6.798 8.050 1.00 84.63 H

ATOM 316 CG2 ILE A 18 -5.698 5.791 7.628 1.00 84.63 C

ATOM 317 HG21 ILE A 18 -5.897 6.508 6.833 1.00 84.63 H

ATOM 318 HG22 ILE A 18 -6.536 5.094 7.650 1.00 84.63 H

ATOM 319 HG23 ILE A 18 -5.659 6.323 8.579 1.00 84.63 H

ATOM 320 CD1 ILE A 18 -3.268 6.843 5.896 1.00 84.63 C

ATOM 321 HD11 ILE A 18 -4.143 7.491 5.849 1.00 84.63 H

ATOM 322 HD12 ILE A 18 -2.375 7.461 5.808 1.00 84.63 H

ATOM 323 HD13 ILE A 18 -3.291 6.138 5.066 1.00 84.63 H

ATOM 324 N GLN A 19 -5.390 2.035 8.085 1.00 80.59 N

ATOM 325 H GLN A 19 -4.766 1.884 7.305 1.00 80.59 H

ATOM 326 CA GLN A 19 -6.398 1.014 8.389 1.00 80.59 C

ATOM 327 HA GLN A 19 -7.389 1.469 8.366 1.00 80.59 H

ATOM 328 C GLN A 19 -6.234 0.389 9.771 1.00 80.59 C

ATOM 329 CB GLN A 19 -6.314 -0.118 7.365 1.00 80.59 C

ATOM 330 HB2 GLN A 19 -5.275 -0.326 7.112 1.00 80.59 H

ATOM 331 HB3 GLN A 19 -6.747 -1.025 7.787 1.00 80.59 H

ATOM 332 O GLN A 19 -7.239 0.102 10.415 1.00 80.59 O

ATOM 333 CG GLN A 19 -7.096 0.220 6.111 1.00 80.59 C

ATOM 334 HG2 GLN A 19 -8.143 0.274 6.408 1.00 80.59 H

ATOM 335 HG3 GLN A 19 -6.786 1.185 5.709 1.00 80.59 H

ATOM 336 CD GLN A 19 -6.902 -0.861 5.064 1.00 80.59 C

ATOM 337 NE2 GLN A 19 -5.804 -0.855 4.364 1.00 80.59 N

ATOM 338 HE21 GLN A 19 -5.701 -1.584 3.672 1.00 80.59 H

ATOM 339 HE22 GLN A 19 -5.126 -0.130 4.545 1.00 80.59 H

ATOM 340 OE1 GLN A 19 -7.705 -1.747 4.863 1.00 80.59 O

ATOM 341 N ALA A 20 -4.995 0.152 10.202 1.00 83.06 N

ATOM 342 H ALA A 20 -4.210 0.347 9.597 1.00 83.06 H

ATOM 343 CA ALA A 20 -4.719 -0.356 11.538 1.00 83.06 C

ATOM 344 HA ALA A 20 -5.354 -1.222 11.727 1.00 83.06 H

ATOM 345 C ALA A 20 -5.049 0.689 12.617 1.00 83.06 C

ATOM 346 CB ALA A 20 -3.256 -0.811 11.598 1.00 83.06 C

ATOM 347 HB1 ALA A 20 -3.060 -1.258 12.572 1.00 83.06 H

ATOM 348 HB2 ALA A 20 -3.067 -1.554 10.823 1.00 83.06 H

ATOM 349 HB3 ALA A 20 -2.587 0.038 11.461 1.00 83.06 H

ATOM 350 O ALA A 20 -5.551 0.329 13.675 1.00 83.06 O

ATOM 351 N MET A 21 -4.815 1.975 12.339 1.00 82.77 N

ATOM 352 H MET A 21 -4.340 2.198 11.477 1.00 82.77 H

ATOM 353 CA MET A 21 -5.138 3.071 13.261 1.00 82.77 C

ATOM 354 HA MET A 21 -4.895 2.755 14.276 1.00 82.77 H

ATOM 355 C MET A 21 -6.630 3.424 13.296 1.00 82.77 C

ATOM 356 CB MET A 21 -4.281 4.300 12.911 1.00 82.77 C

ATOM 357 HB2 MET A 21 -3.961 4.257 11.869 1.00 82.77 H

ATOM 358 HB3 MET A 21 -4.851 5.217 13.057 1.00 82.77 H

ATOM 359 O MET A 21 -7.167 3.701 14.361 1.00 82.77 O

ATOM 360 CG MET A 21 -3.063 4.356 13.833 1.00 82.77 C

ATOM 361 HG2 MET A 21 -2.650 3.352 13.927 1.00 82.77 H

ATOM 362 HG3 MET A 21 -3.397 4.676 14.820 1.00 82.77 H

ATOM 363 SD MET A 21 -1.725 5.445 13.286 1.00 82.77 S

ATOM 364 CE MET A 21 -0.688 5.346 14.770 1.00 82.77 C

ATOM 365 HE1 MET A 21 0.201 5.963 14.636 1.00 82.77 H

ATOM 366 HE2 MET A 21 -0.390 4.313 14.947 1.00 82.77 H

ATOM 367 HE3 MET A 21 -1.246 5.713 15.632 1.00 82.77 H

ATOM 368 N ILE A 22 -7.310 3.423 12.148 1.00 75.91 N

ATOM 369 H ILE A 22 -6.797 3.203 11.306 1.00 75.91 H

ATOM 370 CA ILE A 22 -8.709 3.836 11.996 1.00 75.91 C

ATOM 371 HA ILE A 22 -9.177 3.845 12.981 1.00 75.91 H

ATOM 372 C ILE A 22 -9.425 2.799 11.112 1.00 75.91 C

ATOM 373 CB ILE A 22 -8.832 5.281 11.453 1.00 75.91 C

ATOM 374 HB ILE A 22 -8.509 5.295 10.412 1.00 75.91 H

ATOM 375 O ILE A 22 -9.533 2.965 9.891 1.00 75.91 O

ATOM 376 CG1 ILE A 22 -7.943 6.274 12.241 1.00 75.91 C

ATOM 377 HG12 ILE A 22 -8.250 6.294 13.286 1.00 75.91 H

ATOM 378 HG13 ILE A 22 -6.905 5.943 12.195 1.00 75.91 H

ATOM 379 CG2 ILE A 22 -10.313 5.710 11.527 1.00 75.91 C

ATOM 380 HG21 ILE A 22 -10.952 5.008 10.992 1.00 75.91 H

ATOM 381 HG22 ILE A 22 -10.446 6.687 11.063 1.00 75.91 H

ATOM 382 HG23 ILE A 22 -10.635 5.770 12.566 1.00 75.91 H

ATOM 383 CD1 ILE A 22 -7.945 7.703 11.688 1.00 75.91 C

ATOM 384 HD11 ILE A 22 -8.918 8.171 11.838 1.00 75.91 H

ATOM 385 HD12 ILE A 22 -7.196 8.293 12.216 1.00 75.91 H

ATOM 386 HD13 ILE A 22 -7.702 7.692 10.626 1.00 75.91 H

ATOM 387 N PRO A 23 -9.925 1.698 11.703 1.00 72.14 N

ATOM 388 CA PRO A 23 -10.511 0.596 10.942 1.00 72.14 C

ATOM 389 HA PRO A 23 -9.810 0.289 10.165 1.00 72.14 H

ATOM 390 C PRO A 23 -11.837 0.959 10.258 1.00 72.14 C

ATOM 391 CB PRO A 23 -10.661 -0.551 11.950 1.00 72.14 C

ATOM 392 HB2 PRO A 23 -9.774 -1.184 11.905 1.00 72.14 H

ATOM 393 HB3 PRO A 23 -11.555 -1.148 11.769 1.00 72.14 H

ATOM 394 O PRO A 23 -12.173 0.366 9.230 1.00 72.14 O

ATOM 395 CG PRO A 23 -10.712 0.143 13.310 1.00 72.14 C

ATOM 396 HG2 PRO A 23 -10.351 -0.502 14.111 1.00 72.14 H

ATOM 397 HG3 PRO A 23 -11.730 0.476 13.514 1.00 72.14 H

ATOM 398 CD PRO A 23 -9.809 1.353 13.113 1.00 72.14 C

ATOM 399 HD2 PRO A 23 -8.776 1.084 13.336 1.00 72.14 H

ATOM 400 HD3 PRO A 23 -10.136 2.163 13.765 1.00 72.14 H

ATOM 401 N LYS A 24 -12.583 1.940 10.784 1.00 69.19 N

ATOM 402 H LYS A 24 -12.208 2.430 11.585 1.00 69.19 H

ATOM 403 CA LYS A 24 -13.926 2.310 10.316 1.00 69.19 C

ATOM 404 HA LYS A 24 -14.307 1.535 9.651 1.00 69.19 H

ATOM 405 C LYS A 24 -13.892 3.608 9.499 1.00 69.19 C

ATOM 406 CB LYS A 24 -14.891 2.386 11.519 1.00 69.19 C

ATOM 407 HB2 LYS A 24 -15.834 2.815 11.180 1.00 69.19 H

ATOM 408 HB3 LYS A 24 -14.474 3.049 12.278 1.00 69.19 H

ATOM 409 O LYS A 24 -14.002 4.694 10.047 1.00 69.19 O

ATOM 410 CG LYS A 24 -15.181 1.006 12.139 1.00 69.19 C

ATOM 411 HG2 LYS A 24 -14.250 0.562 12.492 1.00 69.19 H

ATOM 412 HG3 LYS A 24 -15.609 0.357 11.375 1.00 69.19 H

ATOM 413 CD LYS A 24 -16.162 1.107 13.319 1.00 69.19 C

ATOM 414 HD2 LYS A 24 -17.089 1.572 12.985 1.00 69.19 H

ATOM 415 HD3 LYS A 24 -15.712 1.736 14.087 1.00 69.19 H

ATOM 416 CE LYS A 24 -16.462 -0.284 13.900 1.00 69.19 C

ATOM 417 HE2 LYS A 24 -17.058 -0.844 13.179 1.00 69.19 H

ATOM 418 HE3 LYS A 24 -15.518 -0.813 14.033 1.00 69.19 H

ATOM 419 NZ LYS A 24 -17.170 -0.203 15.205 1.00 69.19 N

ATOM 420 HZ1 LYS A 24 -16.619 0.295 15.889 1.00 69.19 H

ATOM 421 HZ2 LYS A 24 -18.054 0.275 15.106 1.00 69.19 H

ATOM 422 HZ3 LYS A 24 -17.361 -1.126 15.570 1.00 69.19 H

ATOM 423 N GLY A 25 -13.733 3.493 8.178 1.00 65.80 N

ATOM 424 H GLY A 25 -13.602 2.557 7.820 1.00 65.80 H

ATOM 425 CA GLY A 25 -14.109 4.554 7.223 1.00 65.80 C

ATOM 426 HA2 GLY A 25 -14.706 5.305 7.740 1.00 65.80 H

ATOM 427 HA3 GLY A 25 -14.750 4.118 6.457 1.00 65.80 H

ATOM 428 C GLY A 25 -12.978 5.304 6.507 1.00 65.80 C

ATOM 429 O GLY A 25 -13.196 5.819 5.416 1.00 65.80 O

ATOM 430 N GLY A 26 -11.743 5.299 7.018 1.00 72.23 N

ATOM 431 H GLY A 26 -11.610 4.896 7.934 1.00 72.23 H

ATOM 432 CA GLY A 26 -10.648 6.109 6.451 1.00 72.23 C

ATOM 433 HA2 GLY A 26 -11.019 7.114 6.253 1.00 72.23 H

ATOM 434 HA3 GLY A 26 -9.858 6.192 7.198 1.00 72.23 H

ATOM 435 C GLY A 26 -10.009 5.585 5.158 1.00 72.23 C

ATOM 436 O GLY A 26 -9.053 6.177 4.667 1.00 72.23 O

ATOM 437 N ARG A 27 -10.471 4.459 4.596 1.00 71.20 N

ATOM 438 H ARG A 27 -11.299 4.047 5.001 1.00 71.20 H

ATOM 439 CA ARG A 27 -9.758 3.780 3.496 1.00 71.20 C

ATOM 440 HA ARG A 27 -8.689 3.874 3.686 1.00 71.20 H

ATOM 441 C ARG A 27 -9.994 4.415 2.130 1.00 71.20 C

ATOM 442 CB ARG A 27 -10.100 2.283 3.433 1.00 71.20 C

ATOM 443 HB2 ARG A 27 -9.315 1.800 2.851 1.00 71.20 H

ATOM 444 HB3 ARG A 27 -11.059 2.148 2.934 1.00 71.20 H

ATOM 445 O ARG A 27 -9.173 4.217 1.239 1.00 71.20 O

ATOM 446 CG ARG A 27 -10.139 1.559 4.775 1.00 71.20 C

ATOM 447 HG2 ARG A 27 -10.959 1.945 5.380 1.00 71.20 H

ATOM 448 HG3 ARG A 27 -9.200 1.751 5.295 1.00 71.20 H

ATOM 449 CD ARG A 27 -10.357 0.054 4.552 1.00 71.20 C

ATOM 450 HD2 ARG A 27 -9.564 -0.327 3.908 1.00 71.20 H

ATOM 451 HD3 ARG A 27 -11.312 -0.095 4.048 1.00 71.20 H

ATOM 452 NE ARG A 27 -10.368 -0.688 5.827 1.00 71.20 N

ATOM 453 HE ARG A 27 -10.394 -0.148 6.680 1.00 71.20 H

ATOM 454 NH1 ARG A 27 -10.357 -2.829 4.972 1.00 71.20 N

ATOM 455 HH11 ARG A 27 -10.353 -2.445 4.038 1.00 71.20 H

ATOM 456 HH12 ARG A 27 -10.225 -3.820 5.115 1.00 71.20 H

ATOM 457 NH2 ARG A 27 -10.131 -2.507 7.170 1.00 71.20 N

ATOM 458 HH21 ARG A 27 -10.131 -1.907 7.982 1.00 71.20 H

ATOM 459 HH22 ARG A 27 -10.063 -3.508 7.288 1.00 71.20 H

ATOM 460 CZ ARG A 27 -10.286 -2.002 5.978 1.00 71.20 C

ATOM 461 N MET A 28 -11.122 5.096 1.933 1.00 78.22 N

ATOM 462 H MET A 28 -11.743 5.247 2.715 1.00 78.22 H

ATOM 463 CA MET A 28 -11.566 5.487 0.592 1.00 78.22 C

ATOM 464 HA MET A 28 -11.450 4.624 -0.063 1.00 78.22 H

ATOM 465 C MET A 28 -10.694 6.604 0.018 1.00 78.22 C

ATOM 466 CB MET A 28 -13.058 5.846 0.634 1.00 78.22 C

ATOM 467 HB2 MET A 28 -13.554 5.183 1.344 1.00 78.22 H

ATOM 468 HB3 MET A 28 -13.198 6.872 0.975 1.00 78.22 H

ATOM 469 O MET A 28 -10.251 6.498 -1.121 1.00 78.22 O

ATOM 470 CG MET A 28 -13.739 5.647 -0.723 1.00 78.22 C

ATOM 471 HG2 MET A 28 -13.421 4.694 -1.145 1.00 78.22 H

ATOM 472 HG3 MET A 28 -13.444 6.447 -1.403 1.00 78.22 H

ATOM 473 SD MET A 28 -15.542 5.613 -0.572 1.00 78.22 S

ATOM 474 CE MET A 28 -16.016 5.064 -2.233 1.00 78.22 C

ATOM 475 HE1 MET A 28 -15.592 4.080 -2.435 1.00 78.22 H

ATOM 476 HE2 MET A 28 -15.657 5.779 -2.973 1.00 78.22 H

ATOM 477 HE3 MET A 28 -17.103 5.004 -2.295 1.00 78.22 H

ATOM 478 N LEU A 29 -10.344 7.596 0.841 1.00 84.39 N

ATOM 479 H LEU A 29 -10.735 7.613 1.772 1.00 84.39 H

ATOM 480 CA LEU A 29 -9.510 8.723 0.435 1.00 84.39 C

ATOM 481 HA LEU A 29 -9.961 9.141 -0.465 1.00 84.39 H

ATOM 482 C LEU A 29 -8.075 8.310 0.046 1.00 84.39 C

ATOM 483 CB LEU A 29 -9.558 9.805 1.532 1.00 84.39 C

ATOM 484 HB2 LEU A 29 -9.039 9.452 2.423 1.00 84.39 H

ATOM 485 HB3 LEU A 29 -10.595 9.980 1.818 1.00 84.39 H

ATOM 486 O LEU A 29 -7.685 8.568 -1.092 1.00 84.39 O

ATOM 487 CG LEU A 29 -8.945 11.145 1.089 1.00 84.39 C

ATOM 488 HG LEU A 29 -7.995 10.971 0.585 1.00 84.39 H

ATOM 489 CD1 LEU A 29 -9.893 11.888 0.146 1.00 84.39 C

ATOM 490 HD11 LEU A 29 -10.853 12.065 0.631 1.00 84.39 H

ATOM 491 HD12 LEU A 29 -9.457 12.850 -0.124 1.00 84.39 H

ATOM 492 HD13 LEU A 29 -10.047 11.319 -0.771 1.00 84.39 H

ATOM 493 CD2 LEU A 29 -8.680 12.024 2.309 1.00 84.39 C

ATOM 494 HD21 LEU A 29 -8.247 12.972 1.990 1.00 84.39 H

ATOM 495 HD22 LEU A 29 -7.976 11.532 2.981 1.00 84.39 H

ATOM 496 HD23 LEU A 29 -9.610 12.224 2.841 1.00 84.39 H

ATOM 497 N PRO A 30 -7.282 7.626 0.898 1.00 83.39 N

ATOM 498 CA PRO A 30 -5.903 7.306 0.550 1.00 83.39 C

ATOM 499 HA PRO A 30 -5.395 8.207 0.205 1.00 83.39 H

ATOM 500 C PRO A 30 -5.818 6.263 -0.575 1.00 83.39 C

ATOM 501 CB PRO A 30 -5.257 6.855 1.861 1.00 83.39 C

ATOM 502 HB2 PRO A 30 -4.900 7.732 2.401 1.00 83.39 H

ATOM 503 HB3 PRO A 30 -4.424 6.169 1.706 1.00 83.39 H

ATOM 504 O PRO A 30 -4.925 6.356 -1.411 1.00 83.39 O

ATOM 505 CG PRO A 30 -6.421 6.236 2.632 1.00 83.39 C

ATOM 506 HG2 PRO A 30 -6.242 6.234 3.707 1.00 83.39 H

ATOM 507 HG3 PRO A 30 -6.622 5.228 2.269 1.00 83.39 H

ATOM 508 CD PRO A 30 -7.568 7.168 2.250 1.00 83.39 C

ATOM 509 HD2 PRO A 30 -7.571 8.014 2.937 1.00 83.39 H

ATOM 510 HD3 PRO A 30 -8.516 6.633 2.300 1.00 83.39 H

ATOM 511 N GLN A 31 -6.762 5.314 -0.671 1.00 84.05 N

ATOM 512 H GLN A 31 -7.507 5.292 0.011 1.00 84.05 H

ATOM 513 CA GLN A 31 -6.827 4.400 -1.820 1.00 84.05 C

ATOM 514 HA GLN A 31 -5.857 3.923 -1.962 1.00 84.05 H

ATOM 515 C GLN A 31 -7.165 5.124 -3.123 1.00 84.05 C

ATOM 516 CB GLN A 31 -7.888 3.319 -1.610 1.00 84.05 C

ATOM 517 HB2 GLN A 31 -8.074 2.836 -2.569 1.00 84.05 H

ATOM 518 HB3 GLN A 31 -8.820 3.782 -1.287 1.00 84.05 H

ATOM 519 O GLN A 31 -6.574 4.813 -4.156 1.00 84.05 O

ATOM 520 CG GLN A 31 -7.469 2.224 -0.632 1.00 84.05 C

ATOM 521 HG2 GLN A 31 -6.598 1.698 -1.023 1.00 84.05 H

ATOM 522 HG3 GLN A 31 -7.226 2.661 0.337 1.00 84.05 H

ATOM 523 CD GLN A 31 -8.610 1.236 -0.480 1.00 84.05 C

ATOM 524 NE2 GLN A 31 -9.526 1.489 0.414 1.00 84.05 N

ATOM 525 HE21 GLN A 31 -9.487 2.389 0.871 1.00 84.05 H

ATOM 526 HE22 GLN A 31 -10.337 0.886 0.407 1.00 84.05 H

ATOM 527 OE1 GLN A 31 -8.758 0.283 -1.222 1.00 84.05 O

ATOM 528 N LEU A 32 -8.111 6.069 -3.088 1.00 84.87 N

ATOM 529 H LEU A 32 -8.586 6.274 -2.221 1.00 84.87 H

ATOM 530 CA LEU A 32 -8.466 6.872 -4.253 1.00 84.87 C

ATOM 531 HA LEU A 32 -8.748 6.201 -5.065 1.00 84.87 H

ATOM 532 C LEU A 32 -7.258 7.680 -4.722 1.00 84.87 C

ATOM 533 CB LEU A 32 -9.665 7.772 -3.909 1.00 84.87 C

ATOM 534 HB2 LEU A 32 -10.494 7.146 -3.579 1.00 84.87 H

ATOM 535 HB3 LEU A 32 -9.387 8.428 -3.084 1.00 84.87 H

ATOM 536 O LEU A 32 -6.915 7.608 -5.893 1.00 84.87 O

ATOM 537 CG LEU A 32 -10.159 8.642 -5.078 1.00 84.87 C

ATOM 538 HG LEU A 32 -9.331 9.219 -5.490 1.00 84.87 H

ATOM 539 CD1 LEU A 32 -10.778 7.788 -6.187 1.00 84.87 C

ATOM 540 HD11 LEU A 32 -11.182 8.441 -6.961 1.00 84.87 H

ATOM 541 HD12 LEU A 32 -10.018 7.155 -6.646 1.00 84.87 H

ATOM 542 HD13 LEU A 32 -11.587 7.175 -5.788 1.00 84.87 H

ATOM 543 CD2 LEU A 32 -11.213 9.620 -4.566 1.00 84.87 C

ATOM 544 HD21 LEU A 32 -11.555 10.254 -5.384 1.00 84.87 H

ATOM 545 HD22 LEU A 32 -12.066 9.080 -4.155 1.00 84.87 H

ATOM 546 HD23 LEU A 32 -10.785 10.259 -3.793 1.00 84.87 H

ATOM 547 N VAL A 33 -6.554 8.351 -3.807 1.00 85.89 N

ATOM 548 H VAL A 33 -6.912 8.383 -2.863 1.00 85.89 H

ATOM 549 CA VAL A 33 -5.321 9.095 -4.111 1.00 85.89 C

ATOM 550 HA VAL A 33 -5.545 9.861 -4.853 1.00 85.89 H

ATOM 551 C VAL A 33 -4.259 8.176 -4.723 1.00 85.89 C

ATOM 552 CB VAL A 33 -4.805 9.794 -2.838 1.00 85.89 C

ATOM 553 HB VAL A 33 -4.738 9.062 -2.033 1.00 85.89 H

ATOM 554 O VAL A 33 -3.671 8.511 -5.748 1.00 85.89 O

ATOM 555 CG1 VAL A 33 -3.425 10.433 -3.027 1.00 85.89 C

ATOM 556 HG11 VAL A 33 -3.152 10.990 -2.131 1.00 85.89 H

ATOM 557 HG12 VAL A 33 -3.439 11.116 -3.877 1.00 85.89 H

ATOM 558 HG13 VAL A 33 -2.667 9.667 -3.190 1.00 85.89 H

ATOM 559 CG2 VAL A 33 -5.768 10.909 -2.409 1.00 85.89 C

ATOM 560 HG21 VAL A 33 -5.754 11.715 -3.143 1.00 85.89 H

ATOM 561 HG22 VAL A 33 -5.465 11.308 -1.441 1.00 85.89 H

ATOM 562 HG23 VAL A 33 -6.791 10.543 -2.327 1.00 85.89 H

ATOM 563 N CYS A 34 -4.065 6.978 -4.166 1.00 87.75 N

ATOM 564 H CYS A 34 -4.550 6.766 -3.306 1.00 87.75 H

ATOM 565 CA CYS A 34 -3.109 6.003 -4.691 1.00 87.75 C

ATOM 566 HA CYS A 34 -2.125 6.471 -4.709 1.00 87.75 H

ATOM 567 C CYS A 34 -3.437 5.534 -6.120 1.00 87.75 C

ATOM 568 CB CYS A 34 -3.080 4.804 -3.741 1.00 87.75 C

ATOM 569 HB2 CYS A 34 -3.412 5.116 -2.751 1.00 87.75 H

ATOM 570 HB3 CYS A 34 -3.759 4.030 -4.099 1.00 87.75 H

ATOM 571 O CYS A 34 -2.546 5.333 -6.945 1.00 87.75 O

ATOM 572 SG CYS A 34 -1.438 4.117 -3.533 1.00 87.75 S

ATOM 573 N ARG A 35 -4.726 5.377 -6.436 1.00 85.49 N

ATOM 574 H ARG A 35 -5.415 5.528 -5.714 1.00 85.49 H

ATOM 575 CA ARG A 35 -5.189 5.031 -7.785 1.00 85.49 C

ATOM 576 HA ARG A 35 -4.511 4.299 -8.224 1.00 85.49 H

ATOM 577 C ARG A 35 -5.174 6.217 -8.746 1.00 85.49 C

ATOM 578 CB ARG A 35 -6.595 4.416 -7.711 1.00 85.49 C

ATOM 579 HB2 ARG A 35 -7.123 4.632 -8.640 1.00 85.49 H

ATOM 580 HB3 ARG A 35 -7.163 4.852 -6.889 1.00 85.49 H

ATOM 581 O ARG A 35 -4.866 6.027 -9.913 1.00 85.49 O

ATOM 582 CG ARG A 35 -6.514 2.892 -7.565 1.00 85.49 C

ATOM 583 HG2 ARG A 35 -6.017 2.620 -6.633 1.00 85.49 H

ATOM 584 HG3 ARG A 35 -5.930 2.513 -8.403 1.00 85.49 H

ATOM 585 CD ARG A 35 -7.892 2.231 -7.653 1.00 85.49 C

ATOM 586 HD2 ARG A 35 -7.748 1.189 -7.939 1.00 85.49 H

ATOM 587 HD3 ARG A 35 -8.462 2.698 -8.456 1.00 85.49 H

ATOM 588 NE ARG A 35 -8.650 2.327 -6.389 1.00 85.49 N

ATOM 589 HE ARG A 35 -9.076 3.220 -6.187 1.00 85.49 H

ATOM 590 NH1 ARG A 35 -8.309 0.166 -5.669 1.00 85.49 N

ATOM 591 HH11 ARG A 35 -8.504 -0.586 -5.023 1.00 85.49 H

ATOM 592 HH12 ARG A 35 -7.723 -0.014 -6.472 1.00 85.49 H

ATOM 593 NH2 ARG A 35 -9.578 1.542 -4.461 1.00 85.49 N

ATOM 594 HH21 ARG A 35 -10.074 2.414 -4.346 1.00 85.49 H

ATOM 595 HH22 ARG A 35 -9.668 0.805 -3.776 1.00 85.49 H

ATOM 596 CZ ARG A 35 -8.840 1.348 -5.519 1.00 85.49 C

ATOM 597 N LEU A 36 -5.493 7.426 -8.294 1.00 85.90 N

ATOM 598 H LEU A 36 -5.788 7.516 -7.332 1.00 85.90 H

ATOM 599 CA LEU A 36 -5.619 8.589 -9.176 1.00 85.90 C

ATOM 600 HA LEU A 36 -6.029 8.247 -10.127 1.00 85.90 H

ATOM 601 C LEU A 36 -4.258 9.220 -9.493 1.00 85.90 C

ATOM 602 CB LEU A 36 -6.599 9.606 -8.554 1.00 85.90 C

ATOM 603 HB2 LEU A 36 -7.229 9.123 -7.807 1.00 85.90 H

ATOM 604 HB3 LEU A 36 -6.028 10.383 -8.046 1.00 85.90 H

ATOM 605 O LEU A 36 -3.979 9.521 -10.649 1.00 85.90 O

ATOM 606 CG LEU A 36 -7.537 10.252 -9.586 1.00 85.90 C

ATOM 607 HG LEU A 36 -6.964 10.570 -10.458 1.00 85.90 H

ATOM 608 CD1 LEU A 36 -8.632 9.270 -10.019 1.00 85.90 C

ATOM 609 HD11 LEU A 36 -9.294 9.759 -10.735 1.00 85.90 H

ATOM 610 HD12 LEU A 36 -8.195 8.395 -10.499 1.00 85.90 H

ATOM 611 HD13 LEU A 36 -9.219 8.954 -9.156 1.00 85.90 H

ATOM 612 CD2 LEU A 36 -8.217 11.473 -8.971 1.00 85.90 C

ATOM 613 HD21 LEU A 36 -8.793 11.185 -8.091 1.00 85.90 H

ATOM 614 HD22 LEU A 36 -7.466 12.210 -8.685 1.00 85.90 H

ATOM 615 HD23 LEU A 36 -8.885 11.930 -9.701 1.00 85.90 H

ATOM 616 N VAL A 37 -3.409 9.382 -8.471 1.00 84.27 N

ATOM 617 H VAL A 37 -3.716 9.095 -7.552 1.00 84.27 H

ATOM 618 CA VAL A 37 -2.111 10.069 -8.569 1.00 84.27 C

ATOM 619 HA VAL A 37 -2.181 10.847 -9.329 1.00 84.27 H

ATOM 620 C VAL A 37 -1.016 9.109 -9.013 1.00 84.27 C

ATOM 621 CB VAL A 37 -1.729 10.742 -7.233 1.00 84.27 C

ATOM 622 HB VAL A 37 -1.585 9.977 -6.469 1.00 84.27 H

ATOM 623 O VAL A 37 -0.286 9.396 -9.955 1.00 84.27 O

ATOM 624 CG1 VAL A 37 -0.428 11.545 -7.352 1.00 84.27 C

ATOM 625 HG11 VAL A 37 -0.543 12.329 -8.100 1.00 84.27 H

ATOM 626 HG12 VAL A 37 -0.184 12.003 -6.394 1.00 84.27 H

ATOM 627 HG13 VAL A 37 0.402 10.904 -7.647 1.00 84.27 H

ATOM 628 CG2 VAL A 37 -2.826 11.705 -6.759 1.00 84.27 C

ATOM 629 HG21 VAL A 37 -3.742 11.161 -6.530 1.00 84.27 H

ATOM 630 HG22 VAL A 37 -3.032 12.442 -7.535 1.00 84.27 H

ATOM 631 HG23 VAL A 37 -2.501 12.226 -5.859 1.00 84.27 H

ATOM 632 N LEU A 38 -0.913 7.949 -8.357 1.00 82.76 N

ATOM 633 H LEU A 38 -1.548 7.766 -7.594 1.00 82.76 H

ATOM 634 CA LEU A 38 0.120 6.957 -8.673 1.00 82.76 C

ATOM 635 HA LEU A 38 0.998 7.468 -9.067 1.00 82.76 H

ATOM 636 C LEU A 38 -0.306 5.975 -9.770 1.00 82.76 C

ATOM 637 CB LEU A 38 0.548 6.216 -7.396 1.00 82.76 C

ATOM 638 HB2 LEU A 38 0.849 5.204 -7.668 1.00 82.76 H

ATOM 639 HB3 LEU A 38 -0.299 6.135 -6.715 1.00 82.76 H

ATOM 640 O LEU A 38 0.540 5.245 -10.277 1.00 82.76 O

ATOM 641 CG LEU A 38 1.722 6.849 -6.639 1.00 82.76 C

ATOM 642 HG LEU A 38 2.607 6.872 -7.276 1.00 82.76 H

ATOM 643 CD1 LEU A 38 1.437 8.255 -6.119 1.00 82.76 C

ATOM 644 HD11 LEU A 38 0.531 8.260 -5.513 1.00 82.76 H

ATOM 645 HD12 LEU A 38 1.318 8.933 -6.964 1.00 82.76 H

ATOM 646 HD13 LEU A 38 2.278 8.609 -5.522 1.00 82.76 H

ATOM 647 CD2 LEU A 38 1.989 5.950 -5.439 1.00 82.76 C

ATOM 648 HD21 LEU A 38 2.357 4.990 -5.803 1.00 82.76 H

ATOM 649 HD22 LEU A 38 2.716 6.408 -4.769 1.00 82.76 H

ATOM 650 HD23 LEU A 38 1.069 5.791 -4.877 1.00 82.76 H

ATOM 651 N ARG A 39 -1.598 5.926 -10.129 1.00 77.81 N

ATOM 652 H ARG A 39 -2.228 6.591 -9.702 1.00 77.81 H

ATOM 653 CA ARG A 39 -2.160 4.909 -11.037 1.00 77.81 C

ATOM 654 HA ARG A 39 -3.246 4.939 -10.951 1.00 77.81 H

ATOM 655 C ARG A 39 -1.818 3.481 -10.599 1.00 77.81 C

ATOM 656 CB ARG A 39 -1.842 5.279 -12.494 1.00 77.81 C

ATOM 657 HB2 ARG A 39 -0.821 4.988 -12.741 1.00 77.81 H

ATOM 658 HB3 ARG A 39 -1.920 6.361 -12.591 1.00 77.81 H

ATOM 659 O ARG A 39 -1.544 2.608 -11.415 1.00 77.81 O

ATOM 660 CG ARG A 39 -2.828 4.627 -13.478 1.00 77.81 C

ATOM 661 HG2 ARG A 39 -2.574 3.574 -13.593 1.00 77.81 H

ATOM 662 HG3 ARG A 39 -3.845 4.693 -13.092 1.00 77.81 H

ATOM 663 CD ARG A 39 -2.782 5.297 -14.855 1.00 77.81 C

ATOM 664 HD2 ARG A 39 -3.303 4.644 -15.555 1.00 77.81 H

ATOM 665 HD3 ARG A 39 -1.744 5.389 -15.174 1.00 77.81 H

ATOM 666 NE ARG A 39 -3.434 6.624 -14.845 1.00 77.81 N

ATOM 667 HE ARG A 39 -3.635 7.022 -13.939 1.00 77.81 H

ATOM 668 NH1 ARG A 39 -3.569 6.912 -17.120 1.00 77.81 N

ATOM 669 HH11 ARG A 39 -3.891 7.443 -17.916 1.00 77.81 H

ATOM 670 HH12 ARG A 39 -3.123 6.015 -17.251 1.00 77.81 H

ATOM 671 NH2 ARG A 39 -4.425 8.461 -15.762 1.00 77.81 N

ATOM 672 HH21 ARG A 39 -4.719 8.990 -16.571 1.00 77.81 H

ATOM 673 HH22 ARG A 39 -4.615 8.823 -14.838 1.00 77.81 H

ATOM 674 CZ ARG A 39 -3.804 7.324 -15.904 1.00 77.81 C

ATOM 675 N CYS A 40 -1.836 3.255 -9.285 1.00 75.78 N

ATOM 676 H CYS A 40 -2.064 4.023 -8.671 1.00 75.78 H

ATOM 677 CA CYS A 40 -1.531 1.959 -8.702 1.00 75.78 C

ATOM 678 HA CYS A 40 -0.682 1.539 -9.240 1.00 75.78 H

ATOM 679 C CYS A 40 -2.729 1.010 -8.845 1.00 75.78 C

ATOM 680 CB CYS A 40 -1.133 2.155 -7.233 1.00 75.78 C

ATOM 681 HB2 CYS A 40 -0.686 3.141 -7.103 1.00 75.78 H

ATOM 682 HB3 CYS A 40 -2.021 2.102 -6.603 1.00 75.78 H

ATOM 683 O CYS A 40 -3.779 1.280 -8.261 1.00 75.78 O

ATOM 684 SG CYS A 40 0.070 0.928 -6.698 1.00 75.78 S

ATOM 685 N SER A 41 -2.574 -0.076 -9.610 1.00 70.93 N

ATOM 686 H SER A 41 -1.695 -0.211 -10.089 1.00 70.93 H

ATOM 687 CA SER A 41 -3.621 -1.094 -9.793 1.00 70.93 C

ATOM 688 HA SER A 41 -4.572 -0.579 -9.934 1.00 70.93 H

ATOM 689 C SER A 41 -3.809 -1.990 -8.575 1.00 70.93 C

ATOM 690 CB SER A 41 -3.364 -1.930 -11.041 1.00 70.93 C

ATOM 691 HB2 SER A 41 -3.142 -1.287 -11.893 1.00 70.93 H

ATOM 692 HB3 SER A 41 -2.533 -2.614 -10.869 1.00 70.93 H

ATOM 693 O SER A 41 -2.799 -2.363 -7.928 1.00 70.93 O

ATOM 694 OG SER A 41 -4.547 -2.651 -11.294 1.00 70.93 O

ATOM 695 HG SER A 41 -4.944 -2.856 -10.444 1.00 70.93 H

ATOM 696 OXT SER A 41 -4.986 -2.311 -8.331 1.00 70.93 O

END

# ModelArchive > Procedures & Data

**AlphaFold Input FASTA sequence for SMB**

>SMB

FPIPLPYCWLCRALIKRIQAMIPKGGRMLPQLVCRLVLRCS


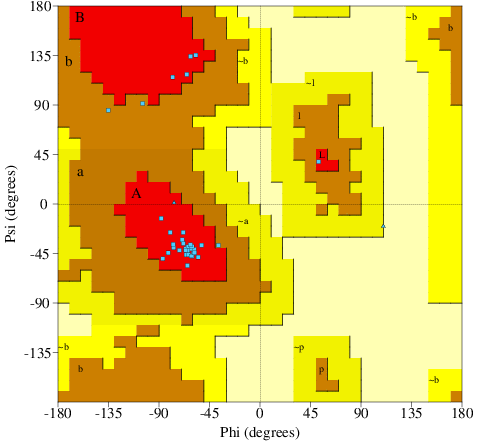


Model structural quality of the molecular dynamics refined structure analyzed by PROCHECK (Laskowski et al., 1993, 1996) generated with PDBsum (<https://ebi.ac.uk>).

**PROCHECK statistics**

**1. Ramachandran Plot statistics**

**No. of**

**residues %-tage**

**------ ------**

Most favoured regions [A,B,L] 29 90.6%

Additional allowed regions [a,b,l,p] 3 9.4%

Generously allowed regions [~a,~b,~l,~p] 0 0.0%

Disallowed regions [XX] 0 0.0%

---- ------

Non-glycine and non-proline residues 32 100.0%

End-residues (excl. Gly and Pro) 2

Glycine residues 2

Proline residues 5

----

Total number of residues 41

Based on an analysis of **118** structures of resolution of at least **2.0** Angstroms and *R*-factor no greater than **20.0** a good quality model would be expected to have over **90%** in the most favoured regions [A,B,L].

**2. G-Factors**

**Average**

**Parameter Score Score**

**--------- ----- -----**

Dihedral angles:-

Phi-psi distribution 0.21

Chi1-chi2 distribution -0.68*

Chi1 only 0.28

Chi3 & chi4 0.63

Omega -0.69*

-0.18

=====

Main-chain covalent forces:-

Main-chain bond lengths 0.54

Main-chain bond angles -0.10

0.17

=====

OVERALL AVERAGE -0.03

=====

**G-factors** provide a measure of how **unusual**, or out-of-the-ordinary, a property is.

Values below -0.5* - unusual

Values below **-1.0**** - highly unusual

**Important note:** The main-chain bond-lengths and bond angles are compared with the Engh & Huber (1991) ideal values derived from small-molecule data. Therefore, structures refined using different restraints may show apparently large deviations from normality.

| \| \| 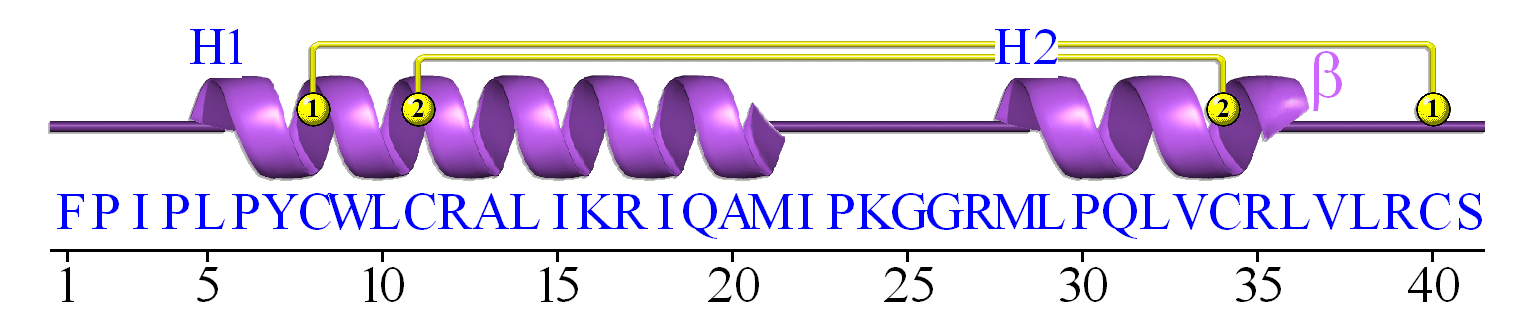 \| \| --- \| \| \| --- \| --- \| \|  \|     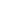 |
| --- | --- | --- | --- |
| \| Disulphides \| \| --- \| \|  \| \| \|  \| \| \| \| \| \| \| \| \| \| \| \| \| \| \| \| --- \| --- \| --- \| --- \| --- \| --- \| --- \| --- \| --- \| --- \| --- \| --- \| --- \| --- \| --- \| \| **1st cysteine** \|  \| **2nd cysteine** \|  \| **Type** \|  \| **Chi1** \|  \| **Chi2** \|  \| **Chi3** \|  \| **Chi2p** \|  \| **Chi1p** \| \|  \| \| \| \| \| \| \| \| \| \| \| \| \| \| \| \| A 8 \|  \| A 40 \|  \| RHH \|  \| -109.1 \|  \| -73.7 \|  \| 142.1 \|  \| 75.0 \|  \| -148.3 \| \| A 11 \|  \| A 34 \|  \| RHH \|  \| -171.2 \|  \| -110.1 \|  \| 172.6 \|  \| 72.8 \|  \| -142.2 \| \|  \| \| \| \| \| \| \| \| \| \| \| \| \| \| \| \| \|  \| |


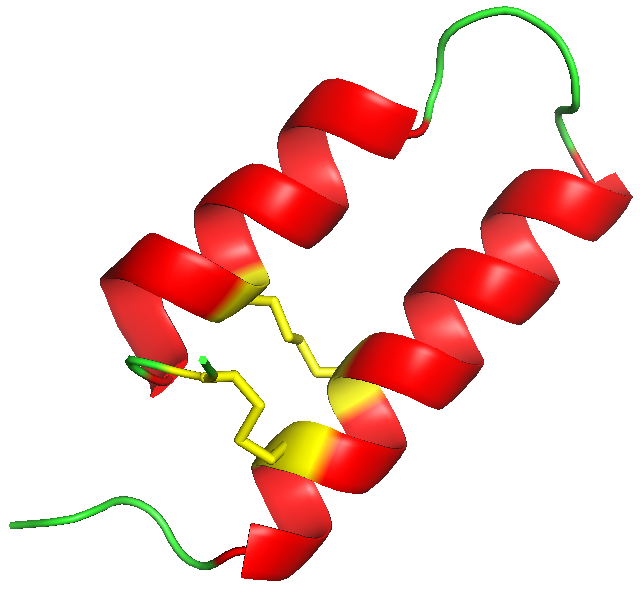

Supplement: S2 File — (DOCX) [file pone.0276787.s002.docx]
