# Supplementary material for: Emulation of the structure of the Saposin protein fold by a lung surfactant peptide construct of surfactant Protein B
Source: PLoS One. 2022 Nov 3;17(11):e0276787. doi: 10.1371/journal.pone.0276787 (PMC9632872; doi:10.1371/journal.pone.0276787)
Supplement: S3 File — (DOCX) [file pone.0276787.s003.docx]

**S3** **- deposited in the ModelArchive (**<https://modelarchive.org>/doi/5452/ma-abz44**)**

**Emulation of the Structure of the Saposin Protein Fold by a Lung Surfactant Peptide Construct of Surfactant Protein B**

Alan J. Waring^1,2^, Julian P. Whitelegge^3^, Shantanu K. Sharma^4^, Larry M. Gordon^1^,

Frans J. Walther^1,5,*^

^1^ Lundquist Institute for Biomedical Innovation at Harbor-UCLA Medical Center

1124 West Carson Street

Torrance, CA, USA

^2^ Department of Medicine

David Geffen School of Medicine

University of California Los Angeles

405 Hilgard Avenue

Los Angeles, CA, USA

^3^ NPI-Semel Institute for Neuroscience & Human Behavior Department of Psychiatry

& Biobehavioral Sciences, David Geffen School of Medicine at UCLA,

760 Westwood Plaza, Los Angeles, CA, USA

^4^ Materials and Process Simulation Center

California Institute of Technology

1200 East California Boulevard

Pasadena, CA, USA

^5^ Department of Pediatrics

David Geffen School of Medicine

University of California Los Angeles

405 Hilgard Avenue

Los Angeles, CA, USA

# **ModelArchive (**[**https://modelarchive.org**](https://modelarchive.org)**/doi/10.5452/ma-abz44)**

**Lowest energy molecular coordinate set for Molecular Dynamics of SMB peptide in simulated surfactant lipid bilayer using the iTasser homology predicted structure and residue specific molecular constraints from experimental measures as a starting conformation.**

ATOM 1 N PHE A 1 7.870 43.080 27.370 1.00 0.00 N

ATOM 2 CA PHE A 1 7.600 43.050 28.820 1.00 0.00 C

ATOM 3 C PHE A 1 8.890 43.160 29.600 1.00 0.00 C

ATOM 4 O PHE A 1 9.830 42.550 29.190 1.00 0.00 O

ATOM 5 CB PHE A 1 6.850 41.740 29.130 1.00 0.00 C

ATOM 6 CG PHE A 1 6.060 41.870 30.340 1.00 0.00 C

ATOM 7 CD1 PHE A 1 4.990 42.690 30.410 1.00 0.00 C

ATOM 8 CD2 PHE A 1 6.370 41.150 31.460 1.00 0.00 C

ATOM 9 CE1 PHE A 1 4.260 42.840 31.580 1.00 0.00 C

ATOM 10 CE2 PHE A 1 5.680 41.300 32.630 1.00 0.00 C

ATOM 11 CZ PHE A 1 4.640 42.160 32.700 1.00 0.00 C

ATOM 12 HA PHE A 1 6.990 43.890 29.010 1.00 0.00 H

ATOM 13 HB1 PHE A 1 7.490 40.890 29.170 1.00 0.00 H

ATOM 14 HB2 PHE A 1 6.130 41.500 28.350 1.00 0.00 H

ATOM 15 HD1 PHE A 1 4.690 43.220 29.560 1.00 0.00 H

ATOM 16 HD2 PHE A 1 7.150 40.470 31.410 1.00 0.00 H

ATOM 17 HE1 PHE A 1 3.420 43.450 31.610 1.00 0.00 H

ATOM 18 HE2 PHE A 1 5.970 40.760 33.480 1.00 0.00 H

ATOM 19 HZ PHE A 1 4.120 42.300 33.590 1.00 0.00 H

ATOM 20 HT1 PHE A 1 8.490 43.130 27.120 1.00 0.00 H

ATOM 21 HT2 PHE A 1 7.590 43.530 26.900 1.00 0.00 H

ATOM 22 HT3 PHE A 1 7.700 42.550 26.970 1.00 0.00 H

ATOM 23 N PRO A 2 9.040 43.880 30.680 1.00 0.00 N

ATOM 24 CA PRO A 2 10.330 44.280 31.200 1.00 0.00 C

ATOM 25 C PRO A 2 10.940 43.300 32.160 1.00 0.00 C

ATOM 26 O PRO A 2 11.780 43.700 32.940 1.00 0.00 O

ATOM 27 CB PRO A 2 10.000 45.580 31.890 1.00 0.00 C

ATOM 28 CG PRO A 2 8.620 45.410 32.380 1.00 0.00 C

ATOM 29 CD PRO A 2 7.970 44.640 31.310 1.00 0.00 C

ATOM 30 HA PRO A 2 11.030 44.380 30.420 1.00 0.00 H

ATOM 31 HB1 PRO A 2 9.970 46.370 31.160 1.00 0.00 H

ATOM 32 HB2 PRO A 2 10.690 45.850 32.680 1.00 0.00 H

ATOM 33 HG1 PRO A 2 8.610 44.840 33.300 1.00 0.00 H

ATOM 34 HG2 PRO A 2 8.130 46.360 32.570 1.00 0.00 H

ATOM 35 HD1 PRO A 2 7.200 44.000 31.700 1.00 0.00 H

ATOM 36 HD2 PRO A 2 7.510 45.330 30.620 1.00 0.00 H

ATOM 37 N ILE A 3 10.570 42.010 32.110 1.00 0.00 N

ATOM 38 CA ILE A 3 11.000 41.030 33.050 1.00 0.00 C

ATOM 39 C ILE A 3 11.820 40.010 32.320 1.00 0.00 C

ATOM 40 O ILE A 3 11.260 39.270 31.510 1.00 0.00 O

ATOM 41 CB ILE A 3 9.810 40.410 33.770 1.00 0.00 C

ATOM 42 CG1 ILE A 3 8.920 41.420 34.460 1.00 0.00 C

ATOM 43 CG2 ILE A 3 10.250 39.340 34.710 1.00 0.00 C

ATOM 44 CD ILE A 3 9.560 42.130 35.570 1.00 0.00 C

ATOM 45 HN ILE A 3 10.000 41.710 31.380 1.00 0.00 H

ATOM 46 HA ILE A 3 11.600 41.490 33.800 1.00 0.00 H

ATOM 47 HB ILE A 3 9.190 39.920 33.060 1.00 0.00 H

ATOM 48 1HG1 ILE A 3 8.040 40.930 34.860 1.00 0.00 H

ATOM 49 2HG1 ILE A 3 8.550 42.140 33.750 1.00 0.00 H

ATOM 50 1HG2 ILE A 3 10.090 38.900 34.730 1.00 0.00 H

ATOM 51 2HG2 ILE A 3 10.220 39.250 35.240 1.00 0.00 H

ATOM 52 3HG2 ILE A 3 10.720 38.990 34.880 1.00 0.00 H

ATOM 53 HD1 ILE A 3 9.390 42.310 36.060 1.00 0.00 H

ATOM 54 HD2 ILE A 3 9.780 42.600 35.590 1.00 0.00 H

ATOM 55 HD3 ILE A 3 9.980 42.010 35.900 1.00 0.00 H

ATOM 56 N PRO A 4 13.140 39.900 32.490 1.00 0.00 N

ATOM 57 CA PRO A 4 13.910 38.720 32.100 1.00 0.00 C

ATOM 58 C PRO A 4 13.400 37.400 32.630 1.00 0.00 C

ATOM 59 O PRO A 4 13.000 37.330 33.740 1.00 0.00 O

ATOM 60 CB PRO A 4 15.310 39.030 32.550 1.00 0.00 C

ATOM 61 CG PRO A 4 15.360 40.530 32.600 1.00 0.00 C

ATOM 62 CD PRO A 4 13.980 40.920 33.040 1.00 0.00 C

ATOM 63 HA PRO A 4 13.840 38.660 31.040 1.00 0.00 H

ATOM 64 HB1 PRO A 4 16.030 38.620 31.850 1.00 0.00 H

ATOM 65 HB2 PRO A 4 15.530 38.620 33.530 1.00 0.00 H

ATOM 66 HG1 PRO A 4 16.120 40.890 33.270 1.00 0.00 H

ATOM 67 HG2 PRO A 4 15.530 40.920 31.630 1.00 0.00 H

ATOM 68 HD1 PRO A 4 13.880 40.920 34.110 1.00 0.00 H

ATOM 69 HD2 PRO A 4 13.720 41.880 32.620 1.00 0.00 H

ATOM 70 N LEU A 5 13.380 36.360 31.820 1.00 0.00 N

ATOM 71 CA LEU A 5 12.810 35.070 32.160 1.00 0.00 C

ATOM 72 C LEU A 5 13.770 34.160 32.930 1.00 0.00 C

ATOM 73 O LEU A 5 14.960 34.150 32.640 1.00 0.00 O

ATOM 74 CB LEU A 5 12.240 34.360 30.920 1.00 0.00 C

ATOM 75 CG LEU A 5 11.000 35.040 30.310 1.00 0.00 C

ATOM 76 CD1 LEU A 5 10.890 35.260 29.200 1.00 0.00 C

ATOM 77 CD2 LEU A 5 9.970 34.590 30.470 1.00 0.00 C

ATOM 78 HN LEU A 5 13.750 36.410 30.950 1.00 0.00 H

ATOM 79 HA LEU A 5 11.960 35.260 32.780 1.00 0.00 H

ATOM 80 HB1 LEU A 5 11.890 33.360 31.200 1.00 0.00 H

ATOM 81 HB2 LEU A 5 13.010 34.240 30.170 1.00 0.00 H

ATOM 82 HG LEU A 5 10.900 35.730 30.620 1.00 0.00 H

ATOM 83 1HD1 LEU A 5 11.590 35.640 29.050 1.00 0.00 H

ATOM 84 2HD1 LEU A 5 10.050 35.740 28.760 1.00 0.00 H

ATOM 85 3HD1 LEU A 5 11.000 34.590 28.900 1.00 0.00 H

ATOM 86 1HD2 LEU A 5 9.810 34.530 31.070 1.00 0.00 H

ATOM 87 2HD2 LEU A 5 10.090 34.080 30.300 1.00 0.00 H

ATOM 88 3HD2 LEU A 5 9.150 34.890 30.150 1.00 0.00 H

ATOM 89 N PRO A 6 13.320 33.380 33.920 1.00 0.00 N

ATOM 90 CA PRO A 6 14.180 32.870 34.970 1.00 0.00 C

ATOM 91 C PRO A 6 15.090 31.740 34.540 1.00 0.00 C

ATOM 92 O PRO A 6 16.250 31.750 34.940 1.00 0.00 O

ATOM 93 CB PRO A 6 13.230 32.440 36.090 1.00 0.00 C

ATOM 94 CG PRO A 6 11.910 32.170 35.400 1.00 0.00 C

ATOM 95 CD PRO A 6 11.910 33.210 34.290 1.00 0.00 C

ATOM 96 HA PRO A 6 14.810 33.670 35.290 1.00 0.00 H

ATOM 97 HB1 PRO A 6 13.110 33.230 36.830 1.00 0.00 H

ATOM 98 HB2 PRO A 6 13.570 31.550 36.640 1.00 0.00 H

ATOM 99 HG1 PRO A 6 11.910 31.180 34.940 1.00 0.00 H

ATOM 100 HG2 PRO A 6 11.020 32.250 36.030 1.00 0.00 H

ATOM 101 HD1 PRO A 6 11.300 32.870 33.450 1.00 0.00 H

ATOM 102 HD2 PRO A 6 11.540 34.170 34.660 1.00 0.00 H

ATOM 103 N TYR A 7 14.610 30.770 33.750 1.00 0.00 N

ATOM 104 CA TYR A 7 15.350 29.640 33.300 1.00 0.00 C

ATOM 105 C TYR A 7 16.310 30.050 32.210 1.00 0.00 C

ATOM 106 O TYR A 7 17.410 29.510 32.110 1.00 0.00 O

ATOM 107 CB TYR A 7 14.380 28.540 32.820 1.00 0.00 C

ATOM 108 CG TYR A 7 15.000 27.170 32.620 1.00 0.00 C

ATOM 109 CD1 TYR A 7 15.590 26.540 33.670 1.00 0.00 C

ATOM 110 CD2 TYR A 7 14.890 26.500 31.440 1.00 0.00 C

ATOM 111 CE1 TYR A 7 16.020 25.210 33.570 1.00 0.00 C

ATOM 112 CE2 TYR A 7 15.300 25.170 31.340 1.00 0.00 C

ATOM 113 CZ TYR A 7 15.870 24.520 32.390 1.00 0.00 C

ATOM 114 OH TYR A 7 16.260 23.190 32.310 1.00 0.00 O

ATOM 115 HN TYR A 7 13.700 30.860 33.390 1.00 0.00 H

ATOM 116 HA TYR A 7 15.920 29.250 34.130 1.00 0.00 H

ATOM 117 HB1 TYR A 7 13.840 28.800 31.910 1.00 0.00 H

ATOM 118 HB2 TYR A 7 13.630 28.430 33.610 1.00 0.00 H

ATOM 119 HD1 TYR A 7 15.680 27.030 34.600 1.00 0.00 H

ATOM 120 HD2 TYR A 7 14.380 26.950 30.650 1.00 0.00 H

ATOM 121 HE1 TYR A 7 16.480 24.700 34.370 1.00 0.00 H

ATOM 122 HE2 TYR A 7 15.190 24.670 30.430 1.00 0.00 H

ATOM 123 HH TYR A 7 15.950 22.870 31.470 1.00 0.00 H

ATOM 124 N CYS A 8 15.970 31.080 31.410 1.00 0.00 N

ATOM 125 CA CYS A 8 16.850 31.750 30.500 1.00 0.00 C

ATOM 126 C CYS A 8 18.020 32.410 31.190 1.00 0.00 C

ATOM 127 O CYS A 8 19.150 32.330 30.720 1.00 0.00 O

ATOM 128 CB CYS A 8 16.140 32.720 29.530 1.00 0.00 C

ATOM 129 SG CYS A 8 17.190 33.240 28.130 1.00 0.00 S

ATOM 130 HN CYS A 8 15.050 31.450 31.510 1.00 0.00 H

ATOM 131 HA CYS A 8 17.290 30.970 29.900 1.00 0.00 H

ATOM 132 HB1 CYS A 8 15.770 33.610 30.050 1.00 0.00 H

ATOM 133 HB2 CYS A 8 15.280 32.180 29.150 1.00 0.00 H

ATOM 134 N TRP A 9 17.820 33.070 32.350 1.00 0.00 N

ATOM 135 CA TRP A 9 18.940 33.560 33.130 1.00 0.00 C

ATOM 136 C TRP A 9 19.840 32.460 33.650 1.00 0.00 C

ATOM 137 O TRP A 9 21.040 32.600 33.510 1.00 0.00 O

ATOM 138 CB TRP A 9 18.460 34.550 34.240 1.00 0.00 C

ATOM 139 CG TRP A 9 19.540 35.100 35.110 1.00 0.00 C

ATOM 140 CD1 TRP A 9 20.390 36.120 34.800 1.00 0.00 C

ATOM 141 CD2 TRP A 9 19.910 34.660 36.410 1.00 0.00 C

ATOM 142 CE2 TRP A 9 20.940 35.520 36.830 1.00 0.00 C

ATOM 143 CE3 TRP A 9 19.480 33.640 37.210 1.00 0.00 C

ATOM 144 NE1 TRP A 9 21.220 36.360 35.840 1.00 0.00 N

ATOM 145 CZ2 TRP A 9 21.520 35.400 38.060 1.00 0.00 C

ATOM 146 CZ3 TRP A 9 20.090 33.470 38.440 1.00 0.00 C

ATOM 147 CH2 TRP A 9 21.080 34.340 38.830 1.00 0.00 C

ATOM 148 HN TRP A 9 16.900 33.190 32.690 1.00 0.00 H

ATOM 149 HA TRP A 9 19.550 34.120 32.450 1.00 0.00 H

ATOM 150 HB1 TRP A 9 17.730 34.050 34.880 1.00 0.00 H

ATOM 151 HB2 TRP A 9 17.960 35.380 33.720 1.00 0.00 H

ATOM 152 HD1 TRP A 9 20.340 36.630 33.880 1.00 0.00 H

ATOM 153 HE1 TRP A 9 21.950 36.990 35.890 1.00 0.00 H

ATOM 154 HE3 TRP A 9 18.680 33.000 36.930 1.00 0.00 H

ATOM 155 HZ2 TRP A 9 22.260 36.080 38.420 1.00 0.00 H

ATOM 156 HZ3 TRP A 9 19.760 32.700 39.070 1.00 0.00 H

ATOM 157 HH2 TRP A 9 21.490 34.180 39.790 1.00 0.00 H

ATOM 158 N LEU A 10 19.290 31.360 34.180 1.00 0.00 N

ATOM 159 CA LEU A 10 20.080 30.200 34.590 1.00 0.00 C

ATOM 160 C LEU A 10 20.930 29.570 33.490 1.00 0.00 C

ATOM 161 O LEU A 10 22.090 29.280 33.720 1.00 0.00 O

ATOM 162 CB LEU A 10 19.200 29.110 35.240 1.00 0.00 C

ATOM 163 CG LEU A 10 18.580 29.430 36.600 1.00 0.00 C

ATOM 164 CD1 LEU A 10 17.600 28.370 37.000 1.00 0.00 C

ATOM 165 CD2 LEU A 10 19.600 29.600 37.700 1.00 0.00 C

ATOM 166 HN LEU A 10 18.320 31.330 34.300 1.00 0.00 H

ATOM 167 HA LEU A 10 20.780 30.530 35.340 1.00 0.00 H

ATOM 168 HB1 LEU A 10 19.790 28.210 35.380 1.00 0.00 H

ATOM 169 HB2 LEU A 10 18.400 28.820 34.560 1.00 0.00 H

ATOM 170 HG LEU A 10 18.070 30.370 36.530 1.00 0.00 H

ATOM 171 1HD1 LEU A 10 17.010 28.450 37.360 1.00 0.00 H

ATOM 172 2HD1 LEU A 10 17.760 27.830 37.370 1.00 0.00 H

ATOM 173 3HD1 LEU A 10 17.290 28.040 36.580 1.00 0.00 H

ATOM 174 1HD2 LEU A 10 19.170 29.790 38.660 1.00 0.00 H

ATOM 175 2HD2 LEU A 10 20.230 30.400 37.450 1.00 0.00 H

ATOM 176 3HD2 LEU A 10 20.190 28.740 37.790 1.00 0.00 H

ATOM 177 N CYS A 11 20.380 29.350 32.270 1.00 0.00 N

ATOM 178 CA CYS A 11 21.200 28.900 31.150 1.00 0.00 C

ATOM 179 C CYS A 11 22.230 29.900 30.650 1.00 0.00 C

ATOM 180 O CYS A 11 23.360 29.540 30.380 1.00 0.00 O

ATOM 181 CB CYS A 11 20.340 28.370 29.970 1.00 0.00 C

ATOM 182 SG CYS A 11 19.330 29.590 29.120 1.00 0.00 S

ATOM 183 HN CYS A 11 19.440 29.580 32.120 1.00 0.00 H

ATOM 184 HA CYS A 11 21.760 28.060 31.500 1.00 0.00 H

ATOM 185 HB1 CYS A 11 19.660 27.620 30.380 1.00 0.00 H

ATOM 186 HB2 CYS A 11 21.040 27.900 29.260 1.00 0.00 H

ATOM 187 N ARG A 12 21.870 31.190 30.560 1.00 0.00 N

ATOM 188 CA ARG A 12 22.780 32.260 30.180 1.00 0.00 C

ATOM 189 C ARG A 12 23.970 32.380 31.130 1.00 0.00 C

ATOM 190 O ARG A 12 25.120 32.410 30.720 1.00 0.00 O

ATOM 191 CB ARG A 12 21.960 33.560 30.100 1.00 0.00 C

ATOM 192 CG ARG A 12 22.640 34.840 29.570 1.00 0.00 C

ATOM 193 CD ARG A 12 23.450 35.630 30.560 1.00 0.00 C

ATOM 194 NE ARG A 12 23.750 36.910 29.890 1.00 0.00 N

ATOM 195 CZ ARG A 12 24.730 37.730 30.160 1.00 0.00 C

ATOM 196 NH1 ARG A 12 24.880 38.830 29.490 1.00 0.00 N1+

ATOM 197 NH2 ARG A 12 25.600 37.550 31.080 1.00 0.00 N

ATOM 198 HN ARG A 12 20.940 31.450 30.740 1.00 0.00 H

ATOM 199 HA ARG A 12 23.160 31.990 29.220 1.00 0.00 H

ATOM 200 HB1 ARG A 12 21.480 33.780 31.070 1.00 0.00 H

ATOM 201 HB2 ARG A 12 21.120 33.380 29.420 1.00 0.00 H

ATOM 202 HG1 ARG A 12 21.830 35.490 29.230 1.00 0.00 H

ATOM 203 HG2 ARG A 12 23.250 34.590 28.700 1.00 0.00 H

ATOM 204 HD1 ARG A 12 24.400 35.130 30.800 1.00 0.00 H

ATOM 205 HD2 ARG A 12 22.900 35.820 31.480 1.00 0.00 H

ATOM 206 HE ARG A 12 23.130 37.230 29.180 1.00 0.00 H

ATOM 207 1HH1 ARG A 12 25.540 39.470 29.780 1.00 0.00 H

ATOM 208 2HH1 ARG A 12 24.150 39.130 28.960 1.00 0.00 H

ATOM 209 1HH2 ARG A 12 26.270 38.230 31.170 1.00 0.00 H

ATOM 210 2HH2 ARG A 12 25.530 36.780 31.680 1.00 0.00 H

ATOM 211 N ALA A 13 23.680 32.380 32.440 1.00 0.00 N

ATOM 212 CA ALA A 13 24.650 32.380 33.510 1.00 0.00 C

ATOM 213 C ALA A 13 25.600 31.200 33.520 1.00 0.00 C

ATOM 214 O ALA A 13 26.810 31.350 33.600 1.00 0.00 O

ATOM 215 CB ALA A 13 23.920 32.550 34.850 1.00 0.00 C

ATOM 216 HN ALA A 13 22.740 32.340 32.730 1.00 0.00 H

ATOM 217 HA ALA A 13 25.280 33.240 33.370 1.00 0.00 H

ATOM 218 HB1 ALA A 13 23.280 31.680 35.050 1.00 0.00 H

ATOM 219 HB2 ALA A 13 23.290 33.440 34.830 1.00 0.00 H

ATOM 220 HB3 ALA A 13 24.610 32.650 35.690 1.00 0.00 H

ATOM 221 N LEU A 14 25.040 29.980 33.350 1.00 0.00 N

ATOM 222 CA LEU A 14 25.800 28.750 33.240 1.00 0.00 C

ATOM 223 C LEU A 14 26.800 28.750 32.060 1.00 0.00 C

ATOM 224 O LEU A 14 27.970 28.500 32.260 1.00 0.00 O

ATOM 225 CB LEU A 14 24.810 27.580 33.100 1.00 0.00 C

ATOM 226 CG LEU A 14 25.430 26.190 32.890 1.00 0.00 C

ATOM 227 CD1 LEU A 14 26.110 25.720 34.090 1.00 0.00 C

ATOM 228 CD2 LEU A 14 24.420 25.220 32.450 1.00 0.00 C

ATOM 229 HN LEU A 14 24.060 29.880 33.310 1.00 0.00 H

ATOM 230 HA LEU A 14 26.360 28.600 34.130 1.00 0.00 H

ATOM 231 HB1 LEU A 14 24.170 27.810 32.250 1.00 0.00 H

ATOM 232 HB2 LEU A 14 24.150 27.560 33.970 1.00 0.00 H

ATOM 233 HG LEU A 14 26.140 26.300 32.120 1.00 0.00 H

ATOM 234 1HD1 LEU A 14 26.250 25.370 34.350 1.00 0.00 H

ATOM 235 2HD1 LEU A 14 26.090 25.730 34.480 1.00 0.00 H

ATOM 236 3HD1 LEU A 14 26.490 25.700 34.370 1.00 0.00 H

ATOM 237 1HD2 LEU A 14 23.690 25.080 33.160 1.00 0.00 H

ATOM 238 2HD2 LEU A 14 24.800 24.260 32.210 1.00 0.00 H

ATOM 239 3HD2 LEU A 14 23.990 25.580 31.610 1.00 0.00 H

ATOM 240 N ILE A 15 26.360 29.110 30.850 1.00 0.00 N

ATOM 241 CA ILE A 15 27.190 29.140 29.660 1.00 0.00 C

ATOM 242 C ILE A 15 28.280 30.180 29.800 1.00 0.00 C

ATOM 243 O ILE A 15 29.440 29.910 29.510 1.00 0.00 O

ATOM 244 CB ILE A 15 26.380 29.310 28.400 1.00 0.00 C

ATOM 245 CG1 ILE A 15 25.390 28.120 28.180 1.00 0.00 C

ATOM 246 CG2 ILE A 15 27.260 29.520 27.160 1.00 0.00 C

ATOM 247 CD ILE A 15 26.050 26.750 28.100 1.00 0.00 C

ATOM 248 HN ILE A 15 25.420 29.340 30.730 1.00 0.00 H

ATOM 249 HA ILE A 15 27.740 28.220 29.620 1.00 0.00 H

ATOM 250 HB ILE A 15 25.760 30.210 28.500 1.00 0.00 H

ATOM 251 1HG1 ILE A 15 24.840 28.330 27.260 1.00 0.00 H

ATOM 252 2HG1 ILE A 15 24.660 28.110 29.000 1.00 0.00 H

ATOM 253 1HG2 ILE A 15 26.660 29.600 26.260 1.00 0.00 H

ATOM 254 2HG2 ILE A 15 27.980 28.730 27.000 1.00 0.00 H

ATOM 255 3HG2 ILE A 15 27.840 30.420 27.210 1.00 0.00 H

ATOM 256 HD1 ILE A 15 26.260 26.430 28.370 1.00 0.00 H

ATOM 257 HD2 ILE A 15 26.430 26.490 27.900 1.00 0.00 H

ATOM 258 HD3 ILE A 15 25.980 26.250 27.980 1.00 0.00 H

ATOM 259 N LYS A 16 27.950 31.360 30.350 1.00 0.00 N

ATOM 260 CA LYS A 16 28.930 32.390 30.670 1.00 0.00 C

ATOM 261 C LYS A 16 30.000 31.940 31.660 1.00 0.00 C

ATOM 262 O LYS A 16 31.180 32.150 31.460 1.00 0.00 O

ATOM 263 CB LYS A 16 28.210 33.670 31.190 1.00 0.00 C

ATOM 264 CG LYS A 16 29.040 34.800 31.780 1.00 0.00 C

ATOM 265 CD LYS A 16 30.070 35.430 30.850 1.00 0.00 C

ATOM 266 CE LYS A 16 30.960 36.510 31.470 1.00 0.00 C

ATOM 267 NZ LYS A 16 30.230 37.720 31.790 1.00 0.00 N1+

ATOM 268 HN LYS A 16 27.020 31.540 30.610 1.00 0.00 H

ATOM 269 HA LYS A 16 29.470 32.610 29.770 1.00 0.00 H

ATOM 270 HB1 LYS A 16 27.490 33.400 31.970 1.00 0.00 H

ATOM 271 HB2 LYS A 16 27.630 34.050 30.340 1.00 0.00 H

ATOM 272 HG1 LYS A 16 29.550 34.420 32.670 1.00 0.00 H

ATOM 273 HG2 LYS A 16 28.350 35.590 32.080 1.00 0.00 H

ATOM 274 HD1 LYS A 16 29.560 35.860 29.980 1.00 0.00 H

ATOM 275 HD2 LYS A 16 30.730 34.640 30.460 1.00 0.00 H

ATOM 276 HE1 LYS A 16 31.760 36.770 30.770 1.00 0.00 H

ATOM 277 HE2 LYS A 16 31.420 36.130 32.390 1.00 0.00 H

ATOM 278 HZ1 LYS A 16 29.740 38.040 30.980 1.00 0.00 H

ATOM 279 HZ2 LYS A 16 30.880 38.440 32.080 1.00 0.00 H

ATOM 280 HZ3 LYS A 16 29.560 37.610 32.540 1.00 0.00 H

ATOM 281 N ARG A 17 29.630 31.240 32.760 1.00 0.00 N

ATOM 282 CA ARG A 17 30.640 30.720 33.660 1.00 0.00 C

ATOM 283 C ARG A 17 31.550 29.640 33.060 1.00 0.00 C

ATOM 284 O ARG A 17 32.740 29.610 33.330 1.00 0.00 O

ATOM 285 CB ARG A 17 30.030 30.280 35.010 1.00 0.00 C

ATOM 286 CG ARG A 17 29.390 31.390 35.860 1.00 0.00 C

ATOM 287 CD ARG A 17 29.980 32.810 35.740 1.00 0.00 C

ATOM 288 NE ARG A 17 31.420 32.810 36.080 1.00 0.00 N

ATOM 289 CZ ARG A 17 32.200 33.860 35.900 1.00 0.00 C

ATOM 290 NH1 ARG A 17 33.500 33.700 35.980 1.00 0.00 N1+

ATOM 291 NH2 ARG A 17 31.740 35.040 35.660 1.00 0.00 N

ATOM 292 HN ARG A 17 28.680 31.050 32.950 1.00 0.00 H

ATOM 293 HA ARG A 17 31.330 31.510 33.890 1.00 0.00 H

ATOM 294 HB1 ARG A 17 30.860 29.850 35.620 1.00 0.00 H

ATOM 295 HB2 ARG A 17 29.290 29.490 34.830 1.00 0.00 H

ATOM 296 HG1 ARG A 17 29.420 31.080 36.910 1.00 0.00 H

ATOM 297 HG2 ARG A 17 28.340 31.460 35.570 1.00 0.00 H

ATOM 298 HD1 ARG A 17 29.430 33.450 36.450 1.00 0.00 H

ATOM 299 HD2 ARG A 17 29.810 33.230 34.740 1.00 0.00 H

ATOM 300 HE ARG A 17 31.900 31.950 36.170 1.00 0.00 H

ATOM 301 1HH1 ARG A 17 33.860 32.800 35.960 1.00 0.00 H

ATOM 302 2HH1 ARG A 17 34.080 34.490 35.830 1.00 0.00 H

ATOM 303 1HH2 ARG A 17 30.770 35.170 35.540 1.00 0.00 H

ATOM 304 2HH2 ARG A 17 32.370 35.740 35.490 1.00 0.00 H

ATOM 305 N ILE A 18 31.030 28.750 32.200 1.00 0.00 N

ATOM 306 CA ILE A 18 31.830 27.790 31.430 1.00 0.00 C

ATOM 307 C ILE A 18 32.770 28.460 30.460 1.00 0.00 C

ATOM 308 O ILE A 18 33.950 28.140 30.420 1.00 0.00 O

ATOM 309 CB ILE A 18 30.950 26.740 30.720 1.00 0.00 C

ATOM 310 CG1 ILE A 18 30.130 25.920 31.740 1.00 0.00 C

ATOM 311 CG2 ILE A 18 31.810 25.770 29.880 1.00 0.00 C

ATOM 312 CD ILE A 18 28.960 25.190 31.090 1.00 0.00 C

ATOM 313 HN ILE A 18 30.050 28.750 32.030 1.00 0.00 H

ATOM 314 HA ILE A 18 32.440 27.260 32.130 1.00 0.00 H

ATOM 315 HB ILE A 18 30.270 27.270 30.060 1.00 0.00 H

ATOM 316 1HG1 ILE A 18 29.740 26.560 32.530 1.00 0.00 H

ATOM 317 2HG1 ILE A 18 30.810 25.200 32.190 1.00 0.00 H

ATOM 318 1HG2 ILE A 18 31.220 24.980 29.450 1.00 0.00 H

ATOM 319 2HG2 ILE A 18 32.550 25.330 30.510 1.00 0.00 H

ATOM 320 3HG2 ILE A 18 32.340 26.250 29.070 1.00 0.00 H

ATOM 321 HD1 ILE A 18 29.070 24.490 30.680 1.00 0.00 H

ATOM 322 HD2 ILE A 18 28.560 25.560 30.530 1.00 0.00 H

ATOM 323 HD3 ILE A 18 28.370 24.960 31.560 1.00 0.00 H

ATOM 324 N GLN A 19 32.320 29.500 29.730 1.00 0.00 N

ATOM 325 CA GLN A 19 33.180 30.210 28.810 1.00 0.00 C

ATOM 326 C GLN A 19 34.320 30.950 29.510 1.00 0.00 C

ATOM 327 O GLN A 19 35.430 31.040 29.000 1.00 0.00 O

ATOM 328 CB GLN A 19 32.370 31.220 27.960 1.00 0.00 C

ATOM 329 CG GLN A 19 31.330 30.650 26.960 1.00 0.00 C

ATOM 330 CD GLN A 19 31.700 29.270 26.440 1.00 0.00 C

ATOM 331 NE2 GLN A 19 31.010 28.250 26.960 1.00 0.00 N

ATOM 332 OE1 GLN A 19 32.580 29.110 25.620 1.00 0.00 O

ATOM 333 HN GLN A 19 31.390 29.830 29.780 1.00 0.00 H

ATOM 334 HA GLN A 19 33.690 29.510 28.170 1.00 0.00 H

ATOM 335 HB1 GLN A 19 33.090 31.820 27.390 1.00 0.00 H

ATOM 336 HB2 GLN A 19 31.870 31.910 28.650 1.00 0.00 H

ATOM 337 HG1 GLN A 19 31.260 31.320 26.100 1.00 0.00 H

ATOM 338 HG2 GLN A 19 30.330 30.640 27.400 1.00 0.00 H

ATOM 339 1HE2 GLN A 19 31.280 27.350 26.690 1.00 0.00 H

ATOM 340 2HE2 GLN A 19 30.410 28.430 27.720 1.00 0.00 H

ATOM 341 N ALA A 20 34.090 31.470 30.730 1.00 0.00 N

ATOM 342 CA ALA A 20 35.050 32.200 31.510 1.00 0.00 C

ATOM 343 C ALA A 20 36.130 31.310 32.150 1.00 0.00 C

ATOM 344 O ALA A 20 37.110 31.780 32.640 1.00 0.00 O

ATOM 345 CB ALA A 20 34.310 32.950 32.600 1.00 0.00 C

ATOM 346 HN ALA A 20 33.180 31.450 31.110 1.00 0.00 H

ATOM 347 HA ALA A 20 35.570 32.880 30.890 1.00 0.00 H

ATOM 348 HB1 ALA A 20 35.000 33.560 33.190 1.00 0.00 H

ATOM 349 HB2 ALA A 20 33.820 32.280 33.280 1.00 0.00 H

ATOM 350 HB3 ALA A 20 33.530 33.570 32.170 1.00 0.00 H

ATOM 351 N MET A 21 36.010 29.990 32.060 1.00 0.00 N

ATOM 352 CA MET A 21 36.990 29.000 32.490 1.00 0.00 C

ATOM 353 C MET A 21 37.930 28.560 31.390 1.00 0.00 C

ATOM 354 O MET A 21 38.910 27.910 31.640 1.00 0.00 O

ATOM 355 CB MET A 21 36.290 27.750 33.060 1.00 0.00 C

ATOM 356 CG MET A 21 35.570 27.940 34.390 1.00 0.00 C

ATOM 357 SD MET A 21 36.600 28.400 35.740 1.00 0.00 S

ATOM 358 CE MET A 21 37.410 26.940 35.970 1.00 0.00 C

ATOM 359 HN MET A 21 35.160 29.640 31.720 1.00 0.00 H

ATOM 360 HA MET A 21 37.610 29.430 33.250 1.00 0.00 H

ATOM 361 HB1 MET A 21 37.030 26.950 33.180 1.00 0.00 H

ATOM 362 HB2 MET A 21 35.550 27.420 32.340 1.00 0.00 H

ATOM 363 HG1 MET A 21 35.070 27.030 34.650 1.00 0.00 H

ATOM 364 HG2 MET A 21 34.810 28.680 34.230 1.00 0.00 H

ATOM 365 HE1 MET A 21 37.430 26.620 35.670 1.00 0.00 H

ATOM 366 HE2 MET A 21 37.460 26.450 36.330 1.00 0.00 H

ATOM 367 HE3 MET A 21 37.930 26.800 36.100 1.00 0.00 H

ATOM 368 N ILE A 22 37.700 28.940 30.160 1.00 0.00 N

ATOM 369 CA ILE A 22 38.470 28.500 29.010 1.00 0.00 C

ATOM 370 C ILE A 22 39.430 29.580 28.650 1.00 0.00 C

ATOM 371 O ILE A 22 39.000 30.670 28.360 1.00 0.00 O

ATOM 372 CB ILE A 22 37.570 28.090 27.860 1.00 0.00 C

ATOM 373 CG1 ILE A 22 36.690 26.890 28.260 1.00 0.00 C

ATOM 374 CG2 ILE A 22 38.380 27.780 26.610 1.00 0.00 C

ATOM 375 CD ILE A 22 35.610 26.550 27.390 1.00 0.00 C

ATOM 376 HN ILE A 22 36.910 29.480 29.970 1.00 0.00 H

ATOM 377 HA ILE A 22 39.010 27.620 29.300 1.00 0.00 H

ATOM 378 HB ILE A 22 36.890 28.940 27.660 1.00 0.00 H

ATOM 379 1HG1 ILE A 22 36.310 27.010 29.200 1.00 0.00 H

ATOM 380 2HG1 ILE A 22 37.260 26.070 28.320 1.00 0.00 H

ATOM 381 1HG2 ILE A 22 37.740 27.430 25.790 1.00 0.00 H

ATOM 382 2HG2 ILE A 22 39.080 26.980 26.820 1.00 0.00 H

ATOM 383 3HG2 ILE A 22 38.930 28.640 26.260 1.00 0.00 H

ATOM 384 HD1 ILE A 22 34.990 27.280 27.360 1.00 0.00 H

ATOM 385 HD2 ILE A 22 35.050 25.720 27.680 1.00 0.00 H

ATOM 386 HD3 ILE A 22 35.930 26.350 26.520 1.00 0.00 H

ATOM 387 N PRO A 23 40.750 29.400 28.670 1.00 0.00 N

ATOM 388 CA PRO A 23 41.690 30.500 28.590 1.00 0.00 C

ATOM 389 C PRO A 23 41.930 31.010 27.200 1.00 0.00 C

ATOM 390 O PRO A 23 43.010 31.480 26.920 1.00 0.00 O

ATOM 391 CB PRO A 23 42.950 29.910 29.240 1.00 0.00 C

ATOM 392 CG PRO A 23 42.870 28.450 28.910 1.00 0.00 C

ATOM 393 CD PRO A 23 41.390 28.150 29.030 1.00 0.00 C

ATOM 394 HA PRO A 23 41.320 31.300 29.170 1.00 0.00 H

ATOM 395 HB1 PRO A 23 42.890 30.030 30.320 1.00 0.00 H

ATOM 396 HB2 PRO A 23 43.880 30.360 28.890 1.00 0.00 H

ATOM 397 HG1 PRO A 23 43.210 28.250 27.900 1.00 0.00 H

ATOM 398 HG2 PRO A 23 43.470 27.880 29.600 1.00 0.00 H

ATOM 399 HD1 PRO A 23 41.070 27.350 28.380 1.00 0.00 H

ATOM 400 HD2 PRO A 23 41.160 27.940 30.060 1.00 0.00 H

ATOM 401 N LYS A 24 40.950 30.980 26.300 1.00 0.00 N

ATOM 402 CA LYS A 24 41.040 31.590 24.980 1.00 0.00 C

ATOM 403 C LYS A 24 39.780 32.330 24.640 1.00 0.00 C

ATOM 404 O LYS A 24 38.720 32.020 25.130 1.00 0.00 O

ATOM 405 CB LYS A 24 41.330 30.570 23.890 1.00 0.00 C

ATOM 406 CG LYS A 24 42.730 30.020 23.970 1.00 0.00 C

ATOM 407 CD LYS A 24 43.170 29.350 22.700 1.00 0.00 C

ATOM 408 CE LYS A 24 44.620 28.930 22.680 1.00 0.00 C

ATOM 409 NZ LYS A 24 45.520 30.020 22.970 1.00 0.00 N1+

ATOM 410 HN LYS A 24 40.050 30.680 26.540 1.00 0.00 H

ATOM 411 HA LYS A 24 41.800 32.330 24.960 1.00 0.00 H

ATOM 412 HB1 LYS A 24 41.220 31.040 22.910 1.00 0.00 H

ATOM 413 HB2 LYS A 24 40.630 29.740 23.900 1.00 0.00 H

ATOM 414 HG1 LYS A 24 42.840 29.320 24.810 1.00 0.00 H

ATOM 415 HG2 LYS A 24 43.370 30.860 24.200 1.00 0.00 H

ATOM 416 HD1 LYS A 24 42.980 30.030 21.870 1.00 0.00 H

ATOM 417 HD2 LYS A 24 42.540 28.480 22.490 1.00 0.00 H

ATOM 418 HE1 LYS A 24 44.870 28.570 21.700 1.00 0.00 H

ATOM 419 HE2 LYS A 24 44.800 28.140 23.390 1.00 0.00 H

ATOM 420 HZ1 LYS A 24 46.360 29.910 23.050 1.00 0.00 H

ATOM 421 HZ2 LYS A 24 45.330 30.370 23.640 1.00 0.00 H

ATOM 422 HZ3 LYS A 24 45.520 30.630 22.450 1.00 0.00 H

ATOM 423 N GLY A 25 39.890 33.340 23.760 1.00 0.00 N

ATOM 424 CA GLY A 25 38.780 34.160 23.320 1.00 0.00 C

ATOM 425 C GLY A 25 37.920 33.550 22.270 1.00 0.00 C

ATOM 426 O GLY A 25 38.000 32.370 22.020 1.00 0.00 O

ATOM 427 HN GLY A 25 40.760 33.560 23.350 1.00 0.00 H

ATOM 428 HA1 GLY A 25 39.190 35.070 22.930 1.00 0.00 H

ATOM 429 HA2 GLY A 25 38.160 34.340 24.160 1.00 0.00 H

ATOM 430 N GLY A 26 37.070 34.340 21.620 1.00 0.00 N

ATOM 431 CA GLY A 26 36.220 33.910 20.530 1.00 0.00 C

ATOM 432 C GLY A 26 35.140 32.960 20.930 1.00 0.00 C

ATOM 433 O GLY A 26 34.740 32.120 20.160 1.00 0.00 O

ATOM 434 HN GLY A 26 37.050 35.280 21.840 1.00 0.00 H

ATOM 435 HA1 GLY A 26 36.810 33.440 19.770 1.00 0.00 H

ATOM 436 HA2 GLY A 26 35.720 34.780 20.160 1.00 0.00 H

ATOM 437 N ARG A 27 34.680 33.040 22.180 1.00 0.00 N

ATOM 438 CA ARG A 27 33.690 32.140 22.740 1.00 0.00 C

ATOM 439 C ARG A 27 32.270 32.520 22.380 1.00 0.00 C

ATOM 440 O ARG A 27 31.940 33.680 22.220 1.00 0.00 O

ATOM 441 CB ARG A 27 33.840 32.070 24.280 1.00 0.00 C

ATOM 442 CG ARG A 27 35.250 31.680 24.780 1.00 0.00 C

ATOM 443 CD ARG A 27 35.930 30.500 24.110 1.00 0.00 C

ATOM 444 NE ARG A 27 35.050 29.320 24.250 1.00 0.00 N

ATOM 445 CZ ARG A 27 35.400 28.120 23.880 1.00 0.00 C

ATOM 446 NH1 ARG A 27 34.580 27.130 24.080 1.00 0.00 N1+

ATOM 447 NH2 ARG A 27 36.490 27.860 23.260 1.00 0.00 N

ATOM 448 HN ARG A 27 35.020 33.730 22.780 1.00 0.00 H

ATOM 449 HA ARG A 27 33.870 31.180 22.310 1.00 0.00 H

ATOM 450 HB1 ARG A 27 33.110 31.370 24.690 1.00 0.00 H

ATOM 451 HB2 ARG A 27 33.570 33.050 24.690 1.00 0.00 H

ATOM 452 HG1 ARG A 27 35.200 31.480 25.860 1.00 0.00 H

ATOM 453 HG2 ARG A 27 35.940 32.530 24.690 1.00 0.00 H

ATOM 454 HD1 ARG A 27 36.880 30.330 24.610 1.00 0.00 H

ATOM 455 HD2 ARG A 27 36.100 30.730 23.070 1.00 0.00 H

ATOM 456 HE ARG A 27 34.180 29.390 24.690 1.00 0.00 H

ATOM 457 1HH1 ARG A 27 33.700 27.300 24.470 1.00 0.00 H

ATOM 458 2HH1 ARG A 27 34.700 26.360 23.490 1.00 0.00 H

ATOM 459 1HH2 ARG A 27 37.110 28.580 23.050 1.00 0.00 H

ATOM 460 2HH2 ARG A 27 36.820 26.940 23.160 1.00 0.00 H

ATOM 461 N MET A 28 31.400 31.520 22.200 1.00 0.00 N

ATOM 462 CA MET A 28 30.000 31.650 21.880 1.00 0.00 C

ATOM 463 C MET A 28 29.150 32.490 22.820 1.00 0.00 C

ATOM 464 O MET A 28 29.410 32.550 24.020 1.00 0.00 O

ATOM 465 CB MET A 28 29.350 30.230 21.680 1.00 0.00 C

ATOM 466 CG MET A 28 29.360 29.320 22.910 1.00 0.00 C

ATOM 467 SD MET A 28 28.740 27.670 22.610 1.00 0.00 S

ATOM 468 CE MET A 28 28.530 27.170 24.270 1.00 0.00 C

ATOM 469 HN MET A 28 31.730 30.590 22.270 1.00 0.00 H

ATOM 470 HA MET A 28 29.950 32.150 20.930 1.00 0.00 H

ATOM 471 HB1 MET A 28 29.910 29.720 20.900 1.00 0.00 H

ATOM 472 HB2 MET A 28 28.330 30.350 21.290 1.00 0.00 H

ATOM 473 HG1 MET A 28 28.790 29.770 23.710 1.00 0.00 H

ATOM 474 HG2 MET A 28 30.360 29.250 23.310 1.00 0.00 H

ATOM 475 HE1 MET A 28 28.310 26.920 24.600 1.00 0.00 H

ATOM 476 HE2 MET A 28 28.500 27.290 24.610 1.00 0.00 H

ATOM 477 HE3 MET A 28 28.680 26.960 24.680 1.00 0.00 H

ATOM 478 N LEU A 29 28.120 33.190 22.320 1.00 0.00 N

ATOM 479 CA LEU A 29 27.320 34.120 23.070 1.00 0.00 C

ATOM 480 C LEU A 29 26.250 33.420 23.900 1.00 0.00 C

ATOM 481 O LEU A 29 25.550 32.600 23.380 1.00 0.00 O

ATOM 482 CB LEU A 29 26.690 35.160 22.110 1.00 0.00 C

ATOM 483 CG LEU A 29 27.680 36.030 21.350 1.00 0.00 C

ATOM 484 CD1 LEU A 29 27.050 36.860 20.310 1.00 0.00 C

ATOM 485 CD2 LEU A 29 28.420 36.900 22.270 1.00 0.00 C

ATOM 486 HN LEU A 29 27.910 33.120 21.360 1.00 0.00 H

ATOM 487 HA LEU A 29 27.970 34.660 23.720 1.00 0.00 H

ATOM 488 HB1 LEU A 29 26.060 35.830 22.680 1.00 0.00 H

ATOM 489 HB2 LEU A 29 26.080 34.660 21.370 1.00 0.00 H

ATOM 490 HG LEU A 29 28.360 35.380 20.880 1.00 0.00 H

ATOM 491 1HD1 LEU A 29 26.330 37.440 20.720 1.00 0.00 H

ATOM 492 2HD1 LEU A 29 26.590 36.260 19.650 1.00 0.00 H

ATOM 493 3HD1 LEU A 29 27.770 37.480 19.800 1.00 0.00 H

ATOM 494 1HD2 LEU A 29 29.090 37.570 21.740 1.00 0.00 H

ATOM 495 2HD2 LEU A 29 28.960 36.310 22.960 1.00 0.00 H

ATOM 496 3HD2 LEU A 29 27.760 37.520 22.820 1.00 0.00 H

ATOM 497 N PRO A 30 26.070 33.660 25.200 1.00 0.00 N

ATOM 498 CA PRO A 30 25.440 32.700 26.090 1.00 0.00 C

ATOM 499 C PRO A 30 23.940 32.630 25.950 1.00 0.00 C

ATOM 500 O PRO A 30 23.370 31.550 25.910 1.00 0.00 O

ATOM 501 CB PRO A 30 25.870 33.160 27.490 1.00 0.00 C

ATOM 502 CG PRO A 30 26.230 34.620 27.330 1.00 0.00 C

ATOM 503 CD PRO A 30 26.750 34.720 25.940 1.00 0.00 C

ATOM 504 HA PRO A 30 25.820 31.730 25.850 1.00 0.00 H

ATOM 505 HB1 PRO A 30 26.750 32.600 27.770 1.00 0.00 H

ATOM 506 HB2 PRO A 30 25.110 33.000 28.240 1.00 0.00 H

ATOM 507 HG1 PRO A 30 25.360 35.220 27.440 1.00 0.00 H

ATOM 508 HG2 PRO A 30 26.950 34.910 28.040 1.00 0.00 H

ATOM 509 HD1 PRO A 30 26.560 35.690 25.520 1.00 0.00 H

ATOM 510 HD2 PRO A 30 27.790 34.540 25.920 1.00 0.00 H

ATOM 511 N GLN A 31 23.230 33.750 25.860 1.00 0.00 N

ATOM 512 CA GLN A 31 21.800 33.810 25.670 1.00 0.00 C

ATOM 513 C GLN A 31 21.370 33.320 24.300 1.00 0.00 C

ATOM 514 O GLN A 31 20.370 32.630 24.160 1.00 0.00 O

ATOM 515 CB GLN A 31 21.210 35.220 25.910 1.00 0.00 C

ATOM 516 CG GLN A 31 21.610 36.390 24.990 1.00 0.00 C

ATOM 517 CD GLN A 31 23.090 36.630 24.810 1.00 0.00 C

ATOM 518 NE2 GLN A 31 23.690 37.330 25.760 1.00 0.00 N

ATOM 519 OE1 GLN A 31 23.720 36.120 23.890 1.00 0.00 O

ATOM 520 HN GLN A 31 23.710 34.610 25.900 1.00 0.00 H

ATOM 521 HA GLN A 31 21.320 33.170 26.390 1.00 0.00 H

ATOM 522 HB1 GLN A 31 21.470 35.510 26.930 1.00 0.00 H

ATOM 523 HB2 GLN A 31 20.110 35.150 25.890 1.00 0.00 H

ATOM 524 HG1 GLN A 31 21.130 37.300 25.320 1.00 0.00 H

ATOM 525 HG2 GLN A 31 21.190 36.210 23.990 1.00 0.00 H

ATOM 526 1HE2 GLN A 31 24.610 37.610 25.620 1.00 0.00 H

ATOM 527 2HE2 GLN A 31 23.150 37.650 26.520 1.00 0.00 H

ATOM 528 N LEU A 32 22.180 33.600 23.260 1.00 0.00 N

ATOM 529 CA LEU A 32 21.970 33.150 21.910 1.00 0.00 C

ATOM 530 C LEU A 32 22.000 31.650 21.790 1.00 0.00 C

ATOM 531 O LEU A 32 21.120 31.060 21.190 1.00 0.00 O

ATOM 532 CB LEU A 32 23.050 33.770 21.020 1.00 0.00 C

ATOM 533 CG LEU A 32 22.960 33.480 19.520 1.00 0.00 C

ATOM 534 CD1 LEU A 32 22.210 34.080 18.830 1.00 0.00 C

ATOM 535 CD2 LEU A 32 23.960 33.650 18.840 1.00 0.00 C

ATOM 536 HN LEU A 32 22.940 34.200 23.400 1.00 0.00 H

ATOM 537 HA LEU A 32 21.010 33.500 21.580 1.00 0.00 H

ATOM 538 HB1 LEU A 32 24.040 33.450 21.320 1.00 0.00 H

ATOM 539 HB2 LEU A 32 23.030 34.840 21.150 1.00 0.00 H

ATOM 540 HG LEU A 32 22.770 32.710 19.390 1.00 0.00 H

ATOM 541 1HD1 LEU A 32 22.100 33.920 18.050 1.00 0.00 H

ATOM 542 2HD1 LEU A 32 22.320 34.610 18.880 1.00 0.00 H

ATOM 543 3HD1 LEU A 32 21.610 34.170 19.060 1.00 0.00 H

ATOM 544 1HD2 LEU A 32 23.970 33.500 17.970 1.00 0.00 H

ATOM 545 2HD2 LEU A 32 24.420 33.300 19.150 1.00 0.00 H

ATOM 546 3HD2 LEU A 32 24.210 34.260 18.830 1.00 0.00 H

ATOM 547 N VAL A 33 22.960 30.950 22.420 1.00 0.00 N

ATOM 548 CA VAL A 33 23.090 29.520 22.410 1.00 0.00 C

ATOM 549 C VAL A 33 21.910 28.830 23.040 1.00 0.00 C

ATOM 550 O VAL A 33 21.360 27.870 22.520 1.00 0.00 O

ATOM 551 CB VAL A 33 24.380 29.090 23.060 1.00 0.00 C

ATOM 552 CG1 VAL A 33 24.470 27.580 23.230 1.00 0.00 C

ATOM 553 CG2 VAL A 33 25.520 29.560 22.170 1.00 0.00 C

ATOM 554 HN VAL A 33 23.660 31.450 22.920 1.00 0.00 H

ATOM 555 HA VAL A 33 23.120 29.210 21.390 1.00 0.00 H

ATOM 556 HB VAL A 33 24.510 29.530 24.040 1.00 0.00 H

ATOM 557 1HG1 VAL A 33 24.680 27.270 23.830 1.00 0.00 H

ATOM 558 2HG1 VAL A 33 24.920 27.120 22.860 1.00 0.00 H

ATOM 559 3HG1 VAL A 33 23.960 27.180 23.170 1.00 0.00 H

ATOM 560 1HG2 VAL A 33 25.690 30.130 22.060 1.00 0.00 H

ATOM 561 2HG2 VAL A 33 25.640 29.450 21.560 1.00 0.00 H

ATOM 562 3HG2 VAL A 33 26.130 29.480 22.250 1.00 0.00 H

ATOM 563 N CYS A 34 21.400 29.350 24.170 1.00 0.00 N

ATOM 564 CA CYS A 34 20.220 28.890 24.820 1.00 0.00 C

ATOM 565 C CYS A 34 18.930 29.070 24.000 1.00 0.00 C

ATOM 566 O CYS A 34 18.050 28.210 23.990 1.00 0.00 O

ATOM 567 CB CYS A 34 20.150 29.560 26.220 1.00 0.00 C

ATOM 568 SG CYS A 34 18.910 28.820 27.300 1.00 0.00 S

ATOM 569 HN CYS A 34 21.850 30.130 24.580 1.00 0.00 H

ATOM 570 HA CYS A 34 20.310 27.830 24.960 1.00 0.00 H

ATOM 571 HB1 CYS A 34 19.950 30.630 26.100 1.00 0.00 H

ATOM 572 HB2 CYS A 34 21.130 29.470 26.710 1.00 0.00 H

ATOM 573 N ARG A 35 18.830 30.200 23.250 1.00 0.00 N

ATOM 574 CA ARG A 35 17.800 30.400 22.230 1.00 0.00 C

ATOM 575 C ARG A 35 17.890 29.490 21.040 1.00 0.00 C

ATOM 576 O ARG A 35 16.870 28.990 20.600 1.00 0.00 O

ATOM 577 CB ARG A 35 17.790 31.840 21.730 1.00 0.00 C

ATOM 578 CG ARG A 35 17.230 32.830 22.750 1.00 0.00 C

ATOM 579 CD ARG A 35 17.430 34.270 22.410 1.00 0.00 C

ATOM 580 NE ARG A 35 16.640 35.060 23.380 1.00 0.00 N

ATOM 581 CZ ARG A 35 16.990 36.140 23.970 1.00 0.00 C

ATOM 582 NH1 ARG A 35 16.170 36.650 24.810 1.00 0.00 N1+

ATOM 583 NH2 ARG A 35 18.070 36.750 23.720 1.00 0.00 N

ATOM 584 HN ARG A 35 19.520 30.910 23.330 1.00 0.00 H

ATOM 585 HA ARG A 35 16.850 30.210 22.690 1.00 0.00 H

ATOM 586 HB1 ARG A 35 17.200 31.930 20.810 1.00 0.00 H

ATOM 587 HB2 ARG A 35 18.800 32.140 21.450 1.00 0.00 H

ATOM 588 HG1 ARG A 35 17.670 32.650 23.730 1.00 0.00 H

ATOM 589 HG2 ARG A 35 16.170 32.630 22.890 1.00 0.00 H

ATOM 590 HD1 ARG A 35 17.060 34.480 21.420 1.00 0.00 H

ATOM 591 HD2 ARG A 35 18.490 34.520 22.470 1.00 0.00 H

ATOM 592 HE ARG A 35 15.720 34.800 23.550 1.00 0.00 H

ATOM 593 1HH1 ARG A 35 15.310 36.220 24.900 1.00 0.00 H

ATOM 594 2HH1 ARG A 35 16.360 37.490 25.150 1.00 0.00 H

ATOM 595 1HH2 ARG A 35 18.590 36.520 22.940 1.00 0.00 H

ATOM 596 2HH2 ARG A 35 18.220 37.620 24.150 1.00 0.00 H

ATOM 597 N LEU A 36 19.090 29.180 20.510 1.00 0.00 N

ATOM 598 CA LEU A 36 19.260 28.260 19.400 1.00 0.00 C

ATOM 599 C LEU A 36 18.810 26.840 19.690 1.00 0.00 C

ATOM 600 O LEU A 36 18.270 26.160 18.830 1.00 0.00 O

ATOM 601 CB LEU A 36 20.720 28.260 18.900 1.00 0.00 C

ATOM 602 CG LEU A 36 21.140 29.520 18.120 1.00 0.00 C

ATOM 603 CD1 LEU A 36 22.600 29.530 17.860 1.00 0.00 C

ATOM 604 CD2 LEU A 36 20.380 29.680 16.840 1.00 0.00 C

ATOM 605 HN LEU A 36 19.910 29.590 20.870 1.00 0.00 H

ATOM 606 HA LEU A 36 18.590 28.570 18.630 1.00 0.00 H

ATOM 607 HB1 LEU A 36 20.890 27.440 18.210 1.00 0.00 H

ATOM 608 HB2 LEU A 36 21.400 28.090 19.740 1.00 0.00 H

ATOM 609 HG LEU A 36 20.930 30.380 18.750 1.00 0.00 H

ATOM 610 1HD1 LEU A 36 23.050 29.290 18.200 1.00 0.00 H

ATOM 611 2HD1 LEU A 36 23.000 30.040 17.770 1.00 0.00 H

ATOM 612 3HD1 LEU A 36 22.890 29.310 17.330 1.00 0.00 H

ATOM 613 1HD2 LEU A 36 19.380 29.810 16.970 1.00 0.00 H

ATOM 614 2HD2 LEU A 36 20.510 28.880 16.220 1.00 0.00 H

ATOM 615 3HD2 LEU A 36 20.700 30.480 16.270 1.00 0.00 H

ATOM 616 N VAL A 37 18.980 26.330 20.910 1.00 0.00 N

ATOM 617 CA VAL A 37 18.540 24.980 21.280 1.00 0.00 C

ATOM 618 C VAL A 37 17.140 25.010 21.890 1.00 0.00 C

ATOM 619 O VAL A 37 16.630 24.040 22.390 1.00 0.00 O

ATOM 620 CB VAL A 37 19.540 24.270 22.190 1.00 0.00 C

ATOM 621 CG1 VAL A 37 20.890 24.160 21.500 1.00 0.00 C

ATOM 622 CG2 VAL A 37 19.680 24.950 23.540 1.00 0.00 C

ATOM 623 HN VAL A 37 19.500 26.840 21.560 1.00 0.00 H

ATOM 624 HA VAL A 37 18.450 24.360 20.410 1.00 0.00 H

ATOM 625 HB VAL A 37 19.210 23.240 22.350 1.00 0.00 H

ATOM 626 1HG1 VAL A 37 21.570 23.600 22.110 1.00 0.00 H

ATOM 627 2HG1 VAL A 37 21.330 25.130 21.350 1.00 0.00 H

ATOM 628 3HG1 VAL A 37 20.820 23.670 20.560 1.00 0.00 H

ATOM 629 1HG2 VAL A 37 19.670 24.620 24.180 1.00 0.00 H

ATOM 630 2HG2 VAL A 37 19.220 25.460 23.810 1.00 0.00 H

ATOM 631 3HG2 VAL A 37 20.260 25.250 23.640 1.00 0.00 H

ATOM 632 N LEU A 38 16.460 26.160 21.810 1.00 0.00 N

ATOM 633 CA LEU A 38 15.050 26.290 22.130 1.00 0.00 C

ATOM 634 C LEU A 38 14.740 26.200 23.630 1.00 0.00 C

ATOM 635 O LEU A 38 13.670 25.830 24.030 1.00 0.00 O

ATOM 636 CB LEU A 38 14.130 25.420 21.230 1.00 0.00 C

ATOM 637 CG LEU A 38 14.310 25.610 19.730 1.00 0.00 C

ATOM 638 CD1 LEU A 38 13.560 24.520 18.970 1.00 0.00 C

ATOM 639 CD2 LEU A 38 13.820 26.940 19.270 1.00 0.00 C

ATOM 640 HN LEU A 38 16.880 26.940 21.360 1.00 0.00 H

ATOM 641 HA LEU A 38 14.820 27.300 21.870 1.00 0.00 H

ATOM 642 HB1 LEU A 38 13.100 25.660 21.480 1.00 0.00 H

ATOM 643 HB2 LEU A 38 14.280 24.360 21.460 1.00 0.00 H

ATOM 644 HG LEU A 38 15.360 25.540 19.500 1.00 0.00 H

ATOM 645 1HD1 LEU A 38 13.680 24.630 17.920 1.00 0.00 H

ATOM 646 2HD1 LEU A 38 12.520 24.540 19.150 1.00 0.00 H

ATOM 647 3HD1 LEU A 38 13.900 23.570 19.250 1.00 0.00 H

ATOM 648 1HD2 LEU A 38 13.720 27.030 18.450 1.00 0.00 H

ATOM 649 2HD2 LEU A 38 14.270 27.620 19.470 1.00 0.00 H

ATOM 650 3HD2 LEU A 38 13.070 27.180 19.530 1.00 0.00 H

ATOM 651 N ARG A 39 15.670 26.590 24.530 1.00 0.00 N

ATOM 652 CA ARG A 39 15.490 26.630 25.960 1.00 0.00 C

ATOM 653 C ARG A 39 15.290 28.050 26.460 1.00 0.00 C

ATOM 654 O ARG A 39 15.200 28.290 27.640 1.00 0.00 O

ATOM 655 CB ARG A 39 16.670 25.970 26.690 1.00 0.00 C

ATOM 656 CG ARG A 39 16.860 24.480 26.370 1.00 0.00 C

ATOM 657 CD ARG A 39 15.750 23.600 26.880 1.00 0.00 C

ATOM 658 NE ARG A 39 16.030 22.210 26.490 1.00 0.00 N

ATOM 659 CZ ARG A 39 15.130 21.270 26.470 1.00 0.00 C

ATOM 660 NH1 ARG A 39 15.390 20.130 25.930 1.00 0.00 N1+

ATOM 661 NH2 ARG A 39 13.940 21.430 26.970 1.00 0.00 N

ATOM 662 HN ARG A 39 16.570 26.840 24.220 1.00 0.00 H

ATOM 663 HA ARG A 39 14.600 26.080 26.210 1.00 0.00 H

ATOM 664 HB1 ARG A 39 16.630 26.130 27.770 1.00 0.00 H

ATOM 665 HB2 ARG A 39 17.570 26.460 26.310 1.00 0.00 H

ATOM 666 HG1 ARG A 39 17.800 24.120 26.800 1.00 0.00 H

ATOM 667 HG2 ARG A 39 16.930 24.350 25.290 1.00 0.00 H

ATOM 668 HD1 ARG A 39 14.830 23.950 26.440 1.00 0.00 H

ATOM 669 HD2 ARG A 39 15.680 23.660 27.960 1.00 0.00 H

ATOM 670 HE ARG A 39 16.900 21.960 26.140 1.00 0.00 H

ATOM 671 1HH1 ARG A 39 16.280 19.950 25.650 1.00 0.00 H

ATOM 672 2HH1 ARG A 39 14.720 19.420 25.930 1.00 0.00 H

ATOM 673 1HH2 ARG A 39 13.790 22.180 27.580 1.00 0.00 H

ATOM 674 2HH2 ARG A 39 13.330 20.670 26.980 1.00 0.00 H

ATOM 675 N CYS A 40 15.200 28.980 25.530 1.00 0.00 N

ATOM 676 CA CYS A 40 14.930 30.370 25.790 1.00 0.00 C

ATOM 677 C CYS A 40 14.230 30.900 24.550 1.00 0.00 C

ATOM 678 O CYS A 40 14.460 30.420 23.460 1.00 0.00 O

ATOM 679 CB CYS A 40 16.230 31.160 26.140 1.00 0.00 C

ATOM 680 SG CYS A 40 16.040 32.920 26.500 1.00 0.00 S

ATOM 681 HN CYS A 40 15.320 28.720 24.590 1.00 0.00 H

ATOM 682 HA CYS A 40 14.220 30.430 26.590 1.00 0.00 H

ATOM 683 HB1 CYS A 40 16.960 31.000 25.360 1.00 0.00 H

ATOM 684 HB2 CYS A 40 16.650 30.670 27.020 1.00 0.00 H

ATOM 685 N SER A 41 13.380 31.920 24.690 1.00 0.00 N

ATOM 686 CA SER A 41 12.680 32.630 23.640 1.00 0.00 C

ATOM 687 C SER A 41 13.330 33.950 23.380 1.00 0.00 C

ATOM 688 CB SER A 41 11.220 32.900 23.960 1.00 0.00 C

ATOM 689 OG SER A 41 10.460 31.750 23.900 1.00 0.00 O

ATOM 690 OT1 SER A 41 13.780 34.690 24.060 1.00 0.00 O

ATOM 691 OT2 SER A 41 13.400 34.260 22.460 1.00 0.00 O

ATOM 692 HN SER A 41 13.180 32.230 25.600 1.00 0.00 H

ATOM 693 HA SER A 41 12.730 32.090 22.770 1.00 0.00 H

ATOM 694 HB1 SER A 41 10.770 33.600 23.240 1.00 0.00 H

ATOM 695 HB2 SER A 41 11.110 33.340 24.930 1.00 0.00 H

ATOM 696 HG1 SER A 41 10.750 31.150 24.530 1.00 0.00 H

END

# **ModelArchive (**[**https://modelarchive.org**](https://modelarchive.org)**) > Procedures & Data**

**FTIR Data of SMB and selectively ^13^C labeled SMB Peptides in Synthetic Surfactant Lipid Multilayer Lipid-Peptide Films**

Unlabeled SMB Peptide Spectrum

Wavelength Absorbance

1720.1924 -9.9000e-4

1718.2639 -9.9000e-4

1716.3355 -9.9000e-4

1714.4070 -9.9000e-4

1712.4785 -9.9000e-4

1710.5501 2.2000e-4

1708.6216 2.7500e-3

1706.6931 6.9400e-3

1704.7647 0.0125

1702.8362 0.0187

1700.9077 0.0250

1698.9793 0.0311

1697.0508 0.0366

1695.1223 0.0419

1693.1939 0.0472

1691.2654 0.0532

1689.3369 0.0607

1687.4085 0.0693

1685.4800 0.0781

1683.5515 0.0863

1681.6231 0.0939

1679.6946 0.1012

1677.7661 0.1083

1675.8377 0.1159

1673.9092 0.1248

1671.9807 0.1347

1670.0523 0.1450

1668.1238 0.1561

1666.1953 0.1682

1664.2669 0.1810

1662.3384 0.1938

1660.4099 0.2064

1658.4815 0.2172

1656.5530 0.2236

1654.6245 0.2248

1652.6960 0.2210

1650.7676 0.2123

1648.8391 0.1993

1646.9106 0.1839

1644.9822 0.1687

1643.0537 0.1546

1641.1252 0.1419

1639.1968 0.1308

1637.2683 0.1211

1635.3398 0.1124

1633.4114 0.1044

1631.4829 0.0972

1629.5544 0.0911

1627.6260 0.0860

1625.6975 0.0813

1623.7690 0.0766

1621.8406 0.0718

1619.9121 0.0665

1617.9836 0.0607

1616.0552 0.0544

1614.1267 0.0476

1612.1982 0.0404

1610.2698 0.0333

1608.3413 0.0270

1606.4128 0.0218

1604.4844 0.0176

1602.5559 0.0144

1600.6274 0.0121

1598.6990 0.0103

1596.7705 8.8400e-3

1594.8420 7.2800e-3

1592.9136 5.5400e-3

1590.9851 3.6200e-3

1589.0566 1.6900e-3

1587.1282 -2.0000e-5

1585.1997 -2.0000e-5

1583.2712 -2.0000e-5

1581.3428 -2.0000e-5

1579.4143 -2.0000e-5

N-terminal ^13^C labeled SMB Peptide Spectrum

Wavelength Absorbance

1720.1924 0.0000

1718.2639 6.1000e-4

1716.3355 2.1600e-3

1714.4070 2.7100e-3

1712.4785 3.2900e-3

1710.5501 4.4700e-3

1708.6216 6.1300e-3

1706.6931 7.0200e-3

1704.7647 8.6600e-3

1702.8362 0.0104

1700.9077 0.0127

1698.9793 0.0157

1697.0508 0.0206

1695.1223 0.0271

1693.1939 0.0352

1691.2654 0.0432

1689.3369 0.0509

1687.4085 0.0578

1685.4800 0.0650

1683.5515 0.0727

1681.6231 0.0815

1679.6946 0.0908

1677.7661 0.1004

1675.8377 0.1100

1673.9092 0.1199

1671.9807 0.1302

1670.0523 0.1414

1668.1238 0.1535

1666.1953 0.1660

1664.2669 0.1781

1662.3384 0.1885

1660.4099 0.1963

1658.4815 0.2007

1656.5530 0.2012

1654.6245 0.1977

1652.6960 0.1914

1650.7676 0.1829

1648.8391 0.1733

1646.9106 0.1628

1644.9822 0.1523

1643.0537 0.1417

1641.1252 0.1319

1639.1968 0.1235

1637.2683 0.1182

1635.3398 0.1146

1633.4114 0.1121

1631.4829 0.1102

1629.5544 0.1090

1627.6260 0.1077

1625.6975 0.1072

1623.7690 0.1060

1621.8406 0.1045

1619.9121 0.1021

1617.9836 0.0988

1616.0552 0.0933

1614.1267 0.0862

1612.1982 0.0766

1610.2698 0.0660

1608.3413 0.0554

1606.4128 0.0461

1604.4844 0.0376

1602.5559 0.0310

1600.6274 0.0255

1598.6990 0.0215

1596.7705 0.0180

1594.8420 0.0152

1592.9136 0.0125

1590.9851 9.7800e-3

1589.0566 6.4000e-3

1587.1282 3.5900e-3

1585.1997 1.6500e-3

1583.2712 7.5000e-4

1581.3428 0.0000

1579.4143 -1.0000e-4

Unlabeled SMB Peptide Spectrum

Wavelength Absorbance

1720.1924 5.1000e-4

1718.2639 5.1000e-4

1716.3355 5.1000e-4

1714.4070 5.1000e-4

1712.4785 5.1000e-4

1710.5501 1.7200e-3

1708.6216 4.2500e-3

1706.6931 8.4400e-3

1704.7647 0.0140

1702.8362 0.0202

1700.9077 0.0265

1698.9793 0.0326

1697.0508 0.0381

1695.1223 0.0434

1693.1939 0.0487

1691.2654 0.0547

1689.3369 0.0622

1687.4085 0.0708

1685.4800 0.0796

1683.5515 0.0878

1681.6231 0.0954

1679.6946 0.1027

1677.7661 0.1098

1675.8377 0.1174

1673.9092 0.1263

1671.9807 0.1362

1670.0523 0.1465

1668.1238 0.1576

1666.1953 0.1697

1664.2669 0.1825

1662.3384 0.1953

1660.4099 0.2079

1658.4815 0.2187

1656.5530 0.2251

1654.6245 0.2263

1652.6960 0.2225

1650.7676 0.2138

1648.8391 0.2008

1646.9106 0.1854

1644.9822 0.1701

1643.0537 0.1561

1641.1252 0.1434

1639.1968 0.1323

1637.2683 0.1226

1635.3398 0.1139

1633.4114 0.1059

1631.4829 0.0987

1629.5544 0.0926

1627.6260 0.0875

1625.6975 0.0828

1623.7690 0.0781

1621.8406 0.0733

1619.9121 0.0680

1617.9836 0.0622

1616.0552 0.0559

1614.1267 0.0491

1612.1982 0.0419

1610.2698 0.0348

1608.3413 0.0285

1606.4128 0.0233

1604.4844 0.0191

1602.5559 0.0159

1600.6274 0.0136

1598.6990 0.0119

1596.7705 0.0103

1594.8420 8.7800e-3

1592.9136 7.0400e-3

1590.9851 5.1200e-3

1589.0566 3.1900e-3

1587.1282 1.4800e-3

1585.1997 1.4800e-3

1583.2712 1.4800e-3

1581.3428 1.4800e-3

1579.4143 1.4800e-3

C-terminal ^13^C labeled SMB Peptide Spectrum

Wavelength Absorbance

1720.1924 -7.1000e-4

1718.2639 0.0000

1716.3355 2.6000e-4

1714.4070 4.2000e-4

1712.4785 9.8000e-4

1710.5501 2.0000e-3

1708.6216 3.8800e-3

1706.6931 6.2800e-3

1704.7647 0.0100

1702.8362 0.0151

1700.9077 0.0208

1698.9793 0.0255

1697.0508 0.0291

1695.1223 0.0326

1693.1939 0.0369

1691.2654 0.0419

1689.3369 0.0486

1687.4085 0.0576

1685.4800 0.0673

1683.5515 0.0759

1681.6231 0.0833

1679.6946 0.0898

1677.7661 0.0963

1675.8377 0.1039

1673.9092 0.1130

1671.9807 0.1229

1670.0523 0.1328

1668.1238 0.1430

1666.1953 0.1536

1664.2669 0.1642

1662.3384 0.1749

1660.4099 0.1857

1658.4815 0.1958

1656.5530 0.2029

1654.6245 0.2058

1652.6960 0.2043

1650.7676 0.1982

1648.8391 0.1877

1646.9106 0.1745

1644.9822 0.1610

1643.0537 0.1492

1641.1252 0.1394

1639.1968 0.1314

1637.2683 0.1251

1635.3398 0.1197

1633.4114 0.1149

1631.4829 0.1108

1629.5544 0.1078

1627.6260 0.1060

1625.6975 0.1049

1623.7690 0.1036

1621.8406 0.1016

1619.9121 0.0978

1617.9836 0.0921

1616.0552 0.0849

1614.1267 0.0763

1612.1982 0.0659

1610.2698 0.0550

1608.3413 0.0450

1606.4128 0.0367

1604.4844 0.0298

1602.5559 0.0244

1600.6274 0.0200

1598.6990 0.0166

1596.7705 0.0136

1594.8420 0.0109

1592.9136 8.1600e-3

1590.9851 5.6100e-3

1589.0566 3.3900e-3

1587.1282 2.3300e-3

1585.1997 1.4500e-3

1583.2712 7.1000e-4

1581.3428 6.0000e-5

1579.4143 -6.6000e-4

FTIR Spectral Smoothing – Signal Averaged data (256 scan averaged) compared with smoothed Buker OPUS Software (Version 6.5) Smooth utility. Scan averaged IR spectra black line, smoothed IR spectra red line. Figure A, unlabeled SMB peptide. Figure B, N-terminal ^13^ C labeled SMB peptide. Figure C, C-terminal ^13^ C labeled SMB peptide.

**A**


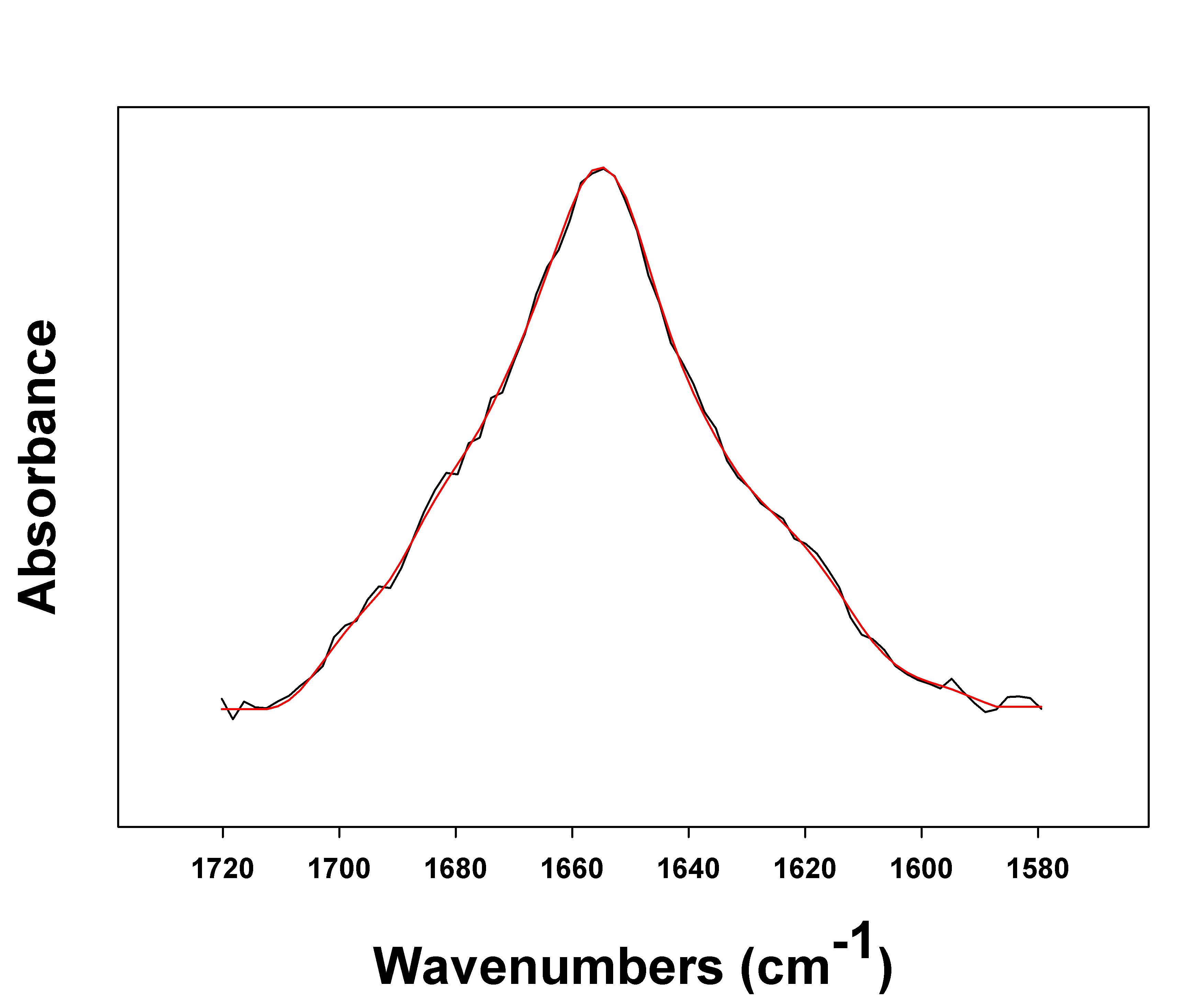

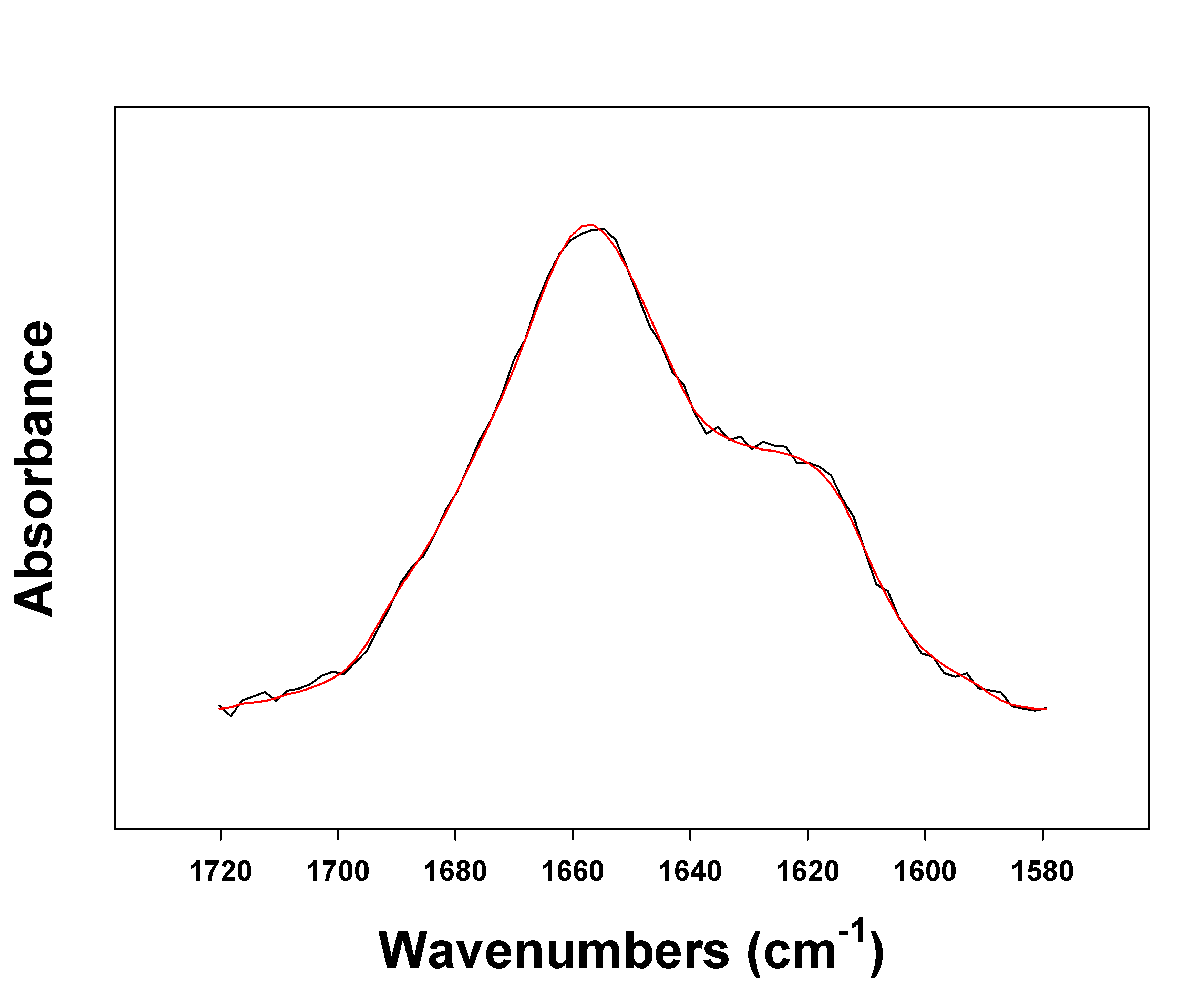


**
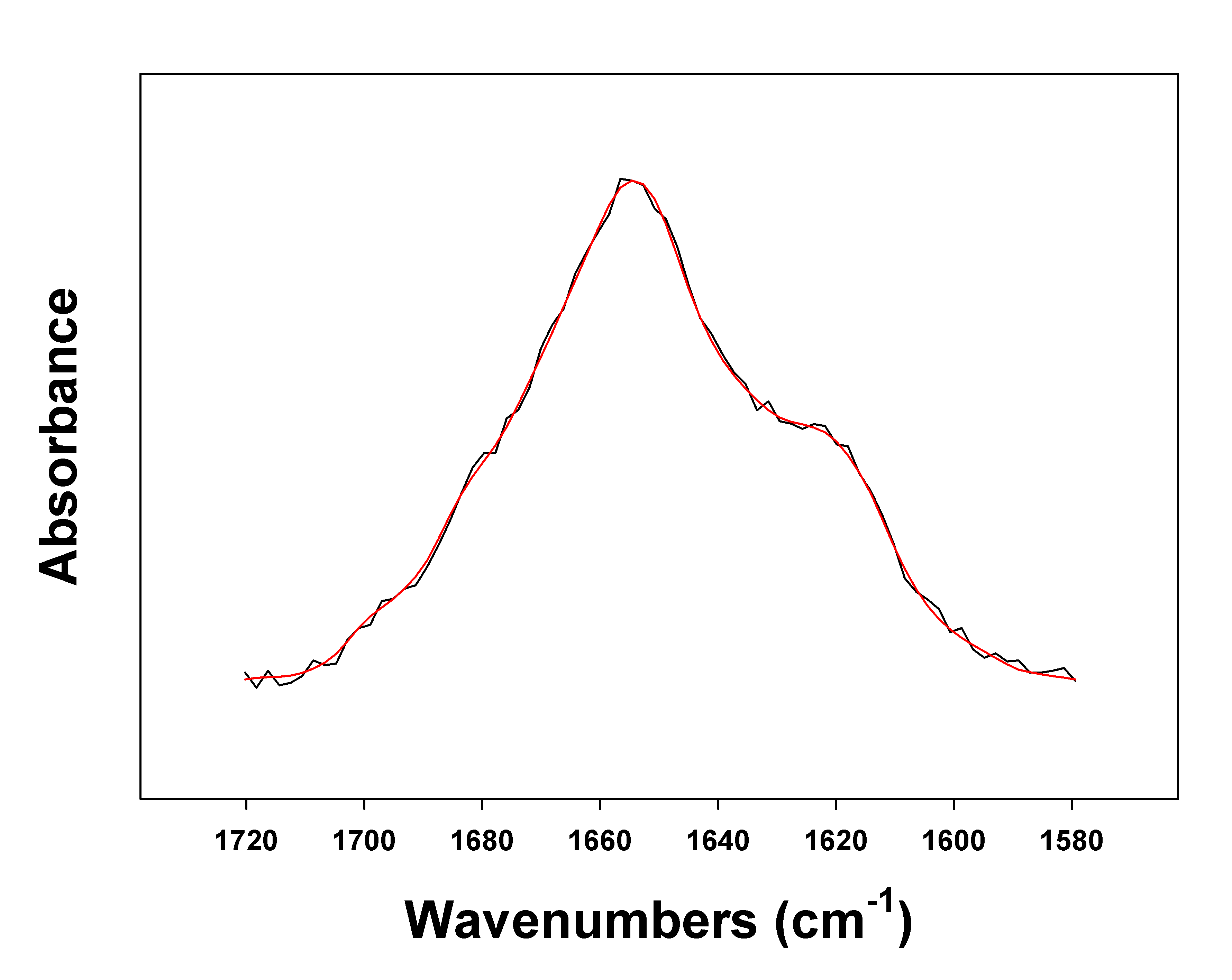
**

**C**

**Molecular Dynamics Refinement of SMB Peptide Structure based on MS and FTIR Residue Specific Experimental Data as constraints using I-Tasser structure prediction as an initial conformation before molecular dynamics refinement in simulated surfactant lipids bilayer**

Molecular simulations were run on a custom desk top computer cluster fitted with a 3.10 GHz Intel Xeon E5-2687W CPU, 256 GB of RAM, SSD Storage and a 2080Ti NVIDIA GPU board (<https://responsiveweb.io/>). The custom system was configured with ubuntu version 22**.**

MD simulations were carried out using the Charmm 36m all atom force field implementation for lipids and proteins in the [Gromacs](http://manual.gromacs.org/documentation/5.1.1/user-guide/environment-variables.html) (Version 2020.3) environment (http://www.gromacs.org). The system was first minimized using a steepest descent strategy followed by a six-step equilibration process at 311^o^K for a total of 500 ns. This included both NVT (constant number, volume, temperature) and NPT (constant number, pressure, temperature) equilibration phases to allow water molecules to reorient around the lipid headgroups and any exposed parts of the peptide, as well as permitting lipids to optimize their orientation around the peptide. Equilibration protocols employed a PME (Particle Mesh Ewald) strategy for Coulombic long-range interactions and Berendsen temperature coupling. A Berendsen strategy was also used for pressure coupling in a semi-isotropic mode to emulate bilayer motion. After equilibration, the system was subjected to a dynamics production run at the same temperature using the Nose-Hoover protocol and pressure (Parrinello-Rahman) values used in the pre-run steps The Verlet cut-off scheme was employed for all minimization, equilibration, and production steps. Detailed protocols and parameter files for this type of membrane simulation are available from the [Charmm-GUI website](http://www.charmm-gui.org/): (http://www.charmm-gui.org). The output of the production run simulations was analyzed with the Gromacs suite of analysis tools.

The structural quality of the molecular dynamics refined structures was analyzed by PROCHECK (Laskowski et al., 1993, 1996) generated with PDBsum (https://ebi.ac.uk). Molecular graphics were rendered using Pymol Version 2.2.3.

Using the combination of the disulfide linkages and ^13^C measurements constraints followed by molecular dynamics refinement, 10 molecular models representing possible conformations of SMB in simulated surfactant lipid bilayers were generated. The lowest energy conformer is shown in as a ribbon-cartoon representation of the lowest energy conformer of the peptide in the surfactant multilayer environment. Geometry parameters of the final models were evaluated using PROCHECK (Laskowski et. al., 1993, 1996) to generate a Ramachandran plot that confirms the backbone torsion angles for the well-defined domains fall within the most favored regions of alpha helix (A labeled core highlighted in red. High levels of backbone conformations associated with alpha helical sequences include residues 6 to 21 in the N-terminal domain while amino acid residues 30 to 37 define the C-terminal domain. Other sequences in the refined molecular model include the more disordered N-terminal insertion sequence (residues Phe-1 to Leu-5) and the bend region including residues 22 to 29 bridging the N-terminal – C-terminal helical domains to form the helix-hairpin structure.

CHARMM-GUI (<http://www.charmm-gui.org>) command script (readme.csh) generated to run Gromacs simulation on computer cluster.

#!/bin/csh

#

# Generated by CHARMM-GUI (http://www.charmm-gui.org) v3.7

# This folder contains GROMACS formatted CHARMM36 force fields, a pre-optimized PDB structure, and GROMACS inputs.

# All input files were optimized for GROMACS 2019.2 or above, so lower version of GROMACS can cause some errors.

# We adopted the Verlet cut-off scheme for all minimization, equilibration, and production steps because it is

# faster and more accurate than the group scheme. If you have a trouble with a performance of Verlet scheme while

# running parallelized simulation, you should check if you are using appropriate command line.

# For MPI parallelizing, we recommand following command:

# mpirun -np $NUM_CPU gmx mdrun -ntomp 1

set init = step5_input

set rest_prefix = step5_input

set mini_prefix = step6.0_minimization

set equi_prefix = step6.%d_equilibration

set prod_prefix = step7_production

set prod_step = step7

# Minimization

# In the case that there is a problem during minimization using a single precision of GROMACS, please try to use

# a double precision of GROMACS only for the minimization step.

gmx grompp -f ${mini_prefix}.mdp -o ${mini_prefix}.tpr -c ${init}.gro -r ${rest_prefix}.gro -p topol.top -n index.ndx

gmx_d mdrun -v -deffnm ${mini_prefix}

# Equilibration

set cnt = 1

set cntmax = 6

while ( ${cnt} <= ${cntmax} )

@ pcnt = ${cnt} - 1

set istep = `printf ${equi_prefix} ${cnt}`

set pstep = `printf ${equi_prefix} ${pcnt}`

if ( ${cnt} == 1 ) set pstep = ${mini_prefix}

gmx grompp -f ${istep}.mdp -o ${istep}.tpr -c ${pstep}.gro -r ${rest_prefix}.gro -p topol.top -n index.ndx

gmx mdrun -v -deffnm ${istep}

@ cnt += 1

end

# Production

set cnt = 1

set cntmax = 10

while ( ${cnt} <= ${cntmax} )

@ pcnt = ${cnt} - 1

set istep = ${prod_step}_${cnt}

set pstep = ${prod_step}_${pcnt}

if ( ${cnt} == 1 ) then

set pstep = `printf ${equi_prefix} 6`

gmx grompp -f ${prod_prefix}.mdp -o ${istep}.tpr -c ${pstep}.gro -p topol.top -n index.ndx

else

gmx grompp -f ${prod_prefix}.mdp -o ${istep}.tpr -c ${pstep}.gro -t ${pstep}.cpt -p topol.top -n index.ndx

endif

gmx mdrun -v -deffnm ${istep}

@ cnt += 1

end

**Analysis of Molecular Simulation**


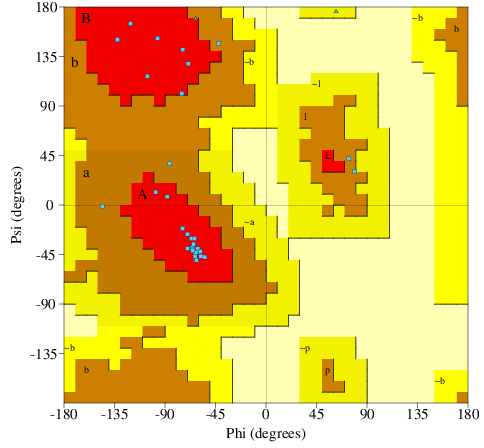


1. Ramachandran Plot statistics

No. of

residues %-tage

------ ------

Most favoured regions [A,B,L] 29 90.6%

Additional allowed regions [a,b,l,p] 3 9.4%

Generously allowed regions [~a,~b,~l,~p] 0 0.0%

Disallowed regions [XX] 0 0.0%

---- ------

Non-glycine and non-proline residues 32 100.0%

End-residues (excl. Gly and Pro) 2

Glycine residues 2

Proline residues 5

----

Total number of residues 41

Based on an analysis of 118 structures of resolution of at least 2.0 Angstroms and R-factor no greater than 20.0 a good quality model would be expected to have over 90% in the most favoured regions [A,B,L].

2. G-Factors

Average

Parameter Score Score

--------- ----- -----

Dihedral angles:-

Phi-psi distribution -0.09

Chi1-chi2 distribution 0.02

Chi1 only 0.18

Chi3 & chi4 0.80

Omega -0.73*

-0.10

=====

Main-chain covalent forces:-

Main-chain bond lengths -2.19**

Main-chain bond angles -1.09**

-1.55**

=====

OVERALL AVERAGE -0.83*

=====

G-factors provide a measure of how unusual, or out-of-the-ordinary, a property is.

Values below -0.5* - unusual

Values below -1.0** - highly unusual

Important note: The main-chain bond-lengths and bond angles are compared with the Engh & Huber (1991) ideal values derived from small-molecule data. Therefore, structures refined using different restraints may show apparently large deviations from normality.


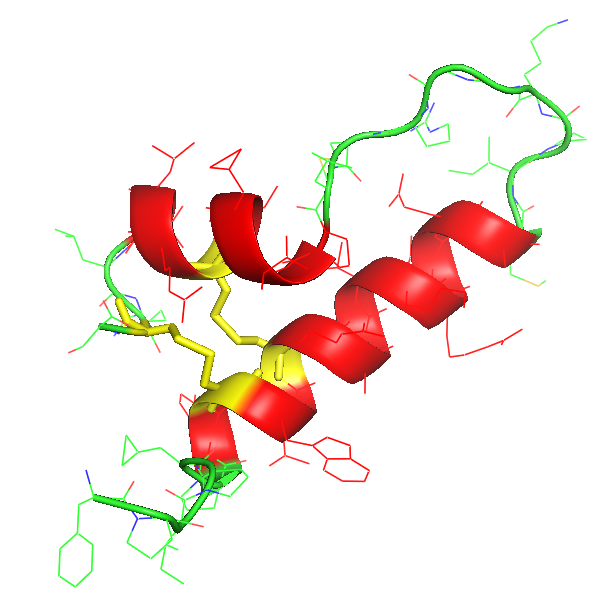


Molecular illustration of SMB three-dimensional molecular dynamics refined structure in synthetic surfactant lipids based on the mass spectral determined disulfide connectivities and ^13^C isotope enhanced backbone mapping of predicted helical domains. Alpha helical domains are highlighted in red ribbons, disulfide linkages and cysteines are shown in yellow, and bend and disordered backbone structures are shown as green tubes.

| **Table of helices** |
| --- |
|  |
| \|  \| \| \| \| \| \| \| \| \| \| \| \| \| \| \| \| \| \| \| \| \| \| \| \| --- \| --- \| --- \| --- \| --- \| --- \| --- \| --- \| --- \| --- \| --- \| --- \| --- \| --- \| --- \| --- \| --- \| --- \| --- \| --- \| --- \| --- \| --- \| \| **No.** \|  \| **Start** \|  \| **End** \|  \| **Type** \|  \| **No. resid** \|  \| **Length** \|  \| **Unit rise** \|  \| **Residues per turn** \|  \| **Pitch** \|  \| **Deviation from ideal** \|  \| **Sequence** \|  \|  \| \|  \| \| \| \| \| \| \| \| \| \| \| \| \| \| \| \| \| \| \| \| \| \| \| \| 1. \|  \| Tyr7 \|  \| Met21 \|  \| H \|  \| 15 \|  \| 23.24 \|  \| 1.52 \|  \| 3.60 \|  \| 5.48 \|  \| 6.2 \|  \| YCWLCRALIKRIQAM \|  \|  \| \| 2. \|  \| Pro30 \|  \| Leu36 \|  \| H \|  \| 7 \|  \| 10.96 \|  \| 1.50 \|  \| 3.57 \|  \| 5.35 \|  \| 9.0 \|  \| PQLVCRL \|  \|  \| \|  \| \| \| \| \| \| \| \| \| \| \| \| \| \| \| \| \| \| \| \| \| \| \| |
|  |
| Number of helices in chain A: 2 |
|  |


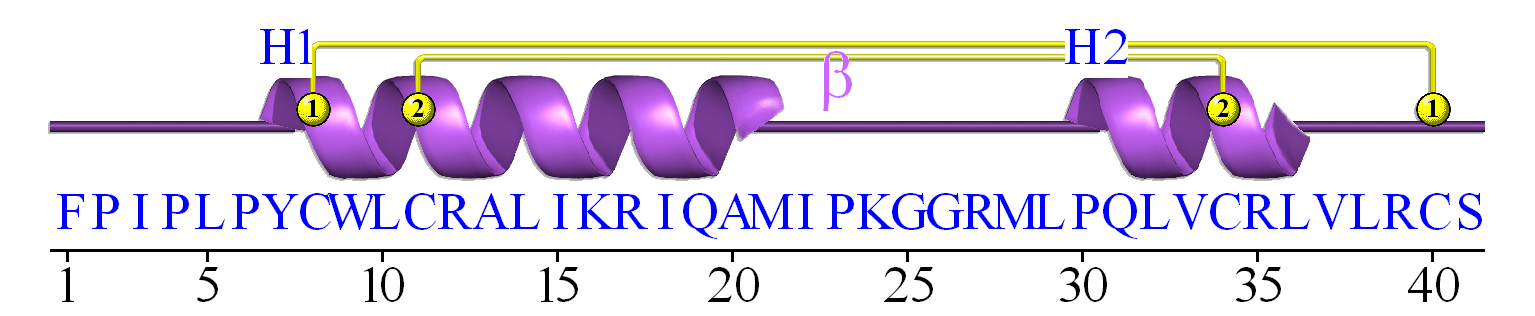

|  | \| **Chain 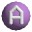 (41 residues)** \| \| --- \| \|    \| Disulphides \| \| --- \| \|  \| \| \|  \| \| \| \| \| \| \| \| \| \| \| \| \| \| \| \| --- \| --- \| --- \| --- \| --- \| --- \| --- \| --- \| --- \| --- \| --- \| --- \| --- \| --- \| --- \| \| **1st cysteine** \|  \| **2nd cysteine** \|  \| **Type** \|  \| **Chi1** \|  \| **Chi2** \|  \| **Chi3** \|  \| **Chi2p** \|  \| **Chi1p** \| \|  \| \| \| \| \| \| \| \| \| \| \| \| \| \| \| \| A 8 \|  \| A 40 \|  \| RHH \|  \| -160.9 \|  \| 66.0 \|  \| 89.8 \|  \| -131.4 \|  \| -50.9 \| \| A 11 \|  \| A 34 \|  \| LHS \|  \| -178.5 \|  \| -92.3 \|  \| -108.9 \|  \| -84.2 \|  \| -63.0 \| \|  \| \| \| \| \| \| \| \| \| \| \| \| \| \| \| \| \|  \| \| Number of disulphides in chain A: 2 \| \|  \| \|  \| \| **[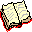](https://www.ebi.ac.uk/thornton-srv/databases/cgi-bin/pdbsum/GetPage.pl?pdbcode=c594&pdb_type=UPLOAD&code=155757&template=doc_p_disulph.html&doc=TRUE&pdbcode=n/a) Motif description** \| \| |
| --- | --- | --- | --- | --- | --- | --- | --- | --- | --- | --- | --- | --- | --- | --- | --- | --- | --- | --- | --- | --- | --- | --- | --- | --- | --- | --- | --- | --- | --- | --- | --- | --- | --- | --- | --- | --- | --- | --- | --- | --- | --- | --- | --- | --- | --- | --- | --- | --- | --- | --- | --- | --- | --- | --- | --- | --- | --- | --- | --- | --- | --- | --- | --- | --- | --- | --- | --- | --- | --- | --- | --- | --- | --- | --- | --- | --- | --- | --- | --- | --- | --- | --- | --- | --- | --- | --- | --- | --- | --- | --- | --- | --- | --- | --- | --- | --- | --- | --- | --- | --- | --- |

Disulphide bridges are identified for two cysteine residues whose sulphur atoms are less than 3Å apart. Richardson (1981) identified several categories of disulphide bridges based on their internal chi angles, in particular the chi2, chi3 and chi2' angles. We have loosely classified disulphides based on the signs of these angles into 5 categories. These are shown below.

**Disulphide type Chi2 Chi3 Chi2'**

left handed spiral (LHS) - - -

right handed hook (RHH) + + -

right handed spiral (RHS) + + +

short right handed hook (SRH) - + -

Note that the chi2 and chi2' values can be interchanged, as they merely reflect which of the two cysteines involved in the bridge is mentioned first. If the other cysteine were mentioned first the chi and chi' values would be interchanged.

Richardson found that the majority of disulphides could be classed as left handed spirals or right handed hooks.

**Table**

The data displayed for each disulphide bridge is as follows: The table shows the residue numbers of the two cysteines involved in the disulphide bridge and the type of disulphide. The type of bridge is abbreviated (RHH: right hand hook; SRH: short right hand hook; LHS: left handed spiral; RHS: right handed spiral). Chi1, chi2, chi3, chi2' and chi1' values and the distance between the C-alpha atoms of the residues involved are also recorded.

***Reference***

| **[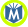](http://www.ncbi.nlm.nih.gov/entrez/query.fcgi?cmd=Retrieve&db=pubmed&dopt=Abstract&list_uids=7020376)** | Richardson JS (1981) The anatomy and taxonomy of protein structure. *Adv. Protein Chem*. **34**, 167-339. |
| --- | --- |

| **Table of beta turns** |
| --- |
|  |
| \|  \| \| \| \| \| \| \| \| \| \| \| \| \| \| \| \| \| \| \| \| \| \| \| \| --- \| --- \| --- \| --- \| --- \| --- \| --- \| --- \| --- \| --- \| --- \| --- \| --- \| --- \| --- \| --- \| --- \| --- \| --- \| --- \| --- \| --- \| --- \| \|  \|  \|  \|  \|  \|  \| **Turn** \|  \| **Residue i+1** \| \| \| \| \|  \| **Residue i+2** \| \| \| \| \|  \| **i to i+3** \|  \|  \| \|  \|  \|  \|  \|  \|  \|  \|  \|  \| \| \| \| \|  \|  \| \| \| \| \|  \|  \|  \|  \| \| **No.** \|  \| **Turn** \|  \| **Sequence*** \|  \| **type** \|  \| **Phi** \|  \| **Psi** \|  \| **Chi1** \|  \| **Phi** \|  \| **Psi** \|  \| **Chi1** \|  \| **CA-dist** \|  \| **H-bond** \| \|  \| \| \| \| \| \| \| \| \| \| \| \| \| \| \| \| \| \| \| \| \| \| \| \| 1. \|  \| Ile22-Gly25 \|  \| IPKG \|  \| I \|  \| -63.8 \|  \| -30.3 \|  \| -19.8 \|  \| -98.3 \|  \| 11.8 \|  \| -64.2 \|  \| 4.8 \|  \| No \| \|  \| \| \| \| \| \| \| \| \| \| \| \| \| \| \| \| \| \| \| \| \| \| \| |
|  |
| Number of beta turns in chain A: 1 |
|  |


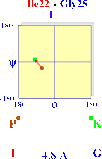


**References**

Laskowski R A, Rullmann, J A C, MacArthur M W, Kaptein R, Thornton J.M. AQUA and 1996. PROCHECK-NMR: Programs for checking the quality of protein structures solved by NMR. Journal of Biomolecular NMR, 1996; 8:477- 496.

Laskowski, R.A., MacArthur, M.W., Moss D.S. and Thornton, J.M. 1993. PROCHECK: a program to check the stereochemical quality of protein structures. J. Appl. Cryst., 1993; 26, 283-291.
