# Supplementary material for: Emulation of the structure of the Saposin protein fold by a lung surfactant peptide construct of surfactant Protein B
Source: PLoS One. 2022 Nov 3;17(11):e0276787. doi: 10.1371/journal.pone.0276787 (PMC9632872; doi:10.1371/journal.pone.0276787)
Supplement: S4 File — (DOCX) [file pone.0276787.s004.docx]

**S4** **– deposited in the ModelArchive (**<https://modelarchive.org>/doi/10.5452/ma-p57c0**)**

**Emulation of the Structure of the Saposin Protein Fold by a Lung Surfactant Peptide Construct of Surfactant Protein B**

Alan J. Waring^1,2^, Julian P. Whitelegge^3^, Shantanu K. Sharma^4^, Larry M. Gordon^1^,

Frans J. Walther^1,5,*^

^1^ Lundquist Institute for Biomedical Innovation at Harbor-UCLA Medical Center

1124 West Carson Street

Torrance, CA, USA

^2^ Department of Medicine

David Geffen School of Medicine

University of California Los Angeles

405 Hilgard Avenue

Los Angeles, CA, USA

^3^ NPI-Semel Institute for Neuroscience & Human Behavior Department of Psychiatry

& Biobehavioral Sciences, David Geffen School of Medicine at UCLA,

760 Westwood Plaza, Los Angeles, CA, USA

^4^ Materials and Process Simulation Center

California Institute of Technology

1200 East California Boulevard

Pasadena, CA, USA

^5^ Department of Pediatrics

David Geffen School of Medicine

University of California Los Angeles

405 Hilgard Avenue

Los Angeles, CA, USA

**Molecular Dynamics Refinement of ModelArchive (**[**https://modelarchive.org**](https://modelarchive.org)**/doi/10.5452/ma-p57c0)**

Lowest energy molecular coordinate set for Molecular Dynamics of SMB peptide in simulated surfactant lipid bilayer using the predicted SMB Peptide Structure based on initial coordinates predicted by AlphaFold with residue specific molecular constraints from experimental measures as a starting conformation.

ATOM 1 N PHE A 1 34.680 9.500 33.040 1.00 0.00 N

ATOM 2 CA PHE A 1 34.210 10.490 34.040 1.00 0.00 C

ATOM 3 C PHE A 1 33.710 11.840 33.450 1.00 0.00 C

ATOM 4 O PHE A 1 34.320 12.430 32.550 1.00 0.00 O

ATOM 5 CB PHE A 1 35.340 10.720 35.160 1.00 0.00 C

ATOM 6 CG PHE A 1 34.780 10.750 36.600 1.00 0.00 C

ATOM 7 CD1 PHE A 1 34.430 9.490 37.210 1.00 0.00 C

ATOM 8 CD2 PHE A 1 34.540 11.940 37.260 1.00 0.00 C

ATOM 9 CE1 PHE A 1 34.010 9.460 38.580 1.00 0.00 C

ATOM 10 CE2 PHE A 1 34.060 11.950 38.560 1.00 0.00 C

ATOM 11 CZ PHE A 1 33.830 10.680 39.230 1.00 0.00 C

ATOM 12 HA PHE A 1 33.380 9.950 34.490 1.00 0.00 H

ATOM 13 HB1 PHE A 1 35.810 11.710 34.980 1.00 0.00 H

ATOM 14 HB2 PHE A 1 36.190 10.030 34.990 1.00 0.00 H

ATOM 15 HD1 PHE A 1 34.530 8.590 36.630 1.00 0.00 H

ATOM 16 HD2 PHE A 1 34.810 12.800 36.660 1.00 0.00 H

ATOM 17 HE1 PHE A 1 33.690 8.570 39.100 1.00 0.00 H

ATOM 18 HE2 PHE A 1 33.730 12.860 39.050 1.00 0.00 H

ATOM 19 HZ PHE A 1 33.600 10.750 40.290 1.00 0.00 H

ATOM 20 HT1 PHE A 1 35.460 9.830 32.450 1.00 0.00 H

ATOM 21 HT2 PHE A 1 33.910 9.110 32.470 1.00 0.00 H

ATOM 22 HT3 PHE A 1 35.100 8.650 33.470 1.00 0.00 H

ATOM 23 N PRO A 2 32.650 12.430 33.940 1.00 0.00 N

ATOM 24 CA PRO A 2 32.360 13.870 33.650 1.00 0.00 C

ATOM 25 C PRO A 2 33.490 14.850 33.840 1.00 0.00 C

ATOM 26 O PRO A 2 34.190 14.730 34.890 1.00 0.00 O

ATOM 27 CB PRO A 2 31.210 14.140 34.600 1.00 0.00 C

ATOM 28 CG PRO A 2 30.390 12.820 34.600 1.00 0.00 C

ATOM 29 CD PRO A 2 31.540 11.780 34.590 1.00 0.00 C

ATOM 30 HA PRO A 2 32.130 14.090 32.620 1.00 0.00 H

ATOM 31 HB1 PRO A 2 30.540 14.990 34.310 1.00 0.00 H

ATOM 32 HB2 PRO A 2 31.570 14.190 35.640 1.00 0.00 H

ATOM 33 HG1 PRO A 2 29.720 12.680 35.470 1.00 0.00 H

ATOM 34 HG2 PRO A 2 29.840 12.750 33.640 1.00 0.00 H

ATOM 35 HD1 PRO A 2 31.760 11.530 35.650 1.00 0.00 H

ATOM 36 HD2 PRO A 2 31.090 10.910 34.060 1.00 0.00 H

ATOM 37 N ILE A 3 33.790 15.770 32.900 1.00 0.00 N

ATOM 38 CA ILE A 3 34.870 16.750 33.030 1.00 0.00 C

ATOM 39 C ILE A 3 34.180 18.020 33.610 1.00 0.00 C

ATOM 40 O ILE A 3 33.090 18.330 33.100 1.00 0.00 O

ATOM 41 CB ILE A 3 35.710 17.030 31.780 1.00 0.00 C

ATOM 42 CG1 ILE A 3 34.760 17.120 30.640 1.00 0.00 C

ATOM 43 CG2 ILE A 3 36.630 15.790 31.580 1.00 0.00 C

ATOM 44 CD ILE A 3 35.450 17.590 29.280 1.00 0.00 C

ATOM 45 HN ILE A 3 33.400 15.720 31.990 1.00 0.00 H

ATOM 46 HA ILE A 3 35.540 16.340 33.770 1.00 0.00 H

ATOM 47 HB ILE A 3 36.330 17.950 31.850 1.00 0.00 H

ATOM 48 HG11 ILE A 3 33.910 17.800 30.850 1.00 0.00 H

ATOM 49 HG12 ILE A 3 34.300 16.120 30.510 1.00 0.00 H

ATOM 50 HG21 ILE A 3 36.020 14.850 31.600 1.00 0.00 H

ATOM 51 HG22 ILE A 3 37.390 15.820 32.380 1.00 0.00 H

ATOM 52 HG23 ILE A 3 37.140 15.780 30.590 1.00 0.00 H

ATOM 53 HD1 ILE A 3 36.020 16.690 28.960 1.00 0.00 H

ATOM 54 HD2 ILE A 3 36.170 18.430 29.380 1.00 0.00 H

ATOM 55 HD3 ILE A 3 34.670 17.750 28.500 1.00 0.00 H

ATOM 56 N PRO A 4 34.770 18.810 34.500 1.00 0.00 N

ATOM 57 CA PRO A 4 33.990 19.870 35.250 1.00 0.00 C

ATOM 58 C PRO A 4 33.870 21.220 34.430 1.00 0.00 C

ATOM 59 O PRO A 4 34.540 22.160 34.780 1.00 0.00 O

ATOM 60 CB PRO A 4 34.720 19.920 36.570 1.00 0.00 C

ATOM 61 CG PRO A 4 36.130 19.540 36.220 1.00 0.00 C

ATOM 62 CD PRO A 4 36.010 18.510 35.160 1.00 0.00 C

ATOM 63 HA PRO A 4 32.950 19.620 35.430 1.00 0.00 H

ATOM 64 HB1 PRO A 4 34.240 19.270 37.340 1.00 0.00 H

ATOM 65 HB2 PRO A 4 34.660 20.950 36.960 1.00 0.00 H

ATOM 66 HG1 PRO A 4 36.760 20.330 35.770 1.00 0.00 H

ATOM 67 HG2 PRO A 4 36.670 19.090 37.080 1.00 0.00 H

ATOM 68 HD1 PRO A 4 36.810 18.510 34.390 1.00 0.00 H

ATOM 69 HD2 PRO A 4 35.990 17.450 35.520 1.00 0.00 H

ATOM 70 N LEU A 5 33.120 21.190 33.320 1.00 0.00 N

ATOM 71 CA LEU A 5 33.140 22.230 32.280 1.00 0.00 C

ATOM 72 C LEU A 5 32.950 23.610 32.840 1.00 0.00 C

ATOM 73 O LEU A 5 33.740 24.480 32.490 1.00 0.00 O

ATOM 74 CB LEU A 5 32.200 21.930 31.090 1.00 0.00 C

ATOM 75 CG LEU A 5 32.530 20.650 30.240 1.00 0.00 C

ATOM 76 CD1 LEU A 5 31.130 19.930 29.880 1.00 0.00 C

ATOM 77 CD2 LEU A 5 33.320 20.970 28.900 1.00 0.00 C

ATOM 78 HN LEU A 5 32.630 20.340 33.090 1.00 0.00 H

ATOM 79 HA LEU A 5 34.160 22.260 31.940 1.00 0.00 H

ATOM 80 HB1 LEU A 5 32.230 22.840 30.460 1.00 0.00 H

ATOM 81 HB2 LEU A 5 31.130 21.940 31.390 1.00 0.00 H

ATOM 82 HG LEU A 5 33.200 19.970 30.800 1.00 0.00 H

ATOM 83 HD11 LEU A 5 30.570 20.640 29.230 1.00 0.00 H

ATOM 84 HD12 LEU A 5 30.740 19.690 30.900 1.00 0.00 H

ATOM 85 HD13 LEU A 5 31.300 18.900 29.500 1.00 0.00 H

ATOM 86 HD21 LEU A 5 33.510 20.080 28.270 1.00 0.00 H

ATOM 87 HD22 LEU A 5 34.310 21.450 29.070 1.00 0.00 H

ATOM 88 HD23 LEU A 5 32.750 21.740 28.340 1.00 0.00 H

ATOM 89 N PRO A 6 32.050 24.010 33.760 1.00 0.00 N

ATOM 90 CA PRO A 6 32.100 25.400 34.290 1.00 0.00 C

ATOM 91 C PRO A 6 33.360 25.730 35.020 1.00 0.00 C

ATOM 92 O PRO A 6 33.690 26.910 34.860 1.00 0.00 O

ATOM 93 CB PRO A 6 30.720 25.530 35.100 1.00 0.00 C

ATOM 94 CG PRO A 6 30.270 24.050 35.350 1.00 0.00 C

ATOM 95 CD PRO A 6 30.820 23.320 34.160 1.00 0.00 C

ATOM 96 HA PRO A 6 32.060 26.100 33.470 1.00 0.00 H

ATOM 97 HB1 PRO A 6 29.930 25.910 34.420 1.00 0.00 H

ATOM 98 HB2 PRO A 6 30.900 26.240 35.940 1.00 0.00 H

ATOM 99 HG1 PRO A 6 30.850 23.690 36.220 1.00 0.00 H

ATOM 100 HG2 PRO A 6 29.180 24.050 35.560 1.00 0.00 H

ATOM 101 HD1 PRO A 6 30.990 22.260 34.420 1.00 0.00 H

ATOM 102 HD2 PRO A 6 30.180 23.330 33.260 1.00 0.00 H

ATOM 103 N TYR A 7 34.100 24.850 35.660 1.00 0.00 N

ATOM 104 CA TYR A 7 35.380 25.130 36.360 1.00 0.00 C

ATOM 105 C TYR A 7 36.520 25.280 35.410 1.00 0.00 C

ATOM 106 O TYR A 7 37.480 26.040 35.650 1.00 0.00 O

ATOM 107 CB TYR A 7 35.880 24.100 37.430 1.00 0.00 C

ATOM 108 CG TYR A 7 34.900 24.180 38.530 1.00 0.00 C

ATOM 109 CD1 TYR A 7 33.900 23.200 38.780 1.00 0.00 C

ATOM 110 CD2 TYR A 7 34.970 25.350 39.320 1.00 0.00 C

ATOM 111 CE1 TYR A 7 33.120 23.350 39.980 1.00 0.00 C

ATOM 112 CE2 TYR A 7 34.170 25.450 40.560 1.00 0.00 C

ATOM 113 CZ TYR A 7 33.350 24.350 40.850 1.00 0.00 C

ATOM 114 OH TYR A 7 32.470 24.590 41.920 1.00 0.00 O

ATOM 115 HN TYR A 7 33.790 23.910 35.670 1.00 0.00 H

ATOM 116 HA TYR A 7 35.390 26.100 36.830 1.00 0.00 H

ATOM 117 HB1 TYR A 7 36.920 24.240 37.790 1.00 0.00 H

ATOM 118 HB2 TYR A 7 35.870 23.030 37.140 1.00 0.00 H

ATOM 119 HD1 TYR A 7 33.730 22.370 38.100 1.00 0.00 H

ATOM 120 HD2 TYR A 7 35.680 26.100 39.010 1.00 0.00 H

ATOM 121 HE1 TYR A 7 32.280 22.690 40.140 1.00 0.00 H

ATOM 122 HE2 TYR A 7 34.260 26.310 41.190 1.00 0.00 H

ATOM 123 HH TYR A 7 32.570 25.520 42.170 1.00 0.00 H

ATOM 124 N CYS A 8 36.490 24.530 34.250 1.00 0.00 N

ATOM 125 CA CYS A 8 37.530 24.690 33.190 1.00 0.00 C

ATOM 126 C CYS A 8 37.440 26.070 32.720 1.00 0.00 C

ATOM 127 O CYS A 8 38.450 26.760 32.680 1.00 0.00 O

ATOM 128 CB CYS A 8 37.380 23.750 32.040 1.00 0.00 C

ATOM 129 SG CYS A 8 37.490 21.990 32.640 1.00 0.00 S

ATOM 130 HN CYS A 8 35.800 23.820 34.130 1.00 0.00 H

ATOM 131 HA CYS A 8 38.500 24.670 33.670 1.00 0.00 H

ATOM 132 HB1 CYS A 8 38.110 23.900 31.210 1.00 0.00 H

ATOM 133 HB2 CYS A 8 36.390 23.970 31.580 1.00 0.00 H

ATOM 134 N TRP A 9 36.200 26.540 32.410 1.00 0.00 N

ATOM 135 CA TRP A 9 35.880 27.780 31.810 1.00 0.00 C

ATOM 136 C TRP A 9 36.370 28.980 32.660 1.00 0.00 C

ATOM 137 O TRP A 9 36.930 29.950 32.110 1.00 0.00 O

ATOM 138 CB TRP A 9 34.380 27.840 31.370 1.00 0.00 C

ATOM 139 CG TRP A 9 33.780 29.130 30.830 1.00 0.00 C

ATOM 140 CD1 TRP A 9 33.450 29.320 29.510 1.00 0.00 C

ATOM 141 CD2 TRP A 9 33.280 30.230 31.580 1.00 0.00 C

ATOM 142 CE2 TRP A 9 32.610 31.080 30.600 1.00 0.00 C

ATOM 143 CE3 TRP A 9 33.180 30.510 32.940 1.00 0.00 C

ATOM 144 NE1 TRP A 9 32.780 30.520 29.380 1.00 0.00 N

ATOM 145 CZ2 TRP A 9 31.980 32.260 30.970 1.00 0.00 C

ATOM 146 CZ3 TRP A 9 32.470 31.670 33.310 1.00 0.00 C

ATOM 147 CH2 TRP A 9 31.890 32.500 32.390 1.00 0.00 C

ATOM 148 HN TRP A 9 35.380 25.980 32.490 1.00 0.00 H

ATOM 149 HA TRP A 9 36.480 27.830 30.910 1.00 0.00 H

ATOM 150 HB1 TRP A 9 33.780 27.360 32.170 1.00 0.00 H

ATOM 151 HB2 TRP A 9 34.360 27.150 30.500 1.00 0.00 H

ATOM 152 HD1 TRP A 9 33.500 28.480 28.840 1.00 0.00 H

ATOM 153 HE1 TRP A 9 32.380 30.970 28.610 1.00 0.00 H

ATOM 154 HE3 TRP A 9 33.640 30.040 33.800 1.00 0.00 H

ATOM 155 HZ2 TRP A 9 31.460 32.860 30.240 1.00 0.00 H

ATOM 156 HZ3 TRP A 9 32.330 31.870 34.360 1.00 0.00 H

ATOM 157 HH2 TRP A 9 31.270 33.250 32.860 1.00 0.00 H

ATOM 158 N LEU A 10 36.240 28.900 33.980 1.00 0.00 N

ATOM 159 CA LEU A 10 36.700 29.670 35.050 1.00 0.00 C

ATOM 160 C LEU A 10 38.150 29.840 35.140 1.00 0.00 C

ATOM 161 O LEU A 10 38.620 30.960 35.270 1.00 0.00 O

ATOM 162 CB LEU A 10 36.120 29.170 36.410 1.00 0.00 C

ATOM 163 CG LEU A 10 34.700 29.720 36.740 1.00 0.00 C

ATOM 164 CD1 LEU A 10 34.140 28.790 37.780 1.00 0.00 C

ATOM 165 CD2 LEU A 10 34.580 31.180 37.230 1.00 0.00 C

ATOM 166 HN LEU A 10 35.840 28.030 34.280 1.00 0.00 H

ATOM 167 HA LEU A 10 36.360 30.690 34.940 1.00 0.00 H

ATOM 168 HB1 LEU A 10 36.720 29.580 37.250 1.00 0.00 H

ATOM 169 HB2 LEU A 10 35.940 28.080 36.330 1.00 0.00 H

ATOM 170 HG LEU A 10 34.090 29.600 35.820 1.00 0.00 H

ATOM 171 HD11 LEU A 10 33.160 29.180 38.150 1.00 0.00 H

ATOM 172 HD12 LEU A 10 34.750 28.750 38.710 1.00 0.00 H

ATOM 173 HD13 LEU A 10 33.930 27.760 37.430 1.00 0.00 H

ATOM 174 HD21 LEU A 10 33.510 31.460 37.360 1.00 0.00 H

ATOM 175 HD22 LEU A 10 35.020 31.970 36.590 1.00 0.00 H

ATOM 176 HD23 LEU A 10 35.100 31.300 38.200 1.00 0.00 H

ATOM 177 N CYS A 11 38.930 28.690 35.020 1.00 0.00 N

ATOM 178 CA CYS A 11 40.370 28.680 34.920 1.00 0.00 C

ATOM 179 C CYS A 11 40.930 29.300 33.660 1.00 0.00 C

ATOM 180 O CYS A 11 41.850 30.090 33.730 1.00 0.00 O

ATOM 181 CB CYS A 11 40.810 27.160 35.070 1.00 0.00 C

ATOM 182 SG CYS A 11 42.460 26.880 35.690 1.00 0.00 S

ATOM 183 HN CYS A 11 38.510 27.780 35.030 1.00 0.00 H

ATOM 184 HA CYS A 11 40.790 29.280 35.720 1.00 0.00 H

ATOM 185 HB1 CYS A 11 40.700 26.570 34.130 1.00 0.00 H

ATOM 186 HB2 CYS A 11 40.190 26.790 35.920 1.00 0.00 H

ATOM 187 N ARG A 12 40.320 28.930 32.500 1.00 0.00 N

ATOM 188 CA ARG A 12 40.540 29.480 31.200 1.00 0.00 C

ATOM 189 C ARG A 12 40.240 30.920 30.950 1.00 0.00 C

ATOM 190 O ARG A 12 41.040 31.670 30.360 1.00 0.00 O

ATOM 191 CB ARG A 12 39.770 28.740 30.070 1.00 0.00 C

ATOM 192 CG ARG A 12 40.400 27.390 29.560 1.00 0.00 C

ATOM 193 CD ARG A 12 40.950 26.370 30.590 1.00 0.00 C

ATOM 194 NE ARG A 12 41.740 25.330 29.870 1.00 0.00 N

ATOM 195 CZ ARG A 12 41.230 24.320 29.130 1.00 0.00 C

ATOM 196 NH1 ARG A 12 39.970 24.170 29.060 1.00 0.00 N1+

ATOM 197 NH2 ARG A 12 42.060 23.510 28.550 1.00 0.00 N

ATOM 198 HN ARG A 12 39.590 28.250 32.540 1.00 0.00 H

ATOM 199 HA ARG A 12 41.580 29.360 30.940 1.00 0.00 H

ATOM 200 HB1 ARG A 12 39.740 29.320 29.130 1.00 0.00 H

ATOM 201 HB2 ARG A 12 38.720 28.480 30.340 1.00 0.00 H

ATOM 202 HG1 ARG A 12 41.270 27.730 28.960 1.00 0.00 H

ATOM 203 HG2 ARG A 12 39.690 26.810 28.940 1.00 0.00 H

ATOM 204 HD1 ARG A 12 40.180 25.940 31.270 1.00 0.00 H

ATOM 205 HD2 ARG A 12 41.800 26.790 31.160 1.00 0.00 H

ATOM 206 HE ARG A 12 42.730 25.440 29.820 1.00 0.00 H

ATOM 207 HH11 ARG A 12 39.370 24.890 29.400 1.00 0.00 H

ATOM 208 HH12 ARG A 12 39.470 23.500 28.500 1.00 0.00 H

ATOM 209 HH21 ARG A 12 42.840 23.960 28.120 1.00 0.00 H

ATOM 210 HH22 ARG A 12 41.720 22.790 27.940 1.00 0.00 H

ATOM 211 N ALA A 13 39.160 31.530 31.520 1.00 0.00 N

ATOM 212 CA ALA A 13 38.990 32.940 31.540 1.00 0.00 C

ATOM 213 C ALA A 13 40.060 33.770 32.300 1.00 0.00 C

ATOM 214 O ALA A 13 40.380 34.940 32.010 1.00 0.00 O

ATOM 215 CB ALA A 13 37.590 33.250 32.120 1.00 0.00 C

ATOM 216 HN ALA A 13 38.520 30.960 32.040 1.00 0.00 H

ATOM 217 HA ALA A 13 38.960 33.330 30.540 1.00 0.00 H

ATOM 218 HB1 ALA A 13 36.770 32.600 31.760 1.00 0.00 H

ATOM 219 HB2 ALA A 13 37.260 34.270 31.830 1.00 0.00 H

ATOM 220 HB3 ALA A 13 37.670 33.310 33.230 1.00 0.00 H

ATOM 221 N LEU A 14 40.660 33.240 33.370 1.00 0.00 N

ATOM 222 CA LEU A 14 41.850 33.840 33.990 1.00 0.00 C

ATOM 223 C LEU A 14 43.060 33.830 33.110 1.00 0.00 C

ATOM 224 O LEU A 14 43.720 34.900 33.080 1.00 0.00 O

ATOM 225 CB LEU A 14 42.230 33.120 35.290 1.00 0.00 C

ATOM 226 CG LEU A 14 43.200 33.760 36.370 1.00 0.00 C

ATOM 227 CD1 LEU A 14 42.680 34.870 37.240 1.00 0.00 C

ATOM 228 CD2 LEU A 14 43.620 32.620 37.350 1.00 0.00 C

ATOM 229 HN LEU A 14 40.580 32.270 33.570 1.00 0.00 H

ATOM 230 HA LEU A 14 41.620 34.840 34.310 1.00 0.00 H

ATOM 231 HB1 LEU A 14 42.680 32.120 35.090 1.00 0.00 H

ATOM 232 HB2 LEU A 14 41.280 32.980 35.850 1.00 0.00 H

ATOM 233 HG LEU A 14 44.130 34.050 35.830 1.00 0.00 H

ATOM 234 HD11 LEU A 14 43.510 35.300 37.830 1.00 0.00 H

ATOM 235 HD12 LEU A 14 41.910 34.550 37.970 1.00 0.00 H

ATOM 236 HD13 LEU A 14 42.350 35.740 36.620 1.00 0.00 H

ATOM 237 HD21 LEU A 14 42.780 31.900 37.440 1.00 0.00 H

ATOM 238 HD22 LEU A 14 43.830 33.060 38.350 1.00 0.00 H

ATOM 239 HD23 LEU A 14 44.520 32.120 36.930 1.00 0.00 H

ATOM 240 N ILE A 15 43.340 32.780 32.300 1.00 0.00 N

ATOM 241 CA ILE A 15 44.370 32.780 31.270 1.00 0.00 C

ATOM 242 C ILE A 15 44.110 33.960 30.320 1.00 0.00 C

ATOM 243 O ILE A 15 45.010 34.770 30.160 1.00 0.00 O

ATOM 244 CB ILE A 15 44.400 31.520 30.420 1.00 0.00 C

ATOM 245 CG1 ILE A 15 44.190 30.260 31.200 1.00 0.00 C

ATOM 246 CG2 ILE A 15 45.670 31.500 29.550 1.00 0.00 C

ATOM 247 CD ILE A 15 44.090 28.940 30.310 1.00 0.00 C

ATOM 248 HN ILE A 15 42.740 31.980 32.230 1.00 0.00 H

ATOM 249 HA ILE A 15 45.320 32.950 31.760 1.00 0.00 H

ATOM 250 HB ILE A 15 43.520 31.480 29.730 1.00 0.00 H

ATOM 251 HG11 ILE A 15 43.260 30.260 31.810 1.00 0.00 H

ATOM 252 HG12 ILE A 15 45.020 30.190 31.940 1.00 0.00 H

ATOM 253 HG21 ILE A 15 46.520 31.340 30.250 1.00 0.00 H

ATOM 254 HG22 ILE A 15 45.760 32.470 29.010 1.00 0.00 H

ATOM 255 HG23 ILE A 15 45.840 30.760 28.740 1.00 0.00 H

ATOM 256 HD1 ILE A 15 43.260 29.060 29.580 1.00 0.00 H

ATOM 257 HD2 ILE A 15 43.910 28.020 30.900 1.00 0.00 H

ATOM 258 HD3 ILE A 15 45.060 28.850 29.780 1.00 0.00 H

ATOM 259 N LYS A 16 42.890 34.230 29.850 1.00 0.00 N

ATOM 260 CA LYS A 16 42.510 35.420 29.030 1.00 0.00 C

ATOM 261 C LYS A 16 42.880 36.710 29.750 1.00 0.00 C

ATOM 262 O LYS A 16 43.520 37.610 29.190 1.00 0.00 O

ATOM 263 CB LYS A 16 41.000 35.330 28.690 1.00 0.00 C

ATOM 264 CG LYS A 16 40.490 34.140 27.830 1.00 0.00 C

ATOM 265 CD LYS A 16 41.190 34.070 26.420 1.00 0.00 C

ATOM 266 CE LYS A 16 41.070 35.270 25.570 1.00 0.00 C

ATOM 267 NZ LYS A 16 39.700 35.590 25.470 1.00 0.00 N1+

ATOM 268 HN LYS A 16 42.140 33.640 30.160 1.00 0.00 H

ATOM 269 HA LYS A 16 43.000 35.340 28.070 1.00 0.00 H

ATOM 270 HB1 LYS A 16 40.700 36.270 28.180 1.00 0.00 H

ATOM 271 HB2 LYS A 16 40.460 35.280 29.660 1.00 0.00 H

ATOM 272 HG1 LYS A 16 39.380 34.190 27.710 1.00 0.00 H

ATOM 273 HG2 LYS A 16 40.710 33.200 28.390 1.00 0.00 H

ATOM 274 HD1 LYS A 16 40.920 33.160 25.840 1.00 0.00 H

ATOM 275 HD2 LYS A 16 42.270 33.910 26.650 1.00 0.00 H

ATOM 276 HE1 LYS A 16 41.460 35.120 24.540 1.00 0.00 H

ATOM 277 HE2 LYS A 16 41.620 36.170 25.910 1.00 0.00 H

ATOM 278 HZ1 LYS A 16 39.690 36.130 24.580 1.00 0.00 H

ATOM 279 HZ2 LYS A 16 39.230 36.180 26.180 1.00 0.00 H

ATOM 280 HZ3 LYS A 16 39.120 34.730 25.400 1.00 0.00 H

ATOM 281 N ARG A 17 42.630 36.850 31.060 1.00 0.00 N

ATOM 282 CA ARG A 17 43.020 38.060 31.820 1.00 0.00 C

ATOM 283 C ARG A 17 44.560 38.290 32.040 1.00 0.00 C

ATOM 284 O ARG A 17 45.070 39.380 32.050 1.00 0.00 O

ATOM 285 CB ARG A 17 42.260 38.070 33.130 1.00 0.00 C

ATOM 286 CG ARG A 17 40.720 38.150 33.090 1.00 0.00 C

ATOM 287 CD ARG A 17 40.220 39.570 32.750 1.00 0.00 C

ATOM 288 NE ARG A 17 40.150 39.760 31.300 1.00 0.00 N

ATOM 289 CZ ARG A 17 40.370 40.770 30.590 1.00 0.00 C

ATOM 290 NH1 ARG A 17 41.030 41.870 30.790 1.00 0.00 N1+

ATOM 291 NH2 ARG A 17 39.810 40.670 29.370 1.00 0.00 N

ATOM 292 HN ARG A 17 42.060 36.150 31.490 1.00 0.00 H

ATOM 293 HA ARG A 17 42.670 38.810 31.130 1.00 0.00 H

ATOM 294 HB1 ARG A 17 42.590 38.980 33.670 1.00 0.00 H

ATOM 295 HB2 ARG A 17 42.480 37.140 33.690 1.00 0.00 H

ATOM 296 HG1 ARG A 17 40.290 37.890 34.070 1.00 0.00 H

ATOM 297 HG2 ARG A 17 40.390 37.340 32.400 1.00 0.00 H

ATOM 298 HD1 ARG A 17 40.860 40.320 33.270 1.00 0.00 H

ATOM 299 HD2 ARG A 17 39.250 39.790 33.250 1.00 0.00 H

ATOM 300 HE ARG A 17 39.520 39.150 30.830 1.00 0.00 H

ATOM 301 HH11 ARG A 17 41.580 42.000 31.610 1.00 0.00 H

ATOM 302 HH12 ARG A 17 40.960 42.590 30.090 1.00 0.00 H

ATOM 303 HH21 ARG A 17 39.510 39.750 29.160 1.00 0.00 H

ATOM 304 HH22 ARG A 17 40.120 41.340 28.690 1.00 0.00 H

ATOM 305 N ILE A 18 45.310 37.200 32.270 1.00 0.00 N

ATOM 306 CA ILE A 18 46.800 37.250 32.260 1.00 0.00 C

ATOM 307 C ILE A 18 47.270 37.620 30.800 1.00 0.00 C

ATOM 308 O ILE A 18 48.190 38.400 30.530 1.00 0.00 O

ATOM 309 CB ILE A 18 47.340 35.800 32.650 1.00 0.00 C

ATOM 310 CG1 ILE A 18 47.030 35.610 34.150 1.00 0.00 C

ATOM 311 CG2 ILE A 18 48.810 35.390 32.220 1.00 0.00 C

ATOM 312 CD ILE A 18 47.140 34.210 34.650 1.00 0.00 C

ATOM 313 HN ILE A 18 44.870 36.300 32.350 1.00 0.00 H

ATOM 314 HA ILE A 18 47.200 37.970 32.960 1.00 0.00 H

ATOM 315 HB ILE A 18 46.720 35.130 32.000 1.00 0.00 H

ATOM 316 HG11 ILE A 18 46.010 35.940 34.450 1.00 0.00 H

ATOM 317 HG12 ILE A 18 47.670 36.250 34.790 1.00 0.00 H

ATOM 318 HG21 ILE A 18 49.510 36.020 32.820 1.00 0.00 H

ATOM 319 HG22 ILE A 18 49.070 35.480 31.150 1.00 0.00 H

ATOM 320 HG23 ILE A 18 48.990 34.350 32.580 1.00 0.00 H

ATOM 321 HD1 ILE A 18 46.830 34.050 35.710 1.00 0.00 H

ATOM 322 HD2 ILE A 18 48.180 33.830 34.650 1.00 0.00 H

ATOM 323 HD3 ILE A 18 46.540 33.530 34.010 1.00 0.00 H

ATOM 324 N GLN A 19 46.670 37.030 29.710 1.00 0.00 N

ATOM 325 CA GLN A 19 46.920 37.480 28.360 1.00 0.00 C

ATOM 326 C GLN A 19 46.660 38.900 28.120 1.00 0.00 C

ATOM 327 O GLN A 19 47.480 39.660 27.620 1.00 0.00 O

ATOM 328 CB GLN A 19 46.090 36.660 27.340 1.00 0.00 C

ATOM 329 CG GLN A 19 46.510 35.140 27.080 1.00 0.00 C

ATOM 330 CD GLN A 19 45.650 34.360 26.120 1.00 0.00 C

ATOM 331 NE2 GLN A 19 45.440 35.000 24.970 1.00 0.00 N

ATOM 332 OE1 GLN A 19 45.190 33.280 26.360 1.00 0.00 O

ATOM 333 HN GLN A 19 45.950 36.340 29.800 1.00 0.00 H

ATOM 334 HA GLN A 19 47.990 37.360 28.210 1.00 0.00 H

ATOM 335 HB1 GLN A 19 46.140 37.130 26.330 1.00 0.00 H

ATOM 336 HB2 GLN A 19 45.060 36.670 27.760 1.00 0.00 H

ATOM 337 HG1 GLN A 19 46.470 34.550 28.030 1.00 0.00 H

ATOM 338 HG2 GLN A 19 47.550 35.060 26.710 1.00 0.00 H

ATOM 339 HE21 GLN A 19 44.750 34.530 24.420 1.00 0.00 H

ATOM 340 HE22 GLN A 19 45.670 35.970 24.920 1.00 0.00 H

ATOM 341 N ALA A 20 45.560 39.450 28.640 1.00 0.00 N

ATOM 342 CA ALA A 20 45.340 40.880 28.550 1.00 0.00 C

ATOM 343 C ALA A 20 46.380 41.700 29.360 1.00 0.00 C

ATOM 344 O ALA A 20 46.910 42.700 28.920 1.00 0.00 O

ATOM 345 CB ALA A 20 43.890 41.300 28.980 1.00 0.00 C

ATOM 346 HN ALA A 20 44.960 38.790 29.090 1.00 0.00 H

ATOM 347 HA ALA A 20 45.360 41.140 27.500 1.00 0.00 H

ATOM 348 HB1 ALA A 20 43.740 40.950 30.020 1.00 0.00 H

ATOM 349 HB2 ALA A 20 43.050 40.860 28.400 1.00 0.00 H

ATOM 350 HB3 ALA A 20 43.760 42.410 28.990 1.00 0.00 H

ATOM 351 N MET A 21 46.610 41.310 30.580 1.00 0.00 N

ATOM 352 CA MET A 21 47.420 42.000 31.610 1.00 0.00 C

ATOM 353 C MET A 21 48.910 42.050 31.310 1.00 0.00 C

ATOM 354 O MET A 21 49.580 43.070 31.520 1.00 0.00 O

ATOM 355 CB MET A 21 47.110 41.540 33.120 1.00 0.00 C

ATOM 356 CG MET A 21 47.960 42.240 34.220 1.00 0.00 C

ATOM 357 SD MET A 21 47.620 43.980 34.390 1.00 0.00 S

ATOM 358 CE MET A 21 48.590 44.280 35.800 1.00 0.00 C

ATOM 359 HN MET A 21 46.090 40.540 30.960 1.00 0.00 H

ATOM 360 HA MET A 21 47.100 43.020 31.450 1.00 0.00 H

ATOM 361 HB1 MET A 21 47.400 40.470 33.110 1.00 0.00 H

ATOM 362 HB2 MET A 21 46.040 41.780 33.310 1.00 0.00 H

ATOM 363 HG1 MET A 21 49.000 42.070 33.890 1.00 0.00 H

ATOM 364 HG2 MET A 21 47.750 41.710 35.180 1.00 0.00 H

ATOM 365 HE1 MET A 21 48.400 43.540 36.600 1.00 0.00 H

ATOM 366 HE2 MET A 21 48.520 45.300 36.240 1.00 0.00 H

ATOM 367 HE3 MET A 21 49.640 44.090 35.490 1.00 0.00 H

ATOM 368 N ILE A 22 49.500 40.940 30.850 1.00 0.00 N

ATOM 369 CA ILE A 22 50.910 40.750 30.520 1.00 0.00 C

ATOM 370 C ILE A 22 51.130 40.710 28.970 1.00 0.00 C

ATOM 371 O ILE A 22 50.790 39.670 28.460 1.00 0.00 O

ATOM 372 CB ILE A 22 51.520 39.580 31.240 1.00 0.00 C

ATOM 373 CG1 ILE A 22 51.370 39.760 32.750 1.00 0.00 C

ATOM 374 CG2 ILE A 22 53.060 39.470 30.910 1.00 0.00 C

ATOM 375 CD ILE A 22 51.730 38.540 33.510 1.00 0.00 C

ATOM 376 HN ILE A 22 48.900 40.150 30.770 1.00 0.00 H

ATOM 377 HA ILE A 22 51.410 41.650 30.850 1.00 0.00 H

ATOM 378 HB ILE A 22 50.990 38.670 30.900 1.00 0.00 H

ATOM 379 HG11 ILE A 22 50.320 39.990 33.000 1.00 0.00 H

ATOM 380 HG12 ILE A 22 51.960 40.640 33.110 1.00 0.00 H

ATOM 381 HG21 ILE A 22 53.420 40.490 30.650 1.00 0.00 H

ATOM 382 HG22 ILE A 22 53.210 38.720 30.110 1.00 0.00 H

ATOM 383 HG23 ILE A 22 53.640 39.200 31.820 1.00 0.00 H

ATOM 384 HD1 ILE A 22 51.240 38.310 34.470 1.00 0.00 H

ATOM 385 HD2 ILE A 22 52.840 38.490 33.600 1.00 0.00 H

ATOM 386 HD3 ILE A 22 51.280 37.760 32.860 1.00 0.00 H

ATOM 387 N PRO A 23 51.680 41.680 28.190 1.00 0.00 N

ATOM 388 CA PRO A 23 51.650 41.580 26.680 1.00 0.00 C

ATOM 389 C PRO A 23 52.430 40.470 25.980 1.00 0.00 C

ATOM 390 O PRO A 23 52.100 40.120 24.850 1.00 0.00 O

ATOM 391 CB PRO A 23 52.090 42.990 26.200 1.00 0.00 C

ATOM 392 CG PRO A 23 52.900 43.550 27.420 1.00 0.00 C

ATOM 393 CD PRO A 23 52.140 43.000 28.660 1.00 0.00 C

ATOM 394 HA PRO A 23 50.620 41.420 26.410 1.00 0.00 H

ATOM 395 HB1 PRO A 23 51.210 43.660 26.150 1.00 0.00 H

ATOM 396 HB2 PRO A 23 52.720 42.980 25.280 1.00 0.00 H

ATOM 397 HG1 PRO A 23 53.850 42.980 27.330 1.00 0.00 H

ATOM 398 HG2 PRO A 23 53.030 44.650 27.330 1.00 0.00 H

ATOM 399 HD1 PRO A 23 52.730 42.930 29.600 1.00 0.00 H

ATOM 400 HD2 PRO A 23 51.210 43.570 28.830 1.00 0.00 H

ATOM 401 N LYS A 24 53.410 39.820 26.610 1.00 0.00 N

ATOM 402 CA LYS A 24 54.100 38.670 25.980 1.00 0.00 C

ATOM 403 C LYS A 24 54.600 37.710 27.070 1.00 0.00 C

ATOM 404 O LYS A 24 55.000 38.180 28.160 1.00 0.00 O

ATOM 405 CB LYS A 24 55.200 39.090 24.890 1.00 0.00 C

ATOM 406 CG LYS A 24 56.290 39.960 25.510 1.00 0.00 C

ATOM 407 CD LYS A 24 57.330 40.480 24.510 1.00 0.00 C

ATOM 408 CE LYS A 24 57.870 39.390 23.490 1.00 0.00 C

ATOM 409 NZ LYS A 24 58.770 39.940 22.390 1.00 0.00 N1+

ATOM 410 HN LYS A 24 53.790 40.310 27.390 1.00 0.00 H

ATOM 411 HA LYS A 24 53.290 38.190 25.460 1.00 0.00 H

ATOM 412 HB1 LYS A 24 54.700 39.650 24.070 1.00 0.00 H

ATOM 413 HB2 LYS A 24 55.690 38.170 24.510 1.00 0.00 H

ATOM 414 HG1 LYS A 24 56.750 39.270 26.250 1.00 0.00 H

ATOM 415 HG2 LYS A 24 55.960 40.830 26.120 1.00 0.00 H

ATOM 416 HD1 LYS A 24 58.200 41.060 24.900 1.00 0.00 H

ATOM 417 HD2 LYS A 24 56.760 41.190 23.870 1.00 0.00 H

ATOM 418 HE1 LYS A 24 57.070 38.910 22.890 1.00 0.00 H

ATOM 419 HE2 LYS A 24 58.450 38.600 24.010 1.00 0.00 H

ATOM 420 HZ1 LYS A 24 59.350 40.700 22.810 1.00 0.00 H

ATOM 421 HZ2 LYS A 24 58.240 40.310 21.580 1.00 0.00 H

ATOM 422 HZ3 LYS A 24 59.390 39.170 22.070 1.00 0.00 H

ATOM 423 N GLY A 25 54.780 36.360 26.850 1.00 0.00 N

ATOM 424 CA GLY A 25 55.070 35.360 27.850 1.00 0.00 C

ATOM 425 C GLY A 25 53.910 35.000 28.780 1.00 0.00 C

ATOM 426 O GLY A 25 52.900 35.670 28.990 1.00 0.00 O

ATOM 427 HN GLY A 25 54.820 36.020 25.910 1.00 0.00 H

ATOM 428 HA1 GLY A 25 55.830 35.830 28.460 1.00 0.00 H

ATOM 429 HA2 GLY A 25 55.360 34.400 27.440 1.00 0.00 H

ATOM 430 N GLY A 26 54.090 33.820 29.500 1.00 0.00 N

ATOM 431 CA GLY A 26 53.160 33.310 30.550 1.00 0.00 C

ATOM 432 C GLY A 26 51.990 32.600 29.960 1.00 0.00 C

ATOM 433 O GLY A 26 50.950 32.550 30.700 1.00 0.00 O

ATOM 434 HN GLY A 26 54.940 33.300 29.450 1.00 0.00 H

ATOM 435 HA1 GLY A 26 52.830 34.150 31.140 1.00 0.00 H

ATOM 436 HA2 GLY A 26 53.680 32.520 31.070 1.00 0.00 H

ATOM 437 N ARG A 27 52.050 32.130 28.740 1.00 0.00 N

ATOM 438 CA ARG A 27 50.890 31.460 28.170 1.00 0.00 C

ATOM 439 C ARG A 27 50.760 30.010 28.390 1.00 0.00 C

ATOM 440 O ARG A 27 49.670 29.440 28.670 1.00 0.00 O

ATOM 441 CB ARG A 27 50.880 31.760 26.640 1.00 0.00 C

ATOM 442 CG ARG A 27 50.270 33.130 26.330 1.00 0.00 C

ATOM 443 CD ARG A 27 51.110 34.450 26.610 1.00 0.00 C

ATOM 444 NE ARG A 27 50.490 35.670 26.040 1.00 0.00 N

ATOM 445 CZ ARG A 27 50.280 36.820 26.750 1.00 0.00 C

ATOM 446 NH1 ARG A 27 50.660 37.120 27.940 1.00 0.00 N1+

ATOM 447 NH2 ARG A 27 49.630 37.780 26.120 1.00 0.00 N

ATOM 448 HN ARG A 27 52.940 32.130 28.290 1.00 0.00 H

ATOM 449 HA ARG A 27 49.940 31.820 28.540 1.00 0.00 H

ATOM 450 HB1 ARG A 27 50.290 30.950 26.160 1.00 0.00 H

ATOM 451 HB2 ARG A 27 51.890 31.700 26.180 1.00 0.00 H

ATOM 452 HG1 ARG A 27 49.220 33.200 26.670 1.00 0.00 H

ATOM 453 HG2 ARG A 27 50.340 33.030 25.220 1.00 0.00 H

ATOM 454 HD1 ARG A 27 52.120 34.470 26.150 1.00 0.00 H

ATOM 455 HD2 ARG A 27 51.200 34.560 27.710 1.00 0.00 H

ATOM 456 HE ARG A 27 49.970 35.470 25.210 1.00 0.00 H

ATOM 457 HH11 ARG A 27 51.280 36.460 28.370 1.00 0.00 H

ATOM 458 HH12 ARG A 27 50.740 38.100 28.140 1.00 0.00 H

ATOM 459 HH21 ARG A 27 49.150 37.590 25.270 1.00 0.00 H

ATOM 460 HH22 ARG A 27 49.460 38.640 26.610 1.00 0.00 H

ATOM 461 N MET A 28 51.840 29.200 28.260 1.00 0.00 N

ATOM 462 CA MET A 28 51.840 27.790 28.460 1.00 0.00 C

ATOM 463 C MET A 28 51.480 27.390 29.850 1.00 0.00 C

ATOM 464 O MET A 28 50.490 26.680 30.010 1.00 0.00 O

ATOM 465 CB MET A 28 53.230 27.190 28.040 1.00 0.00 C

ATOM 466 CG MET A 28 53.720 25.840 28.520 1.00 0.00 C

ATOM 467 SD MET A 28 52.480 24.550 28.140 1.00 0.00 S

ATOM 468 CE MET A 28 52.350 24.830 26.290 1.00 0.00 C

ATOM 469 HN MET A 28 52.730 29.530 27.960 1.00 0.00 H

ATOM 470 HA MET A 28 51.100 27.340 27.810 1.00 0.00 H

ATOM 471 HB1 MET A 28 53.920 27.970 28.430 1.00 0.00 H

ATOM 472 HB2 MET A 28 53.280 27.250 26.930 1.00 0.00 H

ATOM 473 HG1 MET A 28 53.990 25.830 29.600 1.00 0.00 H

ATOM 474 HG2 MET A 28 54.630 25.690 27.910 1.00 0.00 H

ATOM 475 HE1 MET A 28 53.300 25.180 25.830 1.00 0.00 H

ATOM 476 HE2 MET A 28 51.580 25.610 26.140 1.00 0.00 H

ATOM 477 HE3 MET A 28 51.920 23.870 25.930 1.00 0.00 H

ATOM 478 N LEU A 29 52.260 27.910 30.810 1.00 0.00 N

ATOM 479 CA LEU A 29 52.050 27.500 32.220 1.00 0.00 C

ATOM 480 C LEU A 29 50.650 27.700 32.810 1.00 0.00 C

ATOM 481 O LEU A 29 50.220 26.760 33.380 1.00 0.00 O

ATOM 482 CB LEU A 29 53.190 28.080 33.180 1.00 0.00 C

ATOM 483 CG LEU A 29 53.030 27.810 34.640 1.00 0.00 C

ATOM 484 CD1 LEU A 29 52.950 26.320 35.060 1.00 0.00 C

ATOM 485 CD2 LEU A 29 54.210 28.430 35.380 1.00 0.00 C

ATOM 486 HN LEU A 29 52.930 28.620 30.650 1.00 0.00 H

ATOM 487 HA LEU A 29 52.250 26.440 32.230 1.00 0.00 H

ATOM 488 HB1 LEU A 29 53.280 29.180 33.020 1.00 0.00 H

ATOM 489 HB2 LEU A 29 54.230 27.780 32.930 1.00 0.00 H

ATOM 490 HG LEU A 29 52.080 28.210 35.050 1.00 0.00 H

ATOM 491 HD11 LEU A 29 52.820 26.300 36.170 1.00 0.00 H

ATOM 492 HD12 LEU A 29 53.920 25.840 34.840 1.00 0.00 H

ATOM 493 HD13 LEU A 29 52.120 25.760 34.560 1.00 0.00 H

ATOM 494 HD21 LEU A 29 55.160 27.950 35.090 1.00 0.00 H

ATOM 495 HD22 LEU A 29 54.080 28.220 36.470 1.00 0.00 H

ATOM 496 HD23 LEU A 29 54.270 29.530 35.250 1.00 0.00 H

ATOM 497 N PRO A 30 49.910 28.800 32.700 1.00 0.00 N

ATOM 498 CA PRO A 30 48.560 28.730 33.240 1.00 0.00 C

ATOM 499 C PRO A 30 47.650 27.830 32.470 1.00 0.00 C

ATOM 500 O PRO A 30 46.830 27.190 33.190 1.00 0.00 O

ATOM 501 CB PRO A 30 48.100 30.250 33.200 1.00 0.00 C

ATOM 502 CG PRO A 30 48.870 30.800 31.990 1.00 0.00 C

ATOM 503 CD PRO A 30 50.230 30.140 32.180 1.00 0.00 C

ATOM 504 HA PRO A 30 48.590 28.330 34.240 1.00 0.00 H

ATOM 505 HB1 PRO A 30 48.430 30.670 34.170 1.00 0.00 H

ATOM 506 HB2 PRO A 30 47.010 30.380 33.020 1.00 0.00 H

ATOM 507 HG1 PRO A 30 48.400 30.430 31.050 1.00 0.00 H

ATOM 508 HG2 PRO A 30 48.970 31.910 32.010 1.00 0.00 H

ATOM 509 HD1 PRO A 30 50.590 30.080 31.130 1.00 0.00 H

ATOM 510 HD2 PRO A 30 50.790 30.630 32.990 1.00 0.00 H

ATOM 511 N GLN A 31 47.680 27.670 31.090 1.00 0.00 N

ATOM 512 CA GLN A 31 46.830 26.780 30.340 1.00 0.00 C

ATOM 513 C GLN A 31 47.230 25.320 30.850 1.00 0.00 C

ATOM 514 O GLN A 31 46.320 24.590 31.260 1.00 0.00 O

ATOM 515 CB GLN A 31 47.170 26.810 28.850 1.00 0.00 C

ATOM 516 CG GLN A 31 46.080 26.470 27.860 1.00 0.00 C

ATOM 517 CD GLN A 31 45.380 25.120 28.090 1.00 0.00 C

ATOM 518 NE2 GLN A 31 45.890 23.980 27.390 1.00 0.00 N

ATOM 519 OE1 GLN A 31 44.350 25.070 28.710 1.00 0.00 O

ATOM 520 HN GLN A 31 48.390 28.180 30.600 1.00 0.00 H

ATOM 521 HA GLN A 31 45.830 27.020 30.650 1.00 0.00 H

ATOM 522 HB1 GLN A 31 48.080 26.220 28.610 1.00 0.00 H

ATOM 523 HB2 GLN A 31 47.400 27.900 28.750 1.00 0.00 H

ATOM 524 HG1 GLN A 31 46.520 26.590 26.850 1.00 0.00 H

ATOM 525 HG2 GLN A 31 45.180 27.100 28.010 1.00 0.00 H

ATOM 526 HE21 GLN A 31 45.290 23.190 27.250 1.00 0.00 H

ATOM 527 HE22 GLN A 31 46.640 24.150 26.750 1.00 0.00 H

ATOM 528 N LEU A 32 48.500 25.040 31.000 1.00 0.00 N

ATOM 529 CA LEU A 32 49.040 23.770 31.610 1.00 0.00 C

ATOM 530 C LEU A 32 48.460 23.320 32.980 1.00 0.00 C

ATOM 531 O LEU A 32 48.120 22.180 33.230 1.00 0.00 O

ATOM 532 CB LEU A 32 50.650 23.640 31.590 1.00 0.00 C

ATOM 533 CG LEU A 32 51.400 22.440 32.210 1.00 0.00 C

ATOM 534 CD1 LEU A 32 51.170 21.120 31.480 1.00 0.00 C

ATOM 535 CD2 LEU A 32 52.910 22.850 32.300 1.00 0.00 C

ATOM 536 HN LEU A 32 49.230 25.620 30.660 1.00 0.00 H

ATOM 537 HA LEU A 32 48.660 22.970 30.980 1.00 0.00 H

ATOM 538 HB1 LEU A 32 51.120 24.600 31.900 1.00 0.00 H

ATOM 539 HB2 LEU A 32 50.930 23.540 30.520 1.00 0.00 H

ATOM 540 HG LEU A 32 51.030 22.270 33.240 1.00 0.00 H

ATOM 541 HD11 LEU A 32 51.690 20.340 32.060 1.00 0.00 H

ATOM 542 HD12 LEU A 32 51.570 21.280 30.450 1.00 0.00 H

ATOM 543 HD13 LEU A 32 50.100 20.880 31.330 1.00 0.00 H

ATOM 544 HD21 LEU A 32 53.460 22.010 32.760 1.00 0.00 H

ATOM 545 HD22 LEU A 32 53.080 23.810 32.840 1.00 0.00 H

ATOM 546 HD23 LEU A 32 53.340 22.990 31.280 1.00 0.00 H

ATOM 547 N VAL A 33 48.290 24.250 33.920 1.00 0.00 N

ATOM 548 CA VAL A 33 47.780 23.900 35.240 1.00 0.00 C

ATOM 549 C VAL A 33 46.280 23.720 35.290 1.00 0.00 C

ATOM 550 O VAL A 33 45.770 22.800 35.850 1.00 0.00 O

ATOM 551 CB VAL A 33 48.050 24.930 36.350 1.00 0.00 C

ATOM 552 CG1 VAL A 33 47.380 24.520 37.700 1.00 0.00 C

ATOM 553 CG2 VAL A 33 49.600 25.130 36.350 1.00 0.00 C

ATOM 554 HN VAL A 33 48.520 25.200 33.750 1.00 0.00 H

ATOM 555 HA VAL A 33 48.160 22.930 35.520 1.00 0.00 H

ATOM 556 HB VAL A 33 47.660 25.910 36.000 1.00 0.00 H

ATOM 557 HG11 VAL A 33 47.620 23.470 37.980 1.00 0.00 H

ATOM 558 HG12 VAL A 33 46.280 24.600 37.680 1.00 0.00 H

ATOM 559 HG13 VAL A 33 47.780 25.180 38.510 1.00 0.00 H

ATOM 560 HG21 VAL A 33 49.960 25.500 35.370 1.00 0.00 H

ATOM 561 HG22 VAL A 33 50.110 24.170 36.610 1.00 0.00 H

ATOM 562 HG23 VAL A 33 49.850 25.830 37.170 1.00 0.00 H

ATOM 563 N CYS A 34 45.530 24.500 34.400 1.00 0.00 N

ATOM 564 CA CYS A 34 44.150 24.260 34.160 1.00 0.00 C

ATOM 565 C CYS A 34 43.910 22.820 33.530 1.00 0.00 C

ATOM 566 O CYS A 34 43.090 22.020 33.980 1.00 0.00 O

ATOM 567 CB CYS A 34 43.400 25.340 33.310 1.00 0.00 C

ATOM 568 SG CYS A 34 43.570 27.030 33.940 1.00 0.00 S

ATOM 569 HN CYS A 34 45.930 25.110 33.720 1.00 0.00 H

ATOM 570 HA CYS A 34 43.670 24.170 35.120 1.00 0.00 H

ATOM 571 HB1 CYS A 34 42.340 25.100 33.060 1.00 0.00 H

ATOM 572 HB2 CYS A 34 44.010 25.320 32.380 1.00 0.00 H

ATOM 573 N ARG A 35 44.630 22.570 32.380 1.00 0.00 N

ATOM 574 CA ARG A 35 44.580 21.330 31.640 1.00 0.00 C

ATOM 575 C ARG A 35 44.950 20.090 32.410 1.00 0.00 C

ATOM 576 O ARG A 35 44.210 19.100 32.410 1.00 0.00 O

ATOM 577 CB ARG A 35 45.300 21.610 30.300 1.00 0.00 C

ATOM 578 CG ARG A 35 45.390 20.390 29.360 1.00 0.00 C

ATOM 579 CD ARG A 35 43.970 19.870 29.010 1.00 0.00 C

ATOM 580 NE ARG A 35 44.240 18.810 27.970 1.00 0.00 N

ATOM 581 CZ ARG A 35 43.480 18.430 26.930 1.00 0.00 C

ATOM 582 NH1 ARG A 35 42.260 18.960 26.740 1.00 0.00 N1+

ATOM 583 NH2 ARG A 35 43.940 17.460 26.140 1.00 0.00 N

ATOM 584 HN ARG A 35 45.290 23.250 32.050 1.00 0.00 H

ATOM 585 HA ARG A 35 43.550 21.160 31.400 1.00 0.00 H

ATOM 586 HB1 ARG A 35 46.360 21.870 30.550 1.00 0.00 H

ATOM 587 HB2 ARG A 35 44.910 22.450 29.690 1.00 0.00 H

ATOM 588 HG1 ARG A 35 45.940 19.600 29.910 1.00 0.00 H

ATOM 589 HG2 ARG A 35 45.950 20.600 28.420 1.00 0.00 H

ATOM 590 HD1 ARG A 35 43.400 20.730 28.580 1.00 0.00 H

ATOM 591 HD2 ARG A 35 43.330 19.410 29.800 1.00 0.00 H

ATOM 592 HE ARG A 35 45.150 18.420 28.070 1.00 0.00 H

ATOM 593 HH11 ARG A 35 41.900 19.540 27.480 1.00 0.00 H

ATOM 594 HH12 ARG A 35 41.780 18.640 25.930 1.00 0.00 H

ATOM 595 HH21 ARG A 35 44.720 16.930 26.460 1.00 0.00 H

ATOM 596 HH22 ARG A 35 43.360 17.100 25.410 1.00 0.00 H

ATOM 597 N LEU A 36 46.040 20.190 33.290 1.00 0.00 N

ATOM 598 CA LEU A 36 46.540 19.160 34.150 1.00 0.00 C

ATOM 599 C LEU A 36 45.520 18.730 35.240 1.00 0.00 C

ATOM 600 O LEU A 36 45.300 17.550 35.450 1.00 0.00 O

ATOM 601 CB LEU A 36 47.970 19.570 34.750 1.00 0.00 C

ATOM 602 CG LEU A 36 48.630 18.680 35.840 1.00 0.00 C

ATOM 603 CD1 LEU A 36 49.060 17.380 35.180 1.00 0.00 C

ATOM 604 CD2 LEU A 36 49.910 19.350 36.390 1.00 0.00 C

ATOM 605 HN LEU A 36 46.730 20.910 33.250 1.00 0.00 H

ATOM 606 HA LEU A 36 46.700 18.240 33.600 1.00 0.00 H

ATOM 607 HB1 LEU A 36 47.870 20.570 35.230 1.00 0.00 H

ATOM 608 HB2 LEU A 36 48.700 19.780 33.950 1.00 0.00 H

ATOM 609 HG LEU A 36 47.910 18.430 36.640 1.00 0.00 H

ATOM 610 HD11 LEU A 36 50.150 17.220 35.030 1.00 0.00 H

ATOM 611 HD12 LEU A 36 48.440 17.120 34.300 1.00 0.00 H

ATOM 612 HD13 LEU A 36 48.750 16.580 35.890 1.00 0.00 H

ATOM 613 HD21 LEU A 36 50.440 18.630 37.050 1.00 0.00 H

ATOM 614 HD22 LEU A 36 49.740 20.260 36.990 1.00 0.00 H

ATOM 615 HD23 LEU A 36 50.630 19.690 35.610 1.00 0.00 H

ATOM 616 N VAL A 37 44.840 19.710 35.810 1.00 0.00 N

ATOM 617 CA VAL A 37 43.910 19.440 36.920 1.00 0.00 C

ATOM 618 C VAL A 37 42.440 19.240 36.490 1.00 0.00 C

ATOM 619 O VAL A 37 41.740 18.380 37.100 1.00 0.00 O

ATOM 620 CB VAL A 37 44.010 20.600 37.840 1.00 0.00 C

ATOM 621 CG1 VAL A 37 43.020 20.600 38.980 1.00 0.00 C

ATOM 622 CG2 VAL A 37 45.450 20.540 38.380 1.00 0.00 C

ATOM 623 HN VAL A 37 45.130 20.660 35.720 1.00 0.00 H

ATOM 624 HA VAL A 37 44.190 18.570 37.490 1.00 0.00 H

ATOM 625 HB VAL A 37 43.930 21.560 37.290 1.00 0.00 H

ATOM 626 HG11 VAL A 37 43.190 21.370 39.770 1.00 0.00 H

ATOM 627 HG12 VAL A 37 42.880 19.580 39.410 1.00 0.00 H

ATOM 628 HG13 VAL A 37 42.010 20.840 38.600 1.00 0.00 H

ATOM 629 HG21 VAL A 37 45.420 21.240 39.240 1.00 0.00 H

ATOM 630 HG22 VAL A 37 46.210 20.900 37.660 1.00 0.00 H

ATOM 631 HG23 VAL A 37 45.670 19.540 38.820 1.00 0.00 H

ATOM 632 N LEU A 38 41.960 19.920 35.460 1.00 0.00 N

ATOM 633 CA LEU A 38 40.580 19.880 35.050 1.00 0.00 C

ATOM 634 C LEU A 38 40.280 19.170 33.740 1.00 0.00 C

ATOM 635 O LEU A 38 39.120 19.070 33.360 1.00 0.00 O

ATOM 636 CB LEU A 38 39.940 21.290 35.160 1.00 0.00 C

ATOM 637 CG LEU A 38 40.220 21.960 36.540 1.00 0.00 C

ATOM 638 CD1 LEU A 38 39.720 23.390 36.260 1.00 0.00 C

ATOM 639 CD2 LEU A 38 39.540 21.250 37.750 1.00 0.00 C

ATOM 640 HN LEU A 38 42.480 20.650 35.020 1.00 0.00 H

ATOM 641 HA LEU A 38 40.040 19.310 35.800 1.00 0.00 H

ATOM 642 HB1 LEU A 38 38.850 21.200 34.960 1.00 0.00 H

ATOM 643 HB2 LEU A 38 40.350 21.860 34.300 1.00 0.00 H

ATOM 644 HG LEU A 38 41.310 22.100 36.720 1.00 0.00 H

ATOM 645 HD11 LEU A 38 38.690 23.370 35.840 1.00 0.00 H

ATOM 646 HD12 LEU A 38 40.290 23.860 35.430 1.00 0.00 H

ATOM 647 HD13 LEU A 38 39.750 24.050 37.150 1.00 0.00 H

ATOM 648 HD21 LEU A 38 39.530 21.810 38.710 1.00 0.00 H

ATOM 649 HD22 LEU A 38 40.120 20.360 38.070 1.00 0.00 H

ATOM 650 HD23 LEU A 38 38.500 20.990 37.450 1.00 0.00 H

ATOM 651 N ARG A 39 41.360 18.790 33.020 1.00 0.00 N

ATOM 652 CA ARG A 39 41.250 17.800 31.950 1.00 0.00 C

ATOM 653 C ARG A 39 40.500 18.140 30.690 1.00 0.00 C

ATOM 654 O ARG A 39 40.620 17.520 29.670 1.00 0.00 O

ATOM 655 CB ARG A 39 40.690 16.470 32.540 1.00 0.00 C

ATOM 656 CG ARG A 39 41.600 15.920 33.770 1.00 0.00 C

ATOM 657 CD ARG A 39 43.150 16.190 33.670 1.00 0.00 C

ATOM 658 NE ARG A 39 43.570 15.420 32.460 1.00 0.00 N

ATOM 659 CZ ARG A 39 44.810 15.240 32.130 1.00 0.00 C

ATOM 660 NH1 ARG A 39 45.860 15.570 32.840 1.00 0.00 N1+

ATOM 661 NH2 ARG A 39 45.070 14.570 31.020 1.00 0.00 N

ATOM 662 HN ARG A 39 42.270 19.030 33.340 1.00 0.00 H

ATOM 663 HA ARG A 39 42.220 17.760 31.480 1.00 0.00 H

ATOM 664 HB1 ARG A 39 40.680 15.670 31.780 1.00 0.00 H

ATOM 665 HB2 ARG A 39 39.720 16.730 33.020 1.00 0.00 H

ATOM 666 HG1 ARG A 39 41.450 14.820 33.740 1.00 0.00 H

ATOM 667 HG2 ARG A 39 41.350 16.320 34.770 1.00 0.00 H

ATOM 668 HD1 ARG A 39 43.730 15.850 34.560 1.00 0.00 H

ATOM 669 HD2 ARG A 39 43.290 17.280 33.530 1.00 0.00 H

ATOM 670 HE ARG A 39 42.880 14.840 32.030 1.00 0.00 H

ATOM 671 HH11 ARG A 39 45.720 16.100 33.670 1.00 0.00 H

ATOM 672 HH12 ARG A 39 46.740 15.700 32.390 1.00 0.00 H

ATOM 673 HH21 ARG A 39 44.260 14.260 30.530 1.00 0.00 H

ATOM 674 HH22 ARG A 39 45.960 14.700 30.580 1.00 0.00 H

ATOM 675 N CYS A 40 39.700 19.170 30.640 1.00 0.00 N

ATOM 676 CA CYS A 40 38.940 19.690 29.470 1.00 0.00 C

ATOM 677 C CYS A 40 39.800 20.180 28.250 1.00 0.00 C

ATOM 678 O CYS A 40 40.920 20.590 28.460 1.00 0.00 O

ATOM 679 CB CYS A 40 38.060 20.930 29.790 1.00 0.00 C

ATOM 680 SG CYS A 40 36.790 20.810 31.100 1.00 0.00 S

ATOM 681 HN CYS A 40 39.550 19.730 31.450 1.00 0.00 H

ATOM 682 HA CYS A 40 38.300 18.880 29.160 1.00 0.00 H

ATOM 683 HB1 CYS A 40 37.470 21.330 28.940 1.00 0.00 H

ATOM 684 HB2 CYS A 40 38.790 21.710 30.110 1.00 0.00 H

ATOM 685 N SER A 41 39.190 20.390 27.030 1.00 0.00 N

ATOM 686 CA SER A 41 39.750 21.030 25.840 1.00 0.00 C

ATOM 687 C SER A 41 39.660 22.500 26.130 1.00 0.00 C

ATOM 688 CB SER A 41 38.960 20.760 24.500 1.00 0.00 C

ATOM 689 OG SER A 41 38.830 19.360 24.210 1.00 0.00 O

ATOM 690 OT1 SER A 41 40.600 23.130 25.670 1.00 0.00 O

ATOM 691 OT2 SER A 41 38.710 22.980 26.780 1.00 0.00 O

ATOM 692 HN SER A 41 38.240 20.090 26.960 1.00 0.00 H

ATOM 693 HA SER A 41 40.800 20.800 25.700 1.00 0.00 H

ATOM 694 HB1 SER A 41 39.490 21.200 23.630 1.00 0.00 H

ATOM 695 HB2 SER A 41 37.980 21.280 24.600 1.00 0.00 H

ATOM 696 HG1 SER A 41 39.720 19.040 24.060 1.00 0.00 H

TER

END

ModelArchive (<https://modelarchive.org>/doi/10.5452/ma-p57c0) > Procedures & Data

**Molecular Dynamics Refinement of SMB Peptide Structure based on MS and FTIR Residue Specific Experimental Data**

Molecular simulations were run on a custom desk top computer cluster fitted with a 3.10 GHz Intel Xeon E5-2687W CPU, 256 GB of RAM, SSD Storage and a 2080Ti NVIDIA GPU board (<https://responsiveweb.io/>). The custom system was configured with ubuntu version 22**.**

MD simulations were carried out using the Charmm 36m all atom force field implementation for lipids and proteins in the [Gromacs](http://manual.gromacs.org/documentation/5.1.1/user-guide/environment-variables.html) (Version 2020.3) environment (http://www.gromacs.org). The system was first minimized using a steepest descent strategy followed by a six-step equilibration process at 311^o^K for a total of 100 ns. This included both NVT (constant number, volume, temperature) and NPT (constant number, pressure, temperature) equilibration phases to allow water molecules to reorient around the lipid headgroups and any exposed parts of the peptide, as well as permitting lipids to optimize their orientation around the peptide. Equilibration protocols employed a PME (Particle Mesh Ewald) strategy for Coulombic long-range interactions and Berendsen temperature coupling. A Berendsen strategy was also used for pressure coupling in a semi-isotropic mode to emulate bilayer motion. After equilibration, the system was subjected to a dynamics production run at the same temperature using the Nose-Hoover protocol and pressure (Parrinello-Rahman) values used in the pre-run steps The Verlet cut-off scheme was employed for all minimization, equilibration, and production steps. Detailed protocols and parameter files for this type of membrane simulation are available from the [Charmm-GUI website](http://www.charmm-gui.org/): (http://www.charmm-gui.org). The output of the production run simulations was analyzed with the Gromacs suite of analysis tools.

The structural quality of the molecular dynamics refined structures was analyzed by PROCHECK (Laskowski et al., 1993, 1996) generated with PDBsum (https://ebi.ac.uk). Molecular graphics were rendered using Pymol Version 2.2.3.

Using the combination of the disulfide linkages and ^13^C measurements constraints followed by molecular dynamics refinement, 10 molecular models representing possible conformations of SMB in simulated surfactant lipid bilayers were generated. The lowest energy conformer is shown in as a ribbon-cartoon representation of the lowest energy conformer of the peptide in the surfactant multilayer environment. Geometry parameters of the final models were evaluated using PROCHECK (Laskowski et. al., 1993, 1996) to generate a Ramachandran plot that confirms the backbone torsion angles for the well-defined domains fall within the most favored regions of alpha helix (A labeled core highlighted in red. High levels of backbone conformations associated with alpha helical sequences include residues 6 to 21 in the N-terminal domain while amino acid residues 30 to 37 define the C-terminal domain. Other sequences in the refined molecular model include the more disordered N-terminal insertion sequence (residues Phe-1 to Leu-5) and the bend region including residues 22 to 29 bridging the N-terminal – C-terminal helical domains to form the helix-hairpin structure.

CHARMM-GUI (<http://www.charmm-gui.org>) command script (readme.csh) generated to run Gromacs simulation on computer cluster.

#!/bin/csh

#

# Generated by CHARMM-GUI (http://www.charmm-gui.org) v3.7

# This folder contains GROMACS formatted CHARMM36 force fields, a pre-optimized PDB structure, and GROMACS inputs.

# All input files were optimized for GROMACS 2019.2 or above, so lower version of GROMACS can cause some errors.

# We adopted the Verlet cut-off scheme for all minimization, equilibration, and production steps because it is

# faster and more accurate than the group scheme. If you have a trouble with a performance of Verlet scheme while

# running parallelized simulation, you should check if you are using appropriate command line.

# For MPI parallelizing, we recommand following command:

# mpirun -np $NUM_CPU gmx mdrun -ntomp 1

set init = step5_input

set rest_prefix = step5_input

set mini_prefix = step6.0_minimization

set equi_prefix = step6.%d_equilibration

set prod_prefix = step7_production

set prod_step = step7

# Minimization

# In the case that there is a problem during minimization using a single precision of GROMACS, please try to use

# a double precision of GROMACS only for the minimization step.

gmx grompp -f ${mini_prefix}.mdp -o ${mini_prefix}.tpr -c ${init}.gro -r ${rest_prefix}.gro -p topol.top -n index.ndx

gmx_d mdrun -v -deffnm ${mini_prefix}

# Equilibration

set cnt = 1

set cntmax = 6

while ( ${cnt} <= ${cntmax} )

@ pcnt = ${cnt} - 1

set istep = `printf ${equi_prefix} ${cnt}`

set pstep = `printf ${equi_prefix} ${pcnt}`

if ( ${cnt} == 1 ) set pstep = ${mini_prefix}

gmx grompp -f ${istep}.mdp -o ${istep}.tpr -c ${pstep}.gro -r ${rest_prefix}.gro -p topol.top -n index.ndx

gmx mdrun -v -deffnm ${istep}

@ cnt += 1

end

# Production

set cnt = 1

set cntmax = 10

while ( ${cnt} <= ${cntmax} )

@ pcnt = ${cnt} - 1

set istep = ${prod_step}_${cnt}

set pstep = ${prod_step}_${pcnt}

if ( ${cnt} == 1 ) then

set pstep = `printf ${equi_prefix} 6`

gmx grompp -f ${prod_prefix}.mdp -o ${istep}.tpr -c ${pstep}.gro -p topol.top -n index.ndx

else

gmx grompp -f ${prod_prefix}.mdp -o ${istep}.tpr -c ${pstep}.gro -t ${pstep}.cpt -p topol.top -n index.ndx

endif

gmx mdrun -v -deffnm ${istep}

@ cnt += 1

end

**Analysis of Molecular Simulation**


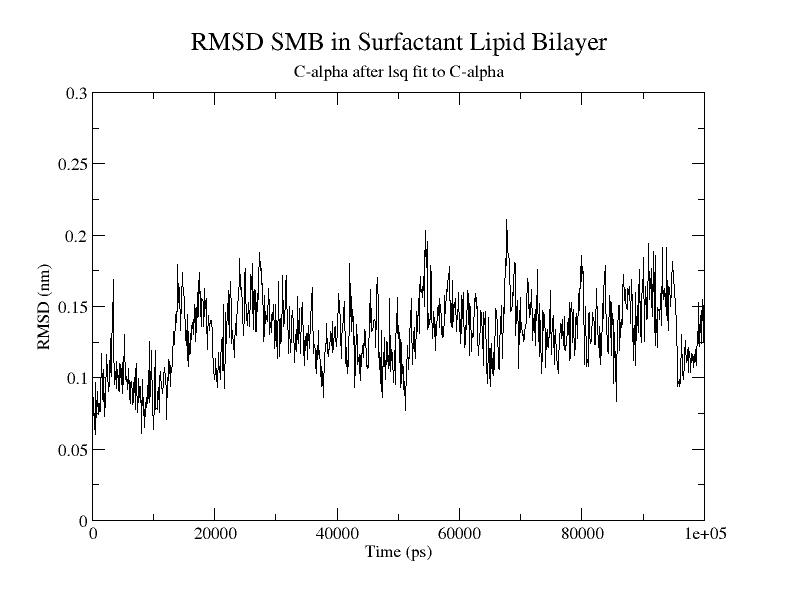


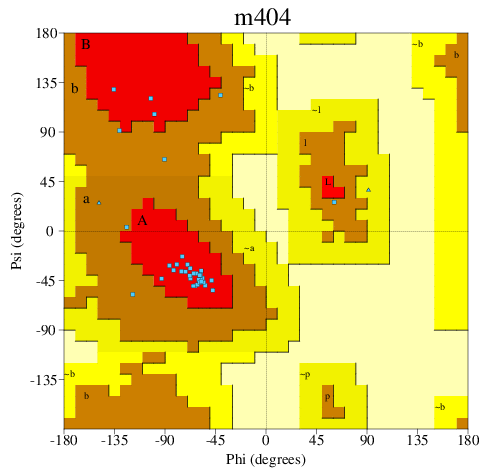


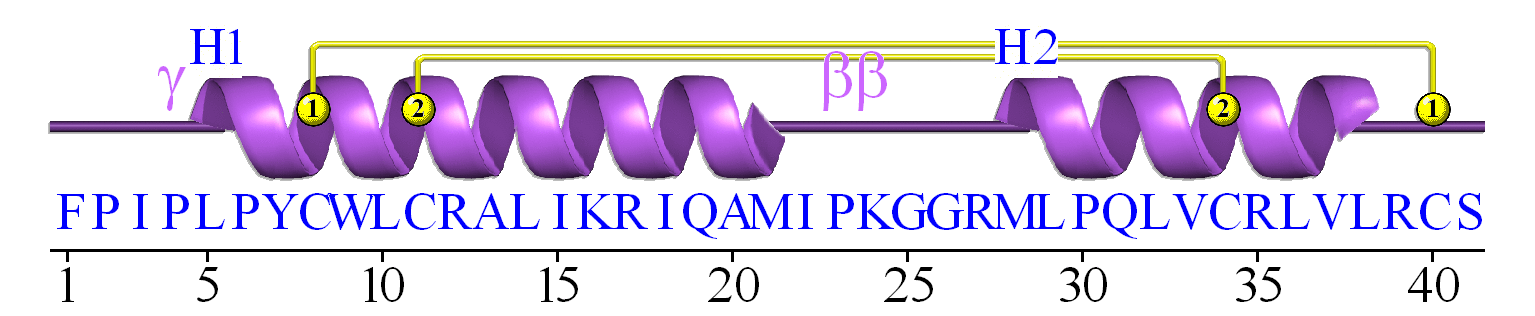


| **Secondary structure summary **   \|  \| \| \| \| \| \| \| \| \| \| --- \| --- \| --- \| --- \| --- \| --- \| --- \| --- \| --- \| \| **Strand** \|  \| **Alpha helix** \|  \| **3-10 helix** \|  \| **Other** \|  \| **Total residues** \| \|  \| \| \| \| \| \| \| \| \| \| 0 (0.0%) \|  \| 28 (68.3%) \|  \| 0 (0.0%) \|  \| 13 (31.7%) \|  \| 41 \|  \| \|  \| \| \| \| \| \| \| \| \|  \| |
| --- | --- | --- | --- | --- | --- | --- | --- | --- | --- | --- | --- | --- | --- | --- | --- | --- | --- | --- | --- | --- | --- | --- | --- | --- | --- | --- | --- | --- | --- | --- | --- | --- | --- | --- | --- | --- | --- | --- | --- | --- | --- | --- | --- | --- | --- | --- | --- |
|  |
| [**2 helices**](https://www.ebi.ac.uk/thornton-srv/databases/cgi-bin/pdbsum/GetPage.pl?pdbcode=m404&pdb_type=UPLOAD&code=162501&template=protein.html&o=HELICES&r=psplot&l=1&c=2&pdb_type=UPLOAD&code=162501&chain=A) ****   \|  \| \| \| \| \| \| \| \| --- \| --- \| --- \| --- \| --- \| --- \| --- \| \| **Start** \|  \| **End** \|  \| **Type** \|  \| **No. resid** \| \|  \| \| \| \| \| \| \| \| Leu5 \|  \| Met21 \|  \| H \|  \| 17 \| \| Met28 \|  \| Leu38 \|  \| H \|  \| 11 \| \|  \| \| \| \| \| \| \| |
|  |
| [**1 helix-helix interaction**](https://www.ebi.ac.uk/thornton-srv/databases/cgi-bin/pdbsum/GetPage.pl?pdbcode=m404&pdb_type=UPLOAD&code=162501&template=protein.html&o=HELIX_INTERACTIONS&l=1&c=3&pdb_type=UPLOAD&code=162501&chain=A) ****   \|  \| \| \| \| \| \| \| \| \| \| \| \| \| --- \| --- \| --- \| --- \| --- \| --- \| --- \| --- \| --- \| --- \| --- \| --- \| \|  \| \|  \| **Helix** \| \|  \| **Interaction** \| \|  \| **No. interacting residues** \| \| \| \|  \| \|  \|  \| \|  \|  \| \|  \|  \| \| \| \| **Helices** \| \|  \| **types** \| \|  \| **type** \| \|  \| **Helix 1** \|  \| **Helix 2** \| \|  \| \| \| \| \| \| \| \| \| \| \| \| \| A1 \| A2 \|  \| H \| H \|  \| C \| N \|  \| 7 \|  \| 4 \| \|  \| \| \| \| \| \| \| \| \| \| \| \| |
|  |
| [**2 beta turns**](https://www.ebi.ac.uk/thornton-srv/databases/cgi-bin/pdbsum/GetPage.pl?pdbcode=m404&pdb_type=UPLOAD&code=162501&template=protein.html&o=BETA_TURNS&r=psplot&l=1&s=1&c=4&pdb_type=UPLOAD&code=162501&chain=A&pdb_type=UPLOAD&code=162501&chain=A) ****   \|  \| \| \| \| \| \| \| \| --- \| --- \| --- \| --- \| --- \| --- \| --- \| \|  \|  \|  \|  \| **Turn** \|  \|  \| \| **Turn** \|  \| **Sequence** \|  \| **type** \|  \| **H-bond** \| \|  \| \| \| \| \| \| \| \| Ile22-Gly25 \|  \| IPKG \|  \| VIII \|  \|  \| \| Pro23-Gly26 \|  \| PKGG \|  \| IV \|  \|  \| \|  \| \| \| \| \| \| \| |
|  |
| [**1 gamma turn**](https://www.ebi.ac.uk/thornton-srv/databases/cgi-bin/pdbsum/GetPage.pl?pdbcode=m404&pdb_type=UPLOAD&code=162501&template=protein.html&o=GAMMA_TURNS&r=psplot&l=1&c=5&pdb_type=UPLOAD&code=162501&chain=A) ****   \|  \| \| \| \| \| \| \| \| --- \| --- \| --- \| --- \| --- \| --- \| --- \| \| **Start** \|  \| **End** \|  \| **Sequence** \|  \| **Turn type** \| \|  \| \| \| \| \| \| \| \| Ile3 \|  \| Leu5 \|  \| IPL \|  \| INVERSE \| \|  \| \| \| \| \| \| \| |
|  |
| [**2 disulphides**](https://www.ebi.ac.uk/thornton-srv/databases/cgi-bin/pdbsum/GetPage.pl?pdbcode=m404&pdb_type=UPLOAD&code=162501&template=protein.html&o=DISULPHIDES&l=1&c=6&pdb_type=UPLOAD&code=162501&chain=A) ****   \|  \| \| \| \| \| \| --- \| --- \| --- \| --- \| --- \| \| **1st cysteine** \|  \| **2nd cysteine** \|  \| **Type** \| \|  \| \| \| \| \| \| A 8 \|  \| A 40 \|  \| RHS \| \| A 11 \|  \| A 34 \|  \| LHS \| \|  \| \| \| \| \| |
|  |
| Table of helices |
|  |
| \|  \| \| \| \| \| \| \| \| \| \| \| \| \| \| \| \| \| \| \| \| \| \| \| \| --- \| --- \| --- \| --- \| --- \| --- \| --- \| --- \| --- \| --- \| --- \| --- \| --- \| --- \| --- \| --- \| --- \| --- \| --- \| --- \| --- \| --- \| --- \| \| **No.** \|  \| **Start** \|  \| **End** \|  \| **Type** \|  \| **No. resid** \|  \| **Length** \|  \| **Unit rise** \|  \| **Residues per turn** \|  \| **Pitch** \|  \| **Deviation from ideal** \|  \| **Sequence** \|  \|  \| \|  \| \| \| \| \| \| \| \| \| \| \| \| \| \| \| \| \| \| \| \| \| \| \| \| 1. \|  \| Leu5 \|  \| Met21 \|  \| H \|  \| 17 \|  \| 26.08 \|  \| 1.52 \|  \| 3.58 \|  \| 5.44 \|  \| 4.2 \|  \| LPYCWLCRALIKRIQAM \|  \|  \| \| 2. \|  \| Met28 \|  \| Leu38 \|  \| H \|  \| 11 \|  \| 15.19 \|  \| 1.39 \|  \| 3.81 \|  \| 5.30 \|  \| 8.9 \|  \| MLPQLVCRLVL \|  \|  \| \|  \| \| \| \| \| \| \| \| \| \| \| \| \| \| \| \| \| \| \| \| \| \| \| |
|  |
|  |
|  |
|  |

Laskowski R A, Rullmann, J A C, MacArthur M W, Kaptein R, Thornton J.M. AQUA and 1996. PROCHECK-NMR: Programs for checking the quality of protein structures solved by NMR. Journal of Biomolecular NMR, 1996; 8:477- 496. PMID: **9008363**

Laskowski, R.A., MacArthur, M.W., Moss D.S. and Thornton, J.M. 1993. PROCHECK: a program to check the stereochemical quality of protein structures. J. Appl. Cryst., 1993; 26, 283-291.
