# Supplementary material for: Emulation of the structure of the Saposin protein fold by a lung surfactant peptide construct of surfactant Protein B
Source: PLoS One. 2022 Nov 3;17(11):e0276787. doi: 10.1371/journal.pone.0276787 (PMC9632872; doi:10.1371/journal.pone.0276787)
Supplement: S5 File — (DOCX) [file pone.0276787.s005.docx]

**S5** **- deposited in the ModelArchive (**<https://modelarchive.org>/doi/10.5452/ma-6jm8t**)**

**Emulation of the Structure of the Saposin Protein Fold by a Lung Surfactant Peptide Construct of Surfactant Protein B**

Alan J. Waring^1,2^, Julian P. Whitelegge^3^, Shantanu K. Sharma^4^, Larry M. Gordon^1^,

Frans J. Walther^1,5,*^

^1^ Lundquist Institute for Biomedical Innovation at Harbor-UCLA Medical Center

1124 West Carson Street

Torrance, CA, USA

^2^ Department of Medicine

David Geffen School of Medicine

University of California Los Angeles

405 Hilgard Avenue

Los Angeles, CA, USA

^3^ NPI-Semel Institute for Neuroscience & Human Behavior Department of Psychiatry

& Biobehavioral Sciences, David Geffen School of Medicine at UCLA,

760 Westwood Plaza, Los Angeles, CA, USA

^4^ Materials and Process Simulation Center

California Institute of Technology

1200 East California Boulevard

Pasadena, CA, USA

^5^ Department of Pediatrics

David Geffen School of Medicine

University of California Los Angeles

405 Hilgard Avenue

Los Angeles, CA, USA

# Human SP-B AlphaFold Deposition Data Predicted SP-B Monomer Structure AI Modeling using AlphaFold

ModelArchive (<https://modelarchive.org>/doi/10.5452/ma-6jm8t)

SEQRES 1 A 79 PHE PRO ILE PRO LEU PRO TYR CYS TRP LEU CYS ARG ALA

SEQRES 2 A 79 LEU ILE LYS ARG ILE GLN ALA MET ILE PRO LYS GLY ALA

SEQRES 3 A 79 LEU ARG VAL ALA VAL ALA GLN VAL CYS ARG VAL VAL PRO

SEQRES 4 A 79 LEU VAL ALA GLY GLY ILE CYS GLN CYS LEU ALA GLU ARG

SEQRES 5 A 79 TYR SER VAL ILE LEU LEU ASP THR LEU LEU GLY ARG MET

SEQRES 6 A 79 LEU PRO GLN LEU VAL CYS ARG LEU VAL LEU ARG CYS SER

SEQRES 7 A 79 MET

HELIX 1 1 LEU A 5 MET A 21 1 17

HELIX 2 2 LYS A 24 GLN A 33 1 10

HELIX 3 3 VAL A 34 ARG A 36 1 3

HELIX 4 4 VAL A 41 ARG A 64 1 24

HELIX 5 5 MET A 65 VAL A 74 1 10

ATOM 1 N PHE A 1 8.347 14.357 -22.415 1.00 43.72 N

ATOM 2 CA PHE A 1 9.344 13.310 -22.124 1.00 43.72 C

ATOM 3 C PHE A 1 8.637 12.105 -21.522 1.00 43.72 C

ATOM 4 O PHE A 1 8.192 12.218 -20.383 1.00 43.72 O

ATOM 5 CB PHE A 1 10.426 13.841 -21.166 1.00 43.72 C

ATOM 6 CG PHE A 1 11.683 14.272 -21.886 1.00 43.72 C

ATOM 7 CD1 PHE A 1 12.704 13.327 -22.105 1.00 43.72 C

ATOM 8 CD2 PHE A 1 11.840 15.595 -22.347 1.00 43.72 C

ATOM 9 CE1 PHE A 1 13.875 13.700 -22.784 1.00 43.72 C

ATOM 10 CE2 PHE A 1 13.012 15.962 -23.033 1.00 43.72 C

ATOM 11 CZ PHE A 1 14.028 15.015 -23.251 1.00 43.72 C

ATOM 12 H2 PHE A 1 8.806 15.167 -22.807 1.00 43.72 H

ATOM 13 H PHE A 1 7.660 14.013 -23.072 1.00 43.72 H

ATOM 14 H3 PHE A 1 7.883 14.619 -21.557 1.00 43.72 H

ATOM 15 HA PHE A 1 9.834 12.994 -23.045 1.00 43.72 H

ATOM 16 HB2 PHE A 1 10.708 13.054 -20.467 1.00 43.72 H

ATOM 17 HB3 PHE A 1 10.037 14.667 -20.571 1.00 43.72 H

ATOM 18 HD1 PHE A 1 12.602 12.313 -21.748 1.00 43.72 H

ATOM 19 HD2 PHE A 1 11.084 16.344 -22.163 1.00 43.72 H

ATOM 20 HE1 PHE A 1 14.663 12.979 -22.946 1.00 43.72 H

ATOM 21 HE2 PHE A 1 13.146 16.975 -23.381 1.00 43.72 H

ATOM 22 HZ PHE A 1 14.931 15.303 -23.769 1.00 43.72 H

ATOM 23 N PRO A 2 8.459 10.986 -22.247 1.00 52.22 N

ATOM 24 CA PRO A 2 8.131 9.729 -21.585 1.00 52.22 C

ATOM 25 C PRO A 2 9.308 9.381 -20.666 1.00 52.22 C

ATOM 26 O PRO A 2 10.460 9.384 -21.101 1.00 52.22 O

ATOM 27 CB PRO A 2 7.901 8.711 -22.705 1.00 52.22 C

ATOM 28 CG PRO A 2 8.755 9.240 -23.859 1.00 52.22 C

ATOM 29 CD PRO A 2 8.796 10.756 -23.648 1.00 52.22 C

ATOM 30 HA PRO A 2 7.221 9.829 -20.993 1.00 52.22 H

ATOM 31 HB2 PRO A 2 8.198 7.704 -22.411 1.00 52.22 H

ATOM 32 HB3 PRO A 2 6.851 8.725 -22.996 1.00 52.22 H

ATOM 33 HG2 PRO A 2 9.765 8.837 -23.774 1.00 52.22 H

ATOM 34 HG3 PRO A 2 8.327 8.978 -24.827 1.00 52.22 H

ATOM 35 HD2 PRO A 2 8.050 11.231 -24.285 1.00 52.22 H

ATOM 36 HD3 PRO A 2 9.791 11.129 -23.888 1.00 52.22 H

ATOM 37 N ILE A 3 9.037 9.180 -19.375 1.00 57.63 N

ATOM 38 CA ILE A 3 10.055 8.742 -18.419 1.00 57.63 C

ATOM 39 C ILE A 3 10.583 7.393 -18.930 1.00 57.63 C

ATOM 40 O ILE A 3 9.769 6.536 -19.286 1.00 57.63 O

ATOM 41 CB ILE A 3 9.472 8.614 -16.990 1.00 57.63 C

ATOM 42 CG1 ILE A 3 8.936 9.937 -16.395 1.00 57.63 C

ATOM 43 CG2 ILE A 3 10.527 8.092 -16.006 1.00 57.63 C

ATOM 44 CD1 ILE A 3 7.515 10.299 -16.829 1.00 57.63 C

ATOM 45 H ILE A 3 8.075 9.203 -19.069 1.00 57.63 H

ATOM 46 HA ILE A 3 10.856 9.481 -18.410 1.00 57.63 H

ATOM 47 HB ILE A 3 8.663 7.884 -17.010 1.00 57.63 H

ATOM 48 HG12 ILE A 3 8.898 9.846 -15.309 1.00 57.63 H

ATOM 49 HG13 ILE A 3 9.614 10.757 -16.632 1.00 57.63 H

ATOM 50 HG21 ILE A 3 10.946 7.150 -16.358 1.00 57.63 H

ATOM 51 HG22 ILE A 3 10.043 7.896 -15.049 1.00 57.63 H

ATOM 52 HG23 ILE A 3 11.323 8.824 -15.867 1.00 57.63 H

ATOM 53 HD11 ILE A 3 6.866 9.426 -16.772 1.00 57.63 H

ATOM 54 HD12 ILE A 3 7.124 11.080 -16.176 1.00 57.63 H

ATOM 55 HD13 ILE A 3 7.516 10.682 -17.850 1.00 57.63 H

ATOM 56 N PRO A 4 11.906 7.172 -18.994 1.00 65.51 N

ATOM 57 CA PRO A 4 12.437 5.901 -19.457 1.00 65.51 C

ATOM 58 C PRO A 4 11.892 4.766 -18.574 1.00 65.51 C

ATOM 59 O PRO A 4 12.006 4.812 -17.347 1.00 65.51 O

ATOM 60 CB PRO A 4 13.962 6.076 -19.437 1.00 65.51 C

ATOM 61 CG PRO A 4 14.202 7.188 -18.416 1.00 65.51 C

ATOM 62 CD PRO A 4 12.974 8.073 -18.593 1.00 65.51 C

ATOM 63 HA PRO A 4 12.117 5.733 -20.486 1.00 65.51 H

ATOM 64 HB2 PRO A 4 14.291 6.419 -20.417 1.00 65.51 H

ATOM 65 HB3 PRO A 4 14.494 5.161 -19.174 1.00 65.51 H

ATOM 66 HG2 PRO A 4 14.210 6.768 -17.410 1.00 65.51 H

ATOM 67 HG3 PRO A 4 15.126 7.731 -18.615 1.00 65.51 H

ATOM 68 HD2 PRO A 4 12.752 8.586 -17.657 1.00 65.51 H

ATOM 69 HD3 PRO A 4 13.149 8.799 -19.387 1.00 65.51 H

ATOM 70 N LEU A 5 11.317 3.736 -19.213 1.00 68.92 N

ATOM 71 CA LEU A 5 10.812 2.499 -18.594 1.00 68.92 C

ATOM 72 C LEU A 5 11.675 1.938 -17.439 1.00 68.92 C

ATOM 73 O LEU A 5 11.078 1.470 -16.461 1.00 68.92 O

ATOM 74 CB LEU A 5 10.636 1.418 -19.681 1.00 68.92 C

ATOM 75 CG LEU A 5 9.457 1.688 -20.644 1.00 68.92 C

ATOM 76 CD1 LEU A 5 9.944 1.818 -22.087 1.00 68.92 C

ATOM 77 CD2 LEU A 5 8.426 0.562 -20.584 1.00 68.92 C

ATOM 78 H LEU A 5 11.192 3.830 -20.211 1.00 68.92 H

ATOM 79 HA LEU A 5 9.832 2.714 -18.169 1.00 68.92 H

ATOM 80 HB2 LEU A 5 11.566 1.316 -20.240 1.00 68.92 H

ATOM 81 HB3 LEU A 5 10.466 0.464 -19.183 1.00 68.92 H

ATOM 82 HG LEU A 5 8.964 2.620 -20.367 1.00 68.92 H

ATOM 83 HD11 LEU A 5 9.104 2.075 -22.732 1.00 68.92 H

ATOM 84 HD12 LEU A 5 10.692 2.608 -22.158 1.00 68.92 H

ATOM 85 HD13 LEU A 5 10.376 0.877 -22.426 1.00 68.92 H

ATOM 86 HD21 LEU A 5 8.878 -0.388 -20.870 1.00 68.92 H

ATOM 87 HD22 LEU A 5 7.605 0.782 -21.267 1.00 68.92 H

ATOM 88 HD23 LEU A 5 8.018 0.486 -19.576 1.00 68.92 H

ATOM 89 N PRO A 6 13.029 2.003 -17.448 1.00 77.47 N

ATOM 90 CA PRO A 6 13.826 1.588 -16.291 1.00 77.47 C

ATOM 91 C PRO A 6 13.458 2.303 -14.986 1.00 77.47 C

ATOM 92 O PRO A 6 13.461 1.663 -13.939 1.00 77.47 O

ATOM 93 CB PRO A 6 15.293 1.850 -16.664 1.00 77.47 C

ATOM 94 CG PRO A 6 15.209 2.848 -17.812 1.00 77.47 C

ATOM 95 CD PRO A 6 13.956 2.353 -18.523 1.00 77.47 C

ATOM 96 HA PRO A 6 13.693 0.518 -16.131 1.00 77.47 H

ATOM 97 HB2 PRO A 6 15.869 2.250 -15.830 1.00 77.47 H

ATOM 98 HB3 PRO A 6 15.748 0.926 -17.020 1.00 77.47 H

ATOM 99 HG2 PRO A 6 16.088 2.807 -18.455 1.00 77.47 H

ATOM 100 HG3 PRO A 6 15.054 3.851 -17.414 1.00 77.47 H

ATOM 101 HD2 PRO A 6 13.609 3.103 -19.234 1.00 77.47 H

ATOM 102 HD3 PRO A 6 14.198 1.447 -19.080 1.00 77.47 H

ATOM 103 N TYR A 7 13.093 3.590 -15.008 1.00 79.75 N

ATOM 104 CA TYR A 7 12.796 4.339 -13.781 1.00 79.75 C

ATOM 105 C TYR A 7 11.458 3.913 -13.160 1.00 79.75 C

ATOM 106 O TYR A 7 11.334 3.787 -11.943 1.00 79.75 O

ATOM 107 CB TYR A 7 12.833 5.844 -14.077 1.00 79.75 C

ATOM 108 CG TYR A 7 12.953 6.711 -12.837 1.00 79.75 C

ATOM 109 CD1 TYR A 7 11.939 7.619 -12.489 1.00 79.75 C

ATOM 110 CD2 TYR A 7 14.111 6.638 -12.039 1.00 79.75 C

ATOM 111 CE1 TYR A 7 12.032 8.371 -11.302 1.00 79.75 C

ATOM 112 CE2 TYR A 7 14.218 7.407 -10.864 1.00 79.75 C

ATOM 113 CZ TYR A 7 13.159 8.252 -10.476 1.00 79.75 C

ATOM 114 OH TYR A 7 13.219 8.978 -9.331 1.00 79.75 O

ATOM 115 H TYR A 7 13.008 4.062 -15.897 1.00 79.75 H

ATOM 116 HA TYR A 7 13.576 4.119 -13.052 1.00 79.75 H

ATOM 117 HB2 TYR A 7 11.932 6.111 -14.630 1.00 79.75 H

ATOM 118 HB3 TYR A 7 13.692 6.061 -14.712 1.00 79.75 H

ATOM 119 HD1 TYR A 7 11.088 7.754 -13.140 1.00 79.75 H

ATOM 120 HD2 TYR A 7 14.920 5.982 -12.322 1.00 79.75 H

ATOM 121 HE1 TYR A 7 11.275 9.088 -11.020 1.00 79.75 H

ATOM 122 HE2 TYR A 7 15.105 7.339 -10.251 1.00 79.75 H

ATOM 123 HH TYR A 7 14.056 8.877 -8.873 1.00 79.75 H

ATOM 124 N CYS A 8 10.474 3.592 -14.001 1.00 83.72 N

ATOM 125 CA CYS A 8 9.214 2.984 -13.584 1.00 83.72 C

ATOM 126 C CYS A 8 9.419 1.632 -12.896 1.00 83.72 C

ATOM 127 O CYS A 8 8.831 1.363 -11.845 1.00 83.72 O

ATOM 128 CB CYS A 8 8.379 2.756 -14.838 1.00 83.72 C

ATOM 129 SG CYS A 8 7.426 4.166 -15.394 1.00 83.72 S

ATOM 130 H CYS A 8 10.650 3.699 -14.990 1.00 83.72 H

ATOM 131 HA CYS A 8 8.683 3.646 -12.901 1.00 83.72 H

ATOM 132 HB2 CYS A 8 9.020 2.439 -15.660 1.00 83.72 H

ATOM 133 HB3 CYS A 8 7.687 1.933 -14.657 1.00 83.72 H

ATOM 134 N TRP A 9 10.249 0.777 -13.497 1.00 83.60 N

ATOM 135 CA TRP A 9 10.562 -0.539 -12.952 1.00 83.60 C

ATOM 136 C TRP A 9 11.330 -0.422 -11.633 1.00 83.60 C

ATOM 137 O TRP A 9 10.977 -1.082 -10.655 1.00 83.60 O

ATOM 138 CB TRP A 9 11.340 -1.335 -13.998 1.00 83.60 C

ATOM 139 CG TRP A 9 11.464 -2.786 -13.673 1.00 83.60 C

ATOM 140 CD1 TRP A 9 10.562 -3.738 -14.000 1.00 83.60 C

ATOM 141 CD2 TRP A 9 12.550 -3.479 -12.985 1.00 83.60 C

ATOM 142 NE1 TRP A 9 11.009 -4.970 -13.565 1.00 83.60 N

ATOM 143 CE2 TRP A 9 12.230 -4.869 -12.934 1.00 83.60 C

ATOM 144 CE3 TRP A 9 13.783 -3.079 -12.425 1.00 83.60 C

ATOM 145 CZ2 TRP A 9 13.089 -5.818 -12.362 1.00 83.60 C

ATOM 146 CZ3 TRP A 9 14.655 -4.025 -11.851 1.00 83.60 C

ATOM 147 CH2 TRP A 9 14.312 -5.389 -11.820 1.00 83.60 C

ATOM 148 H TRP A 9 10.685 1.050 -14.365 1.00 83.60 H

ATOM 149 HA TRP A 9 9.631 -1.067 -12.743 1.00 83.60 H

ATOM 150 HB2 TRP A 9 12.336 -0.910 -14.117 1.00 83.60 H

ATOM 151 HB3 TRP A 9 10.832 -1.248 -14.959 1.00 83.60 H

ATOM 152 HD1 TRP A 9 9.655 -3.560 -14.559 1.00 83.60 H

ATOM 153 HE1 TRP A 9 10.553 -5.841 -13.792 1.00 83.60 H

ATOM 154 HE3 TRP A 9 14.069 -2.038 -12.463 1.00 83.60 H

ATOM 155 HZ2 TRP A 9 12.830 -6.866 -12.366 1.00 83.60 H

ATOM 156 HZ3 TRP A 9 15.606 -3.704 -11.451 1.00 83.60 H

ATOM 157 HH2 TRP A 9 15.000 -6.107 -11.399 1.00 83.60 H

ATOM 158 N LEU A 10 12.300 0.495 -11.578 1.00 84.84 N

ATOM 159 CA LEU A 10 13.063 0.806 -10.375 1.00 84.84 C

ATOM 160 C LEU A 10 12.152 1.302 -9.249 1.00 84.84 C

ATOM 161 O LEU A 10 12.210 0.772 -8.143 1.00 84.84 O

ATOM 162 CB LEU A 10 14.135 1.854 -10.720 1.00 84.84 C

ATOM 163 CG LEU A 10 15.234 1.952 -9.650 1.00 84.84 C

ATOM 164 CD1 LEU A 10 16.253 0.824 -9.824 1.00 84.84 C

ATOM 165 CD2 LEU A 10 15.962 3.289 -9.766 1.00 84.84 C

ATOM 166 H LEU A 10 12.558 0.971 -12.430 1.00 84.84 H

ATOM 167 HA LEU A 10 13.547 -0.110 -10.035 1.00 84.84 H

ATOM 168 HB2 LEU A 10 14.609 1.599 -11.668 1.00 84.84 H

ATOM 169 HB3 LEU A 10 13.654 2.825 -10.841 1.00 84.84 H

ATOM 170 HG LEU A 10 14.794 1.892 -8.655 1.00 84.84 H

ATOM 171 HD11 LEU A 10 16.721 0.884 -10.807 1.00 84.84 H

ATOM 172 HD12 LEU A 10 15.769 -0.146 -9.714 1.00 84.84 H

ATOM 173 HD13 LEU A 10 17.027 0.911 -9.061 1.00 84.84 H

ATOM 174 HD21 LEU A 10 16.414 3.387 -10.753 1.00 84.84 H

ATOM 175 HD22 LEU A 10 15.261 4.108 -9.607 1.00 84.84 H

ATOM 176 HD23 LEU A 10 16.744 3.349 -9.009 1.00 84.84 H

ATOM 177 N CYS A 11 11.257 2.252 -9.536 1.00 85.65 N

ATOM 178 CA CYS A 11 10.270 2.731 -8.573 1.00 85.65 C

ATOM 179 C CYS A 11 9.410 1.571 -8.058 1.00 85.65 C

ATOM 180 O CYS A 11 9.242 1.406 -6.852 1.00 85.65 O

ATOM 181 CB CYS A 11 9.425 3.832 -9.225 1.00 85.65 C

ATOM 182 SG CYS A 11 8.079 4.458 -8.186 1.00 85.65 S

ATOM 183 H CYS A 11 11.285 2.678 -10.452 1.00 85.65 H

ATOM 184 HA CYS A 11 10.791 3.161 -7.718 1.00 85.65 H

ATOM 185 HB2 CYS A 11 10.087 4.665 -9.460 1.00 85.65 H

ATOM 186 HB3 CYS A 11 9.004 3.465 -10.162 1.00 85.65 H

ATOM 187 N ARG A 12 8.922 0.705 -8.954 1.00 83.49 N

ATOM 188 CA ARG A 12 8.077 -0.433 -8.575 1.00 83.49 C

ATOM 189 C ARG A 12 8.822 -1.413 -7.669 1.00 83.49 C

ATOM 190 O ARG A 12 8.251 -1.875 -6.684 1.00 83.49 O

ATOM 191 CB ARG A 12 7.538 -1.093 -9.850 1.00 83.49 C

ATOM 192 CG ARG A 12 6.272 -1.912 -9.572 1.00 83.49 C

ATOM 193 CD ARG A 12 5.634 -2.439 -10.863 1.00 83.49 C

ATOM 194 NE ARG A 12 5.149 -1.343 -11.739 1.00 83.49 N

ATOM 195 CZ ARG A 12 4.134 -1.381 -12.590 1.00 83.49 C

ATOM 196 NH1 ARG A 12 3.363 -2.424 -12.676 1.00 83.49 N

ATOM 197 NH2 ARG A 12 3.883 -0.400 -13.415 1.00 83.49 N

ATOM 198 H ARG A 12 9.110 0.877 -9.932 1.00 83.49 H

ATOM 199 HA ARG A 12 7.245 -0.041 -7.990 1.00 83.49 H

ATOM 200 HB2 ARG A 12 7.284 -0.305 -10.559 1.00 83.49 H

ATOM 201 HB3 ARG A 12 8.303 -1.725 -10.301 1.00 83.49 H

ATOM 202 HG2 ARG A 12 6.520 -2.754 -8.924 1.00 83.49 H

ATOM 203 HG3 ARG A 12 5.543 -1.280 -9.067 1.00 83.49 H

ATOM 204 HD2 ARG A 12 6.370 -3.035 -11.403 1.00 83.49 H

ATOM 205 HD3 ARG A 12 4.803 -3.085 -10.580 1.00 83.49 H

ATOM 206 HE ARG A 12 5.697 -0.495 -11.747 1.00 83.49 H

ATOM 207 HH11 ARG A 12 2.706 -2.420 -13.443 1.00 83.49 H

ATOM 208 HH12 ARG A 12 3.694 -3.290 -12.276 1.00 83.49 H

ATOM 209 HH21 ARG A 12 3.368 -0.560 -14.269 1.00 83.49 H

ATOM 210 HH22 ARG A 12 4.480 0.413 -13.458 1.00 83.49 H

ATOM 211 N ALA A 13 10.088 -1.699 -7.967 1.00 84.37 N

ATOM 212 CA ALA A 13 10.945 -2.543 -7.140 1.00 84.37 C

ATOM 213 C ALA A 13 11.224 -1.911 -5.766 1.00 84.37 C

ATOM 214 O ALA A 13 11.152 -2.598 -4.745 1.00 84.37 O

ATOM 215 CB ALA A 13 12.243 -2.814 -7.911 1.00 84.37 C

ATOM 216 H ALA A 13 10.486 -1.291 -8.801 1.00 84.37 H

ATOM 217 HA ALA A 13 10.443 -3.495 -6.967 1.00 84.37 H

ATOM 218 HB1 ALA A 13 12.017 -3.289 -8.866 1.00 84.37 H

ATOM 219 HB2 ALA A 13 12.887 -3.474 -7.331 1.00 84.37 H

ATOM 220 HB3 ALA A 13 12.773 -1.880 -8.101 1.00 84.37 H

ATOM 221 N LEU A 14 11.489 -0.603 -5.734 1.00 86.49 N

ATOM 222 CA LEU A 14 11.778 0.139 -4.511 1.00 86.49 C

ATOM 223 C LEU A 14 10.555 0.194 -3.592 1.00 86.49 C

ATOM 224 O LEU A 14 10.649 -0.182 -2.426 1.00 86.49 O

ATOM 225 CB LEU A 14 12.272 1.548 -4.885 1.00 86.49 C

ATOM 226 CG LEU A 14 13.130 2.188 -3.783 1.00 86.49 C

ATOM 227 CD1 LEU A 14 14.552 1.619 -3.809 1.00 86.49 C

ATOM 228 CD2 LEU A 14 13.214 3.697 -3.995 1.00 86.49 C

ATOM 229 H LEU A 14 11.560 -0.106 -6.610 1.00 86.49 H

ATOM 230 HA LEU A 14 12.567 -0.390 -3.977 1.00 86.49 H

ATOM 231 HB2 LEU A 14 12.876 1.501 -5.791 1.00 86.49 H

ATOM 232 HB3 LEU A 14 11.411 2.182 -5.099 1.00 86.49 H

ATOM 233 HG LEU A 14 12.680 2.000 -2.808 1.00 86.49 H

ATOM 234 HD11 LEU A 14 15.018 1.809 -4.776 1.00 86.49 H

ATOM 235 HD12 LEU A 14 15.148 2.098 -3.033 1.00 86.49 H

ATOM 236 HD13 LEU A 14 14.538 0.546 -3.618 1.00 86.49 H

ATOM 237 HD21 LEU A 14 13.834 4.145 -3.219 1.00 86.49 H

ATOM 238 HD22 LEU A 14 12.217 4.135 -3.935 1.00 86.49 H

ATOM 239 HD23 LEU A 14 13.647 3.918 -4.971 1.00 86.49 H

ATOM 240 N ILE A 15 9.387 0.568 -4.125 1.00 85.00 N

ATOM 241 CA ILE A 15 8.140 0.605 -3.355 1.00 85.00 C

ATOM 242 C ILE A 15 7.781 -0.792 -2.847 1.00 85.00 C

ATOM 243 O ILE A 15 7.412 -0.936 -1.685 1.00 85.00 O

ATOM 244 CB ILE A 15 6.986 1.235 -4.167 1.00 85.00 C

ATOM 245 CG1 ILE A 15 7.249 2.703 -4.573 1.00 85.00 C

ATOM 246 CG2 ILE A 15 5.683 1.168 -3.351 1.00 85.00 C

ATOM 247 CD1 ILE A 15 7.401 3.694 -3.413 1.00 85.00 C

ATOM 248 H ILE A 15 9.375 0.872 -5.088 1.00 85.00 H

ATOM 249 HA ILE A 15 8.305 1.219 -2.469 1.00 85.00 H

ATOM 250 HB ILE A 15 6.848 0.659 -5.082 1.00 85.00 H

ATOM 251 HG12 ILE A 15 8.160 2.768 -5.168 1.00 85.00 H

ATOM 252 HG13 ILE A 15 6.429 3.041 -5.206 1.00 85.00 H

ATOM 253 HG21 ILE A 15 5.865 1.474 -2.321 1.00 85.00 H

ATOM 254 HG22 ILE A 15 4.928 1.834 -3.770 1.00 85.00 H

ATOM 255 HG23 ILE A 15 5.298 0.148 -3.345 1.00 85.00 H

ATOM 256 HD11 ILE A 15 6.510 3.699 -2.784 1.00 85.00 H

ATOM 257 HD12 ILE A 15 8.273 3.441 -2.810 1.00 85.00 H

ATOM 258 HD13 ILE A 15 7.543 4.696 -3.819 1.00 85.00 H

ATOM 259 N LYS A 16 7.977 -1.837 -3.661 1.00 82.91 N

ATOM 260 CA LYS A 16 7.746 -3.221 -3.231 1.00 82.91 C

ATOM 261 C LYS A 16 8.665 -3.633 -2.076 1.00 82.91 C

ATOM 262 O LYS A 16 8.215 -4.315 -1.157 1.00 82.91 O

ATOM 263 CB LYS A 16 7.884 -4.143 -4.446 1.00 82.91 C

ATOM 264 CG LYS A 16 7.299 -5.533 -4.185 1.00 82.91 C

ATOM 265 CD LYS A 16 7.408 -6.390 -5.451 1.00 82.91 C

ATOM 266 CE LYS A 16 6.622 -7.688 -5.253 1.00 82.91 C

ATOM 267 NZ LYS A 16 6.790 -8.607 -6.406 1.00 82.91 N

ATOM 268 H LYS A 16 8.271 -1.660 -4.611 1.00 82.91 H

ATOM 269 HA LYS A 16 6.725 -3.288 -2.854 1.00 82.91 H

ATOM 270 HB2 LYS A 16 8.931 -4.223 -4.737 1.00 82.91 H

ATOM 271 HB3 LYS A 16 7.320 -3.707 -5.271 1.00 82.91 H

ATOM 272 HG2 LYS A 16 7.837 -6.020 -3.372 1.00 82.91 H

ATOM 273 HG3 LYS A 16 6.251 -5.429 -3.907 1.00 82.91 H

ATOM 274 HD2 LYS A 16 6.994 -5.842 -6.297 1.00 82.91 H

ATOM 275 HD3 LYS A 16 8.459 -6.608 -5.641 1.00 82.91 H

ATOM 276 HE2 LYS A 16 6.972 -8.167 -4.339 1.00 82.91 H

ATOM 277 HE3 LYS A 16 5.571 -7.437 -5.111 1.00 82.91 H

ATOM 278 HZ1 LYS A 16 6.278 -9.462 -6.245 1.00 82.91 H

ATOM 279 HZ2 LYS A 16 7.764 -8.846 -6.522 1.00 82.91 H

ATOM 280 HZ3 LYS A 16 6.456 -8.177 -7.257 1.00 82.91 H

ATOM 281 N ARG A 17 9.934 -3.203 -2.080 1.00 84.15 N

ATOM 282 CA ARG A 17 10.854 -3.417 -0.948 1.00 84.15 C

ATOM 283 C ARG A 17 10.421 -2.657 0.299 1.00 84.15 C

ATOM 284 O ARG A 17 10.409 -3.242 1.375 1.00 84.15 O

ATOM 285 CB ARG A 17 12.291 -3.016 -1.306 1.00 84.15 C

ATOM 286 CG ARG A 17 13.052 -4.102 -2.080 1.00 84.15 C

ATOM 287 CD ARG A 17 14.459 -4.236 -1.483 1.00 84.15 C

ATOM 288 NE ARG A 17 15.315 -5.158 -2.249 1.00 84.15 N

ATOM 289 CZ ARG A 17 16.502 -5.603 -1.866 1.00 84.15 C

ATOM 290 NH1 ARG A 17 17.012 -5.312 -0.701 1.00 84.15 N

ATOM 291 NH2 ARG A 17 17.211 -6.354 -2.660 1.00 84.15 N

ATOM 292 H ARG A 17 10.246 -2.656 -2.870 1.00 84.15 H

ATOM 293 HA ARG A 17 10.832 -4.467 -0.657 1.00 84.15 H

ATOM 294 HB2 ARG A 17 12.300 -2.088 -1.879 1.00 84.15 H

ATOM 295 HB3 ARG A 17 12.815 -2.816 -0.372 1.00 84.15 H

ATOM 296 HG2 ARG A 17 12.544 -5.063 -1.998 1.00 84.15 H

ATOM 297 HG3 ARG A 17 13.114 -3.821 -3.131 1.00 84.15 H

ATOM 298 HD2 ARG A 17 14.928 -3.252 -1.468 1.00 84.15 H

ATOM 299 HD3 ARG A 17 14.363 -4.591 -0.457 1.00 84.15 H

ATOM 300 HE ARG A 17 14.995 -5.431 -3.167 1.00 84.15 H

ATOM 301 HH11 ARG A 17 16.493 -4.724 -0.064 1.00 84.15 H

ATOM 302 HH12 ARG A 17 17.921 -5.660 -0.431 1.00 84.15 H

ATOM 303 HH21 ARG A 17 16.877 -6.600 -3.580 1.00 84.15 H

ATOM 304 HH22 ARG A 17 18.119 -6.682 -2.362 1.00 84.15 H

ATOM 305 N ILE A 18 10.047 -1.388 0.154 1.00 83.62 N

ATOM 306 CA ILE A 18 9.557 -0.571 1.271 1.00 83.62 C

ATOM 307 C ILE A 18 8.316 -1.229 1.881 1.00 83.62 C

ATOM 308 O ILE A 18 8.235 -1.385 3.091 1.00 83.62 O

ATOM 309 CB ILE A 18 9.295 0.878 0.799 1.00 83.62 C

ATOM 310 CG1 ILE A 18 10.628 1.564 0.412 1.00 83.62 C

ATOM 311 CG2 ILE A 18 8.591 1.696 1.898 1.00 83.62 C

ATOM 312 CD1 ILE A 18 10.448 2.831 -0.433 1.00 83.62 C

ATOM 313 H ILE A 18 10.119 -0.966 -0.761 1.00 83.62 H

ATOM 314 HA ILE A 18 10.316 -0.543 2.052 1.00 83.62 H

ATOM 315 HB ILE A 18 8.646 0.847 -0.075 1.00 83.62 H

ATOM 316 HG12 ILE A 18 11.253 0.880 -0.162 1.00 83.62 H

ATOM 317 HG13 ILE A 18 11.180 1.818 1.318 1.00 83.62 H

ATOM 318 HG21 ILE A 18 9.188 1.693 2.810 1.00 83.62 H

ATOM 319 HG22 ILE A 18 8.443 2.727 1.576 1.00 83.62 H

ATOM 320 HG23 ILE A 18 7.608 1.281 2.118 1.00 83.62 H

ATOM 321 HD11 ILE A 18 9.909 2.589 -1.348 1.00 83.62 H

ATOM 322 HD12 ILE A 18 9.907 3.596 0.123 1.00 83.62 H

ATOM 323 HD13 ILE A 18 11.429 3.225 -0.699 1.00 83.62 H

ATOM 324 N GLN A 19 7.392 -1.720 1.057 1.00 77.51 N

ATOM 325 CA GLN A 19 6.187 -2.416 1.515 1.00 77.51 C

ATOM 326 C GLN A 19 6.456 -3.741 2.207 1.00 77.51 C

ATOM 327 O GLN A 19 5.762 -4.060 3.165 1.00 77.51 O

ATOM 328 CB GLN A 19 5.293 -2.691 0.319 1.00 77.51 C

ATOM 329 CG GLN A 19 4.639 -1.394 -0.125 1.00 77.51 C

ATOM 330 CD GLN A 19 4.043 -1.529 -1.500 1.00 77.51 C

ATOM 331 OE1 GLN A 19 4.364 -2.381 -2.312 1.00 77.51 O

ATOM 332 NE2 GLN A 19 3.177 -0.616 -1.827 1.00 77.51 N

ATOM 333 H GLN A 19 7.497 -1.552 0.067 1.00 77.51 H

ATOM 334 HA GLN A 19 5.654 -1.789 2.231 1.00 77.51 H

ATOM 335 HB2 GLN A 19 4.510 -3.400 0.588 1.00 77.51 H

ATOM 336 HB3 GLN A 19 5.896 -3.122 -0.481 1.00 77.51 H

ATOM 337 HG2 GLN A 19 3.855 -1.134 0.587 1.00 77.51 H

ATOM 338 HG3 GLN A 19 5.357 -0.574 -0.151 1.00 77.51 H

ATOM 339 HE21 GLN A 19 2.700 -0.783 -2.702 1.00 77.51 H

ATOM 340 HE22 GLN A 19 2.913 0.098 -1.164 1.00 77.51 H

ATOM 341 N ALA A 20 7.451 -4.503 1.750 1.00 78.04 N

ATOM 342 CA ALA A 20 7.888 -5.701 2.459 1.00 78.04 C

ATOM 343 C ALA A 20 8.452 -5.368 3.851 1.00 78.04 C

ATOM 344 O ALA A 20 8.377 -6.195 4.755 1.00 78.04 O

ATOM 345 CB ALA A 20 8.915 -6.431 1.588 1.00 78.04 C

ATOM 346 H ALA A 20 7.947 -4.222 0.916 1.00 78.04 H

ATOM 347 HA ALA A 20 7.029 -6.356 2.609 1.00 78.04 H

ATOM 348 HB1 ALA A 20 9.228 -7.347 2.088 1.00 78.04 H

ATOM 349 HB2 ALA A 20 9.790 -5.800 1.430 1.00 78.04 H

ATOM 350 HB3 ALA A 20 8.471 -6.683 0.625 1.00 78.04 H

ATOM 351 N MET A 21 8.986 -4.155 4.021 1.00 79.63 N

ATOM 352 CA MET A 21 9.491 -3.642 5.293 1.00 79.63 C

ATOM 353 C MET A 21 8.384 -3.056 6.184 1.00 79.63 C

ATOM 354 O MET A 21 8.561 -2.986 7.397 1.00 79.63 O

ATOM 355 CB MET A 21 10.585 -2.608 4.986 1.00 79.63 C

ATOM 356 CG MET A 21 11.702 -2.616 6.029 1.00 79.63 C

ATOM 357 SD MET A 21 13.044 -1.441 5.687 1.00 79.63 S

ATOM 358 CE MET A 21 13.759 -2.130 4.170 1.00 79.63 C

ATOM 359 H MET A 21 9.042 -3.541 3.221 1.00 79.63 H

ATOM 360 HA MET A 21 9.938 -4.472 5.840 1.00 79.63 H

ATOM 361 HB2 MET A 21 10.151 -1.610 4.927 1.00 79.63 H

ATOM 362 HB3 MET A 21 11.036 -2.840 4.021 1.00 79.63 H

ATOM 363 HG2 MET A 21 12.126 -3.619 6.092 1.00 79.63 H

ATOM 364 HG3 MET A 21 11.271 -2.369 7.000 1.00 79.63 H

ATOM 365 HE1 MET A 21 13.044 -2.048 3.352 1.00 79.63 H

ATOM 366 HE2 MET A 21 14.018 -3.176 4.333 1.00 79.63 H

ATOM 367 HE3 MET A 21 14.660 -1.571 3.916 1.00 79.63 H

ATOM 368 N ILE A 22 7.236 -2.657 5.618 1.00 75.86 N

ATOM 369 CA ILE A 22 6.087 -2.179 6.397 1.00 75.86 C

ATOM 370 C ILE A 22 5.437 -3.387 7.091 1.00 75.86 C

ATOM 371 O ILE A 22 4.917 -4.284 6.418 1.00 75.86 O

ATOM 372 CB ILE A 22 5.072 -1.388 5.539 1.00 75.86 C

ATOM 373 CG1 ILE A 22 5.712 -0.086 5.005 1.00 75.86 C

ATOM 374 CG2 ILE A 22 3.817 -1.024 6.363 1.00 75.86 C

ATOM 375 CD1 ILE A 22 4.901 0.610 3.904 1.00 75.86 C

ATOM 376 H ILE A 22 7.140 -2.748 4.617 1.00 75.86 H

ATOM 377 HA ILE A 22 6.475 -1.486 7.142 1.00 75.86 H

ATOM 378 HB ILE A 22 4.768 -2.010 4.697 1.00 75.86 H

ATOM 379 HG12 ILE A 22 6.699 -0.302 4.596 1.00 75.86 H

ATOM 380 HG13 ILE A 22 5.859 0.611 5.829 1.00 75.86 H

ATOM 381 HG21 ILE A 22 3.103 -0.479 5.746 1.00 75.86 H

ATOM 382 HG22 ILE A 22 4.093 -0.407 7.218 1.00 75.86 H

ATOM 383 HG23 ILE A 22 3.314 -1.924 6.717 1.00 75.86 H

ATOM 384 HD11 ILE A 22 5.495 1.420 3.480 1.00 75.86 H

ATOM 385 HD12 ILE A 22 4.644 -0.095 3.113 1.00 75.86 H

ATOM 386 HD13 ILE A 22 3.987 1.035 4.318 1.00 75.86 H

ATOM 387 N PRO A 23 5.406 -3.427 8.435 1.00 71.19 N

ATOM 388 CA PRO A 23 4.795 -4.529 9.156 1.00 71.19 C

ATOM 389 C PRO A 23 3.287 -4.535 8.894 1.00 71.19 C

ATOM 390 O PRO A 23 2.544 -3.704 9.418 1.00 71.19 O

ATOM 391 CB PRO A 23 5.157 -4.306 10.629 1.00 71.19 C

ATOM 392 CG PRO A 23 5.366 -2.796 10.729 1.00 71.19 C

ATOM 393 CD PRO A 23 5.930 -2.431 9.361 1.00 71.19 C

ATOM 394 HA PRO A 23 5.227 -5.473 8.822 1.00 71.19 H

ATOM 395 HB2 PRO A 23 6.097 -4.812 10.849 1.00 71.19 H

ATOM 396 HB3 PRO A 23 4.377 -4.652 11.307 1.00 71.19 H

ATOM 397 HG2 PRO A 23 6.054 -2.533 11.532 1.00 71.19 H

ATOM 398 HG3 PRO A 23 4.407 -2.297 10.868 1.00 71.19 H

ATOM 399 HD2 PRO A 23 5.612 -1.423 9.093 1.00 71.19 H

ATOM 400 HD3 PRO A 23 7.018 -2.492 9.386 1.00 71.19 H

ATOM 401 N LYS A 24 2.808 -5.520 8.123 1.00 69.35 N

ATOM 402 CA LYS A 24 1.371 -5.739 7.855 1.00 69.35 C

ATOM 403 C LYS A 24 0.541 -5.798 9.148 1.00 69.35 C

ATOM 404 O LYS A 24 -0.630 -5.428 9.148 1.00 69.35 O

ATOM 405 CB LYS A 24 1.181 -7.032 7.037 1.00 69.35 C

ATOM 406 CG LYS A 24 1.817 -6.983 5.635 1.00 69.35 C

ATOM 407 CD LYS A 24 1.617 -8.312 4.886 1.00 69.35 C

ATOM 408 CE LYS A 24 2.248 -8.260 3.487 1.00 69.35 C

ATOM 409 NZ LYS A 24 2.130 -9.562 2.782 1.00 69.35 N

ATOM 410 H LYS A 24 3.490 -6.054 7.603 1.00 69.35 H

ATOM 411 HA LYS A 24 0.987 -4.895 7.282 1.00 69.35 H

ATOM 412 HB2 LYS A 24 0.112 -7.211 6.919 1.00 69.35 H

ATOM 413 HB3 LYS A 24 1.605 -7.869 7.592 1.00 69.35 H

ATOM 414 HG2 LYS A 24 2.885 -6.787 5.723 1.00 69.35 H

ATOM 415 HG3 LYS A 24 1.359 -6.175 5.063 1.00 69.35 H

ATOM 416 HD2 LYS A 24 0.551 -8.516 4.786 1.00 69.35 H

ATOM 417 HD3 LYS A 24 2.077 -9.117 5.459 1.00 69.35 H

ATOM 418 HE2 LYS A 24 1.731 -7.491 2.912 1.00 69.35 H

ATOM 419 HE3 LYS A 24 3.295 -7.970 3.575 1.00 69.35 H

ATOM 420 HZ1 LYS A 24 2.339 -9.495 1.796 1.00 69.35 H

ATOM 421 HZ2 LYS A 24 1.168 -9.869 2.791 1.00 69.35 H

ATOM 422 HZ3 LYS A 24 2.669 -10.299 3.213 1.00 69.35 H

ATOM 423 N GLY A 25 1.156 -6.224 10.257 1.00 67.23 N

ATOM 424 CA GLY A 25 0.541 -6.257 11.585 1.00 67.23 C

ATOM 425 C GLY A 25 0.144 -4.880 12.130 1.00 67.23 C

ATOM 426 O GLY A 25 -0.907 -4.770 12.752 1.00 67.23 O

ATOM 427 H GLY A 25 2.125 -6.493 10.173 1.00 67.23 H

ATOM 428 HA2 GLY A 25 -0.351 -6.882 11.557 1.00 67.23 H

ATOM 429 HA3 GLY A 25 1.246 -6.700 12.288 1.00 67.23 H

ATOM 430 N ALA A 26 0.909 -3.819 11.845 1.00 69.69 N

ATOM 431 CA ALA A 26 0.611 -2.478 12.356 1.00 69.69 C

ATOM 432 C ALA A 26 -0.705 -1.924 11.788 1.00 69.69 C

ATOM 433 O ALA A 26 -1.501 -1.335 12.517 1.00 69.69 O

ATOM 434 CB ALA A 26 1.791 -1.552 12.041 1.00 69.69 C

ATOM 435 H ALA A 26 1.706 -3.923 11.234 1.00 69.69 H

ATOM 436 HA ALA A 26 0.498 -2.529 13.439 1.00 69.69 H

ATOM 437 HB1 ALA A 26 1.586 -0.555 12.431 1.00 69.69 H

ATOM 438 HB2 ALA A 26 1.946 -1.487 10.964 1.00 69.69 H

ATOM 439 HB3 ALA A 26 2.694 -1.933 12.518 1.00 69.69 H

ATOM 440 N LEU A 27 -0.989 -2.178 10.506 1.00 67.77 N

ATOM 441 CA LEU A 27 -2.268 -1.765 9.930 1.00 67.77 C

ATOM 442 C LEU A 27 -3.442 -2.571 10.490 1.00 67.77 C

ATOM 443 O LEU A 27 -4.510 -1.994 10.661 1.00 67.77 O

ATOM 444 CB LEU A 27 -2.258 -1.870 8.404 1.00 67.77 C

ATOM 445 CG LEU A 27 -2.355 -0.524 7.660 1.00 67.77 C

ATOM 446 CD1 LEU A 27 -0.999 -0.110 7.103 1.00 67.77 C

ATOM 447 CD2 LEU A 27 -3.346 -0.550 6.496 1.00 67.77 C

ATOM 448 H LEU A 27 -0.315 -2.671 9.938 1.00 67.77 H

ATOM 449 HA LEU A 27 -2.446 -0.729 10.217 1.00 67.77 H

ATOM 450 HB2 LEU A 27 -3.155 -2.428 8.133 1.00 67.77 H

ATOM 451 HB3 LEU A 27 -1.378 -2.429 8.086 1.00 67.77 H

ATOM 452 HG LEU A 27 -2.695 0.249 8.349 1.00 67.77 H

ATOM 453 HD11 LEU A 27 -0.278 -0.041 7.918 1.00 67.77 H

ATOM 454 HD12 LEU A 27 -1.081 0.869 6.630 1.00 67.77 H

ATOM 455 HD13 LEU A 27 -0.649 -0.838 6.371 1.00 67.77 H

ATOM 456 HD21 LEU A 27 -4.329 -0.839 6.869 1.00 67.77 H

ATOM 457 HD22 LEU A 27 -3.438 0.457 6.090 1.00 67.77 H

ATOM 458 HD23 LEU A 27 -3.013 -1.204 5.690 1.00 67.77 H

ATOM 459 N ARG A 28 -3.272 -3.865 10.810 1.00 66.27 N

ATOM 460 CA ARG A 28 -4.346 -4.633 11.472 1.00 66.27 C

ATOM 461 C ARG A 28 -4.761 -3.964 12.779 1.00 66.27 C

ATOM 462 O ARG A 28 -5.952 -3.815 13.031 1.00 66.27 O

ATOM 463 CB ARG A 28 -3.949 -6.081 11.783 1.00 66.27 C

ATOM 464 CG ARG A 28 -3.633 -6.945 10.560 1.00 66.27 C

ATOM 465 CD ARG A 28 -3.562 -8.403 11.029 1.00 66.27 C

ATOM 466 NE ARG A 28 -2.935 -9.285 10.033 1.00 66.27 N

ATOM 467 CZ ARG A 28 -3.102 -10.597 9.963 1.00 66.27 C

ATOM 468 NH1 ARG A 28 -3.946 -11.248 10.721 1.00 66.27 N

ATOM 469 NH2 ARG A 28 -2.430 -11.287 9.080 1.00 66.27 N

ATOM 470 H ARG A 28 -2.370 -4.291 10.656 1.00 66.27 H

ATOM 471 HA ARG A 28 -5.232 -4.639 10.837 1.00 66.27 H

ATOM 472 HB2 ARG A 28 -3.093 -6.093 12.457 1.00 66.27 H

ATOM 473 HB3 ARG A 28 -4.787 -6.537 12.310 1.00 66.27 H

ATOM 474 HG2 ARG A 28 -2.674 -6.639 10.141 1.00 66.27 H

ATOM 475 HG3 ARG A 28 -4.412 -6.840 9.805 1.00 66.27 H

ATOM 476 HD2 ARG A 28 -4.576 -8.736 11.247 1.00 66.27 H

ATOM 477 HD3 ARG A 28 -2.981 -8.456 11.950 1.00 66.27 H

ATOM 478 HE ARG A 28 -2.327 -8.850 9.353 1.00 66.27 H

ATOM 479 HH11 ARG A 28 -4.106 -12.242 10.643 1.00 66.27 H

ATOM 480 HH12 ARG A 28 -4.570 -10.766 11.352 1.00 66.27 H

ATOM 481 HH21 ARG A 28 -1.788 -10.835 8.444 1.00 66.27 H

ATOM 482 HH22 ARG A 28 -2.590 -12.283 9.029 1.00 66.27 H

ATOM 483 N VAL A 29 -3.782 -3.514 13.568 1.00 68.84 N

ATOM 484 CA VAL A 29 -4.029 -2.788 14.822 1.00 68.84 C

ATOM 485 C VAL A 29 -4.771 -1.477 14.555 1.00 68.84 C

ATOM 486 O VAL A 29 -5.745 -1.187 15.245 1.00 68.84 O

ATOM 487 CB VAL A 29 -2.717 -2.544 15.594 1.00 68.84 C

ATOM 488 CG1 VAL A 29 -2.953 -1.750 16.885 1.00 68.84 C

ATOM 489 CG2 VAL A 29 -2.058 -3.873 15.988 1.00 68.84 C

ATOM 490 H VAL A 29 -2.827 -3.683 13.286 1.00 68.84 H

ATOM 491 HA VAL A 29 -4.680 -3.396 15.451 1.00 68.84 H

ATOM 492 HB VAL A 29 -2.022 -1.984 14.969 1.00 68.84 H

ATOM 493 HG11 VAL A 29 -2.019 -1.659 17.439 1.00 68.84 H

ATOM 494 HG12 VAL A 29 -3.302 -0.744 16.654 1.00 68.84 H

ATOM 495 HG13 VAL A 29 -3.692 -2.254 17.508 1.00 68.84 H

ATOM 496 HG21 VAL A 29 -2.723 -4.444 16.635 1.00 68.84 H

ATOM 497 HG22 VAL A 29 -1.125 -3.679 16.517 1.00 68.84 H

ATOM 498 HG23 VAL A 29 -1.829 -4.468 15.103 1.00 68.84 H

ATOM 499 N ALA A 30 -4.381 -0.725 13.522 1.00 69.78 N

ATOM 500 CA ALA A 30 -5.063 0.514 13.149 1.00 69.78 C

ATOM 501 C ALA A 30 -6.527 0.282 12.719 1.00 69.78 C

ATOM 502 O ALA A 30 -7.423 0.975 13.195 1.00 69.78 O

ATOM 503 CB ALA A 30 -4.249 1.216 12.056 1.00 69.78 C

ATOM 504 H ALA A 30 -3.554 -0.993 13.008 1.00 69.78 H

ATOM 505 HA ALA A 30 -5.087 1.168 14.021 1.00 69.78 H

ATOM 506 HB1 ALA A 30 -3.228 1.378 12.402 1.00 69.78 H

ATOM 507 HB2 ALA A 30 -4.235 0.615 11.146 1.00 69.78 H

ATOM 508 HB3 ALA A 30 -4.701 2.182 11.834 1.00 69.78 H

ATOM 509 N VAL A 31 -6.810 -0.723 11.877 1.00 68.34 N

ATOM 510 CA VAL A 31 -8.197 -1.038 11.473 1.00 68.34 C

ATOM 511 C VAL A 31 -9.016 -1.532 12.671 1.00 68.34 C

ATOM 512 O VAL A 31 -10.167 -1.130 12.843 1.00 68.34 O

ATOM 513 CB VAL A 31 -8.257 -2.054 10.312 1.00 68.34 C

ATOM 514 CG1 VAL A 31 -9.700 -2.262 9.831 1.00 68.34 C

ATOM 515 CG2 VAL A 31 -7.479 -1.578 9.076 1.00 68.34 C

ATOM 516 H VAL A 31 -6.048 -1.269 11.503 1.00 68.34 H

ATOM 517 HA VAL A 31 -8.668 -0.116 11.130 1.00 68.34 H

ATOM 518 HB VAL A 31 -7.851 -3.010 10.641 1.00 68.34 H

ATOM 519 HG11 VAL A 31 -10.106 -1.325 9.450 1.00 68.34 H

ATOM 520 HG12 VAL A 31 -9.730 -3.012 9.041 1.00 68.34 H

ATOM 521 HG13 VAL A 31 -10.338 -2.602 10.647 1.00 68.34 H

ATOM 522 HG21 VAL A 31 -6.414 -1.497 9.295 1.00 68.34 H

ATOM 523 HG22 VAL A 31 -7.845 -0.601 8.760 1.00 68.34 H

ATOM 524 HG23 VAL A 31 -7.606 -2.290 8.261 1.00 68.34 H

ATOM 525 N ALA A 32 -8.417 -2.340 13.550 1.00 69.42 N

ATOM 526 CA ALA A 32 -9.066 -2.781 14.782 1.00 69.42 C

ATOM 527 C ALA A 32 -9.416 -1.605 15.714 1.00 69.42 C

ATOM 528 O ALA A 32 -10.463 -1.641 16.365 1.00 69.42 O

ATOM 529 CB ALA A 32 -8.159 -3.802 15.480 1.00 69.42 C

ATOM 530 H ALA A 32 -7.480 -2.665 13.362 1.00 69.42 H

ATOM 531 HA ALA A 32 -10.000 -3.278 14.519 1.00 69.42 H

ATOM 532 HB1 ALA A 32 -7.967 -4.641 14.812 1.00 69.42 H

ATOM 533 HB2 ALA A 32 -8.651 -4.174 16.379 1.00 69.42 H

ATOM 534 HB3 ALA A 32 -7.212 -3.339 15.757 1.00 69.42 H

ATOM 535 N GLN A 33 -8.586 -0.555 15.757 1.00 68.48 N

ATOM 536 CA GLN A 33 -8.885 0.680 16.489 1.00 68.48 C

ATOM 537 C GLN A 33 -10.059 1.449 15.878 1.00 68.48 C

ATOM 538 O GLN A 33 -10.911 1.916 16.628 1.00 68.48 O

ATOM 539 CB GLN A 33 -7.654 1.593 16.558 1.00 68.48 C

ATOM 540 CG GLN A 33 -6.624 1.130 17.594 1.00 68.48 C

ATOM 541 CD GLN A 33 -5.446 2.096 17.691 1.00 68.48 C

ATOM 542 OE1 GLN A 33 -5.115 2.832 16.779 1.00 68.48 O

ATOM 543 NE2 GLN A 33 -4.758 2.139 18.810 1.00 68.48 N

ATOM 544 H GLN A 33 -7.721 -0.602 15.236 1.00 68.48 H

ATOM 545 HA GLN A 33 -9.180 0.423 17.506 1.00 68.48 H

ATOM 546 HB2 GLN A 33 -7.182 1.662 15.578 1.00 68.48 H

ATOM 547 HB3 GLN A 33 -7.980 2.593 16.842 1.00 68.48 H

ATOM 548 HG2 GLN A 33 -7.109 1.072 18.569 1.00 68.48 H

ATOM 549 HG3 GLN A 33 -6.246 0.140 17.342 1.00 68.48 H

ATOM 550 HE21 GLN A 33 -4.978 1.542 19.594 1.00 68.48 H

ATOM 551 HE22 GLN A 33 -4.008 2.815 18.822 1.00 68.48 H

ATOM 552 N VAL A 34 -10.165 1.532 14.548 1.00 68.13 N

ATOM 553 CA VAL A 34 -11.318 2.178 13.893 1.00 68.13 C

ATOM 554 C VAL A 34 -12.625 1.479 14.278 1.00 68.13 C

ATOM 555 O VAL A 34 -13.590 2.152 14.633 1.00 68.13 O

ATOM 556 CB VAL A 34 -11.137 2.244 12.363 1.00 68.13 C

ATOM 557 CG1 VAL A 34 -12.374 2.818 11.659 1.00 68.13 C

ATOM 558 CG2 VAL A 34 -9.948 3.141 11.997 1.00 68.13 C

ATOM 559 H VAL A 34 -9.420 1.160 13.977 1.00 68.13 H

ATOM 560 HA VAL A 34 -11.393 3.202 14.261 1.00 68.13 H

ATOM 561 HB VAL A 34 -10.953 1.243 11.974 1.00 68.13 H

ATOM 562 HG11 VAL A 34 -12.624 3.794 12.075 1.00 68.13 H

ATOM 563 HG12 VAL A 34 -12.178 2.923 10.592 1.00 68.13 H

ATOM 564 HG13 VAL A 34 -13.226 2.149 11.779 1.00 68.13 H

ATOM 565 HG21 VAL A 34 -10.122 4.157 12.352 1.00 68.13 H

ATOM 566 HG22 VAL A 34 -9.030 2.765 12.448 1.00 68.13 H

ATOM 567 HG23 VAL A 34 -9.815 3.158 10.915 1.00 68.13 H

ATOM 568 N CYS A 35 -12.649 0.143 14.334 1.00 66.75 N

ATOM 569 CA CYS A 35 -13.833 -0.591 14.800 1.00 66.75 C

ATOM 570 C CYS A 35 -14.179 -0.350 16.282 1.00 66.75 C

ATOM 571 O CYS A 35 -15.317 -0.602 16.660 1.00 66.75 O

ATOM 572 CB CYS A 35 -13.673 -2.092 14.541 1.00 66.75 C

ATOM 573 SG CYS A 35 -13.771 -2.604 12.806 1.00 66.75 S

ATOM 574 H CYS A 35 -11.847 -0.373 14.001 1.00 66.75 H

ATOM 575 HA CYS A 35 -14.694 -0.245 14.228 1.00 66.75 H

ATOM 576 HB2 CYS A 35 -12.716 -2.418 14.946 1.00 66.75 H

ATOM 577 HB3 CYS A 35 -14.461 -2.620 15.078 1.00 66.75 H

ATOM 578 N ARG A 36 -13.252 0.145 17.122 1.00 65.08 N

ATOM 579 CA ARG A 36 -13.576 0.569 18.501 1.00 65.08 C

ATOM 580 C ARG A 36 -14.305 1.910 18.556 1.00 65.08 C

ATOM 581 O ARG A 36 -15.006 2.160 19.528 1.00 65.08 O

ATOM 582 CB ARG A 36 -12.325 0.659 19.388 1.00 65.08 C

ATOM 583 CG ARG A 36 -11.742 -0.701 19.774 1.00 65.08 C

ATOM 584 CD ARG A 36 -10.561 -0.472 20.723 1.00 65.08 C

ATOM 585 NE ARG A 36 -9.969 -1.744 21.173 1.00 65.08 N

ATOM 586 CZ ARG A 36 -8.838 -1.884 21.843 1.00 65.08 C

ATOM 587 NH1 ARG A 36 -8.103 -0.858 22.178 1.00 65.08 N

ATOM 588 NH2 ARG A 36 -8.424 -3.068 22.196 1.00 65.08 N

ATOM 589 H ARG A 36 -12.338 0.367 16.752 1.00 65.08 H

ATOM 590 HA ARG A 36 -14.273 -0.143 18.941 1.00 65.08 H

ATOM 591 HB2 ARG A 36 -12.603 1.168 20.311 1.00 65.08 H

ATOM 592 HB3 ARG A 36 -11.561 1.267 18.903 1.00 65.08 H

ATOM 593 HG2 ARG A 36 -12.507 -1.294 20.275 1.00 65.08 H

ATOM 594 HG3 ARG A 36 -11.404 -1.225 18.880 1.00 65.08 H

ATOM 595 HD2 ARG A 36 -10.911 0.088 21.590 1.00 65.08 H

ATOM 596 HD3 ARG A 36 -9.810 0.120 20.200 1.00 65.08 H

ATOM 597 HE ARG A 36 -10.492 -2.583 20.966 1.00 65.08 H

ATOM 598 HH11 ARG A 36 -8.450 0.067 21.970 1.00 65.08 H

ATOM 599 HH12 ARG A 36 -7.276 -0.980 22.745 1.00 65.08 H

ATOM 600 HH21 ARG A 36 -7.573 -3.174 22.729 1.00 65.08 H

ATOM 601 HH22 ARG A 36 -8.981 -3.884 21.985 1.00 65.08 H

ATOM 602 N VAL A 37 -14.123 2.768 17.551 1.00 68.72 N

ATOM 603 CA VAL A 37 -14.820 4.063 17.457 1.00 68.72 C

ATOM 604 C VAL A 37 -16.246 3.873 16.925 1.00 68.72 C

ATOM 605 O VAL A 37 -17.140 4.650 17.248 1.00 68.72 O

ATOM 606 CB VAL A 37 -14.019 5.055 16.587 1.00 68.72 C

ATOM 607 CG1 VAL A 37 -14.643 6.457 16.589 1.00 68.72 C

ATOM 608 CG2 VAL A 37 -12.572 5.207 17.082 1.00 68.72 C

ATOM 609 H VAL A 37 -13.517 2.495 16.791 1.00 68.72 H

ATOM 610 HA VAL A 37 -14.910 4.489 18.456 1.00 68.72 H

ATOM 611 HB VAL A 37 -13.993 4.691 15.560 1.00 68.72 H

ATOM 612 HG11 VAL A 37 -14.735 6.826 17.610 1.00 68.72 H

ATOM 613 HG12 VAL A 37 -14.023 7.143 16.012 1.00 68.72 H

ATOM 614 HG13 VAL A 37 -15.631 6.433 16.129 1.00 68.72 H

ATOM 615 HG21 VAL A 37 -12.039 4.260 17.000 1.00 68.72 H

ATOM 616 HG22 VAL A 37 -12.047 5.941 16.472 1.00 68.72 H

ATOM 617 HG23 VAL A 37 -12.567 5.534 18.122 1.00 68.72 H

ATOM 618 N VAL A 38 -16.482 2.823 16.133 1.00 63.10 N

ATOM 619 CA VAL A 38 -17.809 2.502 15.594 1.00 63.10 C

ATOM 620 C VAL A 38 -18.702 1.904 16.703 1.00 63.10 C

ATOM 621 O VAL A 38 -18.282 0.965 17.381 1.00 63.10 O

ATOM 622 CB VAL A 38 -17.694 1.573 14.371 1.00 63.10 C

ATOM 623 CG1 VAL A 38 -19.064 1.229 13.773 1.00 63.10 C

ATOM 624 CG2 VAL A 38 -16.879 2.234 13.248 1.00 63.10 C

ATOM 625 H VAL A 38 -15.714 2.202 15.923 1.00 63.10 H

ATOM 626 HA VAL A 38 -18.244 3.438 15.246 1.00 63.10 H

ATOM 627 HB VAL A 38 -17.199 0.648 14.665 1.00 63.10 H

ATOM 628 HG11 VAL A 38 -19.684 0.730 14.518 1.00 63.10 H

ATOM 629 HG12 VAL A 38 -18.934 0.550 12.930 1.00 63.10 H

ATOM 630 HG13 VAL A 38 -19.566 2.132 13.427 1.00 63.10 H

ATOM 631 HG21 VAL A 38 -17.354 3.164 12.937 1.00 63.10 H

ATOM 632 HG22 VAL A 38 -15.866 2.454 13.588 1.00 63.10 H

ATOM 633 HG23 VAL A 38 -16.811 1.561 12.394 1.00 63.10 H

ATOM 634 N PRO A 39 -19.935 2.411 16.913 1.00 52.18 N

ATOM 635 CA PRO A 39 -20.779 2.020 18.042 1.00 52.18 C

ATOM 636 C PRO A 39 -21.164 0.524 18.067 1.00 52.18 C

ATOM 637 O PRO A 39 -21.735 -0.027 17.124 1.00 52.18 O

ATOM 638 CB PRO A 39 -21.988 2.966 18.026 1.00 52.18 C

ATOM 639 CG PRO A 39 -21.999 3.548 16.614 1.00 52.18 C

ATOM 640 CD PRO A 39 -20.531 3.533 16.208 1.00 52.18 C

ATOM 641 HA PRO A 39 -20.217 2.235 18.951 1.00 52.18 H

ATOM 642 HB2 PRO A 39 -22.920 2.449 18.251 1.00 52.18 H

ATOM 643 HB3 PRO A 39 -21.823 3.770 18.744 1.00 52.18 H

ATOM 644 HG2 PRO A 39 -22.415 4.555 16.592 1.00 52.18 H

ATOM 645 HG3 PRO A 39 -22.561 2.886 15.954 1.00 52.18 H

ATOM 646 HD2 PRO A 39 -20.451 3.423 15.127 1.00 52.18 H

ATOM 647 HD3 PRO A 39 -20.049 4.456 16.530 1.00 52.18 H

ATOM 648 N LEU A 40 -20.836 -0.085 19.216 1.00 58.21 N

ATOM 649 CA LEU A 40 -21.180 -1.372 19.852 1.00 58.21 C

ATOM 650 C LEU A 40 -21.823 -2.518 19.043 1.00 58.21 C

ATOM 651 O LEU A 40 -21.303 -3.629 19.099 1.00 58.21 O

ATOM 652 CB LEU A 40 -21.991 -1.082 21.138 1.00 58.21 C

ATOM 653 CG LEU A 40 -21.183 -1.289 22.436 1.00 58.21 C

ATOM 654 CD1 LEU A 40 -20.092 -0.232 22.622 1.00 58.21 C

ATOM 655 CD2 LEU A 40 -22.119 -1.228 23.642 1.00 58.21 C

ATOM 656 H LEU A 40 -20.262 0.499 19.807 1.00 58.21 H

ATOM 657 HA LEU A 40 -20.226 -1.795 20.167 1.00 58.21 H

ATOM 658 HB2 LEU A 40 -22.843 -1.761 21.171 1.00 58.21 H

ATOM 659 HB3 LEU A 40 -22.394 -0.070 21.119 1.00 58.21 H

ATOM 660 HG LEU A 40 -20.719 -2.275 22.414 1.00 58.21 H

ATOM 661 HD11 LEU A 40 -19.368 -0.288 21.809 1.00 58.21 H

ATOM 662 HD12 LEU A 40 -20.534 0.764 22.649 1.00 58.21 H

ATOM 663 HD13 LEU A 40 -19.567 -0.411 23.561 1.00 58.21 H

ATOM 664 HD21 LEU A 40 -22.882 -2.002 23.560 1.00 58.21 H

ATOM 665 HD22 LEU A 40 -21.553 -1.397 24.558 1.00 58.21 H

ATOM 666 HD23 LEU A 40 -22.602 -0.252 23.696 1.00 58.21 H

ATOM 667 N VAL A 41 -22.927 -2.315 18.315 1.00 58.73 N

ATOM 668 CA VAL A 41 -23.641 -3.420 17.628 1.00 58.73 C

ATOM 669 C VAL A 41 -22.890 -3.868 16.376 1.00 58.73 C

ATOM 670 O VAL A 41 -22.765 -5.060 16.102 1.00 58.73 O

ATOM 671 CB VAL A 41 -25.094 -3.034 17.288 1.00 58.73 C

ATOM 672 CG1 VAL A 41 -25.850 -4.181 16.603 1.00 58.73 C

ATOM 673 CG2 VAL A 41 -25.868 -2.661 18.560 1.00 58.73 C

ATOM 674 H VAL A 41 -23.276 -1.371 18.231 1.00 58.73 H

ATOM 675 HA VAL A 41 -23.677 -4.281 18.296 1.00 58.73 H

ATOM 676 HB VAL A 41 -25.089 -2.172 16.620 1.00 58.73 H

ATOM 677 HG11 VAL A 41 -26.889 -3.896 16.439 1.00 58.73 H

ATOM 678 HG12 VAL A 41 -25.407 -4.406 15.633 1.00 58.73 H

ATOM 679 HG13 VAL A 41 -25.820 -5.075 17.226 1.00 58.73 H

ATOM 680 HG21 VAL A 41 -25.428 -1.784 19.034 1.00 58.73 H

ATOM 681 HG22 VAL A 41 -25.857 -3.494 19.263 1.00 58.73 H

ATOM 682 HG23 VAL A 41 -26.901 -2.424 18.308 1.00 58.73 H

ATOM 683 N ALA A 42 -22.302 -2.913 15.655 1.00 58.68 N

ATOM 684 CA ALA A 42 -21.407 -3.221 14.551 1.00 58.68 C

ATOM 685 C ALA A 42 -20.006 -3.603 15.044 1.00 58.68 C

ATOM 686 O ALA A 42 -19.260 -4.187 14.276 1.00 58.68 O

ATOM 687 CB ALA A 42 -21.379 -2.029 13.592 1.00 58.68 C

ATOM 688 H ALA A 42 -22.398 -1.951 15.948 1.00 58.68 H

ATOM 689 HA ALA A 42 -21.801 -4.078 14.005 1.00 58.68 H

ATOM 690 HB1 ALA A 42 -21.015 -1.143 14.111 1.00 58.68 H

ATOM 691 HB2 ALA A 42 -20.721 -2.249 12.751 1.00 58.68 H

ATOM 692 HB3 ALA A 42 -22.383 -1.836 13.213 1.00 58.68 H

ATOM 693 N GLY A 43 -19.639 -3.324 16.301 1.00 60.60 N

ATOM 694 CA GLY A 43 -18.279 -3.514 16.820 1.00 60.60 C

ATOM 695 C GLY A 43 -17.773 -4.956 16.723 1.00 60.60 C

ATOM 696 O GLY A 43 -16.655 -5.177 16.267 1.00 60.60 O

ATOM 697 H GLY A 43 -20.317 -2.883 16.906 1.00 60.60 H

ATOM 698 HA2 GLY A 43 -17.592 -2.865 16.276 1.00 60.60 H

ATOM 699 HA3 GLY A 43 -18.263 -3.220 17.869 1.00 60.60 H

ATOM 700 N GLY A 44 -18.604 -5.946 17.071 1.00 67.70 N

ATOM 701 CA GLY A 44 -18.233 -7.367 16.975 1.00 67.70 C

ATOM 702 C GLY A 44 -18.084 -7.853 15.528 1.00 67.70 C

ATOM 703 O GLY A 44 -17.087 -8.482 15.171 1.00 67.70 O

ATOM 704 H GLY A 44 -19.510 -5.701 17.444 1.00 67.70 H

ATOM 705 HA2 GLY A 44 -17.289 -7.534 17.494 1.00 67.70 H

ATOM 706 HA3 GLY A 44 -19.003 -7.969 17.457 1.00 67.70 H

ATOM 707 N ILE A 45 -19.041 -7.491 14.669 1.00 68.96 N

ATOM 708 CA ILE A 45 -19.009 -7.814 13.234 1.00 68.96 C

ATOM 709 C ILE A 45 -17.839 -7.090 12.557 1.00 68.96 C

ATOM 710 O ILE A 45 -17.123 -7.685 11.760 1.00 68.96 O

ATOM 711 CB ILE A 45 -20.369 -7.469 12.581 1.00 68.96 C

ATOM 712 CG1 ILE A 45 -21.508 -8.314 13.196 1.00 68.96 C

ATOM 713 CG2 ILE A 45 -20.313 -7.685 11.060 1.00 68.96 C

ATOM 714 CD1 ILE A 45 -22.912 -7.855 12.783 1.00 68.96 C

ATOM 715 H ILE A 45 -19.818 -6.951 15.021 1.00 68.96 H

ATOM 716 HA ILE A 45 -18.837 -8.884 13.120 1.00 68.96 H

ATOM 717 HB ILE A 45 -20.582 -6.416 12.767 1.00 68.96 H

ATOM 718 HG12 ILE A 45 -21.466 -8.247 14.283 1.00 68.96 H

ATOM 719 HG13 ILE A 45 -21.377 -9.360 12.921 1.00 68.96 H

ATOM 720 HG21 ILE A 45 -19.634 -6.952 10.623 1.00 68.96 H

ATOM 721 HG22 ILE A 45 -21.288 -7.519 10.603 1.00 68.96 H

ATOM 722 HG23 ILE A 45 -19.973 -8.694 10.827 1.00 68.96 H

ATOM 723 HD11 ILE A 45 -23.097 -8.062 11.729 1.00 68.96 H

ATOM 724 HD12 ILE A 45 -23.652 -8.399 13.370 1.00 68.96 H

ATOM 725 HD13 ILE A 45 -23.028 -6.788 12.974 1.00 68.96 H

ATOM 726 N CYS A 46 -17.594 -5.835 12.928 1.00 68.73 N

ATOM 727 CA CYS A 46 -16.492 -5.011 12.455 1.00 68.73 C

ATOM 728 C CYS A 46 -15.155 -5.581 12.922 1.00 68.73 C

ATOM 729 O CYS A 46 -14.248 -5.639 12.113 1.00 68.73 O

ATOM 730 CB CYS A 46 -16.706 -3.562 12.917 1.00 68.73 C

ATOM 731 SG CYS A 46 -15.691 -2.264 12.163 1.00 68.73 S

ATOM 732 H CYS A 46 -18.231 -5.413 13.588 1.00 68.73 H

ATOM 733 HA CYS A 46 -16.498 -5.020 11.365 1.00 68.73 H

ATOM 734 HB2 CYS A 46 -16.598 -3.510 14.000 1.00 68.73 H

ATOM 735 HB3 CYS A 46 -17.734 -3.292 12.673 1.00 68.73 H

ATOM 736 N GLN A 47 -15.015 -6.079 14.155 1.00 68.41 N

ATOM 737 CA GLN A 47 -13.796 -6.763 14.606 1.00 68.41 C

ATOM 738 C GLN A 47 -13.512 -8.019 13.769 1.00 68.41 C

ATOM 739 O GLN A 47 -12.404 -8.159 13.257 1.00 68.41 O

ATOM 740 CB GLN A 47 -13.906 -7.120 16.099 1.00 68.41 C

ATOM 741 CG GLN A 47 -13.676 -5.922 17.039 1.00 68.41 C

ATOM 742 CD GLN A 47 -12.292 -5.936 17.681 1.00 68.41 C

ATOM 743 OE1 GLN A 47 -11.912 -6.848 18.392 1.00 68.41 O

ATOM 744 NE2 GLN A 47 -11.469 -4.929 17.478 1.00 68.41 N

ATOM 745 H GLN A 47 -15.777 -5.980 14.812 1.00 68.41 H

ATOM 746 HA GLN A 47 -12.943 -6.097 14.471 1.00 68.41 H

ATOM 747 HB2 GLN A 47 -13.183 -7.901 16.333 1.00 68.41 H

ATOM 748 HB3 GLN A 47 -14.895 -7.534 16.293 1.00 68.41 H

ATOM 749 HG2 GLN A 47 -14.407 -5.970 17.847 1.00 68.41 H

ATOM 750 HG3 GLN A 47 -13.836 -4.984 16.507 1.00 68.41 H

ATOM 751 HE21 GLN A 47 -10.546 -5.048 17.870 1.00 68.41 H

ATOM 752 HE22 GLN A 47 -11.736 -4.141 16.905 1.00 68.41 H

ATOM 753 N CYS A 48 -14.510 -8.886 13.556 1.00 69.24 N

ATOM 754 CA CYS A 48 -14.368 -10.086 12.717 1.00 69.24 C

ATOM 755 C CYS A 48 -14.058 -9.749 11.249 1.00 69.24 C

ATOM 756 O CYS A 48 -13.166 -10.339 10.634 1.00 69.24 O

ATOM 757 CB CYS A 48 -15.662 -10.915 12.806 1.00 69.24 C

ATOM 758 SG CYS A 48 -15.404 -12.360 13.868 1.00 69.24 S

ATOM 759 H CYS A 48 -15.393 -8.719 14.016 1.00 69.24 H

ATOM 760 HA CYS A 48 -13.530 -10.681 13.080 1.00 69.24 H

ATOM 761 HB2 CYS A 48 -16.483 -10.311 13.192 1.00 69.24 H

ATOM 762 HB3 CYS A 48 -15.941 -11.272 11.815 1.00 69.24 H

ATOM 763 HG CYS A 48 -15.123 -11.703 14.996 1.00 69.24 H

ATOM 764 N LEU A 49 -14.785 -8.784 10.680 1.00 69.17 N

ATOM 765 CA LEU A 49 -14.555 -8.302 9.323 1.00 69.17 C

ATOM 766 C LEU A 49 -13.196 -7.627 9.218 1.00 69.17 C

ATOM 767 O LEU A 49 -12.480 -7.913 8.276 1.00 69.17 O

ATOM 768 CB LEU A 49 -15.657 -7.321 8.894 1.00 69.17 C

ATOM 769 CG LEU A 49 -16.994 -7.985 8.527 1.00 69.17 C

ATOM 770 CD1 LEU A 49 -18.065 -6.906 8.414 1.00 69.17 C

ATOM 771 CD2 LEU A 49 -16.929 -8.698 7.174 1.00 69.17 C

ATOM 772 H LEU A 49 -15.520 -8.353 11.222 1.00 69.17 H

ATOM 773 HA LEU A 49 -14.541 -9.152 8.641 1.00 69.17 H

ATOM 774 HB2 LEU A 49 -15.311 -6.756 8.028 1.00 69.17 H

ATOM 775 HB3 LEU A 49 -15.810 -6.606 9.702 1.00 69.17 H

ATOM 776 HG LEU A 49 -17.285 -8.698 9.299 1.00 69.17 H

ATOM 777 HD11 LEU A 49 -18.063 -6.287 9.310 1.00 69.17 H

ATOM 778 HD12 LEU A 49 -17.863 -6.265 7.556 1.00 69.17 H

ATOM 779 HD13 LEU A 49 -19.044 -7.370 8.289 1.00 69.17 H

ATOM 780 HD21 LEU A 49 -16.212 -9.518 7.216 1.00 69.17 H

ATOM 781 HD22 LEU A 49 -17.910 -9.110 6.934 1.00 69.17 H

ATOM 782 HD23 LEU A 49 -16.640 -7.995 6.393 1.00 69.17 H

ATOM 783 N ALA A 50 -12.799 -6.788 10.170 1.00 69.97 N

ATOM 784 CA ALA A 50 -11.501 -6.132 10.186 1.00 69.97 C

ATOM 785 C ALA A 50 -10.388 -7.168 10.220 1.00 69.97 C

ATOM 786 O ALA A 50 -9.475 -7.072 9.413 1.00 69.97 O

ATOM 787 CB ALA A 50 -11.387 -5.179 11.379 1.00 69.97 C

ATOM 788 H ALA A 50 -13.428 -6.595 10.937 1.00 69.97 H

ATOM 789 HA ALA A 50 -11.390 -5.552 9.270 1.00 69.97 H

ATOM 790 HB1 ALA A 50 -10.377 -4.773 11.429 1.00 69.97 H

ATOM 791 HB2 ALA A 50 -12.095 -4.359 11.257 1.00 69.97 H

ATOM 792 HB3 ALA A 50 -11.594 -5.707 12.310 1.00 69.97 H

ATOM 793 N GLU A 51 -10.470 -8.193 11.065 1.00 71.29 N

ATOM 794 CA GLU A 51 -9.473 -9.265 11.104 1.00 71.29 C

ATOM 795 C GLU A 51 -9.342 -9.959 9.743 1.00 71.29 C

ATOM 796 O GLU A 51 -8.234 -10.135 9.248 1.00 71.29 O

ATOM 797 CB GLU A 51 -9.833 -10.268 12.217 1.00 71.29 C

ATOM 798 CG GLU A 51 -8.728 -10.437 13.274 1.00 71.29 C

ATOM 799 CD GLU A 51 -8.028 -11.791 13.141 1.00 71.29 C

ATOM 800 OE1 GLU A 51 -6.836 -11.794 12.739 1.00 71.29 O

ATOM 801 OE2 GLU A 51 -8.707 -12.805 13.411 1.00 71.29 O

ATOM 802 H GLU A 51 -11.233 -8.227 11.726 1.00 71.29 H

ATOM 803 HA GLU A 51 -8.500 -8.823 11.320 1.00 71.29 H

ATOM 804 HB2 GLU A 51 -10.731 -9.934 12.737 1.00 71.29 H

ATOM 805 HB3 GLU A 51 -10.086 -11.232 11.776 1.00 71.29 H

ATOM 806 HG2 GLU A 51 -9.187 -10.379 14.261 1.00 71.29 H

ATOM 807 HG3 GLU A 51 -8.006 -9.623 13.216 1.00 71.29 H

ATOM 808 N ARG A 52 -10.456 -10.269 9.072 1.00 69.28 N

ATOM 809 CA ARG A 52 -10.434 -11.040 7.820 1.00 69.28 C

ATOM 810 C ARG A 52 -10.190 -10.193 6.566 1.00 69.28 C

ATOM 811 O ARG A 52 -9.363 -10.549 5.726 1.00 69.28 O

ATOM 812 CB ARG A 52 -11.720 -11.872 7.769 1.00 69.28 C

ATOM 813 CG ARG A 52 -11.570 -13.105 6.871 1.00 69.28 C

ATOM 814 CD ARG A 52 -12.687 -14.102 7.192 1.00 69.28 C

ATOM 815 NE ARG A 52 -12.514 -15.371 6.463 1.00 69.28 N

ATOM 816 CZ ARG A 52 -13.175 -16.492 6.694 1.00 69.28 C

ATOM 817 NH1 ARG A 52 -14.080 -16.582 7.630 1.00 69.28 N

ATOM 818 NH2 ARG A 52 -12.939 -17.557 5.980 1.00 69.28 N

ATOM 819 H ARG A 52 -11.338 -10.096 9.532 1.00 69.28 H

ATOM 820 HA ARG A 52 -9.587 -11.724 7.883 1.00 69.28 H

ATOM 821 HB2 ARG A 52 -11.937 -12.217 8.780 1.00 69.28 H

ATOM 822 HB3 ARG A 52 -12.557 -11.258 7.436 1.00 69.28 H

ATOM 823 HG2 ARG A 52 -10.611 -13.583 7.071 1.00 69.28 H

ATOM 824 HG3 ARG A 52 -11.613 -12.810 5.822 1.00 69.28 H

ATOM 825 HD2 ARG A 52 -12.673 -14.300 8.264 1.00 69.28 H

ATOM 826 HD3 ARG A 52 -13.647 -13.655 6.931 1.00 69.28 H

ATOM 827 HE ARG A 52 -11.823 -15.385 5.726 1.00 69.28 H

ATOM 828 HH11 ARG A 52 -14.262 -15.778 8.213 1.00 69.28 H

ATOM 829 HH12 ARG A 52 -14.572 -17.448 7.798 1.00 69.28 H

ATOM 830 HH21 ARG A 52 -12.251 -17.537 5.241 1.00 69.28 H

ATOM 831 HH22 ARG A 52 -13.437 -18.413 6.176 1.00 69.28 H

ATOM 832 N TYR A 53 -10.879 -9.061 6.441 1.00 69.12 N

ATOM 833 CA TYR A 53 -10.735 -8.113 5.335 1.00 69.12 C

ATOM 834 C TYR A 53 -9.426 -7.346 5.406 1.00 69.12 C

ATOM 835 O TYR A 53 -8.848 -7.112 4.350 1.00 69.12 O

ATOM 836 CB TYR A 53 -11.890 -7.098 5.252 1.00 69.12 C

ATOM 837 CG TYR A 53 -13.057 -7.556 4.410 1.00 69.12 C

ATOM 838 CD1 TYR A 53 -13.177 -7.073 3.093 1.00 69.12 C

ATOM 839 CD2 TYR A 53 -14.007 -8.458 4.924 1.00 69.12 C

ATOM 840 CE1 TYR A 53 -14.251 -7.482 2.283 1.00 69.12 C

ATOM 841 CE2 TYR A 53 -15.078 -8.879 4.110 1.00 69.12 C

ATOM 842 CZ TYR A 53 -15.203 -8.389 2.793 1.00 69.12 C

ATOM 843 OH TYR A 53 -16.239 -8.797 2.018 1.00 69.12 O

ATOM 844 H TYR A 53 -11.511 -8.820 7.191 1.00 69.12 H

ATOM 845 HA TYR A 53 -10.717 -8.672 4.400 1.00 69.12 H

ATOM 846 HB2 TYR A 53 -12.234 -6.786 6.238 1.00 69.12 H

ATOM 847 HB3 TYR A 53 -11.508 -6.188 4.789 1.00 69.12 H

ATOM 848 HD1 TYR A 53 -12.447 -6.380 2.700 1.00 69.12 H

ATOM 849 HD2 TYR A 53 -13.917 -8.818 5.938 1.00 69.12 H

ATOM 850 HE1 TYR A 53 -14.347 -7.104 1.276 1.00 69.12 H

ATOM 851 HE2 TYR A 53 -15.821 -9.568 4.485 1.00 69.12 H

ATOM 852 HH TYR A 53 -16.245 -8.374 1.156 1.00 69.12 H

ATOM 853 N SER A 54 -8.924 -6.972 6.592 1.00 67.45 N

ATOM 854 CA SER A 54 -7.642 -6.256 6.658 1.00 67.45 C

ATOM 855 C SER A 54 -6.539 -7.098 6.041 1.00 67.45 C

ATOM 856 O SER A 54 -5.780 -6.575 5.242 1.00 67.45 O

ATOM 857 CB SER A 54 -7.232 -5.817 8.068 1.00 67.45 C

ATOM 858 OG SER A 54 -6.948 -6.924 8.898 1.00 67.45 O

ATOM 859 H SER A 54 -9.415 -7.189 7.448 1.00 67.45 H

ATOM 860 HA SER A 54 -7.732 -5.354 6.053 1.00 67.45 H

ATOM 861 HB2 SER A 54 -6.333 -5.204 7.999 1.00 67.45 H

ATOM 862 HB3 SER A 54 -8.021 -5.208 8.510 1.00 67.45 H

ATOM 863 HG SER A 54 -7.800 -7.223 9.223 1.00 67.45 H

ATOM 864 N VAL A 55 -6.492 -8.407 6.300 1.00 67.82 N

ATOM 865 CA VAL A 55 -5.454 -9.274 5.729 1.00 67.82 C

ATOM 866 C VAL A 55 -5.517 -9.279 4.216 1.00 67.82 C

ATOM 867 O VAL A 55 -4.532 -8.950 3.570 1.00 67.82 O

ATOM 868 CB VAL A 55 -5.545 -10.707 6.271 1.00 67.82 C

ATOM 869 CG1 VAL A 55 -4.433 -11.606 5.709 1.00 67.82 C

ATOM 870 CG2 VAL A 55 -5.338 -10.636 7.772 1.00 67.82 C

ATOM 871 H VAL A 55 -7.132 -8.783 6.984 1.00 67.82 H

ATOM 872 HA VAL A 55 -4.481 -8.863 5.997 1.00 67.82 H

ATOM 873 HB VAL A 55 -6.519 -11.145 6.052 1.00 67.82 H

ATOM 874 HG11 VAL A 55 -4.566 -11.741 4.635 1.00 67.82 H

ATOM 875 HG12 VAL A 55 -3.455 -11.161 5.890 1.00 67.82 H

ATOM 876 HG13 VAL A 55 -4.481 -12.589 6.177 1.00 67.82 H

ATOM 877 HG21 VAL A 55 -4.418 -10.075 7.937 1.00 67.82 H

ATOM 878 HG22 VAL A 55 -5.303 -11.635 8.206 1.00 67.82 H

ATOM 879 HG23 VAL A 55 -6.142 -10.075 8.247 1.00 67.82 H

ATOM 880 N ILE A 56 -6.675 -9.608 3.652 1.00 71.47 N

ATOM 881 CA ILE A 56 -6.821 -9.788 2.206 1.00 71.47 C

ATOM 882 C ILE A 56 -6.668 -8.450 1.481 1.00 71.47 C

ATOM 883 O ILE A 56 -5.939 -8.347 0.492 1.00 71.47 O

ATOM 884 CB ILE A 56 -8.183 -10.450 1.915 1.00 71.47 C

ATOM 885 CG1 ILE A 56 -8.245 -11.862 2.549 1.00 71.47 C

ATOM 886 CG2 ILE A 56 -8.441 -10.532 0.398 1.00 71.47 C

ATOM 887 CD1 ILE A 56 -9.678 -12.367 2.743 1.00 71.47 C

ATOM 888 H ILE A 56 -7.458 -9.822 4.253 1.00 71.47 H

ATOM 889 HA ILE A 56 -6.029 -10.446 1.847 1.00 71.47 H

ATOM 890 HB ILE A 56 -8.962 -9.833 2.361 1.00 71.47 H

ATOM 891 HG12 ILE A 56 -7.785 -11.860 3.537 1.00 71.47 H

ATOM 892 HG13 ILE A 56 -7.691 -12.569 1.931 1.00 71.47 H

ATOM 893 HG21 ILE A 56 -7.633 -11.075 -0.092 1.00 71.47 H

ATOM 894 HG22 ILE A 56 -9.382 -11.046 0.202 1.00 71.47 H

ATOM 895 HG23 ILE A 56 -8.517 -9.535 -0.036 1.00 71.47 H

ATOM 896 HD11 ILE A 56 -10.246 -11.657 3.344 1.00 71.47 H

ATOM 897 HD12 ILE A 56 -10.171 -12.499 1.779 1.00 71.47 H

ATOM 898 HD13 ILE A 56 -9.655 -13.327 3.258 1.00 71.47 H

ATOM 899 N LEU A 57 -7.338 -7.411 1.979 1.00 69.56 N

ATOM 900 CA LEU A 57 -7.343 -6.103 1.344 1.00 69.56 C

ATOM 901 C LEU A 57 -5.975 -5.435 1.472 1.00 69.56 C

ATOM 902 O LEU A 57 -5.490 -4.891 0.491 1.00 69.56 O

ATOM 903 CB LEU A 57 -8.477 -5.241 1.924 1.00 69.56 C

ATOM 904 CG LEU A 57 -9.006 -4.209 0.917 1.00 69.56 C

ATOM 905 CD1 LEU A 57 -9.933 -4.875 -0.107 1.00 69.56 C

ATOM 906 CD2 LEU A 57 -9.807 -3.131 1.645 1.00 69.56 C

ATOM 907 H LEU A 57 -7.877 -7.530 2.825 1.00 69.56 H

ATOM 908 HA LEU A 57 -7.531 -6.253 0.281 1.00 69.56 H

ATOM 909 HB2 LEU A 57 -9.315 -5.877 2.208 1.00 69.56 H

ATOM 910 HB3 LEU A 57 -8.116 -4.741 2.822 1.00 69.56 H

ATOM 911 HG LEU A 57 -8.173 -3.732 0.401 1.00 69.56 H

ATOM 912 HD11 LEU A 57 -10.775 -5.350 0.396 1.00 69.56 H

ATOM 913 HD12 LEU A 57 -10.313 -4.122 -0.798 1.00 69.56 H

ATOM 914 HD13 LEU A 57 -9.389 -5.622 -0.685 1.00 69.56 H

ATOM 915 HD21 LEU A 57 -9.165 -2.615 2.358 1.00 69.56 H

ATOM 916 HD22 LEU A 57 -10.646 -3.580 2.176 1.00 69.56 H

ATOM 917 HD23 LEU A 57 -10.184 -2.403 0.927 1.00 69.56 H

ATOM 918 N LEU A 58 -5.295 -5.531 2.619 1.00 69.69 N

ATOM 919 CA LEU A 58 -3.928 -5.026 2.708 1.00 69.69 C

ATOM 920 C LEU A 58 -2.970 -5.809 1.826 1.00 69.69 C

ATOM 921 O LEU A 58 -2.127 -5.184 1.198 1.00 69.69 O

ATOM 922 CB LEU A 58 -3.339 -5.094 4.112 1.00 69.69 C

ATOM 923 CG LEU A 58 -3.547 -3.849 4.966 1.00 69.69 C

ATOM 924 CD1 LEU A 58 -4.957 -3.662 5.534 1.00 69.69 C

ATOM 925 CD2 LEU A 58 -2.498 -3.952 6.077 1.00 69.69 C

ATOM 926 H LEU A 58 -5.692 -6.017 3.411 1.00 69.69 H

ATOM 927 HA LEU A 58 -3.908 -3.992 2.362 1.00 69.69 H

ATOM 928 HB2 LEU A 58 -2.263 -5.175 3.958 1.00 69.69 H

ATOM 929 HB3 LEU A 58 -3.615 -6.009 4.636 1.00 69.69 H

ATOM 930 HG LEU A 58 -3.312 -2.983 4.347 1.00 69.69 H

ATOM 931 HD11 LEU A 58 -5.151 -4.422 6.291 1.00 69.69 H

ATOM 932 HD12 LEU A 58 -5.694 -3.746 4.735 1.00 69.69 H

ATOM 933 HD13 LEU A 58 -5.055 -2.679 5.995 1.00 69.69 H

ATOM 934 HD21 LEU A 58 -1.929 -3.023 6.102 1.00 69.69 H

ATOM 935 HD22 LEU A 58 -2.972 -4.197 7.027 1.00 69.69 H

ATOM 936 HD23 LEU A 58 -1.760 -4.737 5.909 1.00 69.69 H

ATOM 937 N ASP A 59 -3.026 -7.142 1.807 1.00 71.18 N

ATOM 938 CA ASP A 59 -2.050 -7.918 1.035 1.00 71.18 C

ATOM 939 C ASP A 59 -2.166 -7.598 -0.455 1.00 71.18 C

ATOM 940 O ASP A 59 -1.169 -7.360 -1.137 1.00 71.18 O

ATOM 941 CB ASP A 59 -2.212 -9.428 1.274 1.00 71.18 C

ATOM 942 CG ASP A 59 -0.845 -10.071 1.442 1.00 71.18 C

ATOM 943 OD1 ASP A 59 -0.125 -10.271 0.444 1.00 71.18 O

ATOM 944 OD2 ASP A 59 -0.426 -10.242 2.609 1.00 71.18 O

ATOM 945 H ASP A 59 -3.701 -7.631 2.377 1.00 71.18 H

ATOM 946 HA ASP A 59 -1.053 -7.620 1.358 1.00 71.18 H

ATOM 947 HB2 ASP A 59 -2.753 -9.898 0.453 1.00 71.18 H

ATOM 948 HB3 ASP A 59 -2.764 -9.619 2.194 1.00 71.18 H

ATOM 949 N THR A 60 -3.402 -7.492 -0.944 1.00 70.55 N

ATOM 950 CA THR A 60 -3.686 -7.113 -2.331 1.00 70.55 C

ATOM 951 C THR A 60 -3.316 -5.657 -2.617 1.00 70.55 C

ATOM 952 O THR A 60 -2.648 -5.373 -3.622 1.00 70.55 O

ATOM 953 CB THR A 60 -5.155 -7.373 -2.693 1.00 70.55 C

ATOM 954 OG1 THR A 60 -6.034 -6.772 -1.780 1.00 70.55 O

ATOM 955 CG2 THR A 60 -5.485 -8.864 -2.723 1.00 70.55 C

ATOM 956 H THR A 60 -4.174 -7.679 -0.319 1.00 70.55 H

ATOM 957 HA THR A 60 -3.068 -7.727 -2.986 1.00 70.55 H

ATOM 958 HB THR A 60 -5.345 -6.961 -3.684 1.00 70.55 H

ATOM 959 HG1 THR A 60 -6.048 -7.320 -0.992 1.00 70.55 H

ATOM 960 HG21 THR A 60 -6.530 -8.999 -3.001 1.00 70.55 H

ATOM 961 HG22 THR A 60 -4.858 -9.362 -3.463 1.00 70.55 H

ATOM 962 HG23 THR A 60 -5.308 -9.326 -1.752 1.00 70.55 H

ATOM 963 N LEU A 61 -3.698 -4.732 -1.731 1.00 72.56 N

ATOM 964 CA LEU A 61 -3.475 -3.306 -1.927 1.00 72.56 C

ATOM 965 C LEU A 61 -1.993 -2.957 -1.789 1.00 72.56 C

ATOM 966 O LEU A 61 -1.434 -2.352 -2.699 1.00 72.56 O

ATOM 967 CB LEU A 61 -4.371 -2.486 -0.980 1.00 72.56 C

ATOM 968 CG LEU A 61 -4.668 -1.068 -1.486 1.00 72.56 C

ATOM 969 CD1 LEU A 61 -5.769 -1.101 -2.551 1.00 72.56 C

ATOM 970 CD2 LEU A 61 -5.153 -0.185 -0.339 1.00 72.56 C

ATOM 971 H LEU A 61 -4.275 -5.020 -0.953 1.00 72.56 H

ATOM 972 HA LEU A 61 -3.775 -3.086 -2.952 1.00 72.56 H

ATOM 973 HB2 LEU A 61 -3.911 -2.450 0.007 1.00 72.56 H

ATOM 974 HB3 LEU A 61 -5.338 -2.979 -0.888 1.00 72.56 H

ATOM 975 HG LEU A 61 -3.765 -0.628 -1.909 1.00 72.56 H

ATOM 976 HD11 LEU A 61 -5.951 -0.090 -2.918 1.00 72.56 H

ATOM 977 HD12 LEU A 61 -5.475 -1.730 -3.391 1.00 72.56 H

ATOM 978 HD13 LEU A 61 -6.693 -1.490 -2.125 1.00 72.56 H

ATOM 979 HD21 LEU A 61 -4.375 -0.106 0.421 1.00 72.56 H

ATOM 980 HD22 LEU A 61 -5.380 0.814 -0.711 1.00 72.56 H

ATOM 981 HD23 LEU A 61 -6.050 -0.611 0.110 1.00 72.56 H

ATOM 982 N LEU A 62 -1.332 -3.373 -0.705 1.00 70.46 N

ATOM 983 CA LEU A 62 0.102 -3.161 -0.516 1.00 70.46 C

ATOM 984 C LEU A 62 0.909 -3.970 -1.532 1.00 70.46 C

ATOM 985 O LEU A 62 1.869 -3.441 -2.061 1.00 70.46 O

ATOM 986 CB LEU A 62 0.581 -3.464 0.923 1.00 70.46 C

ATOM 987 CG LEU A 62 0.319 -2.360 1.967 1.00 70.46 C

ATOM 988 CD1 LEU A 62 -1.140 -2.261 2.412 1.00 70.46 C

ATOM 989 CD2 LEU A 62 1.174 -2.602 3.210 1.00 70.46 C

ATOM 990 H LEU A 62 -1.829 -3.924 -0.020 1.00 70.46 H

ATOM 991 HA LEU A 62 0.330 -2.115 -0.722 1.00 70.46 H

ATOM 992 HB2 LEU A 62 1.663 -3.584 0.867 1.00 70.46 H

ATOM 993 HB3 LEU A 62 0.188 -4.420 1.270 1.00 70.46 H

ATOM 994 HG LEU A 62 0.633 -1.402 1.553 1.00 70.46 H

ATOM 995 HD11 LEU A 62 -1.458 -3.217 2.828 1.00 70.46 H

ATOM 996 HD12 LEU A 62 -1.774 -2.006 1.562 1.00 70.46 H

ATOM 997 HD13 LEU A 62 -1.246 -1.483 3.168 1.00 70.46 H

ATOM 998 HD21 LEU A 62 1.019 -1.799 3.930 1.00 70.46 H

ATOM 999 HD22 LEU A 62 2.230 -2.622 2.941 1.00 70.46 H

ATOM 1000 HD23 LEU A 62 0.904 -3.553 3.670 1.00 70.46 H

ATOM 1001 N GLY A 63 0.541 -5.207 -1.863 1.00 69.94 N

ATOM 1002 CA GLY A 63 1.387 -6.071 -2.692 1.00 69.94 C

ATOM 1003 C GLY A 63 1.487 -5.671 -4.168 1.00 69.94 C

ATOM 1004 O GLY A 63 2.569 -5.759 -4.758 1.00 69.94 O

ATOM 1005 H GLY A 63 -0.256 -5.627 -1.406 1.00 69.94 H

ATOM 1006 HA2 GLY A 63 2.394 -6.085 -2.274 1.00 69.94 H

ATOM 1007 HA3 GLY A 63 0.985 -7.084 -2.646 1.00 69.94 H

ATOM 1008 N ARG A 64 0.376 -5.256 -4.796 1.00 70.74 N

ATOM 1009 CA ARG A 64 0.340 -4.924 -6.240 1.00 70.74 C

ATOM 1010 C ARG A 64 -0.175 -3.530 -6.553 1.00 70.74 C

ATOM 1011 O ARG A 64 0.381 -2.891 -7.445 1.00 70.74 O

ATOM 1012 CB ARG A 64 -0.496 -5.949 -7.027 1.00 70.74 C

ATOM 1013 CG ARG A 64 0.301 -7.195 -7.431 1.00 70.74 C

ATOM 1014 CD ARG A 64 -0.527 -8.050 -8.400 1.00 70.74 C

ATOM 1015 NE ARG A 64 0.132 -9.339 -8.697 1.00 70.74 N

ATOM 1016 CZ ARG A 64 0.216 -9.953 -9.868 1.00 70.74 C

ATOM 1017 NH1 ARG A 64 -0.254 -9.436 -10.969 1.00 70.74 N

ATOM 1018 NH2 ARG A 64 0.780 -11.125 -9.957 1.00 70.74 N

ATOM 1019 H ARG A 64 -0.474 -5.222 -4.252 1.00 70.74 H

ATOM 1020 HA ARG A 64 1.355 -4.929 -6.636 1.00 70.74 H

ATOM 1021 HB2 ARG A 64 -0.844 -5.474 -7.945 1.00 70.74 H

ATOM 1022 HB3 ARG A 64 -1.374 -6.235 -6.449 1.00 70.74 H

ATOM 1023 HG2 ARG A 64 0.539 -7.772 -6.537 1.00 70.74 H

ATOM 1024 HG3 ARG A 64 1.228 -6.895 -7.920 1.00 70.74 H

ATOM 1025 HD2 ARG A 64 -1.498 -8.253 -7.947 1.00 70.74 H

ATOM 1026 HD3 ARG A 64 -0.693 -7.475 -9.311 1.00 70.74 H

ATOM 1027 HE ARG A 64 0.474 -9.853 -7.898 1.00 70.74 H

ATOM 1028 HH11 ARG A 64 -0.218 -9.953 -11.836 1.00 70.74 H

ATOM 1029 HH12 ARG A 64 -0.779 -8.574 -10.923 1.00 70.74 H

ATOM 1030 HH21 ARG A 64 1.093 -11.612 -9.129 1.00 70.74 H

ATOM 1031 HH22 ARG A 64 0.786 -11.616 -10.840 1.00 70.74 H

ATOM 1032 N MET A 65 -1.208 -3.061 -5.857 1.00 77.20 N

ATOM 1033 CA MET A 65 -1.884 -1.816 -6.238 1.00 77.20 C

ATOM 1034 C MET A 65 -1.099 -0.573 -5.825 1.00 77.20 C

ATOM 1035 O MET A 65 -0.855 0.290 -6.659 1.00 77.20 O

ATOM 1036 CB MET A 65 -3.303 -1.769 -5.663 1.00 77.20 C

ATOM 1037 CG MET A 65 -4.224 -2.848 -6.239 1.00 77.20 C

ATOM 1038 SD MET A 65 -4.561 -2.643 -8.009 1.00 77.20 S

ATOM 1039 CE MET A 65 -5.919 -3.827 -8.210 1.00 77.20 C

ATOM 1040 H MET A 65 -1.597 -3.620 -5.111 1.00 77.20 H

ATOM 1041 HA MET A 65 -1.967 -1.780 -7.324 1.00 77.20 H

ATOM 1042 HB2 MET A 65 -3.256 -1.866 -4.578 1.00 77.20 H

ATOM 1043 HB3 MET A 65 -3.742 -0.796 -5.886 1.00 77.20 H

ATOM 1044 HG2 MET A 65 -3.796 -3.834 -6.062 1.00 77.20 H

ATOM 1045 HG3 MET A 65 -5.173 -2.795 -5.706 1.00 77.20 H

ATOM 1046 HE1 MET A 65 -6.242 -3.838 -9.251 1.00 77.20 H

ATOM 1047 HE2 MET A 65 -6.759 -3.532 -7.580 1.00 77.20 H

ATOM 1048 HE3 MET A 65 -5.586 -4.825 -7.925 1.00 77.20 H

ATOM 1049 N LEU A 66 -0.637 -0.486 -4.577 1.00 80.19 N

ATOM 1050 CA LEU A 66 0.154 0.642 -4.090 1.00 80.19 C

ATOM 1051 C LEU A 66 1.448 0.883 -4.885 1.00 80.19 C

ATOM 1052 O LEU A 66 1.671 2.038 -5.233 1.00 80.19 O

ATOM 1053 CB LEU A 66 0.482 0.482 -2.600 1.00 80.19 C

ATOM 1054 CG LEU A 66 -0.419 1.253 -1.620 1.00 80.19 C

ATOM 1055 CD1 LEU A 66 -0.892 0.375 -0.468 1.00 80.19 C

ATOM 1056 CD2 LEU A 66 0.372 2.407 -0.992 1.00 80.19 C

ATOM 1057 H LEU A 66 -0.871 -1.222 -3.926 1.00 80.19 H

ATOM 1058 HA LEU A 66 -0.442 1.546 -4.211 1.00 80.19 H

ATOM 1059 HB2 LEU A 66 1.474 0.918 -2.479 1.00 80.19 H

ATOM 1060 HB3 LEU A 66 0.530 -0.575 -2.341 1.00 80.19 H

ATOM 1061 HG LEU A 66 -1.291 1.650 -2.140 1.00 80.19 H

ATOM 1062 HD11 LEU A 66 -0.030 0.005 0.086 1.00 80.19 H

ATOM 1063 HD12 LEU A 66 -1.525 0.957 0.202 1.00 80.19 H

ATOM 1064 HD13 LEU A 66 -1.482 -0.455 -0.857 1.00 80.19 H

ATOM 1065 HD21 LEU A 66 1.221 2.033 -0.420 1.00 80.19 H

ATOM 1066 HD22 LEU A 66 -0.277 2.975 -0.326 1.00 80.19 H

ATOM 1067 HD23 LEU A 66 0.725 3.081 -1.774 1.00 80.19 H

ATOM 1068 N PRO A 67 2.304 -0.109 -5.221 1.00 77.31 N

ATOM 1069 CA PRO A 67 3.508 0.186 -5.997 1.00 77.31 C

ATOM 1070 C PRO A 67 3.163 0.711 -7.394 1.00 77.31 C

ATOM 1071 O PRO A 67 3.869 1.566 -7.922 1.00 77.31 O

ATOM 1072 CB PRO A 67 4.316 -1.116 -6.020 1.00 77.31 C

ATOM 1073 CG PRO A 67 3.262 -2.205 -5.856 1.00 77.31 C

ATOM 1074 CD PRO A 67 2.214 -1.543 -4.964 1.00 77.31 C

ATOM 1075 HA PRO A 67 4.093 0.961 -5.503 1.00 77.31 H

ATOM 1076 HB2 PRO A 67 4.888 -1.236 -6.941 1.00 77.31 H

ATOM 1077 HB3 PRO A 67 4.991 -1.149 -5.165 1.00 77.31 H

ATOM 1078 HG2 PRO A 67 2.835 -2.441 -6.831 1.00 77.31 H

ATOM 1079 HG3 PRO A 67 3.674 -3.100 -5.390 1.00 77.31 H

ATOM 1080 HD2 PRO A 67 1.229 -1.947 -5.200 1.00 77.31 H

ATOM 1081 HD3 PRO A 67 2.443 -1.767 -3.922 1.00 77.31 H

ATOM 1082 N GLN A 68 2.048 0.263 -7.979 1.00 79.41 N

ATOM 1083 CA GLN A 68 1.554 0.804 -9.241 1.00 79.41 C

ATOM 1084 C GLN A 68 1.007 2.222 -9.084 1.00 79.41 C

ATOM 1085 O GLN A 68 1.374 3.086 -9.873 1.00 79.41 O

ATOM 1086 CB GLN A 68 0.476 -0.114 -9.821 1.00 79.41 C

ATOM 1087 CG GLN A 68 1.072 -1.435 -10.312 1.00 79.41 C

ATOM 1088 CD GLN A 68 0.025 -2.346 -10.941 1.00 79.41 C

ATOM 1089 OE1 GLN A 68 -1.155 -2.057 -11.001 1.00 79.41 O

ATOM 1090 NE2 GLN A 68 0.418 -3.472 -11.494 1.00 79.41 N

ATOM 1091 H GLN A 68 1.463 -0.391 -7.480 1.00 79.41 H

ATOM 1092 HA GLN A 68 2.379 0.863 -9.952 1.00 79.41 H

ATOM 1093 HB2 GLN A 68 -0.291 -0.315 -9.073 1.00 79.41 H

ATOM 1094 HB3 GLN A 68 0.009 0.389 -10.667 1.00 79.41 H

ATOM 1095 HG2 GLN A 68 1.564 -1.963 -9.495 1.00 79.41 H

ATOM 1096 HG3 GLN A 68 1.820 -1.195 -11.068 1.00 79.41 H

ATOM 1097 HE21 GLN A 68 1.388 -3.755 -11.471 1.00 79.41 H

ATOM 1098 HE22 GLN A 68 -0.314 -3.988 -11.961 1.00 79.41 H

ATOM 1099 N LEU A 69 0.166 2.477 -8.078 1.00 82.57 N

ATOM 1100 CA LEU A 69 -0.425 3.791 -7.824 1.00 82.57 C

ATOM 1101 C LEU A 69 0.637 4.828 -7.478 1.00 82.57 C

ATOM 1102 O LEU A 69 0.629 5.903 -8.060 1.00 82.57 O

ATOM 1103 CB LEU A 69 -1.464 3.729 -6.685 1.00 82.57 C

ATOM 1104 CG LEU A 69 -2.914 3.858 -7.177 1.00 82.57 C

ATOM 1105 CD1 LEU A 69 -3.451 2.517 -7.676 1.00 82.57 C

ATOM 1106 CD2 LEU A 69 -3.805 4.362 -6.044 1.00 82.57 C

ATOM 1107 H LEU A 69 -0.108 1.713 -7.477 1.00 82.57 H

ATOM 1108 HA LEU A 69 -0.908 4.136 -8.738 1.00 82.57 H

ATOM 1109 HB2 LEU A 69 -1.277 4.565 -6.011 1.00 82.57 H

ATOM 1110 HB3 LEU A 69 -1.345 2.822 -6.092 1.00 82.57 H

ATOM 1111 HG LEU A 69 -2.962 4.585 -7.988 1.00 82.57 H

ATOM 1112 HD11 LEU A 69 -3.448 1.789 -6.865 1.00 82.57 H

ATOM 1113 HD12 LEU A 69 -4.471 2.641 -8.039 1.00 82.57 H

ATOM 1114 HD13 LEU A 69 -2.825 2.147 -8.488 1.00 82.57 H

ATOM 1115 HD21 LEU A 69 -4.834 4.443 -6.392 1.00 82.57 H

ATOM 1116 HD22 LEU A 69 -3.474 5.350 -5.721 1.00 82.57 H

ATOM 1117 HD23 LEU A 69 -3.767 3.675 -5.199 1.00 82.57 H

ATOM 1118 N VAL A 70 1.561 4.513 -6.569 1.00 83.72 N

ATOM 1119 CA VAL A 70 2.604 5.446 -6.129 1.00 83.72 C

ATOM 1120 C VAL A 70 3.530 5.778 -7.292 1.00 83.72 C

ATOM 1121 O VAL A 70 3.748 6.950 -7.570 1.00 83.72 O

ATOM 1122 CB VAL A 70 3.386 4.896 -4.921 1.00 83.72 C

ATOM 1123 CG1 VAL A 70 4.543 5.821 -4.526 1.00 83.72 C

ATOM 1124 CG2 VAL A 70 2.475 4.764 -3.692 1.00 83.72 C

ATOM 1125 H VAL A 70 1.506 3.609 -6.121 1.00 83.72 H

ATOM 1126 HA VAL A 70 2.130 6.379 -5.824 1.00 83.72 H

ATOM 1127 HB VAL A 70 3.791 3.915 -5.170 1.00 83.72 H

ATOM 1128 HG11 VAL A 70 5.001 5.469 -3.602 1.00 83.72 H

ATOM 1129 HG12 VAL A 70 4.177 6.836 -4.370 1.00 83.72 H

ATOM 1130 HG13 VAL A 70 5.308 5.831 -5.302 1.00 83.72 H

ATOM 1131 HG21 VAL A 70 1.595 4.161 -3.918 1.00 83.72 H

ATOM 1132 HG22 VAL A 70 2.142 5.750 -3.369 1.00 83.72 H

ATOM 1133 HG23 VAL A 70 3.022 4.289 -2.878 1.00 83.72 H

ATOM 1134 N CYS A 71 4.007 4.791 -8.054 1.00 85.73 N

ATOM 1135 CA CYS A 71 4.841 5.085 -9.222 1.00 85.73 C

ATOM 1136 C CYS A 71 4.054 5.783 -10.341 1.00 85.73 C

ATOM 1137 O CYS A 71 4.598 6.628 -11.045 1.00 85.73 O

ATOM 1138 CB CYS A 71 5.515 3.801 -9.703 1.00 85.73 C

ATOM 1139 SG CYS A 71 6.600 3.080 -8.449 1.00 85.73 S

ATOM 1140 H CYS A 71 3.826 3.828 -7.808 1.00 85.73 H

ATOM 1141 HA CYS A 71 5.626 5.777 -8.917 1.00 85.73 H

ATOM 1142 HB2 CYS A 71 6.110 4.022 -10.589 1.00 85.73 H

ATOM 1143 HB3 CYS A 71 4.749 3.071 -9.967 1.00 85.73 H

ATOM 1144 N ARG A 72 2.757 5.502 -10.499 1.00 84.02 N

ATOM 1145 CA ARG A 72 1.893 6.248 -11.423 1.00 84.02 C

ATOM 1146 C ARG A 72 1.643 7.679 -10.949 1.00 84.02 C

ATOM 1147 O ARG A 72 1.521 8.562 -11.780 1.00 84.02 O

ATOM 1148 CB ARG A 72 0.592 5.471 -11.633 1.00 84.02 C

ATOM 1149 CG ARG A 72 -0.288 6.050 -12.751 1.00 84.02 C

ATOM 1150 CD ARG A 72 -1.574 5.238 -12.939 1.00 84.02 C

ATOM 1151 NE ARG A 72 -1.318 3.802 -13.170 1.00 84.02 N

ATOM 1152 CZ ARG A 72 -2.217 2.834 -13.107 1.00 84.02 C

ATOM 1153 NH1 ARG A 72 -3.487 3.087 -12.951 1.00 84.02 N

ATOM 1154 NH2 ARG A 72 -1.854 1.586 -13.201 1.00 84.02 N

ATOM 1155 H ARG A 72 2.340 4.779 -9.930 1.00 84.02 H

ATOM 1156 HA ARG A 72 2.390 6.325 -12.389 1.00 84.02 H

ATOM 1157 HB2 ARG A 72 0.026 5.444 -10.702 1.00 84.02 H

ATOM 1158 HB3 ARG A 72 0.864 4.453 -11.913 1.00 84.02 H

ATOM 1159 HG2 ARG A 72 -0.572 7.072 -12.500 1.00 84.02 H

ATOM 1160 HG3 ARG A 72 0.265 6.076 -13.691 1.00 84.02 H

ATOM 1161 HD2 ARG A 72 -2.122 5.652 -13.785 1.00 84.02 H

ATOM 1162 HD3 ARG A 72 -2.181 5.360 -12.042 1.00 84.02 H

ATOM 1163 HE ARG A 72 -0.360 3.543 -13.358 1.00 84.02 H

ATOM 1164 HH11 ARG A 72 -3.791 4.049 -12.909 1.00 84.02 H

ATOM 1165 HH12 ARG A 72 -4.165 2.339 -12.909 1.00 84.02 H

ATOM 1166 HH21 ARG A 72 -0.893 1.350 -13.404 1.00 84.02 H

ATOM 1167 HH22 ARG A 72 -2.534 0.844 -13.116 1.00 84.02 H

ATOM 1168 N LEU A 73 1.601 7.940 -9.649 1.00 84.01 N

ATOM 1169 CA LEU A 73 1.368 9.278 -9.114 1.00 84.01 C

ATOM 1170 C LEU A 73 2.651 10.118 -9.119 1.00 84.01 C

ATOM 1171 O LEU A 73 2.649 11.238 -9.616 1.00 84.01 O

ATOM 1172 CB LEU A 73 0.748 9.140 -7.713 1.00 84.01 C

ATOM 1173 CG LEU A 73 0.176 10.453 -7.153 1.00 84.01 C

ATOM 1174 CD1 LEU A 73 -1.042 10.929 -7.950 1.00 84.01 C

ATOM 1175 CD2 LEU A 73 -0.260 10.231 -5.706 1.00 84.01 C

ATOM 1176 H LEU A 73 1.626 7.165 -9.001 1.00 84.01 H

ATOM 1177 HA LEU A 73 0.653 9.783 -9.763 1.00 84.01 H

ATOM 1178 HB2 LEU A 73 1.505 8.758 -7.029 1.00 84.01 H

ATOM 1179 HB3 LEU A 73 -0.064 8.414 -7.753 1.00 84.01 H

ATOM 1180 HG LEU A 73 0.944 11.227 -7.168 1.00 84.01 H

ATOM 1181 HD11 LEU A 73 -1.793 10.141 -8.004 1.00 84.01 H

ATOM 1182 HD12 LEU A 73 -1.478 11.800 -7.461 1.00 84.01 H

ATOM 1183 HD13 LEU A 73 -0.745 11.228 -8.955 1.00 84.01 H

ATOM 1184 HD21 LEU A 73 -1.044 9.475 -5.657 1.00 84.01 H

ATOM 1185 HD22 LEU A 73 0.592 9.911 -5.107 1.00 84.01 H

ATOM 1186 HD23 LEU A 73 -0.639 11.165 -5.291 1.00 84.01 H

ATOM 1187 N VAL A 74 3.757 9.555 -8.623 1.00 83.23 N

ATOM 1188 CA VAL A 74 5.045 10.251 -8.482 1.00 83.23 C

ATOM 1189 C VAL A 74 5.746 10.389 -9.836 1.00 83.23 C

ATOM 1190 O VAL A 74 6.237 11.462 -10.170 1.00 83.23 O

ATOM 1191 CB VAL A 74 5.958 9.534 -7.461 1.00 83.23 C

ATOM 1192 CG1 VAL A 74 7.256 10.310 -7.194 1.00 83.23 C

ATOM 1193 CG2 VAL A 74 5.293 9.378 -6.083 1.00 83.23 C

ATOM 1194 H VAL A 74 3.675 8.629 -8.228 1.00 83.23 H

ATOM 1195 HA VAL A 74 4.858 11.260 -8.115 1.00 83.23 H

ATOM 1196 HB VAL A 74 6.206 8.544 -7.843 1.00 83.23 H

ATOM 1197 HG11 VAL A 74 7.767 10.582 -8.118 1.00 83.23 H

ATOM 1198 HG12 VAL A 74 7.034 11.236 -6.665 1.00 83.23 H

ATOM 1199 HG13 VAL A 74 7.930 9.708 -6.585 1.00 83.23 H

ATOM 1200 HG21 VAL A 74 5.089 10.359 -5.654 1.00 83.23 H

ATOM 1201 HG22 VAL A 74 5.956 8.829 -5.414 1.00 83.23 H

ATOM 1202 HG23 VAL A 74 4.348 8.839 -6.151 1.00 83.23 H

ATOM 1203 N LEU A 75 5.779 9.318 -10.639 1.00 80.89 N

ATOM 1204 CA LEU A 75 6.488 9.299 -11.925 1.00 80.89 C

ATOM 1205 C LEU A 75 5.554 9.423 -13.134 1.00 80.89 C

ATOM 1206 O LEU A 75 6.052 9.432 -14.255 1.00 80.89 O

ATOM 1207 CB LEU A 75 7.342 8.021 -12.065 1.00 80.89 C

ATOM 1208 CG LEU A 75 8.669 7.961 -11.312 1.00 80.89 C

ATOM 1209 CD1 LEU A 75 8.583 8.135 -9.802 1.00 80.89 C

ATOM 1210 CD2 LEU A 75 9.238 6.565 -11.561 1.00 80.89 C

ATOM 1211 H LEU A 75 5.340 8.464 -10.327 1.00 80.89 H

ATOM 1212 HA LEU A 75 7.164 10.152 -11.981 1.00 80.89 H

ATOM 1213 HB2 LEU A 75 7.600 7.899 -13.117 1.00 80.89 H

ATOM 1214 HB3 LEU A 75 6.748 7.147 -11.799 1.00 80.89 H

ATOM 1215 HG LEU A 75 9.326 8.722 -11.733 1.00 80.89 H

ATOM 1216 HD11 LEU A 75 9.555 7.953 -9.345 1.00 80.89 H

ATOM 1217 HD12 LEU A 75 7.843 7.456 -9.378 1.00 80.89 H

ATOM 1218 HD13 LEU A 75 8.309 9.171 -9.600 1.00 80.89 H

ATOM 1219 HD21 LEU A 75 9.393 6.394 -12.626 1.00 80.89 H

ATOM 1220 HD22 LEU A 75 8.539 5.815 -11.192 1.00 80.89 H

ATOM 1221 HD23 LEU A 75 10.177 6.430 -11.023 1.00 80.89 H

ATOM 1222 N ARG A 76 4.221 9.462 -12.970 1.00 76.79 N

ATOM 1223 CA ARG A 76 3.279 9.353 -14.111 1.00 76.79 C

ATOM 1224 C ARG A 76 3.532 8.121 -14.981 1.00 76.79 C

ATOM 1225 O ARG A 76 3.301 8.136 -16.186 1.00 76.79 O

ATOM 1226 CB ARG A 76 3.177 10.684 -14.869 1.00 76.79 C

ATOM 1227 CG ARG A 76 2.392 11.694 -14.020 1.00 76.79 C

ATOM 1228 CD ARG A 76 2.179 13.010 -14.769 1.00 76.79 C

ATOM 1229 NE ARG A 76 3.355 13.894 -14.679 1.00 76.79 N

ATOM 1230 CZ ARG A 76 3.390 15.160 -15.055 1.00 76.79 C

ATOM 1231 NH1 ARG A 76 2.396 15.719 -15.690 1.00 76.79 N

ATOM 1232 NH2 ARG A 76 4.429 15.900 -14.789 1.00 76.79 N

ATOM 1233 H ARG A 76 3.850 9.499 -12.032 1.00 76.79 H

ATOM 1234 HA ARG A 76 2.269 9.167 -13.746 1.00 76.79 H

ATOM 1235 HB2 ARG A 76 2.632 10.533 -15.800 1.00 76.79 H

ATOM 1236 HB3 ARG A 76 4.168 11.071 -15.106 1.00 76.79 H

ATOM 1237 HG2 ARG A 76 1.411 11.273 -13.799 1.00 76.79 H

ATOM 1238 HG3 ARG A 76 2.901 11.883 -13.075 1.00 76.79 H

ATOM 1239 HD2 ARG A 76 1.329 13.514 -14.310 1.00 76.79 H

ATOM 1240 HD3 ARG A 76 1.936 12.803 -15.811 1.00 76.79 H

ATOM 1241 HE ARG A 76 4.163 13.527 -14.197 1.00 76.79 H

ATOM 1242 HH11 ARG A 76 2.420 16.699 -15.932 1.00 76.79 H

ATOM 1243 HH12 ARG A 76 1.574 15.167 -15.892 1.00 76.79 H

ATOM 1244 HH21 ARG A 76 4.434 16.878 -15.041 1.00 76.79 H

ATOM 1245 HH22 ARG A 76 5.195 15.521 -14.251 1.00 76.79 H

ATOM 1246 N CYS A 77 3.982 7.036 -14.352 1.00 77.72 N

ATOM 1247 CA CYS A 77 4.223 5.775 -15.030 1.00 77.72 C

ATOM 1248 C CYS A 77 2.916 5.206 -15.614 1.00 77.72 C

ATOM 1249 O CYS A 77 2.027 4.793 -14.861 1.00 77.72 O

ATOM 1250 CB CYS A 77 4.866 4.807 -14.039 1.00 77.72 C

ATOM 1251 SG CYS A 77 5.609 3.440 -14.924 1.00 77.72 S

ATOM 1252 H CYS A 77 4.181 7.110 -13.364 1.00 77.72 H

ATOM 1253 HA CYS A 77 4.925 5.954 -15.845 1.00 77.72 H

ATOM 1254 HB2 CYS A 77 4.107 4.432 -13.353 1.00 77.72 H

ATOM 1255 HB3 CYS A 77 5.641 5.318 -13.468 1.00 77.72 H

ATOM 1256 N SER A 78 2.782 5.201 -16.940 1.00 65.70 N

ATOM 1257 CA SER A 78 1.681 4.535 -17.642 1.00 65.70 C

ATOM 1258 C SER A 78 2.011 3.046 -17.760 1.00 65.70 C

ATOM 1259 O SER A 78 3.086 2.706 -18.233 1.00 65.70 O

ATOM 1260 CB SER A 78 1.383 5.189 -19.001 1.00 65.70 C

ATOM 1261 OG SER A 78 2.542 5.688 -19.639 1.00 65.70 O

ATOM 1262 H SER A 78 3.557 5.501 -17.513 1.00 65.70 H

ATOM 1263 HA SER A 78 0.775 4.634 -17.044 1.00 65.70 H

ATOM 1264 HB2 SER A 78 0.897 4.461 -19.652 1.00 65.70 H

ATOM 1265 HB3 SER A 78 0.695 6.020 -18.846 1.00 65.70 H

ATOM 1266 HG SER A 78 2.698 6.587 -19.341 1.00 65.70 H

ATOM 1267 N MET A 79 1.104 2.229 -17.204 1.00 53.18 N

ATOM 1268 CA MET A 79 1.086 0.756 -17.076 1.00 53.18 C

ATOM 1269 C MET A 79 2.389 -0.014 -17.318 1.00 53.18 C

ATOM 1270 O MET A 79 2.775 -0.184 -18.490 1.00 53.18 O

ATOM 1271 CB MET A 79 -0.077 0.177 -17.897 1.00 53.18 C

ATOM 1272 CG MET A 79 -1.379 0.146 -17.085 1.00 53.18 C

ATOM 1273 SD MET A 79 -1.361 -1.027 -15.692 1.00 53.18 S

ATOM 1274 CE MET A 79 -3.104 -1.012 -15.210 1.00 53.18 C

ATOM 1275 OXT MET A 79 2.887 -0.509 -16.267 1.00 53.18 O

ATOM 1276 H MET A 79 0.279 2.696 -16.857 1.00 53.18 H

ATOM 1277 HA MET A 79 0.880 0.538 -16.028 1.00 53.18 H

ATOM 1278 HB2 MET A 79 0.157 -0.846 -18.191 1.00 53.18 H

ATOM 1279 HB3 MET A 79 -0.214 0.758 -18.809 1.00 53.18 H

ATOM 1280 HG2 MET A 79 -2.182 -0.148 -17.761 1.00 53.18 H

ATOM 1281 HG3 MET A 79 -1.598 1.147 -16.714 1.00 53.18 H

ATOM 1282 HE1 MET A 79 -3.703 -1.433 -16.017 1.00 53.18 H

ATOM 1283 HE2 MET A 79 -3.241 -1.616 -14.313 1.00 53.18 H

ATOM 1284 HE3 MET A 79 -3.431 0.012 -15.023 1.00 53.18 H

END

ModelArchive (<https://modelarchive.org>/doi/10.5452/ma-6jm8t) > Procedures & Data

SP-B amino acid sequence downloaded from: https://www.uniprot.org

Deposition file:

UniProtKB - P07988 (PSPB_HUMAN)

Input data into AlphaFold:

**>SP-B_Human_monomer:A**

**FPIPLPYCWLCRALIKRIQAMIPKGALRVAVAQVCRVVPLVAGGICQCLAERYSVILLDTLLGRMLPQLVCRLVLRCSM**

Model structural quality of the AlphaFold predicted structure analyzed by PROCHECK (Laskowski et al., 1993, 1996) generated with PDBsum (https://ebi.ac.uk).


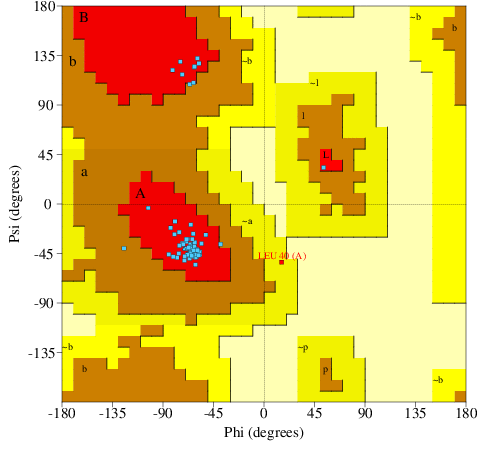


| \| \| PROCHECK statistics1. Ramachandran Plot statistics **No. of**  **residues %-tage**  **------ ------**  Most favoured regions [A,B,L] 64 95.5%  Additional allowed regions [a,b,l,p] 2 3.0%  Generously allowed regions [~a,~b,~l,~p] 1 1.5%  Disallowed regions [XX] 0 0.0%  ---- ------  Non-glycine and non-proline residues 67 100.0%  End-residues (excl. Gly and Pro) 2  Glycine residues 4  Proline residues 6  ----  Total number of residues 79  Based on an analysis of **118** structures of resolution of at least **2.0** Angstroms and R-factor no greater than **20.0** a good quality model would be expected to have over **90%** in the most favoured regions [A,B,L]. 2. G-Factors **Average**  **Parameter Score Score**  **--------- ----- -----**  Dihedral angles:-  Phi-psi distribution 0.37  Chi1-chi2 distribution -0.68*  Chi1 only 0.26  Chi3 & chi4 0.69  Omega -0.47  -0.06  =====  Main-chain covalent forces:-  Main-chain bond lengths 0.55  Main-chain bond angles -0.15  0.14  =====  OVERALL AVERAGE 0.04  =====  **G-factors** provide a measure of how **unusual**, or out-of-the-ordinary, a property is.  Values below -0.5* - unusual  Values below **-1.0**** - highly unusual  **Important note:** The main-chain bond-lengths and bond angles are compared with  the Engh & Huber (1991) ideal values derived from small-molecule data. Therefore,  structures refined using different restraints may show apparently large deviations from normality. \| \| --- \| \| \| --- \| --- \| \|  \| |  |
| --- | --- | --- | --- | --- |


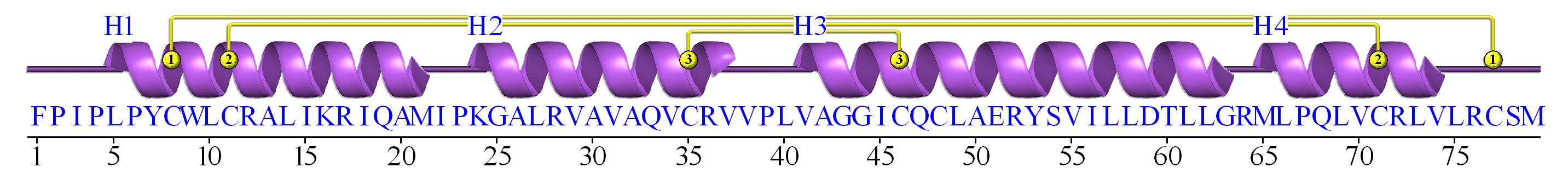


| Disulphides |
| --- |
|  |
| \|  \| \| \| \| \| \| \| \| \| \| \| \| \| \| \| \| --- \| --- \| --- \| --- \| --- \| --- \| --- \| --- \| --- \| --- \| --- \| --- \| --- \| --- \| --- \| \| **1st cysteine** \|  \| **2nd cysteine** \|  \| **Type** \|  \| **Chi1** \|  \| **Chi2** \|  \| **Chi3** \|  \| **Chi2p** \|  \| **Chi1p** \| \|  \| \| \| \| \| \| \| \| \| \| \| \| \| \| \| \| A 8 \|  \| A 77 \|  \| RHH \|  \| -84.3 \|  \| -109.8 \|  \| 131.5 \|  \| 85.5 \|  \| -163.5 \| \| A 11 \|  \| A 71 \|  \| LHS \|  \| 176.3 \|  \| -81.8 \|  \| -97.7 \|  \| -64.4 \|  \| -61.3 \| \| A 35 \|  \| A 46 \|  \| LHS \|  \| -71.2 \|  \| -66.8 \|  \| -74.2 \|  \| -67.6 \|  \| -166.5 \| \|  \| \| \| \| \| \| \| \| \| \| \| \| \| \| \| |
|  |
| Number of disulphides in chain A: 3 |
|  |


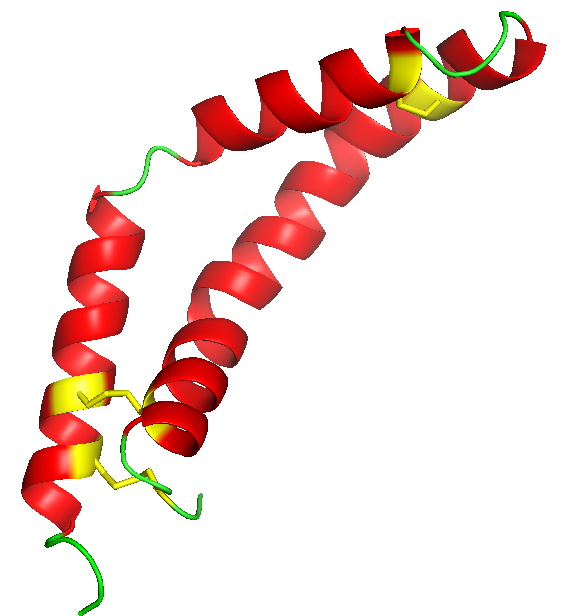


**Human SP-B Monomer Structure Predicted by AlphaFold. Helical domains in red, disordered and bend structures in green and disulfide linkages in yellow.**

**Human SP-B Monomer Overlaid with SMB Molecular Dynamics Refined Structure**

**Superpose Stats**

# Program: needle

# Rundate: Mon 29 Nov 2021 16:29:56

# Commandline: needle

# [-asequence] /var/www/html/superpose/html/tmp/super_1638203396/alignfile0.fa

# [-bsequence] /var/www/html/superpose/html/tmp/super_1638203396/alignfile1.fa

# -gapopen 10.0

# -gapextend 0.5

# -outfile /var/www/html/superpose/html/tmp/super_1638203396/nicealign.needle

# Align_format: srspair

########################################

#=======================================

#

# Aligned_sequences: 2

# 1: PDBA_model_default_chain_A

# 2: PDBB_model_1_chain_A

# Matrix: EBLOSUM62

# Gap_penalty: 10.0

# Extend_penalty: 0.5

#

# Length: 79

# Identity: 41/79 (51.9%)

# Similarity: 41/79 (51.9%)

# Gaps: 38/79 (48.1%)

# Score: 198.0

#

#

#=======================================

PDBA_model_de 1 FPIPLPYCWLCRALIKRIQAMIPKGALRVAVAQVCRVVPLVAGGICQCLA 50

||||||||||||||||||||||||||||

PDBB_model_1_ 1 FPIPLPYCWLCRALIKRIQAMIPKG------------------------- 25

PDBA_model_de 51 ERYSVILLDTLLGRMLPQLVCRLVLRCSM 79

||||||||||||||||||

PDBB_model_1_ 26 -------------------GRMLPQLVCRLVLRCS- 41

Local RMSD

Alpha Carbons Back Bone Heavy All

RMSD 4.36 4.27 5.37 5.58

Atoms 41 163 324 478

Structure Residues

PDBA chain 'A' 1-25, 63-78

PDBB model '1' chain 'A' 1-25, 26-41

Global RMSD

Alpha Carbons Back Bone Heavy All

RMSD 4.36 4.27 5.37 5.58

Atoms 41 163 324 478

Structure Residues

PDBA chain 'A' 1-25, 63-78

PDBB model '1' chain 'A' 1-25, 26-41


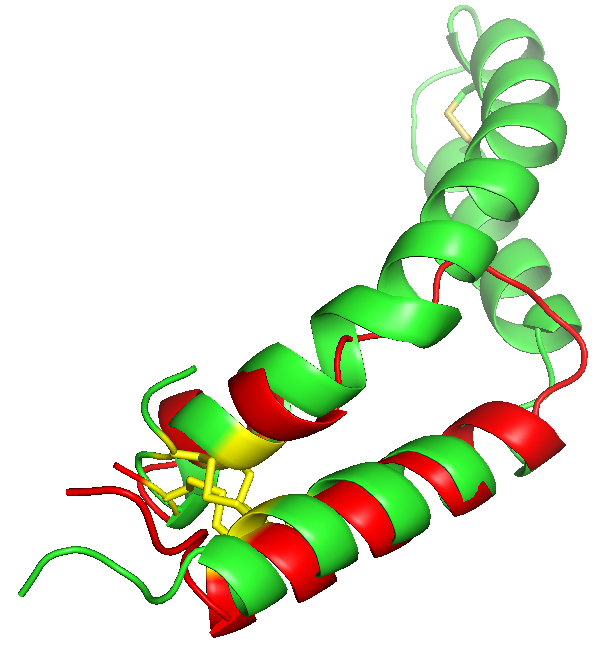


**Human SP-B Monomer in green highlight Overlaid with SMB (red highlight) Molecular Dynamics Refined Structure. Disulfide linkages shown in yellow.**
